# Supplementary material for: Expanding the Repertoire of the Plant-Infecting Ophioviruses through Metatranscriptomics Data
Source: Viruses. 2023 Mar 25;15(4):840. doi: 10.3390/v15040840 (PMC10144540; doi:10.3390/v15040840)
Supplement: Supplementary file 1 [file viruses-15-00840-s001.zip › Supp figures.pdf]

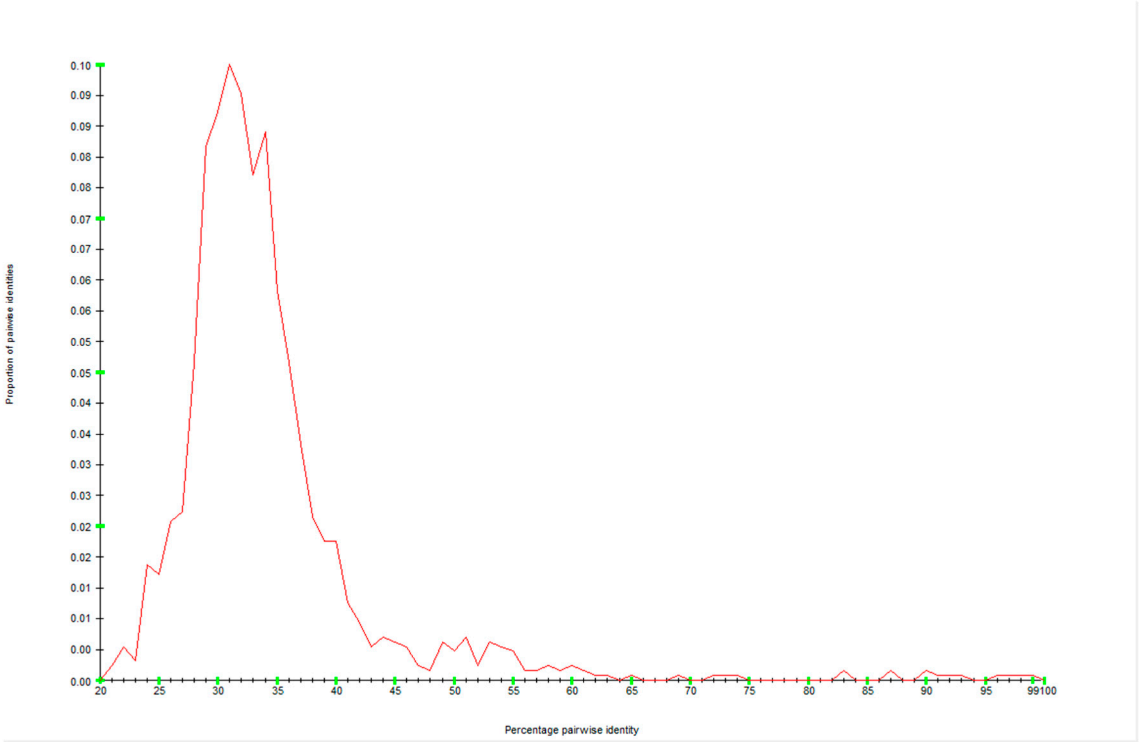

Supplementary Figure S1. Plot of frequency of percentage pairwise identity of ophiovirus complete capsid proteins generated using SDT v1.2 software based on MAFFT amino acid sequence alignments.

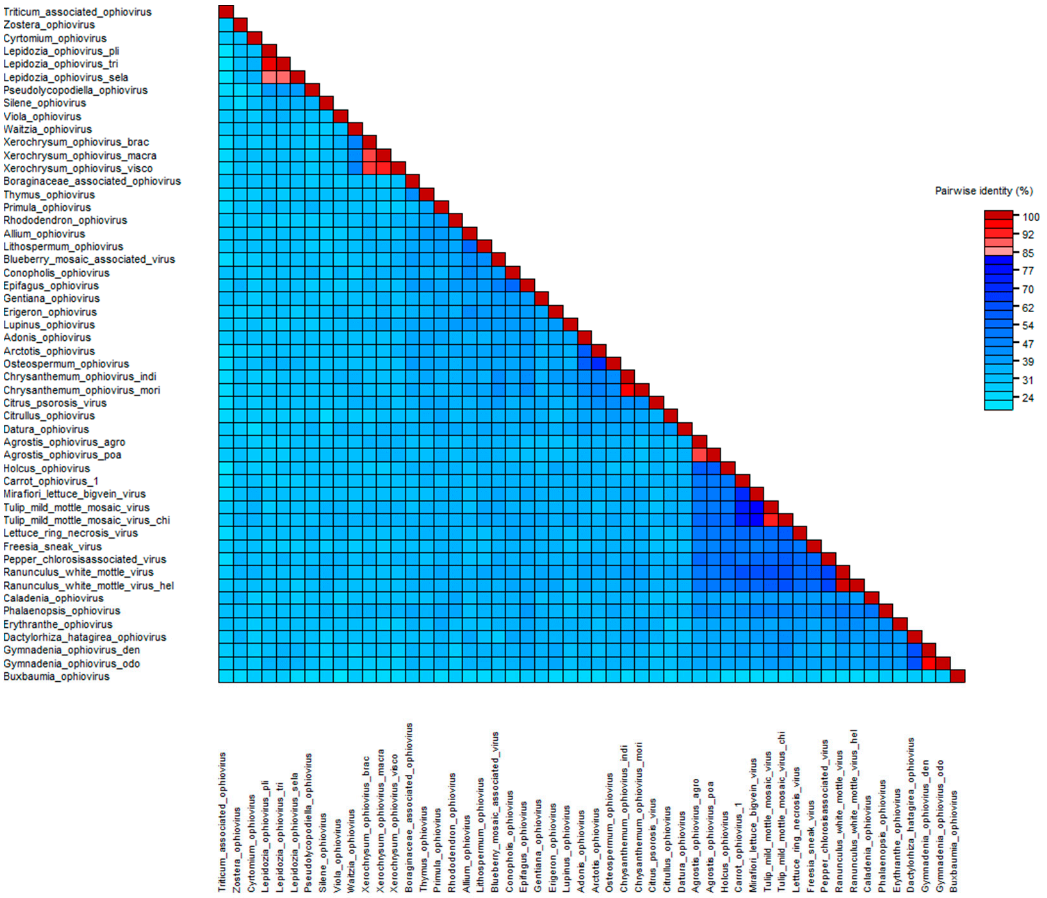

Supplementary Figure S2. Pairwise identity matrix of the amino acid sequences of the ophiovirus complete capsid proteins generated using SDT v1.2 software based on MAFFT alignments. The colored cut-off is based on ICTV demarcation criteria of ophioviruses, which includes CP amino acid sequence identity <85% to be considered novel species (blue-light blue).

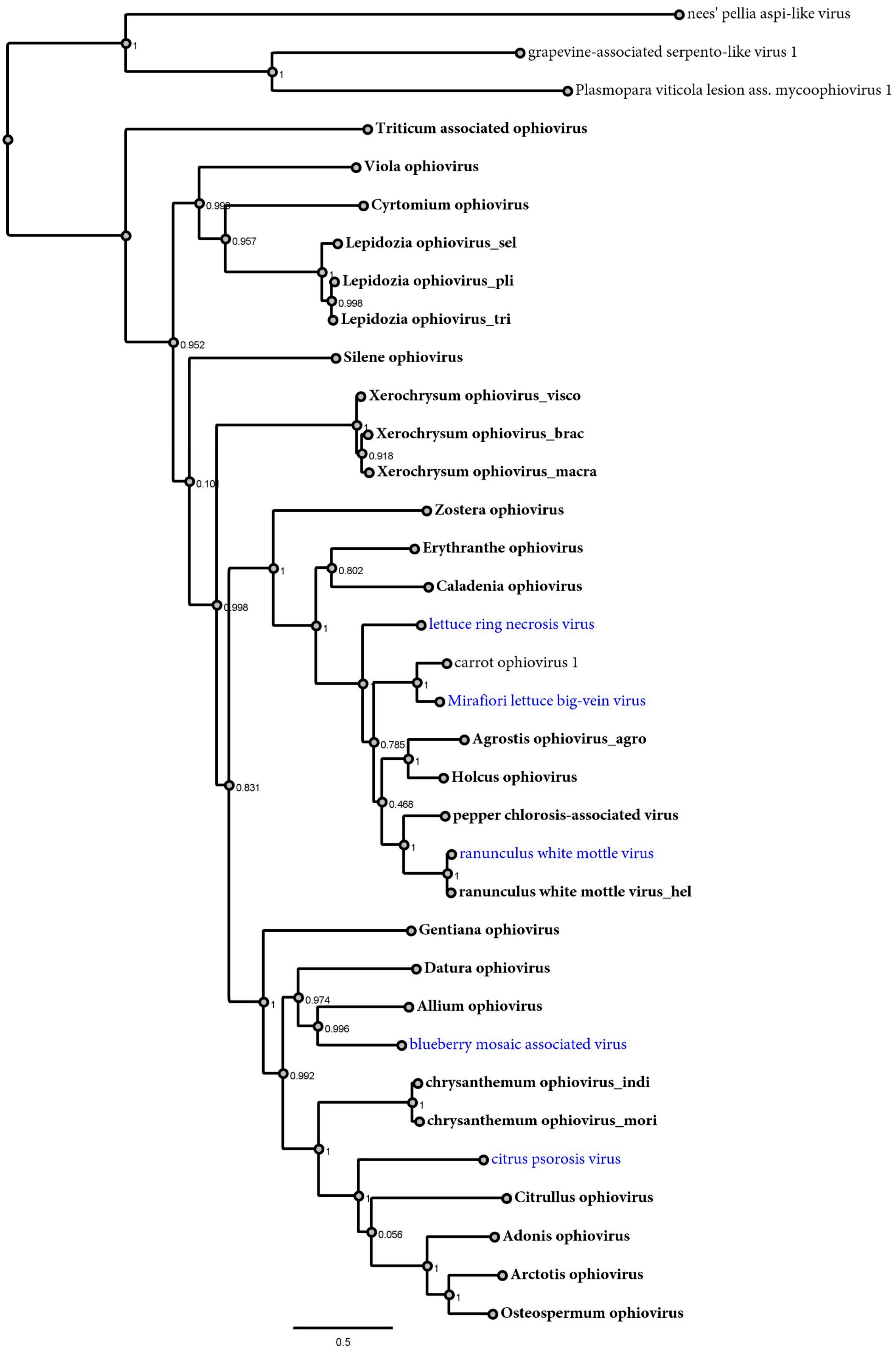

Supplementary Figure S3. Maximum-likelihood phylogenetic tree based on the amino acid MAFFT sequence alignments of the RdRp protein of all the ophioviruses reported thus far and in this study. The

scale bar indicates the number of substitutions per site. The node labels indicate FastTree support values. The RdRp proteins of three related and unclassified aspivirus-like viruses (nees' pellia aspi-like virus CAH2618860, Plasmopara viticola lesion ass. mycoophiovirus 1 QJX19787, grapevine-associated serpento-like virus 1 QXN75438) were used as outgroup.

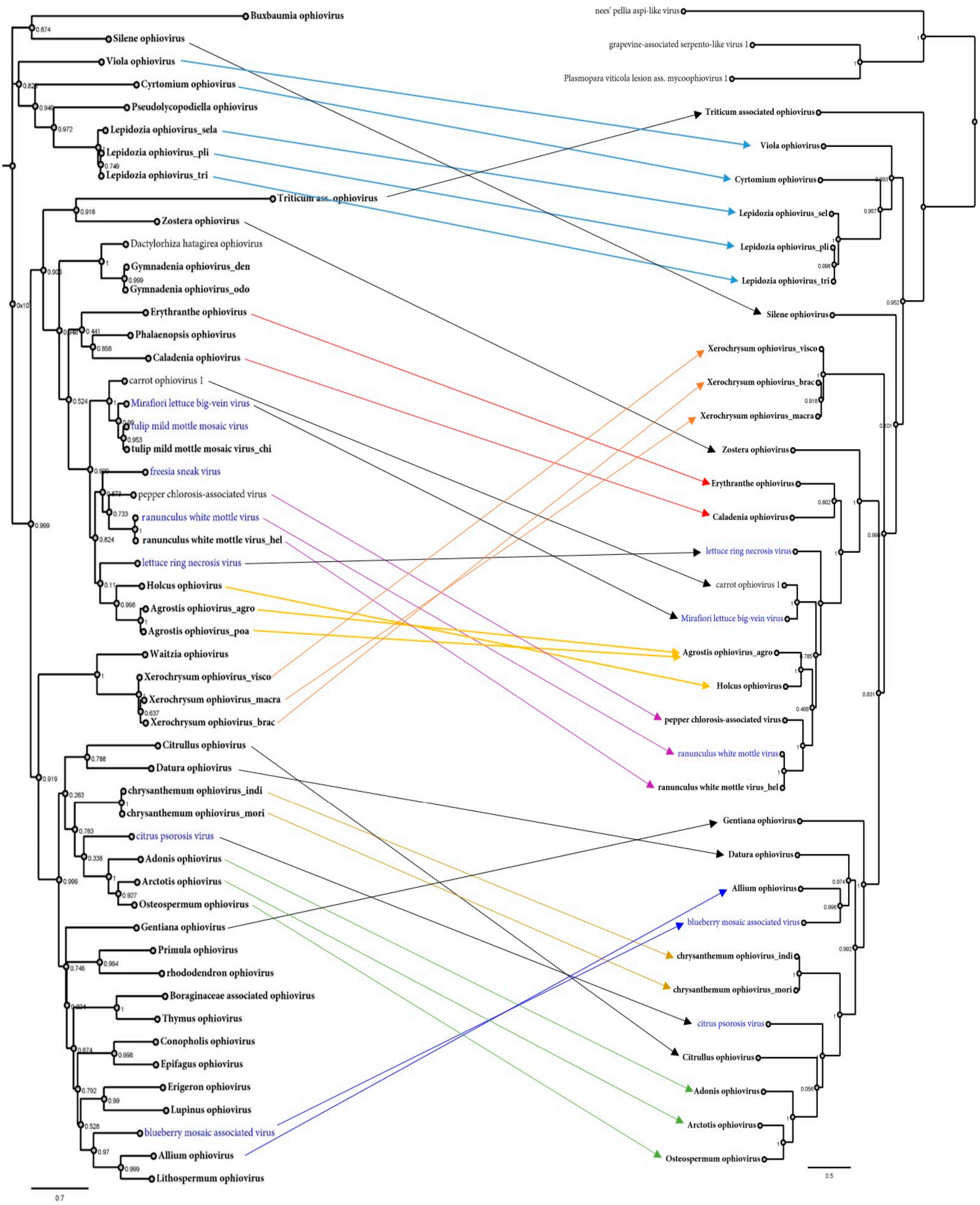

Supplementary Figure S4. Tanglegram contrasting phylogenetic relationships of the ophioviruses predicted with the CP protein (left), against an L protein maximum-likelihood phylogenetic tree shown on the right. Links of well-supported clusters of viruses co-diverging in both trees are indicated in colors. Viruses corresponding to members of ICTV recognized species are depicted in blue. The scale bar indicates the number of substitutions per site.

>Seq1 [organism=Adonis ophiovirus] RNA1 partial sequence
CTTCCTTCCTCGTCCTCATACTCATCAACCCCCTCGGTTGACGGGGATCTGTACTCCTCTTTGC
GTATCATTTCCTGTTGTAGATTACATCAATCTGAGAAGTAGCAATCATCATCAAATCCAAATT
CCTCTCTCATTTCTTCAGCGGTAATGGTTTGATTTCTCTCTATTTCTTCTCGGGAAGTTCCTAT
CAAACCGGCAACCTCTGATGATATCATTTCAGCCATAACTTGATTATTTTCTTCTATGGAGCT
TTGTTCTGCGTATTTGCTTTTGGCCGATTCAGCACTAAAATTTTCTTTCTCGAATTTCATGCAC
CCTTGCAAGCCTTTTCCCAAGTTGCTCAAGAGTTCGTTCCCCCACTGATGATTCACATAATCC
TCAAACTTTGTGAAAGATCTAAGTGCCTCTATTTCAGTCCTCATCTCATTCTCCAATTCTTCCT
TGGATTTGTTGATCTTCTTCCTTTTGGAATAATCTAATCTTATTGTTTCATAATCTCTGTTCTTT
TGTTTTCTCATTTTCTTCAATTCCATGAACTCAGATATCATCCNNNNNNNNNNNNNNNNN
NNNNNNNNNNNNNNNNNNNNNNNNNNNNNNNNNNNNNNNNNNNNNNNNNNNNNNNNNNNN
NNNNNNNNNNNNNNNNNNNNNNNNNNNNNNNNNNNNNNNNNNNNNNNNNNNNNNNNNNNN
NNNNTGCAAGCTTGAGAAAAGCAACTTCCCCGTTTCTTCCCAAATAGGCTTTCTTAACCAAT
TTTGAAATCCTGACTAGTCTGTCGAACGAGTGATATTTTTCTCTAAATGAAGCACCAGTCTCA
ATGCTCATTATCAGTTCTTTAAAGTTCACCTGTCCATCTTCAACTTGCTGAAGTCCCTTGTGT
ATTGCTTTCCATGATTGATACGCTTCAACAGAGTTCCAACGTTGGTTTGTCAAGTGTTCACTG
AGATGTTCGAGTTGTTGTTTCCTACAATCTTTTATATCGACCACGAATTTGCCTGAATTCTTG
ACGAATTTTTTGTCAACAAGCACTTGTGGAGATAAGTCTTTTTTCTCCTCATATTCTTTGCTTA
ATATTTTTAGTAACTCTTTCTTGGACACCCCGTTCACTCCGGTTGTCAACTTAGGAACGGACA
CAATGAGCAAGTCTTCCATCTCCTTTTCCATTGTGACCTTGATCTTTTCTTTCTCTTTGGCTAA
GAAGTATGTTTCCTCTAATTCGGTCACCATAAGATCTCTCATTCTTTGGCCTTTCCTAATTTTT
GATTCTATTTCACCTCCCAGGAAGTTGTCATCTTCTTCTCTTTCCAGCAATATTGTCACCAAT
GATTCGTCAGATATGAGCTCCACTGTGTGGTCGGACACATCACAAGGTTGAGAATTGAAAAT
CGAGTTTCGCCTCATCTTGAAGATCTCATTTGTCAATTTTTCTGCTATTATCGATTCAGTGAA
CGATCTTTTGGCGCTGGTGTCTGTCGCTTTTTCTTTTTCTTTCCTCTTTTCCTTGCTATTACCGG
TCGATTTCTCCAATGTCAAGTAAACATATCCTGGGGATTCAACTGAGGGAACTAGCAGTTTA
GCCGAACCGCCTCTCGTTAACTCTTTCAAGAAACCAATGTCACCTGACAGCAACGAGTTTAT
CCGGAGCACCACCACTTTATTCGAGTCGTATAGATCCTCTATCATGGCCCCAAATGATGACT
TTTCTCCAGAAAAATGGGACACGTCAATCAAGAAGACGTCTCCATCTAGATACTCTGAGTAG
TTTTCTCTCTTAGTCATGTCGATCGATGATTTGATCTCCTTCATACCCGGTACACGGTTTATGA
GATTATATCCATCATTTCTGGAAACAGACTCATGTTGAATTCCAGATTTTGTCATTGCAAAGT
GAAAGTCTCCCCTACCTCCAAACAAGTCCACCACTTTCTTCCCTTTGATCTTTTCTCCGAGAA
AACTCAACAATCCAAGCGCTGATGGATAGACATCTGACATCGTTGGAGAAGACAAATGTTG
TGGCTTACAATGCAGGGCACATATCATTTCATAGCTCTGTGCAGATACCATTTTTTGTCCTGA

GAAAAAATCGTCATCTACATCAATCTCTTCATATGATACATGCCCCACCTCCTTCTCAAATCT
TTCACTATTGACATAGTCCTTGATGTCCGCATCATAGTATTGTACCAACATCTCAGTTTCAAA
TACGTGTCCGTTCAATTTCTCATATAATTCTTCAAGGCATTCAAACATCTCATCCATTCTATA
ACCACTGGTAGGGAAGGCGAAGAGTATCAATTCTGATATCCTTTTGTCTTTTATGTGGAAAT
GGGCCTTCCCCTTCTTCAACCGTGAAAGATTGTTGACACCATTTGCTTTTGTTAAGTCTTTCTT
GATTATAAGTTTCATGGATTTTGCTGACTCCTCTTCCAGTTTGAACACTAAGCAAGTCGATAT
GATGAAGACTTTCATCAGAGTTCTGCGATAGTCGACAGAATATCCGAATTCACTGATCTCCG
TTATCCATTTCGTCTTCTTAAAAGAATCTCCAAGGAGCTCAGTGTTTATTATGCTTCTCATCA
CGGTTTTTGTGTAGCTCTCGTTTCCAATGATGTTCAGCCCCCTATAAAAAGAATAGAACTTGT
CGAGATAATTGTCCCTGTTTAGGCTCATGTTAATTTCTGGAGCGATCTTGGATAGTGTGTCAT
CAGGCAAGAGGTCAGGGGAAATTATCTCTTGGTCTTCCATGTATTTGTGGAGGCTCTTGAAT
GAGGAAATTTCTGAAATTCTCTCGTCTGGAAGAACTGACTTGTGATCAATAAACTTCCCCTTT
ATGTCATTATCGCATGAGATGAAGTATGAATAGTTTCTGTACAATCTCCCTTTCTCCGTGATC
CTTTTTGTTTCTAAACTTTTTGGGATTTTGTTGGGAGGAGGCAAATAGAAAATCTCCCTACTA
TCTCTTATCTGGTCTATACAAAAAGTTACATCCTTTGCATGCAACAAAACGTCGTCAGAAAT
GGTTAGTGCTCCCATATCCCCGTCTGTTGCTTTCAGGTACCCCATTACAACCTTAATATATGT
TATCAGCAAGGAAACATTAAGATTGTTGTCTACACCCCCAGTTAAAGAAATCAATCTATTGA
TTCCTGTCACCTCGTAACAATTCGATCGAATGTTGGAAGTCAAATCCCCTGTTTTCGGGTTGA
AACCCCCTGACACTGCCCTGTGAAAGTACCTTCCGCCTTTTGGACAGACTACGTTTTCGGTGA
CATCAAAAACACTGCAATCGGAATACGTCTTTATCGTAACATCCATTATTTTCATCAAAGAA
TCTATAGAAGACTTTTTGATGCTTCCAAACTTTTCACAATCCATGATTATCCATTTTGAGTAT
CTCACTAACTCATATATCAAGAATATGAGTTTGTTGGTGAACATGCCTTCGATTTCGATGTCT
CTATCAAATTTCGTCGAATTTACATGGGTTTTTGCAGGTCCAGCCAGCCTAAACCGGTTTCTT
TCCGTTCCAGTCCTCACTCTTCCACTCTTTCTGATCACTGAGATCAATCCCATGTCATAGTCG
CTATTCACTTTTCCGATTATTGGCAACTCTTCTATGGACAAAAATTTTAGAGTGACTGGAACT
CTCTCTCCTTTTGAATTGAACATCTTCTTTTCCTTTCTTTCTCTTTCTTGAATCAACCAATCAA
ATCCGACGCCCTCAATTCTATCTTTTGAAAACCTGAATACAATATTGTGATTTGCGTTCCATG
CCTTGTTCCACAGCCTATTCATCTTCTTTTTTCCCAAAAAGAAAGAAAGTGTCGTGGAATTGT
CTATTTTGGAATAGATGTCTTCTACAAACTTATAAACAGAACACTCTATAAACTTTCCGGTTA
TCCTGTGTGACAAACAAAATTGGAATGTGGATAAGAGTTCTTGTTTAAAGGAGATTTCATCC
TCTGTTTTTGACAGATTTAAGATCTCCTTAAGGTTTGAGTTTGTGACCTTGTTTTTTAAGACTC
TCTTTAATTCATCCTTGAGAAGAGTCTTGGTGTCAAAACGTCCTCCTGTCATGGGGAAGGGC
TCATTTAAGTAACTTGTGGCATTGTCTGATAAATTGACCAGGTCATACAGTTTTGATTTCAAT
TTATCGCCAAGCAAAGATTCCACATAACACAGTCTTTTCACGTTTGAATAAGAAAATCCAGA
TATCATACTGTCTGGTATGCTGGTCATGGAATATCCATACATGGTATAAGGTAAGTACAACA
TCATGAGGGTCGCTTCCAGTAGTTCGGTATTCTTCATGTTGAAATGGAAGAAGGCTGATAGC
AGCTCTGATTCTAATGATCCTCGTTCAGAATTTATTACTCCTATCATTTCTTCAAAATTTCCAT
TTCTTTTCTTCAGTTTTCCCAAGCAAAGTTCTGCAAATTTCACCATTGCGTTGCGGTTGTTTAT
GAAGTTGCAGTCCATGTCGAGTTCAAGTGAATGAGACCATCTCACGATTTCCTTCTCTATGA
CAGCAAGAGCTCTGTAATTCCTTATTACAACTTGTTTTCTTATGTCGTCTGATCTGAGCGTGG

ATGACGTGAATCCGCTGTCAATCGTCATCATTCTCTCAAGATCATCCCCCCACAACACCTCAT
TGTTCGGGCTAATAGCCAGGACCCTCTTATATGACATGTCAATTGGCACATCCTTATAATAGT
GGTTTTTGAGCATAGAGCATCTCCCATTGGTTATCTGTGTCTGAGAAAGCTTTACCAAGAGTC
CCATTTTCAACGCATAGGCTTTCACGTAGCATATGATCTCGTTAAGGCTCCTTGGATTTAATC
CTGGCTTGACAAATACTCCACATGAATCGTCACTATAGGTGGTTCCTATATATTTCTTGAAGT
TGAGCGACTTCATCATATCTTCCATCATTAGCTGCGATTGGATTCCCCAGAGTGGACCAAAC
CAGCCTTCAATTGCTCCTTTTTGATGCCTTGATATTACTATGTTGTCAGACATTAACTCCTCGT
ATAAAATATGTATGTCTGAAAAAAGGTAGACAAGCATCACCATTCTTTTCCTTTCTTTTGATC
CGTCAGTGAACCCATACATGTCAGAGATTTCCTCCATTATAAATAGGTTGTTTTCTGGCCTCT
GCGAAGTGTTGTGTCCTGAATAGTCTAGGAATAAAGAATACGAATTCTCTTCGTCTAATAGC
CTTGACATCTCATGCATTATTTCTCTTCTCTCATCTTCTGACATAGTCATCATTTGGCCCGGCA
CCAATTTCATTGCTCGTTTGATCATTTCCATAAGGACTGATATGTAAATTTTCAGTTTGAAGC
TTGCGACACCATAAAAGCGCGCTTTCGTCTTGAGCTCTTTTTCTTTTTCCACCAACCTCACTA
GCTTAAATTTCTCAATTTCCCCTATCTTCTCTTTGTCTTTCCTGTCAGCCACTATTATCTCTCTA
TCGAAGTGTTTTTTGATTTGACTAATATACATATCTTCGACCAGCTCCGGTATGTCATTGTTG
GTGATGTAGTAGATCAATTCTTTGACTGAATTCCCTGTGAAATCATACTCATCTTTTGTGCAC
GCCTTGTCTATCACTCTTGACGCTATGTTCACTTCTTGGCCCGGTTGAAGCATCTGTCCCATT
CTTATACTCTTGTAGTTGATTTGATTTGAGGTTATATCAGAATTGTAACTCCCTCCTGCAGCC
ATCAGATTCAGTTCTTGTTCCAACATTCCGTTCGATCCTATCAGGGGGGGAACCATTCCATAT
TTTTTAATGTAATTTGTTATCACTTTCATCCTGAATAAGTGTCTTAAATTTTCAATTGATTCCT
TGCTCACCGGATGATTTCTGTTAGTCCTTTGTATGTATTTTTCTATTCCTCCAGCTGTGTCTAC
TTCAGCAAATGTGTTGGCTTTGTTTATGCCCGACATTTTGATGCAAGTTTCCGCTGATGAATA
CCTGCATGTTTTTATCAGCTTTTCCGTGAACACTGGGTCAACTTTTACAGCCTCTAGAAGAGC
ATCAATCAGCGGCAAAGTCGACGACACTGACTTGGTGTCTGCCATCATCAAACAAAGGGTTT
CATAGGCTGCAGCCATCTCGGTTCTTTTTGGGTTTTCCATATTTTGGAGAGAGATGATTAATT
TTATGAAATCATTTTCTTCTATATTAGCGCTGAATTTCTCTTTGGAATTCATGTCCTTTATCAG
CGACAAAGTGCATTCGTTTTCGATGACTGTGAACAAATATTTGATCATTGTGTTGTTCCCGAT
GAATACTATGGGCCCTATCATGATCCCGGTGAAAGTCTGGCTGATGAAAAGACTAAACTTGA
AGTTCTCTTCTCCTCTAATTTCTATTTCTGAAGTGCTTGAACCGTCATTCGAAACTTCACACTT
CAACTTTCGGTTCTCGTGCCATAGATCCGCTGAGGCATATTTTCTGTTGCTGCTTAGGCTTGA
GTAGGTCAGTTTAATCTTGTGGTTTATAACTTCAAATAAGTTTAGCAACCACATGAGCTTTGT
AGTCTCCACTCCTAGTTCTATTTTTTCTGATTCTATGGAGTCGGGTTTCTTCTTTGAATGTTTG
TTGAACAGCTGTTCTTTCAGTTTTTGTTCTTCAAACATTCTTAGTTCGTCAGAAAATCTGAAC
AAAGTCTCCATCCTGGTCGACGGCCATGAATTTCTTTTCAATTCTGAAAAAACAAAAAGCAT
ATCATCCTGCTTTCCTGAAATAGCTGTCTTTCCACCCACAAATTCCCTTAACCTCTTCAAGTC
TATCAGAGACCTTATGCCAAATTTTTCCGGGTACAGCTTATTTCTGACCAAGTTATTCAAGTT
CTTTTGAGATATTAGCTTGAAGGGGGAATCGATCCTAGACATCAGAGCTGGTTTGTCAATAT
ATGTTTCGCTCACGTACTCTCTTTCCCACACAATCCAATGAGAGATCCTTGATTCATACTTTA
CTTTCACTTTTTTCGGCTCCAGTTTTTCTTCGTCTTCTTGTGACACTCTGAGATCAATGAAGTT
TTCTTTTTCATCCAAAAGCATGGACACTGTGCAAGGGCAACCCGTGATTTTCAGCTTTTTGAT

CAGAGATTCAAACTCTTCTCCATACTTTGTCCTCTGACTATGGGAATCTTCTGCTTCGTTCGC
ATGATTTTCCATGTCACCTTCGTTTGGCGTTGGTTCTTCTTCAATCTTCGTGGCAAAAGTACC
ACTCTTCTTTTTATCCTCTACCTCTGCTTCATCAGCCCAATTCATCTTCTTATTTTCCTTGTTGA
TTCCGTTGTTTTTCATTTCCTCCATAATTGTAATTTTTGGCTTGTTTCCTTCTTTCCTTCTTTCTT
TGTTCCTTTTCTTTTCTTTTTTCTTTTCCCTTTTCTTTTTTCTTCTTCTTTTTTTTCTTT
>Seq2 [organism=Adonis ophiovirus] RNA2 genomic sequence
GTCCACAGGCCTTCTGGCGAGTGGTTTGGAGCGAATATTGGATCTCTCAATCCTCACACTCA
AAGTTTTTATTAAAACGAAAAAAGAAGCAGTCTACCGACCTGCAAATGCCATTGTCTCCTGC
GCAGAAATATATTTCTCATCACCTACCAGACTTCATTTTTCCCTCCTTTGGTCGCCTCATTCCA
CACATCATCCTTCAGCCACATTCTTCCCGGCTGTTTGATGTCAAACATCATCACTATCTCCCT
GTCTTTATAAAGGAACAGCGCGCTCCCAGCAGATTCCATCGAATCAACCACCTTAGCGTCTT
CCAGTTTCTTGTATGACATTATATTAAATTTTATATCCTCAGTGTTGAACAGGAAAGCCTCTG
GCAGAATGTGACTGTTTCCAAGCACTTCCAATCTCACTGCTGCTTGTTCTACTCTTAAAACTT
CCACGCCTGCAGCGTTCTTGACCTCCCTGTTTGGCTTTGCCCCTGAAAAGTAAAAGTAGTGGT
CAGGGGACCCAGTGTCCAACATCGTCATCATACCATTTCCATTTTTTATGGTGGTTCTTACTG
CCGTTTCCGGTTTGTCATCCTTCCTCCAGACTCTATACATAGCTGGAGCCATTATCTTCTCTCT
TTCCTCCTCACTCTCACTTGAAACTGTTTGCTTCGATTCATATTCTATCAGCAGGTTTTTGTTC
CTCCTGGCTGATTCTCTCCTCAAGGTTCTCTCCTTTTCTGAGAATTTGTTTCTCTTTTCTTTCAC
TCTTTCTTGCATCTCTTCGCCGATCTTTTTGAATACTGAGCTGCTGTTAAGGTCCTTAGGTCTC
TTTGCTGGGTCAATATACAACACCGTTGAAGCCCTAACATCTGTTTTCATCGGAATACTGGC
AGAGTGAGTGAAAAACGCCACATTAAGAGCAGCATATGCTCTTCCCACTATTGGAACTCCTT
CTGCTTCAATAAATATACTTATTTTCTCCATATCCGTCTTTTTTACAAAGAAATTCATCGACA
ACTCAATCATGGCCATTTCTGAAGCATCAAATTTTACTTGCTGGATGACTTCATCTTTACCAG
CTTTTTCCTTTCCATCATCTATGAGTGAAATGGTGAGAGTACCATTATCTTCCTCATCTTTGTC
AAAGAGTGGAAGATACATAAACTGGATCTTGTCAAATGTGATGAAAGGGCTGTCTTCGATTC
CTCCTATCTTTTTAAATGCTGAAACTATCCTTTTCATGGTGGCCAACTTAATGCTTTTTCTATG
CTCATTTTCTTTCAACTGCAATGTGAGGGGGACAATCCCTGTATCTATCACCCCTGTGTGGAT
CTTCGAAAAGTCCATCAATATCTCCTCAATTCCTCCTGGGAGCTCTGATTCTCCTTCTTTGAG
AGATATTACGCTCACTTCACCTGATTTGACGAAACTCTGCCCAATAGACTTTGCTTCAATCCT
CTTCGGCCGAGAAATTGTCCCGTCATATCTGTTGAGAGAAGACATTTCTTCTATTCTGTTAAG
ATCACTGGTCCTTAATGCGCGGGATAGTGAGTGCATCAACTCTTGGTTCTCTTATCAGGGTTG
CAAACGGGCAGATCTGTT
>Seq3 [organism=Adonis ophiovirus] RNA3 genomic sequence
ATAACGAAGCACAAATGCACCACCATCCACACTGCACCAGCAATCAAGCATGGATTTTGTA
AATGTTCTTCAATCCCTCTACTGAAAGACCACTGAAGTCAGCATCTATATTTTTCAATATTGG
CCAATCTCTCTTCTCAGCATCACCGGTTCCGAAGAAATGAATGTCTCTCTTGAAAGCTTCATT
CTTGTCAGAAATGATTTTTTCTGCCATGGCTTTCCTCCCGCTTTTACTCAGAGAAAAGATTAT
CGCACATGTAAGCTTTAAAGTGAAGTTTTTTGTAGATGGTTTCGACTCTGAGAGTGGATGCA
TCACTAACTGATTTTTGTAGCAACCATCGAGAGAAGCCAGTGTTTCTCGCAGCTCCCTAGCA
AGTTTGATTTTCTTGTTTTCCTCCATCTTTTGATACATTGCACTCACGGGAGTGTCGTCACTTA

TAGCCAATTCCTCCCGCTTGTCGAAAGAAGCTGCATACACTGCATACCGAATACCTCGGTTA
CCAGCGACATTCAGTTTACACCTGGATGCCACGGGAAGTGGCAGCAGGTCCAAGTTCATTTG
CAACAAAACTCTCGCAGGAAACTTAACCGTACTCACGGCACTGAGATCCTTCGCAATTTCAT
CCATAGAAATATTCTCTCCCTCATAAAGTTTATCGATTATGAAGTTCGGAACCTTCCTGTCTT
CATGAGCTTTGTTGCCTGGCAAGCTTCCTTGGACAAATACTAGCACTATTCCTGCCTGTAGTG
CTCTCTTCCCTTTCTTTGTGTTCATCACTATTTCCCTCTCAGTGATCCCTTCCTTCACTTCTTTT
TCTACAAAAAGACCATTATTTGCAGCATCTTCCCTCATGCTGGCAAAAGCAATTGCGTCTAA
TATTTTCGTTCCAGCAGCAATCAACATCGGAACATCAGCCAATCTATCCCCAAAGTGGAAGA
TTGGGAGATCACCTTGCTTGTATGTTTCATCTTCTAATCTGATAGTTCGGACAAACTTCTCTA
GAGTGGCCTTCAACTTTTCCATATCCATAGTATAACTCTGCTGGTCGATAGCTTTGGTGATGT
CTTCTTTGCTGGAGAGAATTATGTCCGATGACCTGGCCACATCCACCCTCATGCCAACTCTAC
TCTCCTCCTTAGGTTCTGGGGCTAGGGAGGCATCCTTGGTGCGGGCAATAGCTTGTAGTTTTC
CACCAACTAACATCACTTTTGCATCAGGGGTCTTGTTTTGAATATCCTCCATTAAGATACTAT
TCTGAGTGTTGTATTTTTTATCTTCCGTGATGAGACCATATTCCTTAAGTTTTGCGACAGTTTG
AGGTTCAGCTTCTTCCGAATCTACACTGAATGCATCAAAAAATTTCTTCAAGGATGACACGG
CGATTGGAGACGACATCTTGGGATTTTGGGAATTTGTTTACTTTTGAAGACTTGCAGGTTTGT
ACTTG
>Seq4 [organism=Agrostis ophiovirus_agro] RNA1 partial sequence
CTGAACTAAATCAGCGGGTCGCAATGACCGTAAGGTGAAACGTCTAACACAAAAATCAATT
GACCACTTCAATATCCTATTCATCATCATCACAATCTGCCCACATCCCCATTATCTCATCCTC
GCCATATTCTTCCAACAAGTCATTGAAAGTGCTCAACCTTTCATTTTCCCGGATTTCTTCTTCT
GTTGCCATGACTCCCATGCCTAATATGACATTAGTCATGCTATCAAAGTCTGACGTTGTGTTC
TCTAATCGATCAGTCAGAGCAGTCAGAGCCCCAATAGCATCATTGAAGTGGTTCATAAAAGA
AAAATATTCTTGATCAAGTTCAAAGAACATCTTGTCTAATTCCTCAGCAGTCATCATGTCAAC
ATGATATCGAGACATATCAATGTCTTGTGGATTCTCTGAATATGAATTCCATATTTGANNNNN
NNNNNNNNNNNNNNNNNNNNNNNNNNNNNNNNNNNNNTTCTTCAGGTAAATTTTTCACGA
TTAAAAGTATTCGATTCATATATAATTTATAATGAAGTATGTTATTTCTTGTTGTTTTGTCTTT
ATGGTTTAGTATTGACTTCCTCCTTGTATTGTCTAGATTTGCAACACATAGTAACCCTTCTAN
NNNNNNNNNNNNNNNNNNNNNNNNNNNNNNNNNNNNNNNNNNNNNNNNNNNNNNNNNNNN
NNNNNNNNNNNNNNNNNNNNNNNNNNNNNNNNNNNNNNNNNNNNNNNNNNNNNNNNNNNN
NNNNNNNNNNNNNNNNNNNNNNNNNNNNNNNNNNNNNNNNNNNNNNNNNNNNNNNNNNNN
NNNNNNNNNNNNNNNNNNNNNNNNNNNNNNNNNNNNNNNNNNNNNNNNNNNNNNNNNNNN
NNNNNNNNNNNNNNNNNNNNNNNNNNNNNNNNNNNNNNNNNNNNNNNNNNNNNNNNNNNN
NNNNNNNNNTTTCTCTTTGTAGTGTTTTTGTCATTCTCATATGATACTTGTTTCTGTCATTCAC
GTTTTTTCGGTTGTCTTGCCAGACTCTCCTTTCTGTTATTCTCTTGTCTTTGCCTCTGTTTACTC
TGTTCAGTTTGTCTTCATTTTGATTTTTTTTGTCTATTCTTTTGGGTCTGTCTAGTCCACTTCTC
TTTAATTCGTTTCCTCTTCTCTTTACTTCATATCTTCTTTCTCTGAAATTTATGAATTCTTCTAT
TTGTATGTTANNNNNNNNNNNNNNNNNNNNNNNNNNNNNNNNNNNNNNNNNNNNNNNNNN
NNNNNNNNNNNNNNNNNNNNNNNNNNNNNNNNNNNNNNNNNNNNNNNNNNNNNNNCACTTGA
TTTATCAATGCAGAAGATAATAATGACCGATTATACCCCTTCAATTTTACTGTATTCTCTTCA

TTAGAAGTGCTTTTATTGCTAAAATCTATTGTTAGGTACACATATCCTGGTGATTCAAGGCTA
GGAATACAAGCATTTACTCTTTTGCCCTTTAGTTCTTTCAGGATTTCTCCGTTTAAGAACTTGT
TTAAACCATTGAGTCTAATTATCAACTTCTTTCTCAAGAGGCTGCAATCTCCTATTATGCTGC
TTAGACAGTCTTTCTTTTCAGTAATGTGGGAGATATCAAGTAAGATGATTGAGTGGTTGAAA
TAACTTATATAATCATTTTTAAGGAAACAGTTGAAATTAGTCTTTTTGTCAGTCATTCCTGGG
ATCCTCATTGCTAAATTGTATCCGTCACGCCTTGACAATGTAGTGTGGTTTATCCCTGCTTCC
AGCATAGCGAGATGGAAATCTCCTCTTCCACCACACAACTCTATCACTGAATCATTTTCTCCA
ATAAAATTATTTCCTTTCAATAACTCTAATAGTCCTCGTGCTGAAGGATACACTGCAGACAT
AGTAGGTGAAGATAATTTCTCTGGACTAACCATGAGTGAACAGGCTGTTTCGAAACCTTTTA
GTGCGGAATCTATTCCTACATTATCGAGCAGATCGAACCACTCGATAGACATTTCACTAAAT
TCAACTTTTCCGTAGTTCACCCCCTCTTTTTTTCCTGTTTCAGAAAGATGCATGTTCAGATAGT
AAGAACCCAACCTCATTTGTTCAAATTCGATACCTGAGATATCATCACAGAGTTGTATTGCA
CATACATGAACGTCAGAATAGCAATAACCAAGTGTAGGGAAAGAATGGACTATCATTTCTG
TTATTCTCTTGTCTTTAATATAGAACTGGCAATCCCCTCTTCTCATCCTTTCGAAGTTTGCTTT
GCTGTTCATGACTGTTCTTGTCTTGTGAACATGAAGTTTCATTATCCCATTATCTAACTCTATN
NNNNNNNNNNNNNNNNNNNNNNNNNNNNNNNNNNNNNNNNNNNNNNNNNNNNNNNNNN
NNNNNNNNNNNNNNNNNNNNNNNNNNNNNNNNNNNNNNNNNNNNNNNNNNNTAGAGTTT
TCATTCACTTTAAACAACTCCTCATATAATAGACCCCTGATTACAGACCGAGATGGAGTCTC
ATTACCAATCACATTCAAGGACTTATAATATCTATAAAATTTGTCAAAGAAACTAATTCTTCC
TTCTCTCGATANNNNNNNNNNNNNNNNNNNNNNNNNNNNNNNNNNNNNNNNNNNNNNNNN
NNNNNNNNNNNNNNNNNNNNNNNNNNNNNNNNNNNNNNNNNNNNNNNNNNNNNNNNNN
NNNNNNNNNNNNNNNNNNNNNNNNNNNNNNNNNNNNNNNNNNNNNNNNNNNNNNNNNN
NNNNNNNNNNNNNNNNNNNNNNNNNNNNNNNNNNNNNNNNNNNNNNNNNNNNNNNNNN
NNNNNNNNNNNNNNNNNNNNNNNNNNNNNNNNNNNNNNNNNNNNNNNNNNNNNNNNNN
NNNNNNNNNNNNNNNNNNNNNNNNNNNNNNNNNNNNNNNNNNNNNNNNNNNNNNNNNN
NNNNNNNNNNNNNNNNNNNNNNNNNNNNNNNNNNNNNNNNNNNNNAAGTATATCATCAA
GTATTGCAAGTTAAGATTGTTGTCTGCTCCACCTGTTCTAGAAATAAGTTGATCTAACCCAGT
TAGGTCGTAGTTTGTTGTCAATTTGTTAGATGATAGATCTCCCGTCTTGGGATTAAATCCTCC
TGTCAATGCTCTATGAAAATATCTACCTCCTGCAGGGCAGACAACATAACTTTCTATGTCTTC
ATATGTCGCATCTGTAAACGTCTTCAAGGTAACATTGCATATCTGGATTAAGCAATCAAGAT
CCTTCTTATCCATGGGCGTTGAAAACTTTTCTTGTTCCATCATGATCCATTTTGTGTATCTAAC
CAAGTCATAGGCTTTGAAAATTAACTTGTGTTCGAACATTCCATCTATCCCCAATTCCCTATC
AAATTTGGCAACATTCATGAAAGTCTTTTGTGGGGGACATGCCTTTCTACCTTTCTCAGTGAG
TTTTGTAGTGCAAGTGAAGATGGGTTGGAACATTGATCTATGTTCTGAATGCATTATTTTTCC
CATGAGAGGTATCTCCTCAATCCCAATGAAGTTTATTTTCACTGCTTGCTTGTCTTTGCTATC
GCTTGATACTCTAACATTCAAAGGCGTCATTCTTCTTTCATACATCAAGTCGTCGTAAGTAAT
AATTATCTTGTCATTCAACTTTATCTTCTTCTTGTAATTTATTGACCATGCATCATTGATGACT
CTTGCCATCGCTCTTCCTCCAATGAGCATTTTCATTGTAGAGGAGTTATCTATCTTTGATAAT
ANNNNNNNNNNNNNNNNNNNNNNNNNNNNNNNNNNNNNNNNNNNNNNNNNNNNNNNNN
NNNNNNNNNNNNNNNNNNNNNNNNNNNNNNNNNNNNNNNNNNNNNNNNNNNNNNNNNN

NNNNNNNNNNNNNNNNNNNNNNNNNNNNNNNNNNNNNNNNNNNNNNNNNNNNNNNNNNNN
NNNNNNNNTATTGGCCTAACCATTATTGATGTGTCTTTCCTTCCTCCACGAAGAGGAAATGA
GGTGTCGAGGTAACTGGTTGCATCATTTGACAAGTTTATCAACTTCCCCATGAGTGTATTAAT
TTGCGAAGATAATATTCCTTGCAAGTAGCATATCCTTTTCACATTTGACAGACTGTAGCCTGA
AATCAGAACATCTGGCATTGGAGTTGATGCATAACCATACATTGAGTAAGGAGCATATAAG
AAAAGTATTACTAAATCTTTCACCAGGTGGTTTTCTTTGTGAAATCTGTAGAAATCAATGTTT
CTAGATCTTGAAGTAGGTATGAAGACGTTAGAGTTGAGGTCTTTTTGCATTTCCCTCAATGCT
ATTTTAGCACTTATTTCATAATTATTTGATCCCACATTGTATCTATTGTCAAGTTCTTCAGGTA
TCATCTTTTCTACCATCCTTCTCAATGAACTAGAAATGGCTTTCAATGACCTAAAGTTCCTAA
GGATAGNNNNNNNNNNNNNNNNNNNNNNNNNNNNNNNNNNNNNNNNNNNGCCTGAATCA
ATTAGTCTCATTGACTCAGATTCATCACACATTACCCTTGGGCCATTTGGACTTATGGCCATC
ATCTTCTTTATACACATTTCTATCTGCTTTCCATCATAGTAATGTTCTTTTAACATAGAACATC
TCCCGTTTGTCACCTGAGTCTGTGATAGCTTGACAATCAAACCCATATCTTCTCCATATTTCT
GAACATTCCTTATTATGCCATTCAATTTTTCTACATCTAGTTTTGGCTGAGTGAAAACTCCAC
ATGAATCATCGGAATATGTGGTCCCAAGATAGTCTTTCACACCTAACTGCAAGAACATGTCC
TCGAGCATAAGCTGAGACTGAATTCCCCACAAGCTTCCTAGCCAACCTTCGATTGCCCCAAG
TTGTCCTTTAGAGAAGAGCACTGAATCACTCCAAGAATCTTCAACAATTNNNNNNNNNNNN
NNNNNNNNNNNNNNNNGTGATGTTATCTCTTCATACTCTTTGGAACCTTCATAATACCCGTA
CATGTTTGCGATCTCTTCAAGAATGAAATTAGTGTTCTCAGGTCGTTGTGATGTATTGTGTCC
TGAATAATCCAAGAAAAGCGAATAGGAATCTTTCATCAGGAGTTTCTCCGACATTTTGAACA
TAATGTCTCTTCTTTCATCATCTGTCATGGTCATCATCTGACCTGGAAGTAATTTCATTGCTCT
TTTAATCATTTCCATGACAGAACTGATCCACAACTTCAATTTGAAAGATGCTATGCCATAGA
ACCTGGCAGCCGATTTTTGTTCTCTTTCCTTCTCGGATAATCTGACGTTGAAAAACTCCCTTTT
GTCTTCATCCCCTTCTTTCAATATCTCTTTCCTGTTTTTCACAAATATTTTCCTTGCTTCCTTTT
TATACAATTTCTTATTTATATTCATAGGTTCTTTGAACACTTCTTTCATGTCTCTTGTGATATA
ATACTGCACTTCCTTCTCAGAATTGTTCTGGATATCATAATTGTCTTTGGTGCAAGCTTTATC
AATTATCCTCGATTGGAGGTTCATTTCTTGACCTGGCTCAAGAAATTTCCCTAAAATGACTTC
ATCATAGCGACTTATCTCAGAGATCATGCTTCCTAAATAATTTCCTCCTGATGCTTTCATTTC
CAATTGAGCACCAAGATCCTCAGGAACATTTAATAGGGAAGGGACTCTTCCGTGTTTCTTAA
TATATGAGACTATGACTTTTTGCCTGAACAATGACCTAAGCCTACGAATTGTGTTTGGGTTCA
CTTTGTTATCTCTGTTTGTTCTCTGTATATATTTGTTGATTCCTTTTCCTGGCTTAGTGGCTGCC
AAGATCAAGGTCTTTCCTATACATGACATTTTTATGCATGTTGGAGCATCTGACTTCATGCAG
ATCGAGACTAAATCTTCTGTCAATTTGCTAGATACTTTAATACTTTCGGCAAGATTGTCTAAT
ATAGGCATACTTGCTGAGAGTGTCTTCTCATCAGCCATTAATAAACATGTAGTCTCATATAG
ACTACCCATTCCAACTCTTGTTGTCTCATCCAATCCAAGTAAAGATACATAGTAATCCAAGA
AATCTCTTTCGTTTGTTGTGAGTGAAGATCTTATCAAGGAGAGCGTCAATAAATTGTCTATGA
GGTACATACTGTACTGCAACATATCAGGTCTTCCGAGATGAACAATATTGGTATTGTTCCAG
TAATAACCACATATAGTAGAAGAGACAAAAATTACCAACTTGCAAGGATCATCTTCAAATTC
CACAATTGAAATGCTTGCTCCTATAGGAAAGTATTGAGTGTTTATTCTCTTCCCTTCATAATA
TTCTATCATCTCTGGTATTTCTCTTTCCTTAGACCTAGCGTTTGATTGCATCATTATATTCTTG

TTTAGGTGTTCGAATATATCTAAGACCCAATAATTTTTGCACTATCCTCAGTGATTTCCCAT
TCTTCATTGTTTTTCTTCCACTTCCCAAGAGTGTCTTGCTAAGAGCGGAGATCTCATGATCC
ATTAACTTGCTAGCAAATTTTAGGAGTGCCTCTGACTCTTGTGATGGTTTGCTCTTTTCTCCCT
TCAATTTCAATATAAGAGAAATAGATGATTCTTGTGTAGAAACTAACCTCATGAATTCTCCA
ATATCATTTCGTGCACGGTTCAAAAATACTTCTCTAGTGAGAAGCAAGTCTATGTTATTCTTA
TCCATCCATTTTTCAGGATACGTCTTCTCTGTGATCATTTGAAGATTTTCCAAAGTTATCTCTT
TGTATGGGCTGTCAATTTTGCAGATTAGTTGAGGTACTCCTATTTTCCCAAGGTTGATTAGGT
ATGATCCATCAATCACTGTCTTATCTGTTATGACGTGAGTGAAATCAAACTCAACTCTTGGA
GATTCTTTTTTCTCCTCATCTTCAATTGTCTGGATCAGAGAGTCATATTCCTTGCTGAACTTGC
CTAATAGCCCTACTAGATTCGGTGTATCGTAATAGCACAAACCATAGGTTGTCATGAAACTT
TCCATATTTGCTTTCTTATACTCGTGGTTTTGCTCGCAGATTATTTCCTGTTGATCTTTAGAAC
TTGAATTTGTAATCTTGTGGTGTATCCGCATTTCACTTCTTCCTTTTTATTTAAAACTTTTACT
GTCCTAACATTTCGTTAATTCACCTTCGACTCCCTTCTCCCCTATTCTCTTTGATAATGGAGAA
GTTTCTTATATTTTCTTCTTGTATTTTTCGTTGAGGTTTAGTCAAAGTAGCTGAATATTTTGGG
AAAGCGATGTATTCACAAACTTCATACAGTTGTTCTCCATGATATATCACCATCTCTTCAATT
CCAAACCTAAAATAGTATGATTCAACAACGTCTTTCAATGAATTTGCAAGCACAATTTCAAT
GTCACCCACTGAATCAACATCGACTATCTTATTGTTACTCTGTATAAGAATGACCGATTTTTT
ATGGAAAGGATGTTTTAACTCCTTTTCATTCAACAGCCAAGAACCAACCAGAAATCTATAAG
TAGAACGATAACTAGTTTTATATTCACACAATTTCATAAATGATCTTTTATTCCTCTTATTTAT
CTTCTCATTGTCAATTCTAATCTCTCTCAAATTCATTGTTTCCCTGATGTCTCTGTGAATAATC
TTAGAAGGATCACTATTTGACAATGATCTGGGCTCCATGTTAACTCAAAGATTGTTTTTGATC
TAGATGGTTTCTTAGCTTTGAAGTTTGTTGTAGTCAGGTAGCTATTTAATTGTGTTA
>Seq5 [organism=Agrostis ophiovirus_agro] RNA2 genomic sequence
CAATATATTCAGGTGTTAGTTAAAACCGTTTCCTGACTAACTGATGTTCCTTGGGGCAGTCAT
CCTGTATCCATAGGGAGTTTCAGAACATACTAGCCTTTCCAGGAGGTCTCAGCAGGCTGCGA
TAGCCGGACCGGAAACACCATTACTGATGCTTCGAACAACCTTTTTATTTAAAACATCAAAA
TGCAAAAGCACTCATAGTAATCACACACAAGACAATCTCGATGTCTTACTCAACCAGGATAG
GTCACTATGCAACCACTTAATACCATCTACTTCTATTCTGCAATCGGCTAGCCACTTTCAACA
AGCATCTGAAGACTAAGCTCATCAGACGTCTTAAACACCATATAATGACCACTACAGTCCAA
CTCGAATATCACCTTCCCATCTTTGCACAACACTGCAGTTTTCTCCTTATCATATGACTGCAT
GCTGTCTATGATTCCTCCTTCCTGTAGTTTCTTGTAAGATATCAAAGGGAAGTCTGGCTTGTC
ACCAGGTAAAGAATAACAAATCTCTTTGAATTTCACCCAGTGCTGCCCTAGCTTTATCTCAA
CCCCTTTCACATTGTAGTATGTGATTCCCCTTTCATGTTGTGCACCTCTTGGTTTCTCAGATAA
ATTAATCAGTCCTGGGTAATAGAACCAGTGGTCAGGTGCTCCTGTGTCCAATGCGATCTTAT
CGGGATGGATAAAGGGCAACCTCCTTGGTAAGTAATCAGATATATTTTTCTCTGATATCTTAT
GTATTTCACTCATTTCTGGCATTCTTTCGATGCATATTCCTGAGTCTGTCCTTGATGAACTTCT
GATCTTTTCATCTCCCATCGTCAGAGATCTCATGCTCCCAGTGAATCTCATGCTTGACTTCCT
TTCTTCTTCTTCTTGATCGTATTTATCTTCTGCTTTCTCAAATCTCTCCTTATTGTTTTCAATCC
TCCTATTAACTGTGTTGACTATTCCTTTCAGTGAGGATGTCTTGTTTATATCCTTTGGTAACTC
AATGTCGTCTATCAACAGAGAAATTGGCTTCTTAAACTCATAAGTTATTGGTAAATATTCAC

CCTGAATGTAAAAACAACAAACCATGAATCCAAAAGCACGCCCTTTTACAGGAACTCTCTTC
ACCTTGTAATTGAACTCTATCATGCTTAGGTGTTCACTCGGGATGAAGTGGTCAGCGTATAG
TTCAATCATACTCATCTTGTTCAGATAAGTTGATATGCTAGAAATAGACTTTGACTTGTTTCC
AACCTTCACGGAAGTATCGTTGATTGAGAACGTGATCTTCTCTTCCTTGGCTTCATCCCTAGA
AAACAGAGGGAGATAGAGTATCTTTAGTCTTACAATCCTATAAAATGGGTAACTATGACCCG
CTATTATTGTCTTCAGATTATCCATAGTCCCCAACTTCACTTTCTTCGAACCCTCCTTAGACTC
TGCAGTCATCTTCATGGGAGAAACTACTGAGCTAATCTTTCCTTTTATTGCATCTTTGAACTC
TGCTGCCATGCATTCCACTTCTGGTGATATCGCTCCATCAGTGTTCAACGAAGCCAAAATTGT
CACTCCTTCTTCCACCATCTGATCTGTTGCTTGGGAAAATTTCACTGCCTTCATGTCAACTAG
CTCCTTGTTCGATTTTTCTTCCTTGCCATTGAACAGTGATGAATTCATCTTGTTCATCGTACTT
GTTGATCCCATTGCCAAAGGTAGTCTTGCGTGGACTTGGATTTTTATCAGGTTATTCTCTTTC
ACCGTAAGATTATTCACCCGTGATATGTTTTGCATGTAGATACGTTGGCTAGCGTGCTTGTTA
AAGATTATGAGGGTTTATAGTTTCGAACGTTGCTCTCTT
>Seq6 [organism=Agrostis ophiovirus_agro] RNA3 genomic sequence
TATTTAAAGCTATCATTGTTCAGACTCCAAAGTCGCGCCTTCAACTCGTCTTAACCGTGCTCC
TTCTCTCTCAACAAGTCCATCAGCTCGTGAAGTCGTAATATTTTCACTTCATCCAGACTTCCC
CCATGCAGTTTTAATAGCATCGACAGAAAGTTCAGAGAAGTCTGCGTCAGAATCAAAGAGT
ACTCTGTGTTCTGGTTCATTGATTTGGTTCACAGTACCATAGAAGTTTGCATCCTTTTTGAAC
GCTTCTATCTTTCCATCGTTTATTGCTTTCCTCAAGTCCAGTCTCCCCTTAGGCGAGAGAGAG
AATATGATTGCACAGGTCAACTGTAAAGTCAAATTCTTTCTCACTGTCCTTTTCGGGTTAGCA
GGATTCATGTGTTTCTGGGCTTCGAAATCAGATGCAAGTGCTGAAAGAGTGTCTCTTATCTTC
TGAGCCTTGGTCAATTTTTCGCTTTCTGTTGTATACTTAACAAGGGCATCAGTATCAGTGCTC
GTTGGTTTGGTGATAGTATTCTTCTCAAACTTCATAGAAAGAGTCGCATATCTGATTGCTCTG
TTCCCTGCAACTGACATCTTGCACCTTGATGCGACTTCAGTGGGCAAGAATTTCAGGTCTATT
TCCAAAAAAACACCTGCTGGAAACAGTCCGGGATCAGCAGTGGAGAGCATCTCTACGAGCT
TGTTTGCAGTCAGTTCCTTTGTGCCTTTAAACAAGGTGTCCTTGATGAATTTAGATAGATTCT
TTTCATCTGAGGTCCTGGAAGGTAACGAACCCTGATTATAAACCATGCAGAAAGATGCAAGT
AGTGCTTTCTTTCCTTCATCCATCCTTTTTGCTATTTCACTTGTGTTCACCGTCTTAGGGGACA
GTTCCTTAGTTACGAAGATAAAGTTGTGATCTTTAGAAGATTTATAAGCCATATACATTATG
GCGTCCAGTATTTTAGTTCCAGCAGACAAGAGGCTGCTGATGCTGTCTTCAACACCTGAATA
CCAGAAAATTGATATGTCACCCGTTTTGTATGTCTCTGCTGTTTTTGGCAAATCTTTTACATA
GGTTTCAAGCACTTTCTGTATTTTACTAGAATCAATTGTTAGGCTCTTTACTTGGATCATCTTT
TTCAATGCTTCTGCAGAGAATTTCACATCAAGTCCAGCTTCTTCAATCTTATCATCTGTTTTA
ACTGTGCTGCTTGCTTCTCCTTCCTTCGCTTTGCCTTTTCCTTCTGATCCTGTGATAACAGCAG
TCGGTGCTGTGAATTCCAGGGTACCTTCAGGCTTCAAGTACACTAAGTAACTTGGAGTCGTT
TTCTCAACTGCTTTCAGTGCATCAGTCATCTTTGTGTTCACAGTTTCATCAGTGTTCAAAACC
TTGATTGATTTCATGAACGTCTTCTCATCAACACTTAATACAGGTTTCCTGGCCAGATCGAGC
ACTTCCTTGACAGTTTTGACTCCTGACATTTCGATTTCTCTCTAGTAGCTTGATTAAAAGTGA
AAGTTTGAGGTTGCTTATTGCTCGACAGTTGCT
>Seq7 [organism=Agrostis ophiovirus_agro] RNA4 genomic sequence

ATTTAAAACTTGACAAGATCAATACCTCACATTGACTATTATTCTCTAATCTACCCATTAATC
GGTATCATACTGATATGACTCGAAATTATCATGGAAGTTATCTTCTCGATCAACCTCATAAAT
CTCCCAATACTCACGAAGATAGCTTCCATACCAGCTTTCAAGCATTTCGAAATCTTCTCGAGC
ATCTGTGTGCATTTCAAATATCTCAGATAAAAGATTGCTCCTGATCTTCCTTGGTATCTTCAC
TGGACGATTGTGGAAAGTCTCGAATCTTACATCGTGATATATCACGATAAGGTAGTTCAAGT
CTATCTCAGTGTCTCTCTTAAAAAGTTCTATCACTCTGTAGAGATTCATTTGATAACCTTAAA
CTTTTGAATTGTGTGTTATGTTCATCTAACCTTTTATTTAAAACATTGAAATTGTAATCCATGT
GAATTGATCAACCTGATTCCTGCTTCTCTCTCTTTGTCTCATCACCACTTCTCACCTAACCAAC
TGGGAAGCATATCAACTTGTTGAGGTAAAATGAATCAAACCAGTTCTCTAACATAGCTAGGT
CACGTTTGGCATCAGAATGTTTCAACAGGATTTCTGACAATGACCAGTCTCTCAACACACTC
TCCATGAGAAAGTACTTTCCACACGTTTCATACCTAACATCACGATATATCAATATGAGATA
GCTGAGATCAATATTCATATGTTCCCTGAATAAGCGTATTACAGTGTAGAAGGTCATTCCTA
TAGGATACACTTTGCTATCTCGATAGATGTATCCCATTACGTGGTTAGGCATCATCTTATTAT
TCATTCAGTACTGTGATTGTTCACTTGTCTTTTCATAAGTTAAAACAAAACTATCACCACCAA
GGGATATCTCTCGAAAGCAATCTTGTAACATCCTTCTCAATCTTCCTAATTCTTGCTGCCTCA
GCTTCAATAGGGCGCCTTGGATACATGTAATCAAATAATTGTTTGTCAAATAGACTTGTAGT
AGTCCATAATCTCATCATTATAGATGATTCTATTATCAACTTATCCATTATATCTTCCAATAA
AGAAGGATCACCCCTATATCTAGAGATTACAGAGTACCTTCCACTTGGAAAACCTAGCTCGA
AGACACAATCCACTCTTTGTTTCATAAACTGTGTGAGGAAAACATCCTTTCTAAGGCCTATA
GAATTCCAAGTAAAATTAATTACTCCGAATAAGTCTAAGCAGGAGCTATTATTTAGGTTCTC
AAGTGAATACTTTCCTCCATAGCCTTTTCTTCCATGTGCATGATATCTGTCAATGACGTATAA
CCCTGGACCTGTCCTTCTAGGATGCATCTTCATCATGTAATCCAGCCCTTCTTCTAGTGTTCTT
ATCAAACTTTTCTTGATTGGGTTAATTGAGAATTCTTTCTCGTGCAAGATTAAGCTGTGCTCC
ATTTCCTTCTGATAGAATATTACAAAATTGTTTTTCTCCAAAGCACCAATAGATTCTAAGCTT
TCAGTCATTTCTTCGAACAAAGGAACCAGCATATCTGTCAACATGTGATTCTGCAACCGATT
CAGCTGGTCCTTGTAACGCATAAGAATCCTTGTCCTATCCTCTGACCTCAACATGATAGTGTT
TGTCTTGTCACTTGACATGTATTTGAATTTCTCATCAATGAACGAAATTCTGTTTCTTAGTGA
TATCTCATCACTGTTTTCCAACTGATCAAGACCATTGAAAGGCTTGAAGGCGATGTTCAAGA
TTTCTCTAATATGTGGAACAATTGCAACTATTTTCGAGAAAATAGGCTCAATCAAGGAATGC
CACTTCAACGAAAAACTCATCTTCTGAAAGTCTGTTAGTGTCAATTTTATACTGTTCAGCTCT
TTGATTCTTTTATCTTCTGACCTTAA
>Seq8 [organism=Agrostis ophiovirus_poa] RNA1 partial sequence
ACACAGGCCGTTGTCTATCATAAATTGTTCCATATTTTTTCCTCTTTGTTTTTTTCTGGTTTGA
TCTAGTCCTTTGTGTTTGTATAGAAGTTGAGCTTTGATCCGGAATTTTTCTTGTGTCTCACGGC
CCCTTTTGTGTTTTATTTAAAACATCACCCATATCACATGGATCCTGACTTATCTCTTACTTCC
TCCTCCTCTACATCCTTTAATAATGGAGAAGTTTCTCACATTTTCTTCCTGTATCTTCCTCTGA
GGTTTGGTCAAAGTTGCAGAATACTTTGGAAGGGCAATACAGTCGCAGACTTCATACAATTG
TTCATTGTGATAAATTACCATATCCTCGATTCCAAACCTGAAATAATATGATTCCACCACATC
TTTTAGAGAATTGGCAAGCACAATCTCAATGTCACTTATCGAATCTACATCAACAATCTTACT
ATTGCTCTGAATAAGAATAACTGATTTCTTGTGAAAAGGATGTTTAAACTCTTTCTCATTTAA

CAGCCAAGAACCGACTAAGAATCTGTGAGAGGAACAATAACTGGTTTTGTAATCACACAAT
TTCTTAAAGGATCTCTTATTTCTTTCATTTATTCCATCATCATCAAGTCTTATTTCCTTTAAGT
CCATAATCTCAGTTATGTCCCTTTCAGCAATTTTTACTGGACAACAGATCAGTGGATTGCTGG
CTTCCATTTTAGTATATGATTTGCTCAAGATTTTGATAGTTTCTTAGTTTGATGGCCTGTATTG
GTCTGTGAACCCTTAATTGTGTTAAAAAACTATCAAAATCTTGATTTGCTCAAGAGATCGGA
AGA
>Seq9 [organism=Agrostis ophiovirus_poa] RNA2 genomic sequence
TATATTCTTCCTAACTGGTTTACCTTGGAGCTGTCATTCTGTGTCCATAGGGAGTCTCAGAAC
ATACTAGCCTTTCCAGGTGGGTTCAGCAGGCTGCAATAGCCGACCCGGAACCACCATTACTG
ATGATTCAACCAACCGTTTTATTTAAAACATAACACTGCAAGAGAGTAAATTACAATCACAC
AAACACGCAATCCTGACATTTTATTCAATGAGAATACGTAACAATATACTCATTTGATTTCGT
CTTTTCTCCCCCTTTAGTACTCTATTAATTGTTTTCCACAAGCATTTGAAGACTGAGCTCATCA
GAATTCTTGAATACCATGTAGTGTCCACTACAATCCAATTCAAATATGACCTTTCCATCTTTG
CATAATACTGCAGTCTTTTCCTTGTCGTAGGATTGCATGCTATCTATGATACCTCCCTCTTGC
AGTTTCTTGTAAGAGATCAGTGGGAAGTCAGGTTTGTCAGCTGGCAAAGAATAGCAGATCTC
TTTGAATTTCACCCAGTGCTGTCCCAACTTCACCTCGACTCCTTTTACATTATAATATGTTGTC
CTTTCTTCATGTTGTGCTCCTCTTGGTTTTTCCACTAAATTTATCAGTCCTGGATAATAAAACC
AGTGATTAGGTGCTCCAGTGTCTAATGCTATCTTGTCTGCATGGATAAATGGTAATCTCCTAG
GCAAGTAGTCAGATATATTCTTTTCTGATATCTTGTGCACTTCACTCATCTCTGGTACCCTCTC
AATACATATTCCTGAATCTGTTCTCATAGGATTCCTAATCTTTTCTCCGCCCATTGTTAGAGT
CTTTAAATTTCCCGTAAATCTCATGCTTGCTTTCCTCTCCTCTTCTTCTTGTTCGTATTTGTCTT
CTGCTTTCTCAAACCTTTCCTTATTATCTTCTATTCTTTTATTGACTGTTTTCACTATCCCTTTC
AGTGCTGATGTCTTGTTGATATCCTTTGGCAACTCGATGTCGTCGATTAATAGAGAAATTGGC
TTCTTGAATTCATAAGTTATTGGCAAGTATTCTCCCTGGATGTAGAAGCAGCAAACCATGAA
ACCGAAAGCACGCCCTTTCACAGGAACTTTCTTCACTTTGTAATTGAACTCTATCATGCCTAA
ATGTTCACTCGGTATAAAGTGGTCAGCGTAGAGTTCGATCATGCTCATCTTGTTTAAATATGT
AGAGGTGCTAGATATAGCCTTTGACTTGGTTCCAACTTTCACAGAAGTATCATTTATCGAGA
ACGTAATCTTCTCGTCTTTTGCTTCATCTCTAGAGAATAGAGGAAGATACAATATCTTCAGTC
GAACAATTCTATAGAAAGGATAGCTGTGTCCAGCTATTATTGTTTTCAAATTGTCCATTGTTC
CCAGCTTAACTCTCCTTGACCCCTCCTTGGCGTCCGCAGTCATCTTCATCGGAGAAACTACGG
AACTTATCTTTCCTTTTATTGCATCCTTGAATTCTGCTGCCATGCATTCCACTTCAGGTGATAT
TTCGCCACTAGTGTTAAGAGAGGCCAAGATCGTAACACCCTCCTCCACCATCTGATCTGTTG
CTTGAGAGAATCTCACTGAACTCATATTGATTAAGTCTCTGTTCACCTTCTCTTCCTTTCCGTT
GAACACAGATGAGGTAACCTTGTTCATTGTGCTCGTTGATTCCATTATTAAAGGGAGTCTTG
CGTGAACTTGGATTTTGATCAAATTATTCTCTCTGACCTTAAGCTTGTTCACCCGTGAGATGT
TTTGCATGCAGTCACTTCGATTGGAATGATCGTTTAAGGTTTAGATTGCTTATAGCTCGAT
>Seq10 [organism=Agrostis ophiovirus_poa] RNA3 genomic sequence
GCTATCATTGGTCAGAAGCAATATGTGCACTCACACTCCATCTTAACCGCACTCCTTCTCTCC
AGACAAGTTATTCCTCTCTTGAGTTCGTAGGAAGTTCACATCATCCTTGTTTGCCCCACGCGG
TTTTGATAGCATCAACAGAAAGTTCAGCGAAGTCAGCCTCAGCATCAAAGAGCACCCGGTG

CTCTGGTTCATTGATCTGGTTTACAGTACCATAGAAGTTGGCATCCTTCTTGAATGCTTCTAT
TTTCCCATCATTAATTGCTTTCCTCAAGTCCAACCTGCCCTTTGGGGAAAGAGAGAAGATGA
TTGCACATGTCAATTGCAAAGTTAGATTCTTCCTAACTGTCCTTCTTGGATTAGCAGGGTTCA
TGTGCTTCTGAGCTTCAAAGTCAGATGCAAGTGCAGAAAGAGTGTCTGTTATCTTCTTAGCCT
TGGCCAATTTCTCTACTTCTGCAGAATATTTCACTATGACATCAGTATCAGTGCTAGTAGGCT
TACTGATTGTATTCTTTTCAAACTTCATTGAAAGAGTAGCATATCGAATTGCTCTATTCCCTG
CTACAGACATTTTGCACCTTGACGCGACCTCAGTCGGTAGAAACTTCAGATCGATTTCCAAG
AACACACCTGCTGGGAATAGTCCAGGATCAGCAGTGGACAACATTCCGACCAACTTGTTAGC
AGTCAATTCTTTAGTGCCTTTGAACAATGTTTCCTTGATAAATTTGGAGAGATTCTTTTCATC
AGAAGTCTTCGAAGGGAGAGAGCCTTGATTGTAAACCATGCAAAATGATGCAAGTAGTGCT
TTCTTCCCTTCATCCATTCTCTTAGCTATCTCTCCAGGGTTCACTGTTTTCGGTGACAGTTCTT
TAGTGACAAAAATGAAGTTGTGATCTTTAGAAGACTTATATGCCATGTACATGATAGCATCT
AGTATTTTAGTGCCAGCAGACAAAAGGCTACTGATGCTATCCTCAACCCCTGAGTACCAGAA
CACTGAGATGTCACCAGTCTTGTATGTTTCGGCTGTTTTGGTAGGTCTTTGACATACGCTTC
AAGAACTTCTTTAATTTTGCTAGAGTCAATTGTTAGACTTTTTACTTGGATCATCTTTCTCATT
CCCTCAGCTGAGAATGCTACTTCGGTTCCTGCCACTTCAGCTTTGTCATCCGTTTTTTCTGTGC
TGCTCGTTCCCATGTCTTTTCCCTTACTTTTGTCCACAGATCCGGTAGAAACAGAAACCGGTG
CTGTGAATTCCAGGGTACCTTCAGGTTTCAAGTACACCAGATAACTCGGTGTTGTTTTCTCGA
CAGCCTTCAGTGCATCAGTCATTTTCGTGTTCACAGTTTCATCATCGTTCAGGACCTTGATCG
ATTTCATGAAAGTTTTCTCATCAACACTTAACACGGATTTCCTGGCCAGATCGAGCACCTCCT
TGACAGTTTTAACTCCTGACATTTCTCCTTCTCTCTAGTAGCTTGTTAAAAGGTGAAGATTTG
AGATTGCTTATTGCTCGAC
>Seq11 [organism= Agrostis ophiovirus_poa] RNA4 genomic sequence
CTCAGTCGTAGTCAGTCCTTTATTACTTGTTTCTTAACCGATTCAGAATGCAGTTAACTTGTC
CAAGTAAAATAATTCGAACCAGTTTTCCAACAGTGCCAGATCATACTCAGCTCTAGAATGTG
TGAACAAGATCACCGATAAAGGTAAATCCCTCATAACTCTCGAAATGAGGAAATACTTCTCG
ACCGTTTCGTATCTAACATCATGATATATCAGAATGAGATAACTCAGATCAATACCTTCGTG
TTCTTTGAATAAACGTATCACAGTATAGAAGTTCATGCCTACAGGATAAACTCGATTATTCTT
ATAGAGATGTCCCATGATGTAATCAGGCATTTTCTTGTTATTCCTTCAACTCTGTGATTGTTC
ACTCATCCTTTAGTTATTTAAAACCAGACTACGGCCACCAAGGTATGTCTCTCGAAAGTGAC
CTTGTGATTTCCTTTTCAATTCTCCTGATTCTAGCTACTTCAGCCTCTATAGGGCGCCTAGGGT
ACATGTATTCGAACAACTGTTTATCAAAAAGATTAGTAGTATTCCAGAGCCTCATCATTATG
GATGATTCTATTATCAATTTGTCCATTATATCTTCTAGCAGGGAAGGATCATTTCTTTCTCTTA
AGATCACAGAGCACCTCCCACGTGGGAAACCTAACTCAAAGACGCAGTTCACTCTCTGCTTC
ATAAATTGGGTGAGGAAAACATCTTTCCTAAGATTCACAGAATTCCAGGTGAAGTTGATAAC
TCCGAATAAATCTAAACATGAGCTATTATTCAAGTTTTCAAGTGAATATTTTCCTTCTTGATT
TGCTCTTCCAAATGCTTGATATCTATCAATGACATATAGACCTGGGCTCGTCCTTCGTGGGTG
CATTTTCATCAGATAATCAAACCCTTCTTCAAGTGTCCTTATCAAACTTTTCTTGATCGGATC
AATCGAGAATTCTTTCTCATGCAAGATCAGGCTGTGCTCCATCTCCTTTAGATAGAATCTCAC
AAAGCTGTTTTTTTCTAAATTACCGATAGCTTCTAAGCTTTCAGTCATTTCTTCAAACAAGGG

TATCAACATATCAGCTAGCATATGCTTTTGCAATAGGTTCAGCTGATCCTTATAACGCATCAG
TATCCTTGTCCTGTCTTCAGATCTCAGCATGATAGCATTTGTTGTGTCATTTGACATGTACTTA
AATTTCTCGTCAATAAACAAGATTTTATTCCTCAATGACATTTCATCGCTAATTTCTAACTGG
TCGAGACCATTGAAAGGTTTAAATGCAATGTTCAGTATTTCTCGAATATGGGGAACGATCGC
TACTATTCTCGAGAAAATAGGCTCAACCAGGGAATGCCACTTCAGTGAAAAGCTCATTTTCC
AAATTTCTGCTAATCTCAGCTTTCTAAGGTTATGATTTTTGAGATCGG
>Seq12 [organism=Allium ophiovirus] RNA1 genomic sequence
CATAATTTGCGATCCTCACATTGAGAAGCGACTTTTTGAGTACACAATGTACGCCAACTGGA
AAATTAAATGTAAAAGAATGATTTCTATTGCGAATCTTAATTAAACATAAATTTAGATTACA
TAATTTCCTACTAATAGTCTTCATATTCTTCATATTCTTGGTCAGAAAGATTTTCCAATGCATC
TTCTAACTCTTCATACGTCATTGTTGTGTTCTTCTCATAAGTTTCGATTGAAGTATCTGTTATA
AAGTTATTCATTAATTCTGAAACCCCAGATTCCATATCACCAATCAATTCACAACTAGGATTC
ATGGACATCTCATTTTGCAGGCTTTCTACAAAGGCATCAAAAAGATCATTTGATTTTTGATTC
TTAAAATAATCTTCTACTGTGTCAAAGGATGATATGAATTCAATCAGTTCTTTCTCTTCTTTA
TCATTGATTCTTCTTTTGTGATGAGATCTTAACAAATCTTTAATCTTCATATAATTATAAGAA
ATATTTAATGCCAAATTTCTATGTATTCTTGTTATACGATTAATTATTAGTTTGTAATTAAGG
CAGTCATATCGCTTTGCTTTTGATTTATTTTTCATTAATTCATTCATATTTGGTTTTTTCATGCC
TGAAATGATCATTAATCCTTCGATTATCTTTCCAGATTTATATGCTTTTATAGCAGTATGAGT
TGTGTGATAGAGCTTATCAATTGTTCTTGATTGATCAGGTTTGAGCTTAGACAAATTCAAATT
TTCTAATATTTCTGAAGAATATATCTTGTCTATTATACCTTCTGATAATTTGTGCATAAATTTC
CACATTATGAGTGAATTTGGAGTTACGCTCTTATGTGATATTACTAATTTTGTTAACTCAAAT
ATTTCGCTCTCTGTTAAATTTTCATAAGATATTATGAACCCTTTAACACCTATGTTTCCTATAT
AATTGTTTACTATCAATGTGTCAGGAAAGTTTTTTGATTGTGCTTCAAGGTAAGCATGTTTAT
AATTTGTAATTTTTGAGTCAGCTGCTATTAATAACCTCGGATTATATATCTTCAATTTTTCTTT
CTCTATAAGTTCTTTTATGAAAGCATTATTTTTCTTTGAATCATCATCAAAAAGATTTCTGG
GTATGGAACGGGTGTAAAAATCATGCTTTCTATATCTACCTTAGATTTTATTTCATCTTCTAT
AAAACAAGGCATTTCTATGTAATCAGGGTCATTTGATATCAACATCTCTGTCAATTTGCTGTC
CGAGATTAACTCTTCTGTATGATCTTTTGTATTACTCTTATCGTTATCAAATAGATTCACTTTA
GACATAGAATTTATAGAATTGACCAATTTTTCAAGGACAATTGAATCTGAAATTTTATTTTTC
TTACTTCCTTTCAAATCAATTTTCTTTCCTGTTGGATCATGAATTAAATAAATATATCCAGGT
GATTCTACTGAAGGTATCATTATTTCTGTTTTGCCTAATGACAATTTTTCTATTATCTCTTCTG
TCATATATTTCATAATTGAATTGATCCTTATTATTATCCTTTTATTGAAAGAGCAGAGTTCAT
TGATCATTTCTGAAAGCTTCTTTGCTTTGCTATTAAAATGTGAAATATCGATCAAAAAGATAT
CATAATTTACATATTGTATATAATTCTCTCTATCTGTAATGTCAATAGATGATTTAATTTCTGT
CATCCCTTTAATTCTATTAATTAAATTATATCCGTCATTCCTAGATACACTGTTGTGTTTTATA
TTCAATAAATTCATGCATAAATGTGAGTCACCCCTTCCTGCAAATAAATCACATATCCTATCA
GTTTTCTTGATGAAATTTGACTCCATCAATCTTTTCATTGCAGAAAATATTGAAGGGAAAAC
GTCAGACATTGTAGGAGAAGATACATTTTTTGGCTTACAATTCATAGCACATATCATCTCAA
ATCCTTTAATTGCAGCCATGAATCTCTTTTTGTCCAGCAACTCAATAGGATTTATCTCTATCA
TTTCATAATCAATATCATTTGGCGTATTTTTTGTAAAAGGATCATCTTCACTCACATAATGTT

TGACTTCTTCATTATAATATGAGTTCAATTGATATTGCTGGAATGATTTATATTGATATTCTTT
TAAAATTTCTGATACTCCTTCTCTTATCTCATACATTAAGTACCCTGAAGTCGGAAATGCCAT
CAACAATAATTCAGATATCCTTTTATCTTTAATATGAAAATGCGCTTTGTTATTCTTAATTCTC
CTTAAATTGTTAACCCCGTTCATGATTGTCATTGTCTCATTTATATTTATTCTATATTCACCTA
TTCTCAATTCTTCAAGACGATAAACTAAACTTGTAGAGATTAAGAATAATTTCAACAACACA
TTTCTATAATCCTTTGAATAACCTTTTTCACAAATTTCTCTTGTCCAGTTTCCACCTGATTCAT
TTGGTCTGAATAATTCATTGTAAAGTAGACTTCTGATCACACTTCTTGTTGGTGTCTCATTAT
CAATTATATTGAGTCCTTTATAAAATCTGTAAAAATCATCAAAATAAGTATAACGATTTGAG
TAATCCTCTAATTCAGGTGCTAATTTTATTAATGTTTCATCTGGTATTGTTTCTGGAGATAAA
ATTCCTTGATCTTCCATGTATTTATGCACGGATCTGAATGAATTCTGTCTCATTATTATTTCAT
TTGGTATTGTAACTGAATGATTTATGAATTTCCCTGTTATATTATCATCGGTGGAAATAAAAT
AAGAAAAGCTTTTATACAGTTTCGCCTTTGACCGTATTTCTTTCAATTCGGTTAATATATTGT
CATTGCTATAGAATGTTGTGATCATGTTATCAGGTTTCATATTACTCAAGACAAAAGATACA
TCTCTACAATGTTTTAAGACATCATTTTTCAGTCTTAATGGTTGCAACTCATAGGAACTTGGA
TTTATGTAACTGAGAGATGCTTTTAATGACATCATCAAGTATTGCAAGTTGATATTATTATCA
ACTCCACCGGTTGATTCTATTAATCTTTGGACTCCTGTAATTTCATATTTTTGTGTTTCTTCAT
TTGATGAAAGATCTCCTGTTTTAGGGTTAAAACCTGCTGAGACAGCACGGTGAAAATATCTT
CCTCCTTTTGGCGCTACTACATGTTCTGATAAATCTTTAAATGTTACATTTGAGAATGTGCTC
AGAGTACTGTCAGATATCTTGTACAATGAATTTTTTGTTTCCTCTGAGCATTGACTATATTTC
TCCATATCCATTATTATCCATTTCACGTATCTTACTAGTTCATAACTTAGGAAAATCAATTTA
TTTTGAAACATGCCCTCTATCTCTAAATCTCTGTCGAATTTTGTCTTATTAACATTAGTTTTTG
TTGGAGGTCTTAATTTTCGAGTTTCATCTTTCATTCCTTGCTTTACAGGCACTATCTGGCCATT
TATTTCATCGGACATTATTTTTCCAAGTAATGGAACTTCTTCTATTTTCAAAAATTTTATCCTT
ATGTCATGTTTTTTATCTCTTAGCTTGAAATGTTTTTTTACATTATCTCTTGCTTCAATTATTCC
TTCATAATCCAGTTCTATCTCATTGAAATTATTTGTATTCGAAATTCAATTTTATGATTTCTT
TCCCATGCTTTGTTCCATAATTTACTCACTTTTAATTTTCCTATGATGTAGCTGAAGGTTGTAG
AATTATCAATCTTTGAGTATATTTCATCAAAATGGCTAAATATTGAACATTCATAGAATTTGC
TGACTATCCTTGCAGAAAAACACTCTTTAAATGTTGTTATCAATTCAGCTAAATATTGTTCTT
TGTCTAGTGCCCGCACTTTATTGTGTAAATTTAATAAGTCACTATTCTTGATTCTAGATGGAA
GAATTTCTTTTAATACATCTTTCAATAATAATGAGGTATCATGCCTTCCTCCTATCATAGGAA
ATGGGTTATTAATATAATTTGTCCCATCTTCTGAAAATTCTATGAATGAGTATAAATGTCCTG
TAAGAAAGCCTCCTTCTAATATAGACTCTATATATGATATTCTTTTGATATTAGATATCGAAT
ATCCTGATAATATAGCATCAGGTATAGGAGTGCATGCAAAACCATACATTGAATAAGGCAT
ATAAAATAGCATGAGTGTGCTTTTTAACAAAAATATATTTTCTTGATTGAACAAGAAAAAAT
CATTTATTATATTCTCTTCTGATGGTAATGAGATTATGTCATTGAATTTTCCCTTCCTGTAATA
AATTTTTGCTAAATAATTTTCAGCAAATGAATTCAGGTAATATCGATGATCTAGGTCTAACTG
CAGATAATAAGAGAATCTCATTATTTCTTTTCTTATGATCATTATGACTCTATAGTTCCTAAT
GATTGTTTGCAATTTATGATTGTTTGATCTTGAAACTGAAGAAGTGTATCCACTATCAATGTT
TTTAATCATTTCCAATTCATCTCCCCATAATACTGATGTGTTTGGTGATATTCCCATAATTCTT
TTGTAATTATTTTCTATGGCTCTTCCTTTAACATAATGGTTCTTCAACATTGAACATCTTCCAT

TCGTGACTTGGGTTTGAGACAATTTGACCAATAACCCCATTTTAAGTGCATAAGACTGAACT
CTAGATATAATATCATTAAGTTTTCTTTCATTCAATTCTTTTTCGATGAATACTCCACAAGAA
TCATCACTGTAAGTAGTGGCAATATAATTCTTAAACCCTAATGATTTGAACATGTCCTCCATC
ATTAATTGTGATTGTATTCCCCAAAGAGGGCCTAACCAACCTTCTATAGCACCTCGTTGATAC
TTGCTCAAATAGACTGTGTCTGAATAGGGATTTTCATAAATGACGTCTATGTTATTGAATACA
TAAAGTACTTGCAAAAAATGTTTCCTGGCATCTGAATTGTCATCAAACCCATACATGTCTGC
AATCTCTTCCATGATGAATAAGTTGTTGTCCGGTCTTTGAGATGTATTGTGTCCAGAATAATC
CAAGAAAATTGAGTAGCTGTTCTTATCATTCAACAACACAGACATTTTGTGCATTATCGTCCT
TCTTTCATCCTCTGTCATAGTCATCATTTGTCCAGGAATCAATTTCATTGCTCTTTTAACCAGT
TCCATTATTGTAGATATGTATATCTTAAGTTTAAAACTCCCTACTCCAAAAAATCGTGCAGCT
GATTTTAATTCTTTTTCTTTCTCAACTAGCCTCACAATCAATCCTTCTTTTAAATCAATCAACT
CTTCATCTTTCCTATCAAGAATTTTAATTATTCTTTCACAGTTGTTTTCAGAAGAAGGTAAAC
ATATTTCTTCTATCATTTCAGTTAAATTATTATGTTCTATATAATAGATCAATTCCTTGATGGA
ATTTCCTGAAGGATCATATTTGTCCTTTGTACATGCTTTATCTATGATTCTTGTCGAAACATTC
ATTTCTTGGCCCATTTCCACCATTTTTCCCAGTCTTAGATTCCTATAATTTGATGAATTTGAAA
CGACATTATTTGAGAAATTTCCTTTGCTTGCCATTAAGGAGAGTTCTTGTAATAAAATGTCCG
GAGCATCATAAAGATTTGGCATTATACCATATTTCTCACATAATTCGTGACAACTTTCATCC
TGAAAAGTGATCTTAAATCTTCTATTGTTTTCTTGTCGACTTTATGCATCCTATTTGTTCTTTG
TGTGTATTTCCTCAGCCCTTTATCTTCATTAAGTTCGGCATATATTAATACTTTTCCTATACAA
GACATTTTTACATTAATATCAGGTTCACTATTTATACATATTTTAACTAATTCCTCCGTTTTTT
CTGCCGAGATCATAATTGAATCCACCAAATTATCAATCAATGGCAATGTGGAAGAGTCAGAT
CTATAATCACTCATGAGTAGGCAAGTTGATTCATAAAGAGATGCAAGACCTACTCTTGATTC
TGTTTTAAGATATAGTAAATTTGTTATATAGTCGATAAATTTTTCTTCATTATTGTTTTTCTTA
TTGGATTCTATGTCTTTTATAAGCAACAAATTGGATTGATGGTCTGCTATAGCCATTAAATAG
TTCATCATATCTGCATTTCCAAAGAATACAATATTCTCACATGAGAATGCAGTGAATGATTG
TGACAGGAAAAGATAAAAGACATCTATCATATCACAGTCCTCAATTTCTATTTAGTAATTG
ATGATCCATCTGTGTAACATTGGGTATTGATTCTTCTGTTTTCATTGAAATTCACAGATTTTTC
CATTTTTCTTTTCTTTGTTTGTGCATTGTATGCCAGTTTAACTTTAAGATTTATCGTCTCAAGC
AAATTTAAGACCCATAAAGAGTTCAAAGTATTGTTTGAATATTCAGTTTTCTTTTGATTCAAT
TCCATCTTTGATGATTTTCCGATAAGATTATACTCAATTGATTGCCTCTCACTTAATTCTAAAT
TTTCCGCGTAATGCAATGCTGAGTCTGAGAATGTGCTTGTCTTTCCATATGAAGATAATATTT
GAAATATGTCAATTGAAGATTGTTGATTGGCACTGATCCTTGAGCAATCCTTTGAGAATTCTT
TTAGGAAATTCCTGAGCCTATAAATGTCTATTTTCATTCTCATTGTAGACTTTTCAGGGTATA
GATCTTCTTTAAATATCATATGTTTAAAATCTTCATTGATCAATCTGAATGGTGATTTTATTTT
TGACATAATCCATGGTTTGTCAGTATATTTCTTTGCCATTTCATCAAATTTCCATGTCACTTTA
TGAATGAGCTTCGATTCAAAATGAAATTCAGGCTCTGGAGGTTCAATGGCTTTTTCCTCTTCC
CTTGATTCCATCAATTGTATAAAAGATTCTTTTGATTTTCCAATGATGTCTAATAAGAAAGGC
TTGTCTTTTAAAGAAATCAATAACCCATCATATTCTTTTAGATAAACACTTGAATCTTTTTCA
AATAATGTCATATCAAATCCTGCAATTTTGTACTCTAACCATTTTTCAGGAGTGATGAGCAAT
TCTTTAGTGTCTGTATCATCATAACCTGAATCAACGCTTTCATCTGAAGATATATCACCCCAG

TTCGCCATAAGTGCTTTTGTGCTTAATGATTATTAATACTCCAAAATTTTATAATCTAATTTTC
AACTCATATATTTTTATTTATTTAAAACAATTAAAATTGATCAATCAAAATCAAAATAAACA
TAGCAATTAAAATAAATAGAATCACATGAAAGATAAACCAATTCAAGATCTTATAACTAAA
ATGATGATAAAGAAGACATAAAATAAAATAAAATAAAATAAAATAAAATA
>Seq13 [organism= Allium ophiovirus] RNA2 genomic sequence
CATAATTTGCATATCCTCACTTTGAGAACCGTTATCTTTTCAGATCCCGAATTAACAAAGTGT
TGGGATAAGACTTTTTAAGGGAACAATCCCCGGGAAAGATGCATCTGGCATTAAAATGAGA
CCAGACCGGAAATCTACATCTTTCGCATTTGTTTTTATTTAAAACCATAATAAGCATACTAGC
ATAACAGAATACCTAATTCTGATTCAATGATATATCATCACATACATTCACGTTATATATGAC
CCATAGGTTGGATGTAAGCATATATTCATCCTTGTAGAATATCATTAATAACTAATTAATATA
ATATAAAATAATATTATTATATAACATTATATTTAATCCCTAATCCTTTCTTCAAATCTGATC
CAGGTGGTCCGTCCCTCATTAACTCAACTTTATCATTCAAAATTTCTTTTTCTCCATACCAGA
TGCTGTCCTTCAACCACATCCTTGCAGGACAGCGTGTATCAAATTGCATCACAATAGTTTTAT
CTTTTTCTATAAACAGTGTGTCACCCTCAGATGACATACTGTCTACGAGACCCACCTCTTTCA
TTTTTGAATAAGAAAGTAGATTTGACCCTACCCTCTGATCATTGAATATCCAAGACTCTCCAA
GATGAAAAACTTCACCATCTACTAAGTAATCAACCTCAGCTTTCTCTACTGCAAAACAATCT
ATTCCCCCAAAATGATTCACTAATCTATGCACTTTTATCTTGTCAGGTGTGTAAATGTAATGA
TCTCCCGCACCAGTGTCCAACATTGTCTTGTAATGTGATTTTATTCTGACTCTTCTCTTCTCTT
GCTCCGTCATTGGGTTGTATTCAGGAACTTCAGTTTCTGGAAAATTATTTTGAACTATCTCAA
CCTCACTTTCTGAGGAAACATTGTCTTCAGAGCCGGAACTTCTAGAAATTTGCAAATTTCTCA
TTTTTCTTCTCATGTCATTTCTATTATCATTTCTTCTCTTAATCATCTCACTCCGCTTGCCTTTG
ATCTCATTGTGAATCTTATCTCCCAATCCCTTAAAGACAGATGTCGTGTTTATATCTATTGGG
GCATCTAAAGAATCAACATAAATTAGTGTTGAAGGTCTGATGGACAGCCTCATTGGGCTATG
TTCTTCATGGATGAAGAAGGCTATCATCATAGAAGCATATTCTCTTCCTTGAATTGGCAGCTG
GTCGACTGACATTATCAAACTGATTGAACTCATGTCTTTTTTTGCGACAAAGAAATTCATGG
ATAACTCTATCAAAGCCATCTCATCAGCATCAAATGAAACAGTCTGAATTATTGGGTCAAAT
CCAGCAGCCTCTTTCCCTGCATCAATCAGAGAGACTGTCATCTCACCATGTTCGCTTTCCTCT
TTACCAAACAATGGGGCATACATTATTTGAACTTTCTCAAACCTAACAAATGGTCTTTCATCA
TTTTTGATCACCTTTGAAAATATGGATTTCATTTTATCCAAACTGGTCAGTTTTAAATTCTTCT
TATGTTCTTCATTTTTCAACTTCAGTGTTATTGGGCTTACAATGGAATTTATTACTCCTCTCTG
AACTTTTGCAAAATCAGCAGCAATACATTCAACCCCTTCAGGCAATTCTCCAGATTCATCTTT
CAATGAAATTATACGCAAGTCCTTTGAACTTTTCAAATCATCCACGACAGCACTTCCACTTA
GCCTGCCTACTCTTGTTTCATCTTTCCTTTTGTTCACATTTGACTTGATCAAAGAAGTGGATCT
CATTGAAGAACTGGTTCCAAGGAAAGACATTCTAGCAAATAATGAAGACTGAGTTTTGCGA
AAAATAAA
>Seq14 [organism=Allium ophiovirus] RNA3 genomic sequence
CATAATCACTCTTTATTGCTTTATTTAAAACAAGCGCTATCATTTAAAATAGAAATACATTCC
CAAATCATGATCCTTTTGATCTCATACGATGCCATATACATTTTTGAGGGATGCGACACTGAT
GTCAGCAAAATCTGCTTCAGAATTCTCCATAATCCTCCAGACCCTTGACCCATCCACCTCTTC
TCCAGAAAAATTGGAATCATTCAGGAATGAATTATTCTTTGCTGTGACCATTTTGCTATAGA

GCTCGATCCTCCCATTTTCATTTAAACTCTCAATTATTGCCCGTGTTATTTTGAGCAACAATG
ATTTCACTACGGGCCTATTTGGTGCAAGAGGATGCATCTTTAACTGAGCCTGCACATTCCCAT
TTAAACTTCTCAAGAAATCAGAAATGCTTACTGCTAACTTTATTTTTGTATTCCATTCTTGAA
TTATTGCGATATCCTTCCCTTTCATAGTGTCATTGATCTCAATTTCTTTATTCTTCTCGCAAGT
CCCGGCAAATACAGCATATCTGATTCCTTTATTTCCAGCTATGCTCAATTTTGCTCTCGTGGC
GACAGGAACCGGAAACTTGTTTATGTCAATGTTTAGGAAGATTTTCGATGGAAATTTTTCGG
TATTTGCAGAAGATAATTGTTTACCTATGACTCCAAGATTTGCAATTTCCCCCTTGTATATCT
TGTCGGAGATGAATTTTGGAACCATTTTGTCATCAGTCTGTTTTGGAAGTGATCCTTGTATAT
AGACCAATATTGCTGCTGCTTGGATTGCTCTTTTTGCTTCCTGGACATATTTCCCAACCGAGA
GTTGAGTCAATTCAGTAACTTTAATCAAGCTAAACATTCCTTTGGCACTAGGAATTTTCCCCA
TGGAAGCATAAAGAAGAGCGTCTAGGACTTTGGTCCCTGCTCCTATTAGATTCATTACTTCA
GAAGTTTCTTCCTCATAAAAATATACTTTCAGCTCCCCTGTCAAATATCCTTCATCTTCTACA
GTGACAGATTCTAAGAATTTCAAAAGATGACTCTCTATAGCTTTCGAGTCAATAGAATATGA
ATCTCTGTTTATGCTCGATTTAAATTCAGTGATTGTTTTAACTTTTTCAGCCCCCAAGCTTTGA
TGTTCCGGAGCATCTTTTGTCATTGGATCCACAACAACACTTGCAGATGGCTTTTCTTTTCCT
GACTCCATCAAATAAGCAGTCTCAGTCAAATAGTGAATGACTTTTGATTGAATTTTCACCCA
TCCATTGGGCAATGAAGCAAATTTTGCCTTTGTCCTTGTCCTATTCTGAATTGCTGTTAGTTC
ATTTCCAAGACTTTCGAACTTTTTGAGTATTGTCAACTCTTCATTCGATCCATTGTTGTTATTG
TTTTCATATTTCTCAATTATTGAAACTAAATCTTTGATGGAGAGGTATTGAGTAATTCCAGCC
ATGATGAATTCTGAAGATGTGTTTTGAATGATTAAGTTTGCGCAAAAG
>Seq15 [organism=Arctotis ophiovirus] RNA1 genomic sequence
CGGTCCTGTATAACAGGATATCTTGTTCATGTGAGAAGTACCCACAATCAGGCGCAATGCCA
TAATTTTGATATCCATGTTTTGAGGTCATTATGAAGTTGGGTTAGTTTGGTCTTCTATTCTGTG
AGATTGTTTGTTTCATCATTCTGGTCTATTGACAAATCATGATATGATTTCTGAATAAGAGTG
AAGAGTGAAGTCGAGGTATCTAGTTACAACAAATATGTTCATCAATTAAATAAGATGCAATA
ATATGAAAAAACACACACTTATAAAGCGAAGTAAAATTAAAATTAAAATTTGAATAAAAAT
TATGGACCTTTTCTTCTAGCAAGTCATTTACATTTCATAATCTTCCCAACAATCCTCTTCCAAC
TCCTGTCTCATCTCTTGTGCTGTCATCGTAGCATTCTTTTGAACTTCTTCTGCTGTTGAACCAA
ACAATCCTGCTACTTCCATTTCTAATCTCATCATTATTTCGTCGTTGTACGCGTTCTCTATCTC
GTCCTTAGTATCGATGGGTAACTCACAAGTCTCCATTTCTCCTCTTTCAAAACTGGTTGTCAC
AACACTTGTTATTGAATCAAACAATTCATTGCCTAACCTATCATTCAAGTACTCTTCAAGAGA
TTCAAATTCTCGCAATGCTAATATTTCCTTTTGTATCCTTTCTTCTATTTCTTCTTCAGATACTT
TCGGTTTGTTTTGACCATCCCTTGATAATCTAATTGTCTCGTAGCTCCTTGATTTGTTACCTTT
GGACTTTCTCAGGTCTTGTAATTCTATTATGAGTTCCTTTATCTCTAACGGTTTGGCGATGAG
GTCTAAAAATAATTTTTCATGTAGCAGTATCATTCTATTCAACAATAGCTTGAAGTTCATGCA
ATCATACCTATGTGATCTTGTTTTGTTGTGAATTAAGCTTTTCATTGACCCTTTCCTCACTCCT
GCTAATTGTAAAAATACCATTTCAATATTTCTTCCTAAATATGCCTTTTTTGCTAGTTTTGCAA
TTTTTACAAGTCTGTCATATGAGTGATATTTGTCCCTAAATGAGGAGCCTGTTTCTATACTGG
CGTACAGTCTTTCTAATTCTACAGTTTCATCATTAACTTGTTCTAAACTCGTTTTAACTGTTTT
CAGTGATTGATAGGCCTCCTTTATTATGTATCTTTGTTGCATTAAATGATCTATTATTTTTTCA

ATGTCCCTTTTGTTTTTTCCTCTTATTCCCACTACGAAAGAACCAGATCTTATAGTGATGTCTT
CATGTATCTCTATTTCCGGGTTTTGATTTTTATTATTGTGGTATTCTGCATAAATTTGTCTCAG
TATTTTCTTGATATTCTTTTCTCCTGTTTTTGACCATCTGAATGTGGGGTAAGAAAGCTCTAAT
AATGATATCTCTCTGGTGTCTAGTTCTTTGATAACTCGCTTTATCTTGCCATCTACATTCTTAT
CAATAGTGTAGAATTCCTCTAGTTCCGTGACAATCATCTCTTTTATTTTTTTACCTTTTAGGAT
TTGCTTTTCGATTTCCTGTTCAACAAATTTGTTTTTGTTCTCATCTTCAGTTAATATCTTTATCA
AGGTCTCATCAGATATCAATTCTACTGTATGGTCATTTACGTCTACTGCAGACCTGTTGAACA
ATGAGTCTCTTCTCATATTGTATATTTCATTTGTTAGTTTTTCTGCAATTATTGATTCTGTAAA
CTTTTTCTTTCTTTCTTTTCTATTTTCTTCATCTTTTGTTGATAATCTCTGATTATTGATCTTTTT
ATTTCTTTTTGCTAAGCAGAGGTATATGTAACCGGGGGATTCGATCGATGGTATCAATATTTC
CCCTGATCCTTCATCGCATAGTGTTTGTAAGAATTTAATATCGTTACTTATAATTGAATTTAG
CCTCACCACTAATGTTTTGTTGTTGTCAAATAATTCCTCTATCATCATTTGTAGTGTTCTCACA
TTCCCTGTGTAGTGAGATACGTCAATCAAGAACACATCATGATTCATATATTGACCATAATTT
TCTGGCAAAGTGATGTCTGTTCCTGCCTTTAATTCGATCATTCCTGGCACTCTGTTTATGAGG
TTATATCCATCATTTCTTGATATCGATATATGTTTGATATTCATGTGAGTCATAGCGAAATGA
AAATCTCCTCTTCCTGCAAAGAGATCAACTATTGTTTTGTTTTCCAAGATAGGTTTAATTTGA
GACAAAACACCCACAGCAGATGGGTAAATGTCAGACATGGTGGGTGATGACACGTGCTGAG
GTTTGCAATGAATCGTGCACACCATTTCATAGCTCTTCAATGCGGCCGAAGTCATCTTTTGGC
TTGCAAAAAAATCTTCATCCACTGGTATAGTTTCATATCTGATTGCCCCAAAGTCTTTGGTGT
TAAATTCTTCTCCTATATAGTGCTTTATGTCATGATCGTAATATTGAACTAACATTTCTGTTTC
AAACACTCTTTTGTGAAATCTCCTATATATTTCATCCACTCCTTCAAACAATTCAGTCATTCT
GTAGCCACTTGTAGGGAAGGCCATCATTATTAATTCTGAGACCCTTTTGTCTCTTATATGGAA
ATGAGCTTGATTCCGCTTCATTCTAGCCAAGTTATTTATACCGTTTTGTTTTGTGTTATCTTTC
TTTATTAACATTTTCATACATCTGGGGTTTATTTCTTCTAACCTAAAGACTAAACAAGTTGAA
ATTATAAAAACTTTCATCAATGTTTTCCTATAGTTGATGGAGTAACCATAAGATTCCATTTCT
TTTATCCAATTTCCTGTGTCGTGGTGATCCCCTAGTAGTTCATTGTTGATTATGCTTCTCATCA
CAGTTTTCGTATAACTTTCATTTCCAATTATATTCAATCCTTTGTAAAATTTATAAAACTTATC
CAAATAGTTGTCCTTGTTTATGCTCATCCTAATCTCGGGGGCTATTTTTTCTAATGTATCATCT
GGTATCATCTCAGGCGATATAATTTCTTGATCTTCCATATATTTGTGTAAACTTTTGAAAGAA
GATATTTCGTCTATTTTCTCTTGTGGCATCACCGATTTATGATCAATGAATTTCCCCTTGATTT
CTTGATCACAAGATATAAAATAAGAATAATTTTTGTACAATCTTCCTTTTGATTTTATCTTTTC
CGTGCTCAACTCATCAGGGATATCTCTAGTGGGTTCCATTATAATGATCTCCCCTTCTGGCTT
AATTGTTTCCAGGTTGAAGCTAACATCTTTGATGTGTCTCAAAACGTCTTCTTTTATTGTGAG
TGGCCTTATTTCTGAATCTTTCGCTTTTAAGTGTCCCATCATGACCTTAGCGTAAACTATAAG
TAAGGATACATTTAGGTTATTGTCTACTCCTCCTGTTAAATCTATAAGCGTGTTTAAGCCGGT
TATTTCATACTGATTTGATTTAATGTTGGATGTAAGATCACCTGTTTTTGGGTTGAACCCTCC
CCCAATGGCTCGATGAAAGTATCTTCCTCCTTTTGGGCACACTACATTTTCAATTACGTCTTC
AATTCTACAGTCAGAATATGTTGCTATCGTGCTTTCAGTTATCCTCATCAATGATGCAATTGT
CTTTTCAGAAACTCTAGAAAACTTTTCCATATCCATTATTATCCATTTCACATATCTGACCAA
CTCATATATTAAAAAAATTAATTTATTGGAAAACATTCCTTCTATTTCTATATCTCTATCAAA

CTTTGTTAAATTCAAATGTGTTTTTGTGGGTCCGGCTAATCTAAAGTTTGCTCTGTTTTTTCCT
GTTCTTACTTTTCCGGATTTAGTGACAATGTTGATAACTCCTAGCCTGTCATCGCTGATCAGA
GATCCAATGATTGGTATTTCCTCAATCTTGAGGAATTCTAGTTGAATTGGTTCCCAGATTCCT
TCATTAGTAAGTGCTTTCTTTTCTACTTTGTCTCTCTGAGTTATGAGCCAATCAAAACCATATT
CATCTAATACTATTTCATTAATTTTATAATCTATCATATGATTAGCATTCCATGCTTTGTTCCA
TAATTTATTCATTTTTTTCTTTCCTAAAAAGAAGGACAATGTAGTTGAGTTGTCTATTTTTGA
GTATATGTCGTCTAAGTATGCATATATTGAGCATTCATAAAATTTACTAGTTATTCTGTGTGA
GAAACAAAATTGGAATGTAGACAATATTTCGTTCTTGAAAAATCTCTCATCATCTTTGTTTCC
TAGCTCTAGTAATCCTCTCAGTTGTCTGTTTGTCACTTTTGTCATCAAAACTTTCTTAAGTTCA
TCCTTTAATAATGACTTTGTGTCTTTTCTTCCTCCATCTAATGGAAATGGTTCTTTGATATAAT
TTTCTGCTTTTGAAGAGAGGTTGATTAGACCATACATTTTCCTTTTAAGTTTGTTGGACACAA
GGCTGGACACGTATGTAATTCGTTTGACATTCGAATTTGAAAACCCTGATATCATGGAGTCT
GGGATGCTTGTCATCGCATAACCATACATAGAATAAGGTAAGTACAGTAGCATAATCACCCC
TTCTAACAACTCTTTATTGTACATATTAAAATGGTAATATGATTCTAGTAGTGATGACTCGAA
TGATATTCTGCTTGATTTCGTCATTTCAATCATTTCTGTTTTGTGTTCTTGGGTTGGACTGTTC
AATTTTCCCAGACATATTTCTGCATATTTCCTCAATGCCTTGCTATTGCTCAAGAATGAGCTA
TCTAGTGATATTTCTAATTTGTCAGCCCATCTCAGTACTTCCTTCTCTATAACCGTCATCGCTC
GGTAGTTTCTTATCAATACTTGCTTTTTGATTGAATCAGCCCTCAGAGTAGAAGAAGTAAAA
CCGCTGTCTATAGTCATCATCCTTTCTAGGTCATCTCCCCATAAAATATCATTATTTGGACTT
ATGGAAAGGATTCTTTTGTAAGACATATCTATTGGCGTGTCCTTGAAATAATGGTTTTTTAAC
ATGGAACAGCGGCCATTTGTCACTTGAGTCTGTGATAATTTTACCAACAAACCTAATTTTAA
AGCATAGTTTTGCACATATCTTATTATATTATTTAAGCTATCGCTATCTAGTTCCTTTTTGACG
AAGACACCACATGAGTCATCGCTGTATGTTGTCCCAATATATTTTGTGAAGTTTAATGATCTC
ATCATGTCTTCCATCATTAATTGTGATTGTATTCCCCAGAGTGGACCAAACCAGCCTTCTATT
GCACCTTTCTGGTATCTTGATATCATGATATTATCTGATAATAGTTCTTCATATAAAATGTTG
ATATCTGAAAATAAGTATACTATCATTGTCATCCTTTGTCTTTCTAAAGATCCTGGTGCAAAA
CCATACATATCTGAGATTTCTTCCATGATAAATAGATTGTTCTCTGGTCTTTGCGATGTGTTG
TGACCAGAGTAGTCTAGAAAGAGAGAATACGAGTCTTCATCATCTAATAATTTTGACATTTG
ATGCATGGTTTGTCTTCTCTCATCTTCCGTCATGGTCATCATCTGTCCTGGTATTAGTTTCATT
GCTCTTTTAATCATCTCCATCAAAACAGATATGTATATTTTTAATTTGAAGCTTGCCACACCA
TAAAAGCGAGCTTTTGTCTTTAATTCTTTCTCCTTCTCGACCAATCTTACAATTTTGTATTTTT
CCATTTCCCCGATTTCGTCTTTGTTTTTCCTATCTGTTGTAATATATTCTCGTTCAAATTCTTTT
TTCACCTTACTTATGGATAATGTATTAATCAGTTCTGGTATATCATTTTTTGTAATATAATATA
TTAGTTCTTTCACTGAATTACCAGAAAAGTCATATTCATCTTTTGTGCATGCTTTATCAATGA
CCCTTGATGCTACGTTGACTTCTTGTCCTGGTTGTAACATCTGTCCAAGTTTAACGTTTTTGTA
GTTCGCTTGATTCGTGATGATGTCTGAATTAAAGCTGCCTCCGGCTGCCATTAAATTCAATTC
TTGTTCTAGTGTTCCGGATGCTCCACTTATAGGCGGTACGACTCCATATTTTTTGATATAGTG
TGTTATCACCTTCATTCTAAAGAGCCACCGGAGGTTCTCTATTGCTGATTCTTCAACTTTGTG
ATTCCTGTTAGTTCTTTGGATGTATTTTTCGAGTCCTCCATTTGTGTCCACTTCAGCAAAAGTA
TTTAGTTTTCCTATTCCTGACATTTTGATGCAAGTCTCTGCCTTTGCGTTGGAACAAATAGAT

AATAGTTTTTCTGTGTATTCCATGTTGATTGCTAATGATTCTGTCACAGCATCGAGCATTGGT
AAAGAAGATGAAGTACATTTTGCATCTGCTGATATTAAGCATATTGTTTCATATGATGCAGC
CAAGTCTGTCCTTTGTGGGTTCTTTACAGAGCTAAGCGAAATTACATAATCTATAAATGGATT
TTCTTCTTCATCAGACTCAAATTCATTAATTCGGTTGAGATCTTTGACTATGTTTATCGTGCA
GCTGTTTTCTATTATTGTGAGCAAATATTCTACCATAGTTTTCATGCCCACATACACCAGGGG
ACCTATAGAAACAGCAGAAAAGGTATGGCTCAAAAACATATCAACCTTCAATATCTCTTCAG
CATTTGTGGCCTTTTTCCTCAACTTTATTGTTGTTTTGCTTGAACCGTCGCTATTGAGCTGGCA
CTTAATCTTTCTTTCTTCATGCCAAAGATCCTTCGATTTGTATGTTCTTTTATTACTCATGCTT
GAATAGGTTAATTTTATTTTGTGATTAATCATCTCCATAAGATTTAGAAGCCACATTGATTCG
ATTGTTTCCTTTCTTAACTCTATTTTGTCTTCTTTTTTTATTGGTCTTCTTTTAATCATGGTTTTT
GCAATGTTTTCTTCTTCTGTTTTTCTCAGTTGGTCTGCAAATCTAAATAATGTTTCTAATCTAG
TTGACGGATGAGCATATTCTGTCAAGATGTCTTGCACAGATATCATATCATCTTGTGTGCCCG
ATTCCACAATGAGCCGTCCTTTCGTAAATTGTCTTAGCCTTTTTAGGTCAATTCTTGCTCTCAT
TCCAAACTTTTCAGGATAGTACTTTTCATTGATCATCTTAACTAAGTTGTCTTGTTCTATTTTC
TTGAAAGGAGAGTCAATTCTTGAAAGGAGAGCAGGCTTGTCTATGTAGTTTTCTGACAGATA
TTCTCTTTCCCATGGTTCATAGAACATCAATAGGCTTTTATAGCCCAGCTTTACGTTTTCTGG
CTCTTGGGCTTTCTCGGCTTCCTTTGATTTCATAAGGAGAATATAGTTTTCTCTTTCTGTTTCT
ATCATACTCTTTGTTGTCACCAAATTATTTTGGGTGAGAGTTTCTATGAGATCATCAAATTCC
TTGTTGTATTTTTTTGTATCTTCTTCGCTGATTTCTAGACCATCAATTTCTAGTGAAGTATTAG
TTTCTTCCTTTGTTCTGATGGGTGTAGAAATTCCTTCATCAAACTCACTGCTGCTCTCGACGA
TTTTTTCATCAGTCCATTCCATGATTCGATGCTTTTTGAAATGTTTGTTGTATTTCAGAAGGTT
TTTGTTAAAACGATCTAATGAGTGAACCTTCGTTTGTATTCAGATGAGTTGAATTTTGTGTTG
TTGTGTTCCTTACCTGGTATTCTATTAATTATATTGCTTTCCTTCCATTTTTCTATATCATTGAT
CTTGCTTGCTTTTCCTTCTTCTTCTGTTGCTGCTACTTCTTCTCGCAACAAATCAAGAACTGCG
ATTATATCATCTCTTATTACACTTGGTTCGCTTCTGAGCTCTTTAATTGCAATGGTTATCATCA
CTTTTTTGTCCTTCTGCATTAATAAATGATAAAGATTGCTTCCTATAATGATCAATCCTGTGC
TTGGCTCCAACTGTATCTCTTCAAACAAATTGAGATAACTATAATCACTCCATTTCACTGAAT
TTGAGTTGATGATCATTTTATCATCTCTGATTTGCATTCCCTCAAAATGATTGTTCTCTTCTTT
AGATATTGTTCTGAATTTTTCCATTAAGATGAAGTCGTTACAGCAGGCTCGTTTTGTTTCTTC
TTTTAAGAACCAGTTGATTCCAATTCCACCTATAGCTTTTATTATATCGCCACTTAATGTTTTA
TCATTGAATTCAACTATCTTTGATTCCAATGTTCTCATAACATGTTCAATTGTACTTTTTGCAT
GTTCGGTCTTGATTTTGTTGGCATTGATAGTTGTAGTTTTTGTCATTGCGAACACGTGGTACT
TAAAAAATGTATCGGTC
>Seq16 [organism=Arctotis ophiovirus] RNA2 genomic sequence
TTGATTACCTGATCACCGTTTTAGTGTCAGGAGGCGCAATGCCATAGGTGAGCTTCTAATCC
GGGTGGAGTGTCTTTAACTCAACTGGTTTTTATTAAAACGATTCAATATAACTCTTTAATCAG
AGTTTAGTAGACGCATTCATTCATGTTGTTTAGGACTAATAAAGCACCATTTGATGTTTGCCT
CACATCTCCGTCACATTATTCTCTCTCTCCTCTTGCTTCTGACACGATCTTGTTCACATAGCTG
TTTGCTCCCTTTTCACCCCCGCACGTTACCTCATTCCAAACATCATCTTTCAACCACATCCTTC
CAGCCTCACTTGTATCAAACATCATGATTATCTCCTCTTTCAGTTTCAAGAACAAAGTATTCC

CAGCAGTTTCCATTGAATCGACAAGGCCATCTTCCACCAGTTTGGTGTAGGATAATAAATTG
CTTCCAATCCTCATGTCATTAAAAGCATAGAGAGATGGTAGTTTCAGCCAGTTGCCAAACAC
AAAATAGTCTGCGGATACCTCTTCTACAGCTAACCTATCTACCCCACCAAAGTGATCAACTT
TGTTGTTTGGCCTGATCATCGGAACGTACAGATAATGATCTGCAGACCCAGTGTCAAGCATG
GTGAGAAGATTCCCACTTTCTTTGATTTGCACATTTCTTTTTGGTTGCTTGAAGGGGCTGGGT
GCCGTTCTTTGATCTTCGAGATATCTTGGTAAAACAGAAATACTTCTTCTGCCTTCTTCGATC
AATTCCTTGATATCACTGGAAGAACCAGATGATGTTGATTTAGTTTCCATTCGAAAGTTTCTT
TTCTCTCTGTTCATCTTCTTGCTCCTTTCCAACTCCCTTCTTCGGAATTCCTTCCTCTTTTCATC
AATATTCCTTGTAACATTGTCTCCAATTTTCTTAAAGACTGATGATCTTGTCAAGTCCTTTGG
TCGATTTACGGAGTCTATGTACAAGACTGTTGATGATCTCATTTGAGACTTAATTGGAATTGA
TTCTTCAAAAGTGAAGAAAGCAACATTCAGAGCTCCATATGATCTTCCTTCTATCGGCACCC
CGTTCGCTGTTATGTTGATGACTATCTTCTCCATATCCTCCTTCCTTACAAAAAAATCCATCG
AAAGCTCTACCAATGCCATTTCTGATGCATCAAAGATAACCTCTTGAATTATTGGATCTTTCC
CTGCGTCTTCTTTTCCCAGATCATGAAGGGATATCCTCATCTCACCATTTTCACCATCTTCCTT
TGAGAACAGAGGAATATACATAAACTGGATTTTTTCGAACTTGATAAACGGTTTGCTTTCTTT
GCCTCCCAATTTTTTCATGGCTGAAACAATTTTGTCCATAGTCGCCATTTTCAACTTTTTGTTG
TGTTCACTTTCTTTCAGTTTTAGAGTCAGAGGAATAACGGCGGTTTCAATCACTCCTTTATGA
ATGTTTGCAAAGTCCATTGTGATTTCTTCAATACCTTCGGGCATTTCTGAATCTCCGTCTTTCA
AAGAGATCACATTCAGAGCTCCTTTTTTGACAAAATTGTTTCCTACCGAGTCCGCAGATATCC
TATTTGGCTTGTAGATTGTTCCATCGAATCTCTTAGAAGCCAGTGCATTTTTTGCTGTTTTGTA
GCTTGCTCCTGATTTGACAGACATTGTTTCAGTGAGACTGGTCGCTATGGATTTGTGAATATT
TGGCAAGTAGAACAAGAGGTACTGAAAAAAAGTAT
>Seq17 [organism=Arctotis ophiovirus] RNA3 genomic sequence
AAACGAAAGGTGGAAATTGCCATGAAACCATGGGAAGATATGCAACTATCATCACTGGTGA
AAATGTCTTTGTTACCACAACCATTCAAGCAACTTTGTAGATGTTTTTCAGTCCCTCAGCAGA
TAGACCACCGAAATCAGCGTCTACGTTTCTGATGACTAGCCACTCTCTTTCATTTCCTTCACC
AGTCCCAAAGAAATTGAGGTCTCGTTTAAAGGCCTCGTTTTTGTCAGCTATGATCTTCTCCGC
CATCGCTTTTCTTCCGTTTTTACTTAAAGAGAAAACTATAGCACAAGTCAGTTTTAAAGTGAA
GTTTTTGATTGTTGGTTTGACATCAGACAAAGGATGCATCACAAGCTGATTCTTGTAGCAAC
CATCAAGAGAAGTAAGAGATTTTTTGAGTTCTTTTGATAGTTTCAGTTTTCTGTTTCCTTCCAT
TATAGCAGCTATAGCGACCCTAGGGGTGGTTTCAGTGATTTCCACTTCTTCAGCTTCTTCAAA
TCCACTAGCGAACACAGCATATCGAATCGCTCTGTTTCCTGCTACATTTAATTTGCATCTAGA
TGCAACCGGCACTGGAAGCAGATCAATGTTCATCTGCAACAACACTTTCGCAGGGAATTTAG
CGGTGCTCACATTGCTCAACTCCTTTCCAATCTTCTCCATTGTCACACTCTCACCATCATATA
ATTTGTCCACGATAAAGTTTGGAACTTTCCTTCCTTCTGATGTTTTGCTTCCCGGCAGACTTCC
TTGGATATATACAAGAACTATACCCGCTTGCAGTGCTCTCTTCCCTTCCCGAACGTTTGAAAC
AATTTCCTTCTCAGAGATCCCCTCTTCCACCCTTTCTTCAATGAACAAGCTGCTGTTTGCCAC
TTCATGCTTCAGACTGTAAAATATCAGGGCATCTAGGATTTTAGTACCAGCAGCGATTAGCA
TTGTTATGTCATCATCTCTGGATCCAAGATATCTCACGAAGATGTCTCCTTGTCTGTAAGCTT
CATCTTCCAAAGTTATGGTCTTCACAAAAGCTTCTAAGACTTCCTTGAGTCTTGTTAATTCAA

GAGTGTAGCTTTGTTGCTTTATTGTCTCGGTTATCTCTTCTTTTTAGTCAATTTCTTGTCAGA
TAGTTTAGTATTTGCAAACCTCTGCTTTTCCTCCTTGGGGTTCTCGTCTGGTTTGGTTCCAGAA
CTGCTAGCTATGGAAGAGAGTTTCTTTCCAACCATCACCACATCACCGTTGGGATTAGTGAC
AAAATACTTTTCCATAGCAGTACAATTGCTAGCGACATAAGAATTGTTTTCATCAAGTATCC
CAAATTGTTTCAATAGATCAACTAACTCGGTTGGTGCGTTATTCTTGTCCTTGGCAAACTTGT
TAAAGGCTTCATGGAGTTGAGAGACTTTGATCTTGGATTCCATTGTTCGGTGATTTCTTGCAA
CGAAGCAAAATAGTAC

>Seq18 [organism=Boraginaceae associated ophiovirus] RNA2 genomic sequence

ACAGAATATGCGATCCTCATAGCAATGGATCTTGCTAATGAGTGGATGGATCGACTTATAAG
CCATCCGACTATGAGCGCAATGCTCACGGAATAGGCAATTGGAAAAGATCCAAATCTCCTAT
TCACAGTTGTTTATTTAAAACGATTAAAAATAGTCACTTTAATCAGTGATCTTTGAGGGAAA
ATAGGAAATACAAAGGACGACCTTAAAATATTTTACATATTATGGTATGTTGTTTAGGCTCT
TCTTCTCTCTCACAAGCCTCTCGATGAGTGCTGGACCGCCCGAAATGACAGCGGTATCATAT
GCATCATCCTTTAACCAAATCCTTCCTTCATTGGAACTGTCAAAAGAGAGGACAATTTCATCT
CCCTTCATGAGGAATGCACCATTCCCAGCAGAAGTGATGTCATCTATGATTCCATCTGCTTTC
AATTTGGAGTAAGAAAGGATGTTGATGCCGAAAGCCAAGTTTGAGAAGAAGTTTACCTCAG
AAAGATGGATCACAGAAGAGCCTGTCTTGAAGTTCATTGATCCTTTCTCTATCTCAAGGCAT
TCTACACCTCCAAAGTTGAACTGCTCTTCATCAGCATTTATCCTGGGCCTGTAGAAATAATGG
GCCCCAGAACCGGTGTCCAGTGCAGTCAGAAGATTATGGTGTAGAAAATGAGGGTAGCAAT
TCCTTTTGTTCCCTGGCCTATTCATCATTGCATCAAGAGGCCCTGTCCTTTCATTGTGGATCTC
CAGATCAGAGAGATACCTTGACACTGCATCAACACTCCTCCTTCCAGCTGCAATAAATGTTT
CACCAGAATCACTTGATCCTTCACTGAAACTATGCCTTGAAGATTCTCGTTCAACTCTGACTC
CTTTCAGCCGCTTTTTCTTCTCAACATTATTCTCTCTCGCCTTCTTCTTAATTGATTCTCTCTTC
GTTTTTATTTCATCCTGGATCACTTTGCTCACATTCCCAAGAACATTCTTCTTGTTAAGATCTT
TTGGCGCCTTGCTGGGGTCAATGCATATTATGGTGCTTTTCTTCTTCTCTACCCTCAATGGTAT
CGCTTCTTCATGTACAAAGAATGCCATAAACAAAGACCCATAAGCTCTTGATGAAACAGGA
ACATCACTTGCACTTATGTTAACAACAATCTGATCAAGATCCTCTTTTCTCACAAAGAAATCC
ATTGATAACTCAACAAGAGCCATCTCATCCATTCTAATTTCCTGTTCTTGAATCAGACTTTTG
TTCCCTGCTTTTTCTTTCCCCATATCCTCAAGTGACACAGTCACTTTCATCTCGGAAACAGCC
TCATCATCATCAGACATGAGAGGCAAATATAGGAATTGAATCCTTTCGAACCTAACAAATGG
TCGACTATCTCCGCCCAGCTTGTCAAAAAGATTCTTGACTGATTTCAGTGTAGTCAACTTAAG
CTTCTTCTTATGAGTCTCCTTTTTCAAACTCAGAGTTAAAGGGCAAATAGCAGACTTGATGGT
GCCCTTCTGAATCTTGCCAAAATCTTCTACAATGTCCTCAACTCCTTCTGGGAGTTCATCACC
GCCAGAAAGAGAAACGATGGTGGTATCACTTAATTTTGAAATTTCATCCTTGATTTGAGTTG
CTTTCATGCGAGGCAGAGCAACTTGCTTCCCCTTCTCGCTGGATCTCCTTGAAATCATGGATG
ACTGAGATGAGCTCCTGATCGACATTCTCTGCAGATAGCAATATGC

>Seq19 [organism=Boraginaceae associated ophiovirus] RNA3 genomic sequence

CTTTTGTCACACGTGTTTATTTAAAACGATAATCAGTGGAAAGCTGAAAACATAATATGCTT
ACTGAGAAACATGGGACCGACCCCTGGATCATGGATTTTGATCTAGTTCAATCCATAGATCT
CTCTTATTGTATCAGGAGTCAATGAGCTGAAGTCTGCATCAGGGTTCGTCATGATGGGAAAT

GTGTGAATACCATCCTTGACCACCCCTACAAAATTCGTGTCCCTTTGGAATGACAGATTTTCA
TCAGCAACCGCCTTCTCAAGGATTTTGTGACGCTCATCCTCGTCAATGGACACTAGTATAGCT
TTCGTCAACTTCAAAGTAAAATCCTTAATTACTGGACGATTGGGTGCTAAAGGATGCATTCT
CAGTTGTGTCTTATAATTTCCAGCTATCATCCTGAGAATGTCAAAGATCTTGTTTGCAAGATT
AACCTGCTTGTTAGACTCCAACATGACGGAATAAGTCATTCCATCCCATTCTCTCCTTGGCTC
AACCTCTTTGCTTCTGGCAAACCCTGCAGAAAAGACTGCATACCTCACCGCCCTATTTCCTGC
AATGGATAATTTGCATCTAGTGCATATAGGTTTTGGAACCTTGTCCAAATCAATGTCTAAGA
ACAGCTTAGCAGGAAATTTCTCAGTATCAGTACTAGAAAGGATCTCAGCTACTCCACTCAGA
TTGGCTATATTCGACTTGAAAATCTTCTCTTTTATGAATTTAGGGACAGGCTTCTTAGAATCT
GCCTTGCTAGGAAGTGATCCTTGTATGAAGACAAGGACAAGCCCTGCTAGGAGAGCCCTCTT
AGCATCAGTCATGTGACCAGAGATCACATTTGCAGTAAGCTTTTCGACCTTTTGAACAACAA
AAAGGTCCTGAGCTTCTGGCTTATCGTTCAATGCAGCATATATCAGTGCATCCAGCACTTTTG
TCCCTGCTGCAATCAGACTATCAACATCTTTCTTACCATTGTAAATATATACCTTGATGTCAC
CAGTTCTGTAAGCATCGTCTTCCCACTTTATCTCATATAAGAACCTTTCAAGGTGGGCTCTTA
TGTCAGCATCAGTTACCACATAAGAGCTGGAAGTGATAAATTTATCCACTTCAGAATCAGTC
AAAGTTCTCCCAACCTCAAAAGTCCTGTGAGGTCCTTGAGATGAACTTCCATCAGAGGATGT
TGACTTAACAACATCATCCTCTCCTTTCCCCAGCCATTCATTCATGTTCACACCCAAGTAATC
ATCAATTGACTGACTCATAGATGAGGGATTTGCAGCAATTTTCAATTCATTGTTCAGTAACAT
AACATCACAGTTCGGATTCTTAGCATCCAATTTAGAGATTGCAATGGTAAGAGTGGAAATTC
CTTTTGTGAGGTCAGTAATTCCAAATGCCTTCAGTGTGGCTTCTATCTCTCTCCTTTTCTCAGC
AGTGATATCTCTAGTCCTTTCAGTGACAAGTTGTTTCAAAACTGCCAAAGGGATAGTGGGGA
TGTTTGAACTGGTAGACATCTTTGCAATTCACTGAGCTTTAGAAGTCTGGTGGTTATCTCTCT
GGAGTAAGTGAAGTAGAGGCTCTTT
>Seq20 [organism=Buxbaumia ophiovirus] RNA3 genomic sequence
CCCTTATAGGTGGTTGCGCATTCCACACTTTGCTGTAATGGTGAATTGATCACTGTTGCAACA
GCCCAGTCATGCATTGGTTCCATGGGATTTGGGACAATGGCTTCCAGTCATTACTGACTTGA
AGGCAGGCTGGGGGGTCACCTTTGAACGATTCATCTTTCTCATGTGCTGGGACCATGCGGAG
AGTTTTTAAGAACTTATCACGATCACCGGGGATGACTGTTAGGAGGATGAATTGGCAACACC
TCAATTGGAATTTCTGTCCGACACATCTCTTTTGAGGCAGTTGTTGTGATGGGTGAAGGCTCA
TGTATGACATTCCTCCCTCCACGGCTGAGGCAAAGGCGTGTTCTACCATGGATGCTCTAGAG
AATATGTCATCCGCATCTTTTTTGCTTGAACCCGCCGGGCACAGGCCTACATGACTCCTTGAG
TAGTTCCTGGATAATTGGTAATACTTCAGAAATCTTGTCCCGGCCAGTCCCAAGACACACCT
ACCCCTAACCTCATATGGTAATGATGAGAAGGGGATGCTGGCAACCACATCAATGGGAAAA
GCAGATATACCAGTGCTGGACAGTGTGCCCGCTAGATCTTGGATGCTGTTTGTTTTAGACTCT
AGTAGCACATCCTTGATAAATTTGTTTGTGCTCCCTGTCCCGACTGGAGCACTGCCAGTACA
GTACCAATGCACTATCATTGACATAAATGACCTTTTTGCAGTTTTCAGGTTGGCATTTATGTC
CACGCTGGATGGGGCCTTCGGAAGAATAGTGAGATTGAATATTTGGCTTCTTTCCTCATCTTC
ACGCAAAGAACAGAAGAGTATAGCATCCAACACTTTGGTGCCTGACCCTATGATGGAGTTC
AGGGTTACCTTTGATCCATAAGTGTAGAAGATCAACACTGATCTTGGCATATAATCATACAA
ACTGCCAATTGTGGATGCGAAGAAGAGGAACATTGAAGCAATCAGACTCTGATTTATGGAG

ACTGGTTGCTCTGAGAAGACCCTGCTAATGTCTTCATCTGGGGTGTCAGATATTGCATTCACG
GCATCCAACACTTCCCACTGCTCTAAGCCCTCTAGGGGGTTTGGATCAATCGGTGCTGCTTGT
GGAATGGGCACAAAGGAGCTGGTGGAAGGCCCCATCAAACCTTTCCCTTTGAGCTTAGATTG
GGAATCATACCATTCCCGAAGTTTCAAGACAGCTTCATTGGGGTCAGGTTTGTTGCGTTCTAT
CTTTGGAGGACCTGGGTATTTGGGCTCCAGGATCCCTGTTGATGTCTCTACGTATGTTGTTCC
TGCAGGCACCTGCACCGATGCTAGTGTGGTCTGCAGGACATCAGTATCTCCTGAAGCCCAAG
CCCTGTCCTTCAGTTTCTCAAATTCATCATGATCCTTTTTCCGCTGCTCAGCTTCTGCTCTTTG
GGCCTCTGCTTTTTCCTGCTCTTGCTGAGCTTGGAGCCTCATCCGTTCTGCATAGTCCTTTCCC
GAATCCATATCGGCTATTTCACTTGTGACTGTGGGAAATCCGCAATCAATACAAACTTTTTGT
GTTCAGACGCTCGAAAGGCAATGGCTTG
>Seq21 [organism=Caladenia ophiovirus] RNA1 genomic sequence
GCTAACTTGTTAGCGAGTCGCAATGACTGTAGTAATAAGAGAAAACAAGAGACAAATCAAA
CAAGAAAAAAGAAGAGAAGGCAAACTAGCAACTGTGTCCCCAGCGGACAAGAACCCTGGTT
TTGATAAATTCATACTACTCATAATCGCCCCAATCCTCAAGTTCCAAAGCTTCGTAAAATTCT
TCCAATCCTACTTCCTCCAACACCTTTTCATGGGTGTCCATTTCTCTATTTCTGAGTATTTCTT
CTTCTGTGACTTCAAATGGTAAAAACTTTATGATTGTTTCTGGATCTAATTCTTTTTGTTCCAG
GAGTACATTTTCTTGCATTTCTAGTATGCTATCACTGAGTCCTCCTAAGAAATCAAATGTATT
GTCATTCTTGAAGAATTCACTTAAACTTTTGTAGTTTTTCATTTTTTCAATTATTTCTTCATCT
CCCAAATTGATATTTATCTTATTGTTCCATGCTTCTTCTTGCTTTTGATAACTTATACCACACC
AATAATTTTCATCAAAGTAATCAGTGAGACACATGATCCTGTTAATTATTAGTTTATAATTAA
GAATATTGGTTTTTGTTGTTTTGTCTTTATGCTTGAGCACTGTTTTTCTTTTCAAGCTTTCTAG
ATTTGCTATTTTTAATAGTCCTTTTTCTATTGTATTTGTTTTATAAGAGAATACTGCATGTGAT
GCAATTGAATAAATATTGTTTAGCGCTTTTATTTTGTTTTTCTCTAATTTGCTGATTTCTAGGC
TTTCTACCATTACTTCTGTGCTTATCAATTTTGGCCTATTAGGAAATTCTAGTATAAACTTCCA
ACACTCTTTTGTCAGTTTTCTATTGTATTTATTAGAATTTATATTTTTGAGAATCTCTAAGAAG
ATATCTTCTGGAGTTTCTGTTATTCCAGTGATTATTTCATAGCTATTTTCTATTAATCTTCTAT
TTACTAAGATTAATGATTCATTGGTTCTAAGATCTTTGCCTAATTCATATAAATTTTGAGCAC
CTTCAGTACCTTTTGTGACAATCTTTTTGGTTGGCACTGTGATATATTCTTTGTATTTTCTGAA
TAAATCTTTATCGACAAATGCACATAGATTGTCAGCGAAGTCTAGACTGTTTGTTACCTTAA
ATATCTCTGGTGTCCTAATCAGTTTTTCTTCATCTAGCATAAATTCTAGTATTTTCTCATCAGA
AATTCCTTCTTGTGTTTGATCTTTTACTTTTATTCCAATGTCACAAAATAAATCTTTTGTATTA
CATTTACCTATTTGAGTTGTCAGTGCACTTGATAACAATGATCTGTTATATCCTTTTTGTAAT
GAATCAATGTTTTTTGTAGTTCCTTTTTCAAAATCTATGTACAAATATATGTATCCTGGAGAA
TTCAATTCAGGGATAAACACTATACATTCTGTATCTAATATTTCTTCTACTATTTTTTCATTAA
AGAATTTAAACATACCATTAAGTCTCATAAGTATTTTTTTCTTGTTGCTCTTGAGGTCTCCTAT
CAATGAGCTTAAACAATCTTTCTTGTTTGTTATATGTGATATATCTAACAAAAAGAAATCATG
ATCAAAATATTTCAAATATTCTTCTCTCTCAAAACAATTGAAAGTAACCTTTTTTGATGTCAT
TCCTGGTATCCTGTTAGCTAAATTGTAACCATCTTCCCTTGACAATGTGGTATGATTTATTTCT
TGTTCTGACATTGCGAGATGGAAGTCACCACGACCCCCGCACAGTTCTATTACATTTTCTTTT
CTTGTGAGTATTTCATCACCAATAATTCTTTGCAATATACCTCTTGCACTAGGGTATACAGCT

GACATTGTTGGTGAAGAAATATTTCTTGGACTTACTGAAAGCTGGCATGACATCTCAAATCC
TTTTATTGCAGAATTTATTCCCTTTTGATCGATTAGATCCATTGGATTGATGTATAATTTGTTA
TAATCTACTATACCATAAGTCAAGTCTGTGTTTTTATCGATGTAACCTGGTTGAGTATCCAGA
AAATATGAGCTGATTATTTGTTGGTCAAATTCTCTATTGTCTACCAAGAAGCAAACTTCTTCT
GCTATGTCTTCTATTTGTTCATACTTGTATGCTAGTGTTGGAAATGAATTAATAATTGTGTTT
GTTATTCTATTATCTTTAATATAGAAATGGAATTTACCTGTTTTAATTTTATCAAAGTTTTGTC
TTGCATTTAGTTTAGTCCTATAGTAATTTACTTGTATGTTAATTGCGTTATTGTTTTCTATGAT
TTTATAACTGAGTGAAGTGGATAAAATGAATAATTTCATTAGTGCTCTTTTATATGCAGAAG
AATAACCGAGATGTGTTATTTCTTCAATCCATAAATTACCTTCTAGATCATGACTGAACAATT
CTTCATATAACAATCCCCTAATTATTGCTCTTGCTGGTGTCTCATTATTTATGATATTCAGTGT
TTTATAATAGGAATAAAATTCCCTATAGAAATTTTCTTTTCCTATTTTCCAGATTTCAGGATC
CAATAATCCTTTGAGTGTTTCATCTGCTATCATTGTTGGGCTTAAAATAAATTGATCTAACAT
ATAATTATATAATTTCCTGAAACTTGATTCCATTTTTATGTCTAACTCTTTTTGTATTGAATTG
TGATTAATGAATTTATTCTTCAATGTATCATCATAACATATATAACTGGAATAATTGTAGTAA
AGTTGTCCTCTTTTGAGTATATTTTGTTTTATTGCCAAGGGAAAGGTTCTCATTCCTTGGAATT
CAAAGTCATTTAGGCTTAAATTGAATGTTACATCTTTTAAGTGGACAATCACGTCTTCTGTTA
TTCCTAGGGGTAATAATTCATAAGGTTTAGGTTCTAATATTCCCAGCACTACTTTTATGTATA
TCATTAAATATTGTATGTTTAAATTATTATCGGCACCCCCTGTGATTGCCAGGATTCTATCAA
TTCCTGAAGTGTCATACTTATTAGTGATTAGGTTAGATGACATATCACCTGTCTTTGGGTTGA
AACCTCCTGTAAGTGCCCTATGGAAATATCTACCTCCTTTTGGACATACAACATATTCTTCTA
TATCTTCATATTTGATATCAGAGAAGGTAGACAATGTGTAATTGCAGGTTTTTATCAGTATAT
TTTTATTTGTGTCAACTAATCCACTAGAGAATCTTTGTATCTCCATGATTATCCACTTTGTGTA
TCTGACTAAGTCATACGCTTTAAATATTAATTTATGTTCAAACATTCCTTCTATGCCTAGTTCT
CTGTCAAATTTTGCAAAATTGTGATGTGTTTTGCTTGGTGGATTTAACACTCTTCCATTTTGTG
TTAATTTTGTTTCACCCTTATATATTGGTTGTATCAAGTTTCTGTATGGTAGGTGCCTCACCTT
TCCCATCAATGGAACTTCTTCTATTTCTATGAATTTTATTTCTATTGTTCCATCTCCCATTCTTT
TTGTTATTGAGTTACGTTCAGCTATTATTTCATCATAACTGATGTTTAGCTGTTCACTTTTCTT
AACTGTTAAATTGTAATTTGATTTCCATGCTTTGTCTATTATTCGTAATAGTTTCTTTGCTCCT
AATAACATCTTCATTGTCCCAGCATTATCTATTTTTGCCAATATTTCATCTATATAACTGAAG
ATACTTTCTTCAATATATTTGCTAGTTATTCTGGAATTAAAAGAATCTTTAAATATATTTACA
ATTGTTTGTTTGAATATTAATTCTTCTTTGTCATTTTTTCTATTAATGTAATCAAGTAATTCTT
CATTTTTAACCTTGTACTTTAATACTTTGCTTATTTCATCCTTTGTATAGTTTATTGTATCTCTT
CTTCCTCCTACCATTGGGAATGGGTTTGTCAAATAATTAAGTGCATTCTTTGACAAAGCCACC
AAGGAACCGATTATTCTTTTATTTGGTATACTCAGTGCACCTTGTATATACATTAATCTCTTA
ATATTAGATAAAGAATACCCTGATATCAACGCATCAGGCATTGGTGTCATTGCATAGCCATA
TGTTGTATATGGTCCATACATTATTGCAATTACTAATTCAAGAACATCTTTATTATCTATGTT
GAACTTATAAAATCCTATTGTGTCAGATCTTTTGGCTGGAACTCTAAATTCTTTTGTTGCACC
TTTTTCTATAAGTCTTAAACTTGTTCTCATTTCGTTGTCATAGTTGTGTGATTTAGATAAATAC
CTGCTATCGATGTTTTCTGTTAACTGGAGTATTATATCCTTCAGGCTTGAAGCTAATAGTTTT
ATAATTCTAAAATTTCTCATGAGAGTTTGTATTGTTATTTTTCCTGATCTCTGACAACTTGCCG

TATACCCTGAATCAATTAATTTTGCATTTTCTAATTGATCATTTAATATTTTTGCACCATTAGG
ACTGATGCTCATCATCTTCTTTATATTCATCTCTATAGGAAAACCTTTATAGTAATGTTCTTTT
AACATTGAGCATCTACCATTTGTGACTTGTGTTTGGGATAATTTAACTAAAAGTCCCATGTCT
CTTCCATATTTCTGAACATTTTGGATTATTTCATCCAATTTATATTGATCCATATTTCTTTGTG
TGAAAACTCCACATGAATCGTCTGAATATGTAGTACCAATATAATCAGTTATTCCCATTTGTT
GGAACATGTCGTCTAACATTAACTGTGATTGGATACCCCATAAACTTCCAAACCACCCTTCA
ATTGCTCCTAATTGTCCTTTTGAATAATATGTATAGTCACTCCATGCATCTTCATGGATGACA
TATATATTAGAGAACAAATAAGACAATGATGTGATTTCATCATATTCACTATCTCCTTTTCTG
TAACCATACATGTTTGCAATCTCTTCTAATATGAAGTTTGTGTTTTCAGGTCGCTGTGAAGTA
TTATGACCTGAATAATCTAAGAAAAGTGAGTATGCATCATCAGTATTTAGTTTAGTAGCCAT
TTTGTACATTGTATCCCTTCTTTCATCTTCTGACATAGTCATCATTTGTCCAGGTAATAATTTC
ATTGCTCTTTTTATCATTTCCATTATGCTGCTTATCCAGAGTTTGAGTTTGAATGAAGCTATTC
CATAAAATCTTGCTGCTTGTTTTTGTTCTTTTTCTTTTTCTGCTAGTCTCACTATCATATATTTT
TTCATTTCTTTTAACATGGGGTCATCTCTTCTTGTAACCCCTACTATCCTGTCTCCATTCTTAT
ACATTGATTTATCTATTTGTATTGGATCTTTAAATACCTCTCCCATATTATGATTAATGTAATA
CTGTATTTCTTTTTCTGAATTATTTTCTATATCATAGTTATCTTTTGTGCAGGCTTTGTCTATTA
TTCTAGTCTGTAAATTAGCTTCTCCTCCTTTATCTAGGAATTTGCCTAATATTATTTTGCTATA
AGCACTTAATTCTCTTACTATATTATTGTTGTAGTTTCCTTTAGCAGCTTTAAGTTCTAATTGC
GAGAGTAGTTCTTCTGGAACATTGAATAGTTGTGGGACTCTACCATGGAATTTAATATATGA
AGAGATTACTCTCATCTTGAATAAATTCCTTAGTCTTTCAATAGTTCCACTTTTTACATTATG
ATCCCTATTAGTCCTGGTAGTATACTTGGTTAGGCCTTCTTTCGCTTTTGTATTTGCTATAATC
AACACTTTGGCAAGACTAGAGAAACGAATACAAGTTTCTCCACTAGCTTTATAACAGATGTC
AATTAATTCTTCTGTCTTATTTTTATCCACCTCCAATGACTCAATCAAGTTGTCTAATATTGGC
ATTACTGCTGAGTCATTTTTCAGATCTGCTAATAGTAGGCAAGATGGTTCATATAAACTTCCT
AGTTTAATTCTTTTCTCTTCTGGAAGATGAATTAAAGACTTATAGTATTTTAGGAATGATCTT
TCACTTTCATTTGATAAACAGTTGTTGATTATGTTTAGACTTAACATATTGTCTATCAAGTAC
ATGGAATAATTTAGCATTTCTGGCCTCCCTAGATACATTGTCCCACTGTCTGAGAAATAAAA
CGCTTGGATAGTTGATGATAAGTATATCAATAATGAGCATTCCTTTTCTTCAAAATCAACTCT
TGTTATAGATGCGCCTATTGGATAGTATCTTGTGTTTATTCTTTTCCCTTCATAGAATTCTATA
TTCTTTCCCAATTTTTTGTTGTTTACATATGTGCTACTGTGCATCATGATGTTTTTGTTGATTG
TCTCCATTACATCTAAGCACCAGTACCACCTGTAAGCTTCTTCTCCTAAGTCATACCTGTTCT
TGTTGACATCTTTTTTCAACATCATGTTTGCAAGGATATTCTCTTCTACGTCCTTTAATTTATG
TCCAAAGTTCCACAATGTCTCTCCTTCTACAGATACCTTCGAGAAGTTAAATAGTTTTTTCAT
TAATATAAATGATGCATCTTGAGTTGAGACAATTCTAGGAAATGCATTTAACTCTTCATTGA
AGTAACATTTTCTGAATAGATACAATTCAATCCTGGCTTTTGTCTTCCACTTAGTAGGGTATA
GATCTTCTTTCAATATAAAGCAAAGATTTTCTGGGGAAATATCTTTATAAGGAGTTTCTATTT
TGCTTATCAAAGCAGGGGTTCCTTCCTTGATCGGTAAATTCTTTTGCTCTTCAAACATAACTA
ATTGATTTCTGAATTCAATTACTACTTCCTTTGCTTCTTCTTGCTTCTCTTCTTCAATTGACTTT
GTTAAGTCAACAAAGGAATCTCTGAACTTTTCCAGTAATCTCATCGACCTTGGATTTTCAAAC
TCATTCAAATTCAGCTTAGCAATAAGGGCATCCATCTTTCAAGAATTTTATTTAAAACCCATA

TATATATCACTTTAAGGCTGAGTATAATTTAAATAGATCTTTCCTCTTTATCATTTCATTTAAC
ATTTCTGGACTTGGTAATATCTTACTTTGTGGTATCTTGATTGATTTGTTTTCAATAAATATA
TATGTGATTTATCATATTGTATGACAAGATCTTCTTGTATAAAATATGATAAATATTCAGAAA
ACATTTTACTGAAATTACTCGTGCATATAACTTCCATGCCCATATGCTCATTGGAAAACATTA
TTTCATTAACAGTCTCAATAATTATTATTGCCTTCTTTGTTAAATGTTTATGGTCTGTCTCGCT
TAACAACCAAGATCCTAATAGGAAGCGACAGTATTCAGGATATTGAGATTGATATTCCTGCA
ACTTACTCAAATCAGCTGAATTGCGATGCCAGACATTGATTGGAGGGGTGATTTTGTTCTCT
ATTTTCAGGGTCTTTACAAATGACTTTGCATTGCAGCATTTAAGAGCCTTGCACATTTTCTTT
TTGCTTGTAGCATACTCATTAGATGAGGAACCCATTGCTTTTTG
>Seq22 [organism=Caladenia ophiovirus] RNA2 genomic sequence
GATCTCTTATCTCTTTCTCCGCTAAAGGAATCTTCCATGACACAAAAAATATGGTAGACAAA
TTACAACAGATACCTATGAGCGACAGAAAGAGATAAGAATGTTAAGATTCCGCTGAGTGGG
ACTAGTCGCTAAGCCACCCAACAATGCTGCACCTCCCCCCATGGATTAAAGGGCCGCTCATG
CTACGTGATGTTAGGGAAAAATTACGTACGCATGAGTTATCTGTGTCACACGGGGGGCATTA
ACACAGAGTACCGGGTCGCAATGACCGTAGTAATGGAAGACTATAGATCGCAATGATACGA
TAGTCTTCATAGGTTTTTATTTAAAACACAACAAACCTGCTAAATTACCCCCTACTTACCAAG
AACTCTTTCTGAAGTGTGATTAACACTTTCAAAAATCATCCTACCATGTTCAGTGTGACGTAA
TTCAAACATTAATTTATCGTCTTTTAACAGAATTGATCTGTCAGGGCTAATTGATTGGAGACA
GTTGATGATTCCATCTTGGACAAGCTGAGTATAGGAAATGAGTGGCAAGTCTGCTGAGGAAC
CATACAACACATTTTTGAGATTCACCCAGTGACACCCGAGCTTGAGTTCAAAATCCCCGATT
ATCCTGGTAAATTTGGGAGGATTGCCACACATCTTGGACCCTTCTTCGACTTTGCAAGTTTCA
TCAGGTAAAAGGTTTGGATTATAAACCCAATGATTGGGAGCTGCGGTGTCTAAAACTATCGA
ATCTTTGAACATAATTGGCAAGTTTCTTGGAAGATAATTCCCTATCTCACTAGGTGTGATGGT
CACAAGATCATGATCAATACTTGATTCAGATTTGTCCACACCACTTGTACCCTCGTCACAAAT
GGCTACTTTTTCCTTCAGTTTCTCCAGCTCGTAAAGATAGACAGCCCTTCTCTTCTCTTTTTCC
TTTTCTTTCTTTTTCAGGTTCAGTCTACTTGTGACCTGATCACATAATGATTGCACAGAACTG
GTAGTATTTATATCCATGGGACGATCTAATGCATCAATTTCAAGAGTTAGAGAATTTTTTCTC
TTTAATGTGCCAGAAAAGAAATCCCGATGGATGTAAAATGCAACCATCATGAATGCATAGG
ATCTATTTCTCACTGGCACCGACCCTGCATGATAGTCTAACTCTATGAAATGTAAGTCTTTGC
GCCTAACAAAGTAGCTCATTGATAACTCAGACATGCTCATCTTGTTCATGCTAACTTCAACG
GTAGAGATGCTCCTGTCCCGCTCAGAAATTATTGATGTGTCACAGAGTGAGAATCTCATTTT
CTCGCCTTCATTCAAGCTTTCATCGAATAAAGGAATGTATAGGATTTTTACCTTGTCAATCCT
ATAGAATGGGTATTCATTTCCGCTCATCTTCTTCAACATTGAAGAGATTTTCTGGAGATTTGT
TAGCACTATCTTTTGTGATCCTTTTCCTTCTTTAAGCTTGAGGCTGAGTGGGGAAATAACTGA
TTCAATCTCTCCACGAGTAACACTTGCAAACTCAGAGACCATACACTCTACATCCTCTGTCA
GGCAGCAGTCTGAGGATAAGGATACAAGAGTATGTACACCTCCTTCCCTGAGATGCTCTGTG
GCATTTGAAAAAGAGACCCTATGACGAGTGGACAGGGGTATCTCAGTTGACTGTGACACAG
ATCTAGTTAGCATGCTTGCAGATTTTAGAACCATGCTTGGTTTACGTGCTAGACGCAGTCGCT
TGTTAATAGTATC
>Seq23 [organism=Caladenia ophiovirus] RNA3 genomic sequence

AACTTATCGGTTCTCGGTACTAACAGCTTTATTTAAAGCCGTACACCAGAAGACTTGCAAAC
AAGTTATGCCCCATAAGCGCCCCGGACAGCTCCCAGACTCAGCCCCACAAAATCCGCCTCAG
GGTTGGTCAAAATAGGGAAAGTCAGACGGTCTTCATTGTCCTTAACTCCGTAGATGTTGATA
TCTCTCTTAAATGCATCGATCTTATCCAGATCTATCCTTGTGCGAAGTTCAGTCCTTCCAGTTT
CAGACAATGAGTACACTATTGCGCAAGTCAGTTGGAGAGTAAAGTTCTTAATCACAGGCTTC
CCTGGATGTAATGGATGCATCCTTCTCTGAGCTTCCCAGTTCGATGATATAGAGCACAGGAA
ATCCACGATTTCTAGTGCCAAGGTTATCCTGGTAGTGGCAGCAATTCTTTCTGCTAATGGAG
GGGGACCAGCAACAGTGAGATCTGGTAAAGTCTTCCTTGTCAATCTCTTAGCCAAGAGAGCA
TAGCGAATCGGTCGATTCCCTGCAATAGACATCTTGCACCGAGAAGCAACATCTGTTGGTAA
CAAATTGATATCTGAAAGGATGAACACGACAGCTGGAAATTTGTTAGTAGAGGTGCAAGAA
AGATGAGCTGCGAATTCCTGTTCAGTTAAGTTGGGCATTCCCATCATAGTCTCTTTCACAAAT
TTTGAGATACCCCTGCCACTCCCTTGACTGGATGGTAAGTAACCCTGATTGTAAACAGTTGA
GAATGCACTTTTGATTGATTTTTGGCCAATGTTGATTCTCTCAGCCACTTCTCTGTCATTACA
GGTTTCCACGTCGATATCATTTATCCTGAAAATGAAATTGTTCTTCTCCGAAGCATGGTAGGT
TGTGAATAAGATAGCATCCAGGATCTTTGTTCCCGCAGCCAGTAAAGATGAAATGCTCAATT
CCAAGTCAGCAAATCGATGGATGACAACTTCCTTTGCAAAATAAGAGTCAGCAGACTTTGGG
AGAATCTTCACATAGGCTTCCAAGGTGTTCTTTATCTCATCCACATCAAGTGCAAGATTCTTC
CCCGCAATGATACTCTCCAAATCAGCTAGAGTCAAATTTGGTGCATCATTTCGAGGGGGTAT
GTAGTTTGGGACTCCCGTATTCAGAGAAGGGGTGGAAGATGAGGATCCTGGCTGTTGATTCA
CTCGTACCAGATCATTCCTATTCACAATTGCATTAGGATTTTGTGCAACCAAAGTCCTGACTC
TAGTTGCAACTGTTTGATTTATGAGCCCATTTGGCTGAATTACTCCAAATAGGCGAAGAGTA
GAAGTGTCTTCATCTCCACGCATGTCATTGTCCATCATGGTGCACAGAGCCCTAGCTGACATT
TCAGTCGCCATCGAGTTACTTGAAGATAGCAGTTAGCAAAACGGTTTTTAAG
>Seq24 [organism=Chrysanthemum ophiovirus_indi] RNA1 genomic sequence
CAGAGCTCGTTTTTACAAAACGAAGAAAAAACCGGTCGCATTGCCGTGATTTTTGATCGATC
AAATCGTTCGATCTGTTCTTTGTTCTTAATTCACCTCTATTTTGTTTATCATTCAACAATATCA
TGTTTAGTTCTTATTACTTGTTTTTTTACATTAGCTCTAAATTCTACTAATTTCGTTTACCAAA
TTTAAAGTTTGTTGTTTGTCAACAAATATAAAACTTATTATTTAACAATTATTAATTCTGTTCC
CTAATCTGATCATCAATAGTCATCAAACTCTTCTATATAATCTTCAGGTGATACGTTTTCCTC
TATCAGATATTGATCCCCCATTTCTTTGTTTTTCCTCATGTTCTCTTCAGGGTTTCCGAATAAA
GTAGCCATCACTCCTTGCATGGCTTTTGCTTCAGTGGAATCTTCTTCTAATGTCTCCGACAAA
AATATCTGATTCTCTTCACTTTTTATACTTGAAATTAGACAAGAAAATAGTTCATTTCCCTCT
AGGGAATTAAAATAGTCTTCTATATTGTTGAATTCCTTTATGAAACTTTCTAACTCTTGAACT
TCTTTCTCAAATTTCTTGTTATCAGTTTTGCTTCCTTGCCACTGTTTTATTTCTTTTAATTTATT
GAACACGAATTTATTTCTATCATTGTGGTCTTCTGTCACTGTTATGTTGAATTCCTTATGTAA
AACCCTTATCCTGTTTATTATCAACTTGTAGTTTAAACAGTCATACCTTTTCTCTTTCTCTTTA
TTTTTCATCAGCTCCGATAAGGTCTTTTTCTTGATTCCTGAAAGGATTAAAAGTCCCTCAGGT
ACCTTTCTTTGTTTATACGAACTCACGGCCAAATTAGCAATGTTGAATATCCTATCAATCATT
TTGTATTCATTCCTCGTTTGGCTAGATTTCGTTATATTTTTCAATATCTCGTTTGATGAATAAA
GCTTATCATTCTTTAACAACTCCGATGATTCTCGTATTATCCTCCAAGAAGCATATGCAGTTT

GATCTATGTATCGTGAGTTCTCCATCAAGCTCGATAATTGCAGCACTTCTTCTTTGTTCAGCT
CTTTGAAGCCTAATATATATAAACCACTAAGATATTTGAAATCTTTATCAACTTGAATTGTCT
GAATATCTTCTGTAGTGAGGTTATTGTCTCTCATGAATTTTCTTATGTCATTCACCCTTTTTAA
GGAAGAGTTTCCTGTTTTCTCAATATATGGCCTCCATTTTATTAACCTACTCCTAATAGAATC
GATAAATGTATTTTCTACTAATATATCCTTCATAAATGTTGATTTGCTTATATTATACTCTACC
ATCAATTCAAAATCTATATTCTTCCTTTTCTTCAATTTTGACTCTAGTTTAGGAGGGATATACT
TGAAAGATGAATCCGATTCTAAAAGCATCTCCACTAAGGTATCATCACTAATAGATTCCTCA
GTATGATCAGAAACTCTAATCGGGTTTTCTGAAAATAATGATTCATTCTTTATCTTCATCATC
TCAACCATCATTTTTTCCGCTAAAATGGATTCACTAAATGATTTCTTCTCTCTTTTGTTTTTGA
ATGCATCATCATATATATCACCGTTTATCATCAGATAGACATAACCTGGTGATTGTACAGAA
GGTATCAGGATACGCATTTCTCGCCTATGTAGGATGTTTTCCCAATCGTCTATCCCTATTTTA
ATTAGACTATTTAATCTTATGATTATTTTCTTTTTCTGTGTCACAAGGTCAAATAACATCTCA
ACGAGTTTTTCTTTCTTGCCTGTGAAATGAGAAATATCCAAAAGAAACACCTTATGATCAAG
ATATTCTGAATAATTCACTTTGTCAGTCATGTCTATATTCTGTTTCACCTCTGTCATCCCTTTT
AGTCTATTTATCAAATTGTATCCATCGTTTCGTGATACAGATCTATGTTGTATATTCATAGCG
TTCATGGCAAAGTGATAATCCCCTCTTCCAGCAAAAAGATCTGCTATTGATTCCCCTTCCTTA
ATGAATCCATTGTTTTTAGCAAAAGATAGCATTGATATAGCCGATGGAAATACATCAGATTT
GGTCGGAGAAGAAACACTCGCAGGCTTACAGTTCATTGCGCATATCATCTCAAAACCCTTTA
ATGCTGAAGAAAACATTTTATGACTATTCAAGTCTTCTGCTGATATGCCAAACTCTGTGTATT
CTATGAAGTTTCGTTCCAATTTAAATCTTGATTCGTCAATATAATTTTTAACGTCGCTTTCGTA
ATAACGATTTATTTGAGATATGCTCAATTTTTTTCCATTATTATCTGCATATATATTATTCACT
GCTTCAGCAAGTTCTGTGAACTCATAACCTGATGTGGGGAAAGCCATTAATAGGAGATTGCT
TATTCTCTTATCCTTAGTGTGAAAATGAGCATCAGATGATTTGAACCTCCTAAGATTGATTAT
GGCATTCTGTCTTGTTCTATCTTTCATTATTGTTAATGTGTATTGCCCAACTTTATTCTCTTCT
AATCTGTATACTAATGATGTAGTCACTATGAAGATCTTTAAGAGAACCATTCTGTATTCTAAT
GAATAACCTCTTCTTGTTAATTCGTAAGCCCAAGTGTTTGCTCCTCCTTCCATTTCAGATCTA
AATAACTCTTGATACAATATTGATCTGACGACCGATCTAGTCGGAGTCTCATTCTCTATTATA
TTTAATCCTTTATAATATTCATAGAATTCTGAGAAATATTGTTCTCTAGTCGCATAATCATTC
ACTTCTGGGGCTATTTTCTTTAACGTCTCAGATGGAATTATATCTGGGCATATTATCTCTTGA
TCTTCCATGTATCTGTGAAGTGAGTGGAATGAACTCATTCTGTTTATTACTTCGGTAGGCACC
ACAGACTTATGATCAATGAATTTTCCTTTGATGTCCTCATCTGTTGATACGTAATATGAAAAA
CTCTTGTACAGTTTTGATTTCTTTAAGATGGAGGACAAATCTCCTTCCTTGATGCAAACCTTC
TCTGGAAGGCATTTTATACTGGAAGATGGTTTCAAGTTCTCTATCTGAAATGTAACGTCTCTT
GAAAATCTTAACACATCTTCTTGCAAGTTCAGCCCTTTTATCTCATTTTGTTCAGCTCCAATC
CCTGCTAACGTGCACTTTATCACCGTAATAATCAATGCAACATTCATATTATTATCCACACCT
CCATACCTTTCTACAACTTTGTTCACTCCCGTTGCTTCGTATTTGCTTGTTTCAAGATTTGATG
TAAGATCTCCAGTTTTAGGATTGAATCCTGCAGAAACTGCTCTGTGAAAATATCTTCCTCCTT
TTGGAGCTACTACGTGTTCTTCAATATCTCTATATTTAGTATCAGAGAATGTGCTTAGAGTGA
GATCAGCAATTCTTTCCAAAGTGCTCTTAGACTCTTCTGAAATCTTGCCAAACTTTTCACAAT
CCATTATAATCCATTTTATGTACCTAACAAGATCGTAGGCCATGAAAATCAATTTATTTTGGA

ACATTCCCTCAATCTCTAAATCCCTGTCGAACTTGGTCGTATTCACATTAGTTCTTGTTGGAC
CGCACCTCCTCATATGCTCCTGATTTTTTCCAGAGTAGACGAATCCTGATCGTTTCACTGGCA
TGATAAGATTGAAGTTCCTATCATGAGATAATTTCCCTAATATCGGTAATTCTTCTATCTTTA
TGAAATCTAGCTTGATCTCGATCTTTTTACCCCTTAGTGTATAGTGTTTTTCAGTAAGATCCCT
CCTTTCAATTAACGAACCAAAACCATATTGTTCTTTCAATCCAGTTATCGCATTCTGAAATTT
ATATTCAGGTTTGTGATTTGCTTTCCATGCTTTGTTCCACATTTTGTTTAATTTCTTCTTCCCCA
AGAGGAAGGAAAGTGTGGTCGAGTTGTCTATTTTTGAGAAAATCTCTGATATATATTGAAAC
ACAGAACACTCATAAAACTTTGATACAATTCTGGATGAAAACGAGTATTTGAACGTGTCAAG
CAATTCTACTTTGTATTGTTCTTCATTCTCTTCATTTCCAGCCAACAGTATTTCTCTCAATTCT
CTATTTTCTATCTTGTTTATCAGTTCTTTTTTTAAGAAGTCTTTCAATAACGTCTTTGTATCAA
ATTTTCCTCCTGTCATAGGAAAAGATTCTTGTATATATGCTTCTGCCCTGTCGCTGAACTTGA
CCAAGTCATATAGTTGTTTTTTCATTTTATCTGTGGTTATTAAACTCTCGACATAGCATATTCT
TTTGATGTTAGATATAGAATAACCAGAAATCATTGCGTCTACATATGATGTGCATGAACATC
CATACATAGCATACGGTAGATACATCATCATCATTATCGCGGACATTAGTTGTTCGTTGTTAT
TGTTGAATCTGAAAAAACCCAATACTATGTCATCTTCTGTTGGCAGTTGGCTCACATCGATTG
TTTCTGCAATTTCGAACTTTTTCAGACATATTTTAGCATATGAAATATAAGCATTCCTGTTTG
ATAAAAATCTACCGTCGAGTTCCACATCTAGGAAATGACTAAATCTGATTATCTCTTTATGTA
TTACTTTTAATACTCTGAAATTTCTCAATATCGTTTGTATCTTGTGTTCGTTAGATCTCAAGAC
CGATGATGTGTATCCACTATCGATTATTGTCAATTGTTCCATTTCATCCCCCCACAGTGTCGC
TGCATTAGGTGTTATTGCCATCATCCTTTTAAAGTTGTTTTCAACTGGTGTGTCTCTGTAATA
ATGACACTTTAACATAGAGCACCTTCCATTTGTCACTTGAGTTTGCGACAATTTCACAAGAA
GTCCCATTTTTAATGCGTATTGTTGTATGAACTCAATGATTTCATCCAACTTTTCCTCAGTTAA
ATCTCTTTCTATGAAGACACCACATGAGTCATCTGAATATGTTGTTCCTATGTATTTTGAAAA
ATTTAATGCTGACAACATATCTTCCATCATCAATTGAGATTGTATACCCCATAATGGACCGA
ACCAACCTTCTATGGCTCCTTTTTGATGTTTTGAGACTACGACAAAATCAGAAAATATATTTT
CAAACAATATTTCAATGTCTGAAAACAAATAAACCAAATTCGTTAATTGTGATCTTTCTAAG
GATCCATCCTCGAATCCATACATATCTGCTATTTCTTCCAAGATGAACAAATTGTTTTCTGGT
CGTTGTGACGTATTATGGCCAGAATAATCTAAAAAGATCGAATAGGAATCTTTTTCATCCAA
GATGCAGGACATTTTATGCATAGTGATCCTCCTTTCATCTTCTGTCATAGTCATCATTTGTCCT
GGAATAAGTTTCATTGCTCTTTTAACCATTTCCATAATTATTGATATGTAGATTTTGAGTTTG
AAACTTGCCACGCCATAAAATCTTGCTTCGGTTTTCAACTCTTTTTCTTTTTCTACTAGTCTAA
CTATCTTAGACTTTTTGAGGGATTTTAAAGTAATATCTTTTCTCGTTGTCAAACGAACATCTC
GTTCTTCTCTCGTTAAATCATCAATTTTCACATCAGCTACCAGTTCACTTAGGTTGTTTTGCGT
GATGTAATATATCAGTTCTTTTACAGAATTTCCTGAGAAATCATATTCATCTTTCGTGCATGC
CTTGTCTATTACTCTGGAATTTATGTTGACCTCGTTTCCTACTTCCAACATTGCTCCTAATCTT
AAAAATCTGTATTGGACTGGATCATTTATTATGTGAGTGTTGTAATTCCCACCAGCTGCCATC
AACGTGAGCTCTTGTTCCAGGATAGCAGGTGCATTCATCAGTTTTGGCACTGATCCAAACTT
CTTTATGTAATTTGTCACAACTTTCATTCTCATCAATTGTTTTAATCTTAACACAGATTCTGCT
TTCACTTCATGATTCCTGTTCGTTCGTTGAACATATTTATCTAATCCTTTTGTTTCTGAGACTT
CTGCATACACTAATACTTTTCCAAGACAAGACATCTTGATACATGTCTCAGCATCAGAAATA

TCGCATATGTCAAACAATCTCATAGTTAAAGATGCATCCTCATTTATTGAATCAACGATGTTG
TCTATTATCGGTAGTATCGCGGAGCTTGATTTCAAGTCCGACAACAATAAACATGTTGCTTC
GAAGAGTGCTGCCATGTTCACTCTTTTCGGATTGTTCTCTTTGACAAGGTCTTTGACGAATGT
GATAAACAATGCTTCTAGGTCTCTTTTTGCTGAATCATTTAATGCTTTTATGAGATTTAATGT
CAATGAATGGTCACACAATGTCATCAGGTAATTGGTCATGTTTCCATCACCAATGATCAACA
AATTGTTTTTTTCGTCTTCAATTGCAGTTATCGTTTTAGAGAGAAAAATATTAATAATTCTCT
CTTTCGGTGTTGATGGAAATATAGTTACTTTTGTAAAGCTTGTTCCATCTGAGTTGACCTTTG
TTTCTATTCTTCTTTGTTCGTGTTCTAGAGATAATTGAGAGTAATTCTTCTCTGAAGACATCAC
AGAATAGTTTATCTTTATCTTGTGATTGATTGTCTCCAACAAATTAACCAACCACATGAGGGT
CTTTGTTTCAGAGTCAAGTTCCATGTGCAAATCCTTCCTCTTCATAATCTTCTTTTTGTTAAGG
AAGTTTTCTGAATTGCGGTTTGTCTCAAACTCCAATAGTTTGATCCCAAGATCAAATAAAAG
GTCTGATCTTTCAGACTCTATCTTCATGTTTGACATATCATTAAATAGATTTACAACAGCATC
TTGCGAAGAGACAGGTTCTATTTTTCTTCCTTCATTGCTTATCTTCTTCACAAATTCTCTAAAT
CTTAACACATCGATCCTAGTTCTTGTGCCAAACTTCTCTGGGTACTTGTCACTTTTCATGAAA
AATTCAACAACATTTTCTGATAATGATCTATATGGTGAGTCAATTTTATTAATCAACACTGGC
TTGTCTATATAAGTATATGATATATATTCTCTGTCCCAGTGCTCAGTGTACAAAAACATCTGT
TCATAATCTATTTTGCCTTTTTCCGGTTCCATCTTTTTTTCTTTTTTTTGAATTTTGTTTAGTTTT
ATAAATTGCTCTTTGTATTTGTACATCAATGATGAGAGTTTCTGATTTTTTTCTATTTTTTTCA
TCATTTCATCGTGTTTCTTCAGGATTTCTTCTTCTTCTAGCAAATCGTTTAGATCTCCTTCGCA
TTCTTCAGACTCAGCGATAGTGAAAATACTCATTCTCTTCTCATTGTTAGGATTCACATATGT
TTCTGGTGTGTTCTCGTGTGATGATTCTACACTGGAATAAATAGTCTCTAGATCTGATTTATC
ACTTGACCACTCCATCTCAAAGATTTGTTTTCAATAATGCTTATAATTACTCTCTTTCTTTTT
ATTTAAAGCGAGGATTTCAGCAAAACATTCTTAATCGTCTTATCTGTTATTAGATTAAAACA
ATTTGTGTCTCTTGCTCAAATAGTTCGTGATCTGTGATCGTTTGCAGTGTTGCTTATCGATCTC
ATCATAAACACAGTTTTTGCAACCCATTGCTTAGTCCTGCTGCACTTTTCTTCTATGGCTTTGT
TCTTTGAGAACATTTCTTCCATCCTATCAACTCTTTTCATTTCTGTCTTACTTTTCAATAGCCA
AAGATTGTTTAATTTCTTCCATTTTTTACAAAGGAATTCTTTTTCTGATGTCGAAAGCATGGG
ATAAATGTCATTCTCTTGTGGCACCTCAGTGACAAATACATCAAATCCTTCCCCATCATAGA
ATATCATATCGGATAACAGATTCACAAATTCTGTTGATTGAAATAAGTTTGCTTTATATTTCA
CCAATTGTAAATCAATTCCATCTTTGATCAATGCAGTTTCAAAGTTAACATTCTTGTGCAATT
TTATTGTTCCTTTTATCATTGCCATGAATTCGTCCTCATTTTTTGATTCTCTTATCATCCAGCA
ACCATGTAACTTACGCCACTTTTCATCAAACGAATTTTCTTCTATTTTGAGAAATTTTCTGTA
AATTTCATCTAAGTCTTCTCTTACCTTAAATCCCATCTTGTTCATCTCCTCTTTTGATACTCTC
ATTTCGTAAAATTGAGCTTCTCCAGTTGATTCCAATAGATCAAATTTCATTTTTGTTTTTATGA
AATCTCAATTATTGGAACCTGTAAG
>Seq25 [organism=Chrysanthemum ophiovirus_indi] RNA2 genomic sequence
CGATCCTGTATATGACAGGGTTTCCTCATTAAGAGATTAAAGATCGCATTGATCCGATTTTGT
GTTGTTTAACATTCATCTTAAGTATCACCGCTTCCGTTCAACAATTGCTTTTCCCTTTGCTTCA
TGCTTCGGTTCGAACCTCTGCTTCTAGTAGCATTGGCCTTCCACAATCCGCGTCGTCGGTACT
AATCCCTCATGTCTCACCAAAGCGGGTACTGGAATAAAATCTACAGGTGACTTTTTTGAATT

GACATCGCTTTTCTTGATTCCAATTACCCTGATCCTATTTTTCCTCTTAGTTGCTTGAACAAAG
CCACCTGTTGATTTTATTCAAGAGTTTTATTTAAAACGATAACTACTAATTAGGAGCAATACA
AAGAAGATGTTTTATAAAATCTTCTTTCCTGATTTCGTTCTTTGATTCAGTACTGATCAAAGA
ATGTCAATCGTTTTAATTAAAATACAACACACAATATTTAATATTACACACTGGCACAAAAC
ACTAATGAGCATCGACTTATGGAACAATTGAAACATTACATTATACAAAGCCATTGATTTCA
ATCATGGTCTTTAATGTTTGTTTTATCACTTATATTAGCTTCACTCTAGACCGCATATCATAGT
TGAAATGATACGCATCATGGCCTTCTATTTAAATTTCATTGTCATCATTTCAGAGTATGCATT
AGCAGACATTCTCTTATTGTCAGTGACTTCCTTCCATACACTGTCCTTCAACCACATCCTCCC
TTCCTCACTTGTATCGAATACCATCACTATTTCACCCTTTAGCTTTAGAAACAAAGAATTTCC
TGCTGTTGTCATCTCATCAACATGTCCGTCGTTCCTTAACATTGAGTAAGACAGAATGTTTAA
TCCCAACCTTCTGTCAGTGAGCATGTATACTTCCTTTAAGACCATATCTTTGGTCATGGTCCT
AAGCAAACATTCAGCTTTCTCAACTTCAATCTCCTCAACTCCCCCGATATGCTTGATCACGTC
AGTTTTATCGATCCTCTCACAGTGTATGAAATGATCTGCTGAACCAGTATCAAGAGTAGTCA
AATAATCTTCCCCATTTGGTAACATGTACCTGTGTGGTGCAAGAGGTTCGATGATAGGGGGA
GGAGGTGCATCCCTTTGGCTAGCAAGATAATTAGGCAACACACTTATGCTTTTCCTGGCAGC
ATCCATAAAATCCCTAGCTCCTTCAGAGCTGCTTGAGCTTTTACTTTCAACGTCGAAGTCTAA
GTATTTTTTGCTCTTCTTTTTCTCTTTTTCATTTTCTCTAAGTTTCTTCTTATAAGCTTCCTTTTT
TGTTTCAAGCTCTTTTGTCACTTTATTGGCCAATGAATTAAAGACAGATTTATTATTGATGTC
TTTGGGCCTTGAAAAGGAATCAATGTAGATGACTGTTGAAGGCTTTTGGTCAATCTTTGTTG
GGATTGTCTCACTGTGAGCATAAAATGCCACGTTTAGAGATCCATACTTCCTTCCAGAAATA
GGAACACCTTCTGTCTCAATATGAACAGTGATTTTTTCCACATCTTTTTCATAAACAAAGAAG
TCCATTGATAACTCCACCATGGCCATCTCAGATGCATTGATTGTCACTTGTTGTATGACCTCA
TCATCTGTTGCTGCTGTATCATGCAGTCCTAGTGTTATTGTCCCATTGTCCTCTTCTGTCTCTC
TCTGACTAAATAGTGGAACATAAAAGAACTGGACTTTATCAAACCTAATGAACTTTTCATTC
GATATGACTCCTTTTGTCATTGAAGTCCAAATCTGTTTCATGGTTGTCATCCTAACCCTCTTTG
TGTGTTCATCTTTTGTCAGATTGAGCACCATGGGAATTATTCCAGTTTTGATTGTTCCTTTAG
ATATGTTCACAAAATCTGATGTTATATCTTCGATTCCATCAGGTATTCCTCCCTCTTGATCTC
GCAGAGAGATGACATTAACTTCACCCTTCTTCACTATCTTATCTGCCACAGAAACTGCCTTCA
TCCTGCTTGTGCTGCTTCCTGAGTCCCTTACAGATCTTGTTGATTCAATTCTCCTTGGACCTCC
ATAGGAATATTTCGAAACTGAAGACATCTTTAATCTAGATTTGTCGTATTACGTCAATGCAA
AC
>Seq26 [organism=Chrysanthemum ophiovirus_indi] RNA3 genomic sequence
GCAGTATTTTCAAATTGATTTATTTAAAACGATGAAGGTGCACACCTGTGACCCTGTCAAGT
TGCGAACCAACAGCAACTCATCAGTGTCATAACATCATCGTTATCATTACAATGACACTATA
TGAAATTATATTATTATTCAAACTTAATTCATCTTATATGCTGCCTTCAAACTCTCTGCTGTG
ATGTCAGAGAAGTCAGCATCAGGGTTGGATAAGATAGGCCATGTGTGTGAATCACCGTCTTT
AATTCCGAAGAAATTCACATCCTTTTGAAATGCGGGATTGTTATCAGCCACTATCTTTTGAGC
CATGGCTATCAATCCACTTGCACTCAGCGAGAAGATTATCGCATGAGTCAGCTTCAAAGTGA
AGTTCTTGATGGTGGGCCTTTCAGATGAGAGTGGATGCATGACAAGCTGGGAAGTGACATTT
GTTGCGACTTGTTTCAAACAGTTCACTATGTCTTTTGACAGCTTCAGTTTAGCATTGAACTTC

ATGATGTCCTCTATCTGAGTCACAGGAGTTCCTTCAGTGATTTTCAAATCTGACTTTGTGCTG
AAAGGACTGGCATATTTGGCATACCTCACTGCCCTGTTTCCAGCGATATTCAGTTTGCATCTT
GACGAAACAGCTGGAGGAAGGATATCAAGCTGTATCTGCAAAAACACCTTAGCAGGAAATT
TCTTTGTGTTTGCACAAGATAACTGTTCTCCTATACTCAGCATGGTCACGCTCTCCCCAGAAT
AGATTTCTTTCGTGATGAACTGAGGAACAGGGGTTTCAGCAGAATTCTTTCCTTTATTCTCCG
GCAAACCTCCCTGTATGTACACAAGCACGGCAGCTGCCTGAATAGCTCTTTTCGCATTGCTC
ATATTCACTGCAATTGTCCTCTCAGATATTCCTGATGTTTCAACCAGTTTAAACATTCCTGTG
TTGTCTGGTGATTGTGCCATTGCGCAGTACAAGATAGCATCGAGTACTTTTGTTCCTGCAGCA
ATGAGCATGTTGACATCAGCACCTCTTTCTCCAATATATTTGATGACAACTTCTCCAGAAACA
TATCCCTCATCTCCAAGAGTGACACTTTTAAGGAAGTTTTCAAGATGTTCTTTGATTTTGGAG
CTATCAATCGAGTAGGACTCAGACTTGATTTTTGCCAAGATGGTCGCTTTATCCTTAAGTAGT
GTATCGGACAAATCTTCGAACCGATTTTCATTATTCTTTTCATTATCATTCTTCTTCTTGTTTT
GATCACCTTGTTGATCCTTATGTTGATCTTCATTCTTGTTATCAGCTACAGGTACAATCTTATC
ACCTTCAAGCATGACCAATTTGTCAGGGTTCATTGACTCCTTTGCAATCGTCACTACATTGTT
TGTGACAAGTGATCCATTATCATGAAGCAATTTCCATAGTTTCAATGTGTTCATTTGTTCTCC
AGTCAATTTGTCATTTCCCAAAGTTTGTTGAATCTCAATGAGAGTCTGTAGGGATTTTGCTAA
GATGAATGTCGACATTATTTAGTGTACAATTGAGAAGCTAAAAAATCTGGAATAAGCAAGTT
TTTT
>Seq27 [organism=Chrysanthemum ophiovirus_mori] RNA1 genomic sequence
GTCGCATTGCCGTGATTTTTTGATCAGTCGAGTTGCCTGATCTGTTCTTTTTGTTTCCGATGAA
AATAATTTTAACATTAAATTTGCATGATAATTTCGAACCATTGTTTATAGATATATATATGTC
TTCATCTTCAAATTTTCTAGACTTATTCTTTGTGGTTACTCCCTACTTATGATGACTTAAGATT
GTTAAATATGTTAAGGGATGTTATGTCATATTCTTACAAAAACAAAATAATTATTTCAGTATT
CATCAAACTCTTCTATATAATCTTCAGGGGAAACATTTTCTTCAATCAAGTATTGTTCTCCCA
TTTCTTTGTTTCTTCTTATATTTTCTTCAGGATCTCCAAACAATGTAGCCATCACCCCTTGCAT
GGCTTTTGCTTCATCAGAATTTTCCTCTAATGTTTCCGATAGGAAAACTTGGTTCTCTTCACTT
TTTATGCTAGAAATCAAGCATGAGAATAATTCATTGCCTTCTAGAGAATCAAAATAATCTTC
AATATTATTGAACTCTTTGATGAAACTTTCCAATTCCTGAACTTCTTTTTCGAACTTTTTGTTA
TCTGTCTTGTCACCTTGCCATTGTTTTATTTCTTTGAGTTTATTGAAGACAAACCTGTTCTTGT
CATTGTGGTCCTCTGTCACAGTAATGTTGAACTCTTTATGTAATACTCTTATTCTATTTATTAT
TAACTTGTAGTTTAAGCAATCGTATCTTTTTTCCTTTTCTTTGTTTTTCATTAATTCTGATAAT
GTCTTTTTCTTGATACCTGAAAGTATCAACAGTCCTTCAGGGACCTTCCTTTGTTTGTATGAA
CTTACAGCCAAATTAGCTATGTTAAATATCCTATCTATCATCTTGTACTCATTCCGGGTTTGA
CTGGACTTCGTTATGTTTTTCAATATCTCATTGGATGAGTACAGTTTGTCTTCCTTCAACAATT
CTGATGATTCACGTATGATCCTCCAAGAAGCGTATGCAGTCTGATCAATATATCTAGAATTC
TCCATCAGGCTGGATAAGTGCATCACCTCTTCCTTGTTCAATTCTTTAAAACCGAGTATGTAA
ACGCCACTTAGGTACTTGAAATCCCTGTCTACCTGTATTGCCTGGATATCATCAGTTGTTAGA
TTATTGTCTCTCATGAATTTCCTTATATCATTAACTCTCTTCAAATTTGAACTTCCTGTCTTCT
CGATGTAAGGTCTCCATTTAATCAATTTGCTTCTGATAGATTCAATAAATGTGTTTTCAACTA
GTAAGTCCTTCATATAAGTTGATTTGCTTACATTGTATTCCACCATTAATTCGAAATCTATGT

TTTTTCGTTTTTTGAGTTTTGATTCCAGCTTGGGAGGGATATATTTGAAAGATGAGTCTGATT
CCAGGAGCATTTCTACTAGGGTATCATCACTAATCGACTCTTCAGTGTGATCTGATACTCTCA
AAGGATTCTCAGAAAATAATGATTCATTTCTTATTCTCATCATTTCTGTCATCATCTTTTCCGC
TAATATTGATTCACTAAATGATTTTTTCTCTCTTCTGTTCTTGAACGCTTCATCATATATATCT
CCATTTATCATTAAGTATACATAACCAGGTGATTGAACTGAAGGTATTAAAATCCGCATCTC
ACGCCTGTGCAATATATTTTCCCAATCATCTATTCCTATTTTGATTAAACTATTTAACCTGATT
ATTATTTTCTTTTTTTGTGTTACAAGGTCAAACAACATCTCTACTAGTTTTTCTTTTTTCCCTGT
AAAGTGAGATATGTCTAAGAGAAATATTTTACTATCCAAATACTCTGAATAATTACTTTTATC
AGTCATGTCTATATTTTGTTTCACTTCTGTCATCCCTTTCAATCTGTTTATTAAATTATAACCG
TCGTTTCTGGAAACAGACCTGTGTTCAATATTCATTGCATTCATAGCAAAATGATAGTCTCCG
CGCCCTGCAAAGAGATCTGCCACTGAGTCTCCTTCCCTTATGAAACCGTTGTTTTTTGCAAAT
GATAACATAGAAATTGCTGAAGGGAACACATCAGATTTTGTCGGAGAAGAAACATTTGCGG
GCTTACAGTTCATGGCGCAAATCATTTCGAATCCTTTCAATGCTGAACTGAACATCTTGTGGC
TATTTAGATCTTCTGCGGAAATCCCAAATTCCGTATATTCAATGAAGTTATGTTCTAATTTGA
ATCTTGATTCATCGATATAATTTTTTACATCACTTTCATAATATCTGTTAATTTGTGATATGCT
TAGTTTCTTTCCATTGTTATCCTTATATATGATATTGACTGCTTCAGCAAGTTCTGCGAATTCA
TAACCTGATGTCGGGAATGCCATTAACAAAAGATTACTTATCCTCTTATCTTTTGTGTGGAAG
TGTGCATCAGATGATTTGAATCTTCTCAAGTTGATTATTGCGTTTTGCCTTGTTCGATCTTTCA
TAATTATCAGAGTGTATTGACCTATCTTGTTTTCTTCGAGTCTATACACTAATGAAGTTGTGA
CAATAAAAATCTTCAACAAAACCATTCTATATTCTAAGGAGTAACCTCTTCGTGTCAGCTCA
TAGGCCCATGTATTGGCTCCCCCTTCCATTTCAGATCTGAATAGTTCTTGGTATAGTATTGAC
CTCACAACGGATCTGGTTGGTGTTTCGTTCTCGATTATATTTAATCCTTTATAATATTCATAG
AACTCTGAAAAATATTGCTCTCTCGTTGCATAATCATTTATCTCTGGTGCTATTTTCTTCAGC
GTTTCTAACGGTATTATGTCAGGACATATTATTTCTTGGTCTTCCATGTATCTGTGTAACGAA
TGAAATGAACTCATTCTGTTAATCACATCAGTGGGCACCACTGATTTATGATCAATAAATTTC
CCTTTAATATCTTCATCTGTAGACACATAATATGAGAAACTTTTGTACAATTTTGACTTCTTT
AGAATGGATGATAGGTCACCTTCTTTAATGCAGATTTTTTCTGGGAGACACCTGATACTTGAT
GAGGGTTTCAGGTTTTCCAATTTGAATGTCACATCTTTAGAAAATCTCAATACATCCTCTTCT
AGATTTAATCCTTTTATTTCACTTTGCTCAGCTCCTATTCCTGCTAAAGTACACTTAATCACTG
TGATTATCAAGGCGACATTCATGTTGTTGTCCACTCCACCATATCTTTCCACAACTTTGTTGA
CACCTGTTGCTTCGTATTTGCTTGTCTCCAAATTAGAGGTGAGATCACCAGTTTTTGGGTTGA
AACCTGCTGATACTGCTCTATGAAAATACCTCCCTCCGTTTGGTGCGACAACATGTTCTTCTA
TGCTTCTGTATGTGGTGTCAGAGAATGTGCTTAATGTTAGATCTGAAATTCTTTCCAGAGTGT
TCCTTGATTCTAATGATATGTTCCCAAACTTTTCACAATCCATAATTATCCATTTTACATATCT
CACAAGATCATACGCCATAAAGATCAATTTATTTTGGAACATTCCTTCTATCTCCAAATCTCT
ATCAAATTTTGTGGTGTTTACATTCGTCCTGGTAGGCCCACACCTTCTCATATGTTCTTGGTTT
TTTCCTGAGTAAACAAACCCTGATCTTTTCACTGGCATTATTAGGTTGAAATTTCTATCATGT
GAAAGTTTTCCTAATATAGGTAATTCTTCTATTTTTATAAAATCTAATTTTATTTCTATCTTCT
TCCCTCTCAGTGTGTAATGTTTTTCAGTATTGTCTCTTCTTTCTATCAAGGATCCAAAACCGTA
TTGTTCTTTTAGTCCTGTCAAGGTATTCTGGAATTTGTACTCAGGCTTATGGTTTGCCTTCCAA

GCTTTGTTCCACATCTTATTCAATTTCTTTTTCCCAACAAGAACGATAAAGTAGTTGAATTG
TCGATTTTTGAGAAAATCTCTGAAATGTATTGGAACACAGAGCACTCATAAAATTTAGACAC
AATTCTAGAAGAAAAAGCATATTTAAAAGTGTCAAGTAATTCTACTTTATACTGTTCTTCATT
CTCTTCATTGCCTGCTAACAATATCTCTCTCAATTCCTTGTTTTCTATTTTGTTGATCAATTCTT
TTTTCAAGAAGTCTTTCAATAATGTTTTTGTATCAAATTTACCACCTGTCATTGGGAAAGGTT
CTTGTATATAAGCTTCTGCTCTATCACTAAACTTAACTAAGTCATACAACTGTTTTTTCATTTT
TTCCGTTATCAATAAACTTTCGACATAACAGATTCTTTTCACATTTGATATTGAGTATCCTGA
AATCATAGCATCGATGTAAGAAGTACAAGAACATCCATACATAGAATATGGTAAATACATC
ATCATCATTATCGCCGACAACAATTGTTCATTATTGTTATTGAATCTAAAGAATCCCAAAAC
AATATCGTCTTCTGTTGGAAGTTGACTCAAGTCTATTTTTTCTGACACTTCAAATTTTTTAAG
ACAGATTCTTGCATATGAGATATAAGCATTCTTGTTGGACAAGAACCGACCGTCCAATTCAA
CATCTAGAAAATGACTGAATCTAATTACTTCTTTGTGTATCACTTTCAACACTCTAAAATTTC
TTAGTATCGTTTGTATCTTATGCTCATTGGATCTCAAGACTGATGATGTGTAACCACTATCAA
TGATTGTCAATTGTTCTAGTTCGTCTCCCCATAACGTTGCTGCATTTGGTGTAATTGCCATCA
TTCTCTTGAAGTTATTTTCTACTGGTGTATCTCTATAATAATGGCACTTCAACATAGAACATC
GCCCATTAGTAACTTGAGTTTGGGATAATTTGACCAATAGACCCATTTTCAAGGCATATTGTT
GTATGAATTCAATGATTTCATCTAATTTCTCCTCTGTCAAATCTTTTTCTATAAAAACACCAC
ATGAATCGTCTGAATAAGTTGTTCCTATGTATTTTGAAAAATTCAAAGCTGATAACATGTCCT
CCATCATCAATTGTGACTGTATCCCCCATAATGGACCAAACCAACCTTCAATAGCCCCTTTCT
GGTGTTTAGACACAACTACGAAATCAGAGAAAATATTTTCAAACAATATCTCAATATCAGAA
AATAAATAAACCAAATTGGTCAGTTGCGACCTCTCGATTGAACCGTCTTCAAAACCATACAT
GTCTGCTATCTCTTCTAAAATAAAAAGGTTATTTTCAGGTCTTTGTGAAGTATTGTGACCAGA
ATAATCCAAAAATATTGAATAAGAATCTTTTTCGTCCAGGATACATGACATCTTATGCATAG
TGATTCTCCTTTCATCTTCTGTCATCGTCATCATTTGGCCTGGAATGAGTTTCATAGCTCTTTT
GACCATCTCCATTATGATAGAAATGTAAATTTTCAATTTGAAACTTGCAACTCCGTAGAATCT
TGCTTCTGTTTTTAATTCTTTTTCTTTCTCGACAAGCCGAACTATTTTAGATTTTTTAAGAGAT
TTCAAAGTTATATCTTTTCTTGTCGTCAACCTCACATCTCTTTTTTCTTTTGTTAGATCATCAA
TTTTCACATCTGAAACTAACTCACTTAAATTGTTCTGCGTTATATAGTAGATGAGTTCTTTAA
CGGAATTCCCAGAGAAATCATATTCGTCCTTTGTGCATGCTTTATCAATAACCCTGGAATTTA
TGTTAACTTCATTCCCTACTTCAAGCATCGCTCCTAATCTCAAAAATCTATATTGAACTGGGT
CATTTATTATCTGGTTGTTATAATTTCCTCCTGCTGCCATGAGTGTCAATTCTTGTTCTAATAC
CGCCGGAGCATTGGTCAACTTCGGTACAGACCCGAATTTCTTCACATAATTTGTCACAACCTT
CATTCTCATCAATTGTTGTAATCTCAGTACAGATTCCTTCTTAACTTCATGATTCCTGTTCGTT
CTTTGTATATATTTGTCTAATCCTTTTGTTTCAGATACCTCAGCATACACCAACACTTTTCCTA
AACAAGACATCTTTATGCATGTTTCTGCATCAGATATATCACATATGTCAAATAATCTCATTG
TTAATTCAGCATCTTCATTTATTGAATCAACAATGTTGTCAATAATTGGTAGGATAGCTGAAC
TCGATTTCAAGTCTGATAGCAATAAACAAGTTGATTCGAATAGCGAAGCCATATTTACTCTT
TTTGGGTTATCTTCCTTAACTAGATCTTTGACAAATGATATGAACAATGCTTCTAAATCTCTT
TTGGTAGAATCATTCAATACTTTGATCAGATTCAAAGTCAATGAATGATCGCATAACGTCAT
AAAATAATTCGTCATGTTTCCATCACCTAGTATCAGTGATCCATTCTTCTCATCATCTATTGC

TGTAATTGTCTTTGATAGAAATATCCTTATGATTCTTTCCTTCGTCGTTGACGGAAAGATAGT
CACCTTTGTGAGACTTGTTCCATCAGAATTGACTTTTGTTTCAATTTTCCTCTGTTCATGCTCT
AATGACAATTGTGAGTAACTTTTTCTGAAGACATGACAGAGTAATTAATCTTGATTTTGTGA
TTTATTGTCTCAAGTAAATTGACTAACCACATGAGAGTTTTTGTCTCTGAATCGAGTTCCATA
TGCAAGTTCTTTTTCTTCATTATCTTCTTTTTATTTAAGAAGTTTTCAGCATTCCTGTTTGTTTC
ATAATCTAACAGTTTCACACCCAAATCGAATAACAGGTCGGATCTTTCTGATTCGATTTTTAT
GTCCGACATTTCATTGAATAATCCTACAATCGCGTCCTGTGATGAGACGGGCTTGATTGATCT
CCCTTCTTTTGTTATGTTTTTAACAAACTCTCTAAACCTCAATACATCAATCCTTGTTCGTGTC
CCGAACTTCTCTGGGTACTTGCTACCATACATGAAGAAGTCCACAACATTTTCTGACAATGA
CCTGTATGGCGAATCTATTTTATTTATTAATACTGGTTTATCTATATATGTGTACGATATGTA
CTCTCTATCCCATGTCTCTATATATAAGAACATTTGTTCATAATCTATCTTTCCTTTCTCTGGT
TCCATCTTTTTTTCCTTTTTTTGTATTTTGTTTAATTTAATATATTGCTCTTTATATTTGTGCAT
AAGAGCTGTAAGCTTCTGGTTCTTTTCTATTTTCTTCATCATCTCGTCATGTTTCTTTAATATT
TCTTCCTCTTCCATAAGGTCTTCCAAATCTTCAACGAACTCTTCAGACTCAGTGATAGTGAAT
ATGTTCATCCTCGTTGCACTGTTAGGGTTCACGTATGTTTCTGGTGTATTTTCATGTGATGAA
TCAACACTAGAATATATTGTCTCCAAATCTGACTTTTCACTTGACCACTCCATTGAAAAAATT
GCTTTTGTGCTTGTTAATTAATAGCAGTTGTTATTTCTCACCATGTTTTTTATTTAAAACGACA
ACAAATGTGATTTAAAACCCCCATTATTTCTTTTTCAATGTTGACTTTATTATTTTGATGATCT
CCTCATATGGTCCTTGATCTATTGTCTTTTGCTACATTATTCATTGATCTCATCATGAAGACA
GTCTTTGCAACCCATTGTTTAGTTCTACTTGTCTTTTCAACAATGGTTTCATTATTTGAAAAGA
TTTCCTCCAATCGATCTGCTCTTCTGATTTCAGCCATGTTTTTTGATAACCAAAGATTATTAA
ACTTTTTCCATTTTCTACAAAGGAATTCTTTTTCTGATCTAGAGAGCATTGGGTAAATGTCAT
TCTCTTGTGGAACTTCTGTGACAAAGACGTCGAATCCCTCCCCGTCATAGAATATCATATCA
GATAATAAGTTTATAAATTCCGTCGAGCTAAACAGACTCGCTTTGAATTTAACCAATTGTAA
GTCTATTCCGTCTTTAATCAATGCCGTTTCGAAGTTGACGTTTTTGTGTAGTTTCACGGTTCCT
TTTATCATTGCCATGAATTCATCTTCATTTTTTGACTCTCTTATCATCCAACAACCATGCAATT
TCCTCCATTTTTCATTGAATGAATTCTCTTCTATTTTAAGGAATTTCTCGTGAATCACATCCAG
GTCTTCCCTGATCTTGAATCCCATATTACTCATTTCTTCTTTCGACACTCTCATTTCATGAAAT
TGAGCTTCACCATTGGATTCCAATAGATCGAATTTCATCTTTGCTTTTGTGAAACTGAAACTA
TTGGAACCTGTAAGCTTTTTCAAAAAATGAGAGTG
>Seq28 [organism=Chrysanthemum ophiovirus_mori] RNA2 genomic sequence
CGATCCTGTATATGACAGGGTTTCCTCATTAAGAGATTAAAGATCGCATTGATCCGAGTTTG
TGTTATTTAATAACCATTCTAAGTATCACCGCTTCCGCTCAACAATTGCTTTTCCCTTCGCTTC
ATGCTTCAGTTCGAACCTCTGCTTCAAGTAGCATTGGCCTTCCACAATCCACTTCGTCGGTAC
TAATCCCTCATGTCTTGCTTAAACGGGTACTGGAATAAAACCTACAGGTTCAATTTGAAAAT
AAACATCGCTTTTCTTGATTCCAATGATTTATCCATCTCTCTACTAATTAGTTGCTTGAACAA
AGTCACCTGTTGGTTTTATTCACAAGAGTTTTATTTAAAACGACAGTTACAAGTCATGAATA
ACTCAAAGAAGATGTTTTATAAGACCTTCAGTTACGACTTGCTTATTTAGCTTAAATTGACTA
GGGACATAAGTCGTTTTAATTAAACTACAACACACAATATTTAGTATTACACACTGACACAA
AATACTAATGAGCATCGACTTATGGAACAATTGGAAAATCAACTAGCATGGGACCACTAAC

TTAGATGGTGATCTTTAATGTTTGTTTTATCGGTTATTTAGCTTCACTTTGGGCCACATGTCA
TAGTTGAAATGACACGTTTCATGACCCCTTACTTGAATTTCATTGTCATCATATCAGAGTATG
CATTCGCTGACATCCTCTTATTGTCAGTGACTTCCTTCCATACACTATCCTTCAACCACATTCT
ACCCTCTTCACTTGTGTCAAATACCATCACAATTTCACCTTTCAACTTCAGAAACAAAGAGTT
TCCAGCTGTTGTCATCTCATCCACATGCCCGTCATTCCTTAACATTGAATAAGACAATATGTT
CAATCCCAATCTCTTGTCTGTAAGCATGTAAACTTCCTTCAGCACCATATCTTTTGTCATAGT
TCTGAGCAGACATTCAGCTTTCTCAACTTCAATCTCTTCAACCCCCCCAATATGTTTGATCAC
ATCAGTCTTATCAATCTTTTCACAATGTATGAAATGATCAGCAGAGCCAGTGTCAAGTGTTG
TCAAATATTCTTCTCCATTCGGCAACATGTACCTGTGTGTCAACAGTGGTTCAATGATGGGA
GGAGGAGGTGCATCTCTTTGACTTGCAAGATAATTAGGCAGGACACTTATGCTTTTTCTTGC
AGCATCCATGAAATCTCTAACTCCTTCAGAACTATCTGAACTTTTACTCTCAACATCAAAGTC
CAAGTATTTTTTGTTCTTCTTCTTCTCTTTCTCATTTTCTCTGAGTTTTTTCTTATAAGCCTCTT
TTTTTGTTTCTAGCTCTTTTGTAACCTTATTAGCTAATGAATTGAAAACAGACTTATTGTTGAT
GTCCTTAGGCCTTGAAAAAGAGTCAATATAAATGACTGTTGAAGGTTTCTGATCAATTTTTGT
CGGTATTGTCTCACTATGTGCATAAAAAGCCACATTTAGAGACCCATACTTTCTTCCTGATAT
TGGAACACCTTCTGTTTCAATGTGAACTGTAATTTTCTCCACATCCTTTTCATAAACGAAGAA
GTCCATTGACAATTCAACCATCGCCATTTCAGATGCATTGATTGTCACTTGTTGTATGACCTC
ATCATCTGTTGCTGCTGTGTCATGTAGTCCTAGTGTTATAGTTCCATTGTCTTCTTCTGTTTCT
CTCTGGCTGAATAGTGGAACGTAAAAAAATTGGACTTTGTCAAACCTAATGAATTTCTCATT
AGATATGACTCCTTTTGTCATTGAAGTCCAGATCTGTTTCATCGTTGTCATCCTCACTCTTTTT
GTGTGTTCGTCTTTTGTCAGATTTAGCACCATTGGAATAATTCCAGTCTTTATCGTTCCTTTCG
ATATATTGACGAAATCTGATGTTATGTCCTCTATTCCGTCAGGGATTCCTCCTTCTTGATCTCT
CAGAGAGATGATGTTAACTTCGCCTTTCTTCACTATTTTGTCTGCCACAGACACAGCTTTCAT
CCTGCTTGTACTACTTCCAGATTCCCTTACAGACCTTGCTGATTCAATCCTCCTCGGACCTCC
ATACGAGTATTTTGATACTGAAGACATTTTCGCTTTAGACGATTACCTCTTGTACAACTGCAA
ACTTTGATCCTTTAGATCGGAA
>Seq29 [organism=Chrysanthemum ophiovirus_mori] RNA3 genomic sequence
GCAGTATTTTCACATTGATTTATTTAAAACGATAAAAGTGCACACCTGTGACCCTGTAAAGT
TGACCACCAGCAGCAACTCGTCAAGATCACAACATCACTCTAATTTTTATAGAAATACAAGA
TGAAATTATATTATCATTCCAAATTAATTCATCTTATATGCAGCCTTCAAACTCTCTGCAGTA
ATGTCAGAGAAATCAGCATCAGGGTTGGACAAGATAGGCCATGTGTGTGAGTCACCATCCTT
TATTCCAAAGAAATTGACGTCCTTCTGGAACGCAGGATTGTTATCAGCTACTATCTTCTGAG
ACATGGCTATCAGCCCACTTGTGCTCAGCGAGAAAATTATCGCATGAGTAAGCTTCAAAGTG
AAGTTTTTGATGGTTGGTCTCTCAGATGAGAGTGGATGCATCACAAGTTGAGAAGTTACGTT
CGTCGCCACTTGTTTAAGACAGTTAACGATGTCTTTTGACAACTTCAGTTTTGAATTAAACTT
CATTATATCCTCAATCTGAGTAACAGGAGTGCCTTCAGTGATCTTCAACTCTGATTTGGTGCT
GAAGGGGCTAGCATACTTTGCATATCTCACAGCTCTGTTCCCTGCAATATTCAACTTACACCT
GGATGAAACAGCAGGAGGAAGAATATCAAGTTGGATCTGTAGAAACACCTTCGCAGGAAAT
TTCTTTGTGTTAGCACAAGAAAGTTGTTCTCCTATGCTCAGCATGGTCACAGTCTCTCCAGAA
TAGATTTCCTTTGTTATGAACTGAGGAACTGGAGTCTCAGCAGAGTTTTTCCCTTTGTTTTCT

GGCAATCCCCCTTGAATGTACACAAGCACAGCAGCTGCTTGAATTGCCCTCTTTGCATTGCTC
ATGTTCACTGCAATTGTCCTTTCAGATATTCCTGATGTTTCAACCAGTTTAAACATTCCTGTG
TTGTCTGGTGATTGTGCCATTGCGCAGTACAAGATAGCATCGAGTACTTTTGTTCCTGCAGCA
ATGAGCATGTTGACATCAGCACCTCTTTCCCCAATGTACTTGATGACAACTTCTCCAGAAAC
ATATCCTTCATCACCGAGAGTGACACTCTTAAGGAAGTTCTCGAGATGCTCCTTGATTTTTGA
GCTATCAATCGAGTAGGACTCAGACTTGATTTTAGCCAAGATGGTCGCTTTATCCTTAAGTA
GTGTGTCGGACAGATCTTCGAACCTATTCTCAGAATTCTTTTCATTATCATTGTTCTTCTTATC
TTGATTGCTCTGCTGATTCTTGTGTTGATCTTCATTCTTATTATCAGCAACTGGTACTATTTTC
TCCCCTTCAAGCATCACTAGTTTGTCAGGGTTGATTGACTCTTTTGCGATTGTTACCACATTA
TTTGTCACAAGTGAACCGTTATCATGAAGTAATTTCCATGATTTCAGTGTGTTCATTTGTTCT
CCGGTCAATTTGTCATTGCCTAAAGTTTGTTGAATCTCGATGAGAGCTTGGAGAGACTTGGC
TAAGATGAATGTAGACATTATTTAGTGTACAATTTGGAAGTCAATAAATCTTAAATAAGCAA
GTTTTTTC
>Seq30 [organism=Citrullus ophiovirus] RNA1 genomic sequence
TTTTATTTATAAAAATAAATAATATTTTTATTTCTATACTAGAAATAGTATTGTCTTTTGGGTG
ACAAATTGATGGCGCAATGCCTTGAGATATCATTCTTCCGACCTTCTGTTGAATTCTCAATTA
CTGTTATCGTTTTTCTCTGATTTTCTACCCAGTATCTATCCATGCTTGTGTCTTTACATAACAA
TCAATCTTATGACACTATTTCGAAACCTTGATGTACTAACTGGTTGATTCTATAAATTATCGC
TAATAAATTGTCACTACTGGATTGTTTTTGTTTTCTCTATTGTGTTTTAATAATCATCATCTAT
AAAGTCGTCATCGTAACCCTCCTGTTCTTTAATCATATCTTCTAAGGACATGTTTTGATTTATT
TGAGTGAATTCCGGATCTAAACCTAAAAATGAGCTTATGACATCTTTCAGTTCTTCCTTAACA
CATTCCTCTGTAACAGGCAATTCTGCCAATTGGTCTTTTGCTACATTCATTTCGCCAGAAAGT
TCTGTTATGACGTTTCCGCGTATACCTGTTATCAAGCCGTTGAACAATTCATTATCATCTAAC
GTCTCTAGGTATTCAGATAGGCTGTTAAATCCAGCTATTTCCTTTAACATTGCTACCTCTTCA
TCATATGTAGATTCTGTCTTTGCATTTCCCTCTGCTATGTCTTCCTTCATCTCTTCATAGCTCC
TGGATCTGCTTGTATTCCTCGAGAGCATCATAATCTCTCTCACTATCTCCCTTCCGCTAACAC
TTCCTTTTATTTCTATATCCATTGTTTTTATGATCTTGAGTATTCTATTCAGCAATAACTTGAA
GTTCAAGCAATCATACTTCTGGCTTTGTGTCTTATTTTTCATCACTAACCTGGTATTTTTCACC
TTTAATCCTAGCAATCTCATTAATATTTCAGGTAATTTCTTGCTTTTGAGGCCCATTATAACA
AGCCTCACTATCCTTTTTGTCTTATCAAGTGATCTAATCCCTGTCTTGTATTTAACGCCTACCT
CTATTGATTGCATGATCTCACTAAATGAGGTTTCATTTGATGGGCTCGTCCTGATGAGCTGGT
TTATGGTTTTCCAAGTTATGTAGCTTGACCTGTCAATCCAACGGCTGTCAATGATAGCCTCAG
AGATTAGCTTTATGTCCCTGCTTGAGTTTGTCGCTAGACCGACAACAAACCTCCCAGATTTCA
TTCGTTTTGCAAGACTGCTGGTGATTGTTATTGAAGGCTCCAAATTTTTTCTTAGGAATTCTT
GTGCCAATATACTTTTTATTATGCCAAAATTTCCTATCCCACCTTTGTTATTTATCTCTATCTC
TGGTTTTGAGATATGTATTGTGGTTGTGTTCTCAACTTTTTCTATCAGTCCGTCCAAAACATG
ATCTTCTAATTCGACAACTGCCATTTCTCCAATTTTTCTACCATTTTGTATTTTGGTCTCAAAG
GACACGGTAGATGGATCATCTTTCTCATTTTCCTCTGTCATCACGGAAATCAACTGTTCATCA
GATATCATTTCATGTGTATGGTCTATCGAGTTTACAATAGCCGATTGAAATAATGATCTCTTG
CTGAGCTTTAGTATCTCATTCGTCAACTTTTCAGTAAAAACCTCATCAGCAAAAGATTTTTTG

ATTCTTCTTTCTGTCCTGATTTCTTCTAGGGTTTCCTCGCAATTTTCATAATTACTTGAGCATT
TTGACATGGCGACTCCATTCTTATTTGTGACTATCGATAAGTATATGTATCCAGGGGATTCTA
TTGATGGAACCAATGCAGACACTTTATGCTCCAATTTCTCAGCTTGTTCCAGTATCGAGGTAA
AGTTATATGCTGCTATAGAATTCAATCTTATGACGATATCTTTCTTCAAGTCCATGATGTCAT
GAATCATAGTTATGATGTTTGTCTCTTTCCCAGAGAAGTGACTTACATCTAATATGAAGAGG
TCATGATCCAAGTAATTCTCGTAATGTTCCTTCATGGTCATGTCAATTGACGATTTGACCTCT
TTCATTCCAGGGAAGCGATTTATTAAGTTATATCCGTCATTACGAGAAATGGATGTGTGGTC
TATCCCTTTGCTGTATAAACAGTAGTGCAGGTCACCTCTTCCTCCGAACATGTCACAGACTTT
AGTCTCGGCAGTTATTACATGAGTGTTCATCAAGTGATTTATTAGCCCTACGGAACTGGGAT
AGACATCCGACATTGTTGGCGATGACACATGCTGAGGCTTACAATGCAATGCACAGATCATT
TCAAAACCTCTCACTGCTGCCCTCACCGAAATAGGGTCTTCCATAGTTTCTTTTCTCAATTCC
ATTTCTGTGTAAGTGATGTCACCGTACTCAATATTTCCATCATTATCTGATGCTTCAGTAATG
TAATTTTTTATTTCTGAAGAGTAATATGACAAGACCAGGTCAGGGGAAAACTCTCTTCCATT
GTAACTCTGATAGATTTCATTCACAGCAATGTACATTTCTTCATGTGTGTATCCCAGAGTCGG
TAGTGCCATAAGGAGCAAGTTGCTGATCGCTTTGTCTTTTATGTTGAAGTGTGCTTGCCCTTT
TCTCAGTCTTTTCAAATTCACGTGTGCGTTCATCTTTGTCTTGTCCTTGAGTATCTGTATCTCA
AGAGTATCTTCATCTTTCTCGACTATTTTGAAAACCAGGCATGATGCTATTATGAATATCTTT
AATAGTGAATTTCTATATTTAAATGAATAACCTAACTCATCAAATTCGCTTTTCATCCCTGAA
TTCTTCTTTAATTCACTGGAAAATATCTCATGGTTCATCATGGATCGCATTACTGATTTTGTCC
TAGATTCATTACCAATGATGTTCATGCTTCTGTAATAAGAATAGAACCTTTCACAAAAGTTCT
CTCTTAACCTTCCTCCTCCGATGCCTCCTGTGATTTTTCTCATGTCAGAATCTGTTATTTGATC
TGGAGTTAATAACTCTTGGTCCTCCATGTATTTTTTAAGATTTTGAAATGAGCTTATGTCGTC
TATTACCCGTTGAGGGACATACGTTTTGTGATCCAGGAATTTGGTCTTTATTTCTTCATCAGA
TGATACGTATAACGAGTAGCTTCTGTATAGGGATGACTTCTTGAGTATATTTTGCGCATCTGT
GTTTATGATTTTTCTTGATGATTCTTTCAGATTCCTGATCAATGGACTGAATTTAGTCGCATCT
TCAATGCCTTCTAGGTTGAAACTCACATCCCTGCAATAATTTCTTATGTCTTGATTCAAATTC
AATCTGCTTGTCAGGTTATTCTCAATCCTTAATTCCCCTAGTAGGACTTTCAAGTAAGTGATT
AGTAATGCGACATTTATATTGTTGTCTTCTCCTCCATTATTCTCTACAACAGAGTTGATTCCA
GTCACTTCAACTTTGTTGGAATTGATGTTTGAGGTCAAATCCCCTGTCTTAGGGTTGAAGTTA
GCCGATAATGCCCGATGAAAGAATCTTCCACCTATGGGTGCAACAACAGAGTCGGAGATAT
CATCGAATCTACAGTCTGAAAATGTTTGCATCGTTAAATTACATAGGCTTATCAGGCTAGAT
TCAGTCTCCTTGCTACATTCTGAGAATTTATTTAAGTCCATGATGATCCATTTGGTATATCTA
ACGATCTCATAGCACTGGAAAATCAACTTATTCTGGAACATCCCTTCAATCTCAACATCCCT
ATCAAATTTTGCCAAGTTGAATTGTGATCGCGCTGGGCCGCCTAGTCTCTTTTTGGTTCGAGT
GTTATCTATGACCAGATTCGATCGTATCACAGGCTTAATTGGGCCTAGCGGGTCCACAGTGA
GGCAGCCTATTATTGGTATTTCTTCTATCTTCAAAAAGTTGAGATTTATCTGGTGTGATTTAC
CATTTAATCTGAAAAACTTCTCTGTGTTGTCACGCTTCTGCACAAGGTAGTCGAATATTCCAT
CATATATCATTACGGGTTCTTCATTCCTCTTGTAAACAATATTGTGATTTTTTGTCCATGCTTT
GTTCCAGAGCTTGTTCATTTTCTTCTTTCCCACAAAGAATGACAACGTTGTGCTGTTATCGAT
TTTGGAAGTTATTGAGTCTACGTAATCAAACAAGGTTGATTCTGCAAATTTAGACGCTATTCT

ATGGCTGAACTTCTTCTGGAGTGTTTGCAATAATATAATCTCAAAAATATTTCTGTCATCTTC
CGAATTGAGCATTAGCAATTCTTTCAATTCTTTGTTGTCTACTTTTGATGGTAAGATCTTCTTC
AACTCATCAGACAATAGTGTTTTCGTGTCGAATCTGCCTCCAACCATTGGAAACGGTTCCTTG
ATGTATGCTCTAGCTGTAGTGGACAAGTTTATCAAACTTCTCATTATTCTCTTTTTCTTGTCTG
AAATTATAGAGTAGACATAACATAGTCTTTTGACGTTTGAATATGAAAATCCTGATATCATG
GTGTTTGGTAATGGTGTCATTGAGTATCCGTATGTTGCATATGGAAGGTACATATTCATCATT
ACTGCTTCTAGCAAGTCAACATTCTTCTCATTGAAACGGAAAAAGTTTGTTGTAAACTCGTTT
TCATCTGGTATATCTGCTTCGACAATTGATTCAAGTTTTTCACATTTTTCTAGGCAGAGTTTC
GCTAGTACCAACATGGAATGCTTGCATTCGAATGTTCTCCTATCTAACTCAACTTCCATAAAC
TTCGACCATTTTATTAACTCCTTCTCAACAACCATCAAGTATCGTAAATTCCTCACTACCATC
TGGTCGAACACTTGATCTGATCTTGTTGTGCTTGAGGTGTATCCACTATCAATAGTCGATATT
TTCTCTATGTCGTCTCCCCATAATATATCTGAATTTGGTGAGATAGCTAGTATTCTTTTGTAA
AAGGTATCGACAGGACGATCATCAAAGTAATGAGTTTTCAGCATGGAGCATCTTCTGCTTGT
CACTTGGGTCTGGGACAACTTAACAATCAGTCCCATCTTCATTGCATAGTCTTGTATGTATCT
TATTATGTCATTTAATGATTTCTCATTCAAGCCCTTCTTTGTAAATACGCCACATGAATCATC
ACTATATGTGGTTCCAATGTATTTGGTAAACCCTATTGATGTCAACATGTCCTCCATCATGAG
TTGTGATTGAATCCCCCATATGGGACCAAACCAACCTTCAATAGCCCCTCGTTGATGCTTGG
AAACAAATACATAGTCTGAGAAAAGGTGTTCATATATTACGTCAATCTTATTGAACAGATGG
ACTATGCTTGTAAAATTTTTCCTTTCTGGTGATTCTAATGCAAATCCATACATATCCGCAACT
TCCTCAACTATGAATTCAGTGTTTTCAGGCCGTTGAGAGGTGTTGTGTCCTGAGTAGTCCAAA
AAGATTGAGTATGAGTCAGGTTCATCCAAGACGCATGACATCTTATGCATGATCTGTCTACG
CTCATCTTCAGTCATAGTCATCATTTGGCCTGGTATCAGTTTCATTGCACGCTTTATCATTTCC
ATTACGATTGATATATAAATTTTCAATTTAAAACTTGCCACTCCATAAAACCTTGCCTCTGTT
TTTAACTCCTTTTCTTTTTCTACTAGTCTGACAATGAAGGATTCTGTTAAATTAAACAATGAT
GGGTCTTTCCGTTCCTTAATGAATAACCGTCTGTTTTCTTCTTTCCAACCCTCTGTTTGCCGTT
CTATTTTAACTTCGTTTATAAGCGATTCTAGATCATTCTGGGTGACATAATAGATCAGCTCTT
TGATAGAATTGCCCTCAGGTGAGTACTTGTCCTTGGTACACGCCTTATCTATTATTCTAGACG
TTATGTTCGTTTCGTTCCCAGGCCTGAGCATCTGGCCTAACCTCACATTGGCATATTGCGCAG
GGTTGCTGATTATGTCGTTATTGAAACTACCTCCTGCTGCCATCAATGTTAGCTCTTGCATCA
GTGTGTAGCTGGAATTCAGTAGTTCGGGAACAACACCATATTTTCTTACATAAGTTGTGACA
ACTCTCATTCTGAACAGCCTTCGTAGTCTCTCGACAGATTCCTTAACTACGTTGTGGTCACGG
TTGGTTCGTTGTGCATATTTCCTCAGCCCTTCATTTTCGCTCACTTCAGCAACTGTATGTAACT
TTCCTAAACAAGACATCTTTACACAAGTATACCCTTGGGCCTCGGTACAGATTCGCATCAAC
TTTTCTGTATGTTTTGGGGAGACTTGATATGAGTCCAGTAAATTGTCTAATAGCGGGAGTGTT
GAAGAAACCCCTTTCATGTCAGACATTAAGAGACAAGTCGCTTCGTACAATGCTGTCATCTC
CACCCTGTCCTTTGTCTTCAGTTCTTTTAACGATATTATATAATCTATAAAAACGTTGTCTTCA
AGGTTGATGTTCGATGATTTGTTTAAATCCTTGCAGATGTTTATCACCATGTCATTCTCAAGA
TTAGTCATCAAGTAGTTCAGCATGGCACTATCCCCAATCACTACCTCATTGTCTTGGTTTGTA
ATATAAGTGAAAGTCTTGCTTATAAACATCTCAAATTCAATTACGAGATCGCAGAGAGCTGA
TCTTACTGTTACCCTATGTAATGAAGAGCCGTCCAAGAATATCACTGTGTCAATCTTTCTGTT

TTCGTGAAATTTGTGTGAATTCTGATATCCGGTGCCCTTGTTACTAAGGCATGAATATGATAT
TTTAGTTTTCAAGTTTATTGTGTCCAACAAATTTACCATCCACATTAGCTTTTGTGTCTCTATA
CTGGGTTCAATCTCCGTTGTGGTTTTCCTTCCTATAGATAGGTCAACGTGACTCATCAGGGAT
GCCTTATTTTTCTCATATTCATGTTCCGCCAAAGATTTTGAGAATTTTAATAGTGTGTTCATCT
TGTTGCTTTTGTTCCCCAAGACTTTTATCCAGTTTATGAAGTTCAGTATCTCGTCTTCTGTTCC
TTGTTTTATCTTTAAGACATCAGACCTTGACATAGGTGAGATGGCATCACGTAATCTCATCAG
GTCAATTTTTGACCTCAGTGAAAATTTTTCAGGATAGTCTTTTCGAAAAACAAATTGATTCAG
ATTGGATTTGCTGATAATTTTGTAAGGGTTGTTCAGCCTAGAGATGAGCGCTGGTTTGTCAAT
GTACTGTCTCATTCGTCCCTCATCTTTCCCTCCTTCCCAGTATATCAAGGTAGATTGGAAATC
AAATTCGGGTTTCTTTGGTTCAAGGGACTTCTCTGATTCCTTTGCCATGGATAAATCATAGAA
CTCTGTTTCTTTGCTCTCTAAGAATTTGAAGAAGAAAAAGTGATTGTTGTCCATAGCTGATTT
TAGTAGTCTATGATATTCCTTCTTTATATGCTTATCAGGTGATACATGCATGGGCTCTCTCTC
AAGGAACGTAGTAGTGCTTTCATGCAGCCCATTATCACTCCTAGTAACTTCAGATGTTATTG
AAGTGTCCATGTCATCTTTTGACGAGATGAACGTTGTTTCTTCAGAAAAATCCATTGTGCTCA
AGCAGAATTGATTGGTTCGATTATTTCAACTGTTTTTTATTAAACCGAATTTGCATGATAAGT
TGGTCCTCACCTCTCAAGAGAATCATCAAGTTGAAAGAGCACACATGTATGAGCATCCTTTG
AAAAAGATTTAGAAATTATCTTCTAACCAAGCTTTAACTCTCTCCCTCTTTAGGTCCTTGTCT
GATCTGATATAAGACAGCTCATCTTTCGAAGGATAATTTATGATTATTGCTTGGCCCTTAGGT
ATTTTACTTCTGTATATCAATCCCATGTGATCCAATGACACACCAACTCTATTCATCAAGGAA
TAAATGTAATTCTTTGTCCTAGGGGAAAATGAATTGAACCAGTAATCATCGGAATACGGCCT
GGACATTTGTTTTATGAATTTACGTGATATCATAACTGATGTAAGGTCTTGAGACCACACTAT
GAGTTCAAATCTTGACACAATCACCTTAGCAGATTTTATTAGATCTCGGGCTTTAGGGGCAA
CATGAATCATCATTTCATTTTGCATAATTGTGGTTTTATCATTGTCATTTCTGGGTTTTGCCCA
AAACTTCAATTCTGCAATTCCTGAATTCTTCATAATCTCTTTTTCTTCATTCGAGAGAAATGG
TCTCAGGCCAAAAGTAGCTATTTTTCTTATGTTTGTAGATTCTCTTCTTCTCAATTCTCTCCTC
TCCCTGTCGGCTTCTATTAGACTGAATCTATTCACTTTGACTCCTAGGTTTTCCATAGTATCA
ATTGAAACTACCACCCAACCATCCTCATTGGTAATAAAAGAATCATTCAATTTCCTTATTTCA
CTTTTTGATATTATTACCCTCGATTCTAGGCTTTTGCTCTTTCCTGATTCCATGAGATCGCTGT
GGCACGTCTTTAAGTCTATTTGTTACTTTGGCTATTTTTCGAAAGAAAAATTATCCCGCCCCA
>Seq31 [organism=Citrullus ophiovirus] RNA2 genomic sequence
TTTTGCGCAAGTAAAAATTATCTTTTAATCATAATTATTATGATCTTCCCCATTACAGGTGTT
TAGAAGCGCAATGCTTTGATTGGACGACAATGACCATTCTCACAAACCCACCTTGTCATTTA
GTCACCTTTTCTTTTTATTAAACCGAAAACACATTAACATAGCAACAGGTAAGAAGATAGCT
TTGATAGAAGCATACAGAACTAGACACATCAAGAGAAAAACTTGATCACATTGTCAAACCA
CATTAGGGCTCAGTTATACTCTAATTAAATCACAGACTTTGACTTTCTGAGCCCTTTCTTAGA
CATATAAGCACTATATTGGTTAGCTGACATATTTCCGTTTTCAGTTATCTCATTCCAGATGTT
GTCTTTTAGCCATACTCTCCCAGGTTCGGAGCTGTCGAACATCATCACAATCTCTCCTTCCTT
CTCTAAATATGTATTGTCGCCCTCATCTCTCATCTTATCTATTATTCCTGATTCCTTCAATTTG
GAAAACGACAGTACATTCGAGGCAAAGGAGTCATCCGAATAAAGCCTAACTTCACCCAACT
CAATCCTGTTGCCAAAGGTTACGATGAAACCAGTGGCTAATTCATACTCAAGGTTTGCAACT

CCTCCGACATTTTCATCCACTCCATCAGGTTGAATAGATGAACAGTAGAAATAGTGTGAACC
TGATCCTGTGTCGAGCATGGTTAGAACATCATCAGTGGTAGGAACTGTTATGGTTCTTTTTC
TTTTATCGGTACACTTTTCAGAAACCTAGGAATCATGGATGTGCTCTTTCTGGCTTGTTCAAT
CAGTGATATCACATCATCTGATGATGAAGACTCCGAATCCTTGTAGTTCTCGTGTTGTTGTGA
TTCTTTTGTTGAGATTGAAGTATTGTCTCTCTTTTTCAGTTCTTTTCTTCGCTCGTTTCTTCTGT
TCTCTATTTCCATTCTCTTGTATTCCTTCTTTTTTCTTTTTACCTCTTCGGAAACTCGATCACCT
AAGCTCTTGAAAATTGTTTTCTTATTCAAATCTACTGGACATGCTACATCGTCGATGTAGAGT
ACTGTGGTCTTTTTTTGCTCACACCTCAATGGTATGGATTCCTCTTGAGCAAAGAAGGCCACA
TTTAGGGCAGCATAGCTCCTCCCAGAAATCGGCACATCATCAGCCATAATGTTTATTTTTATC
TTATCCATGTCATCTTTGCGCACGAAAAAATTCATAGACAACTCCACTAGAGCCATTTCTCCT
GCATCTATTGTTACTTCTTGAATGACCGGATCTCTGCCTGATTCTTCTCTTCCTTCATCCAACA
ATGCAAATTTTACTTTTCCATTATCTTCCTTTCCTCTCTGAAACAGTGGTATGTACATGAATTG
TATCTTCAGAAACCTTACAAACTCTTTGCTAGTCTTACCCATGAGACTGTTTATCCCTTCTAT
GACCTTTTTCATTGTTGCCATGGTGAGTTTTTCTCTGTCTCCTCCTTCTTCAGCTTCATCACC
ATCGGTACGACTGCCGTTTTTATGACTCCCTTTGCCATGTTGCAGAAATCAAAAGTTAGTTCT
TCAATTCCTTCAGGCAGTGCTCCTCCATCTTTAAGGCTAATCACGTTTATCTCGGAAGGTTTT
AAAGAGTAATCAGCAAGATCTTTCGCTTTGATACGTCGGTTTGATTGCATCCTAGTCATCCG
AGATTCATGGTTCACTAGTGCCATTTTTCAAGCTAATAGTGATTACTTATCAGCGGGATG
>Seq32 [organism=Citrullus ophiovirus] RNA3 genomic sequence
TTAAATTACTATTGTTTTTATTAAAGCGAAACGTGCATAATCCCACCGTATCAAACCAACAA
AGCTTTCAGCGGCCGTATGCTTTCTTCAGGGCCTCTTCAGAGATAGACATGAAATCAGCGTC
GTTGTTCTTAAATATGCCCCAAGACCTTTCTCCGTCTATCAGTGTCCCCATCAAGTTCTCATC
CCGCAGAAACGACGAGTTCTTTTCTGCCATTACCTTGTTCGCCATTGATAGCCTTCCTTCTGA
TGTCATGCAATACAGTATTGCACACGTCAGCTTGAGAGTCATCTTTTGTACACTTGGCTTAGT
GTCAGATAGCGGGTGCATACAGAGCTGTGCTTTGTAATCTCCGTTTAGTGATCTTAGCACTG
CTACTAATTTCTTTGCATGGGTTAGCTTGTTACCCTCATCAAAAAACATCTTTAACGTTTCTG
CAGGGACGTTCTCTGGAAGCTTAGTTTCTGGTTTCTCTTGAAATCCTGAGCAATAGACTGCAT
ACCTGATTGCTCTGTTCCCAGCAATATTCAGCTTGCATCTTGATGCAACGGCAGGTTGAAAG
GAGTTCAAAGTCCAGTGACAAGAATACTTTTGCGGGAACTTCTTGGTTGAAGCAACACTGAG
ATCACTTCCAATTTCAGACATCGTTACATTGTCACCCGAGTATATCTTTTCTTTGATGAATCTT
GGTACATTCTTTTCTTCTCCATCAGATTTATTGGGCAAGGTTCCTTGTGTGTACACCAGTATT
GCTCCTGCCTGGATAGCTCTTTTAGCTGCATGAAGATTCCTAACAGCTTCAACATCCCTCAGA
TCTTTTATCTCCTCCAAATTGAACAAATCCTCAGATCCCTTGACTCCTTCCATTGATTTGAAT
ACTATGGCATCTAATATTTTTGTTCCAGCAGCAACCAGCTGTGACACCGATCCAGTGATGTCT
TTGAAATATTTTATGTCTAAATCCCCACAGTTGTAGCTTTCTTCTTCGACTCCGATAGTAGTC
AGAAACTCAACTAGATATTTCTTTATTGAGGTGTGATCGAGAGAGATCGCTTCTTTTCGAAT
GAACTTTTCTAAGTCTTCCTTATTTTTAACAAATTCTCCTGTTTTTCTCACAAGCATCTTTTCT
AAGATACCTTTGTTCTCTGTCTCTTCACTCTCCATCTTCTCTTTCCCTTTACCTTTCCCTCTAGT
CCCCGACCCACTGTCCTTGGAAATCGACTTCCACATTTCCTCCTCCATGACCTTATACTCCCC
GTCTATGATCACAAGCAACTTACTGGATTCCCTAAATTTCGCATGCTCTGTGACCTTCGAGTA

AGCATTTGCGTTGATTCCCTCATCTCCTATAAGCTTGTACTTTCTCAGTATCGATTCTGTCTCC
TTCTTCGTGTCTTCTTTTCCAACCGTGTAATCATCGATAGCTTGAAAAACTCGCTTCACTGCA
TTCCATTGTGCCATGTTAGCTTACTCCTCTTGATTGCTTGGAAACTTGAGTGATTTTTCTAATG
AAAAATTATCCCCCGC
>Seq33 [organism=Conopholis ophiovirus] RNA3 genomic sequence
AGCAGAAATATATAGCCATAACAGTCAGCAGAATATATATATTATTTATTTATTTAAAGCGA
CATGGAATTGTAAACAGAACAATGCAAGATTTAAAGATGAACAAAAGCATAACAAGTTAAA
GAAGTCCATAATAACTCTTAAGCTGCTCAACTGTTATGTCGTTAAAATCAGCATCGGAGTTA
CTCAACACCGTCCACACATGGACACCACTCATCACAGCACCATAGAAATTCTTGTCAGCCTT
GAAGGACTTCACATCATCATCATCAATTTTCCTTATCATAGAGACCCTCCCAATCGGTGATA
AAGACTCTAAGATTGCCCTTATTAACTTCAATGTTAGGTTTTTAACAACAGGTCTAGTAGGA
CAAAGTGGATGCATACGTAACTGCGCCCTCCAATTTCCACTTAACCCCCTTAGAAAAAGAAC
AATGTTCCTAGCTCTTTCAGATTTCTCATTCTTCCTCATATATAATTCCCATTCTTCAGCAGGT
GTATCATCTTTGGTCAATTCAACTTTTCTTGATGCTTCAAAACCTCCAGAATACACTGCATAA
CGAATTGCTTTATTCCCTGCAATTGATAATTTGCATCTTGCTGCAAGAGGAGTGGGTAATATA
TCAGGGCTTACTTTCAAGAACACTCGTGATGGAAATTTCATTGTAGGAGCTGATGATAGTTG
TTTTCCTATAGAGACTATGCTGTCAACTTCACCTTTGTAAATAAAGTCCTTAATAAACTTGGG
AACAGGACGAGTATCTGTTCCAGAGTTTGGTGGCAGTGTTCCTTTAATGAAGCAAAATACAA
GAGCAGATTGTATCGCTTTAGTGGCATCAACAGTATTTTTTGCAGCAGTATTTGACCTTAAAT
CAGGAATCACAGTTATTTCAAATATCCTGGCAGCATCAGGGTTATCAGCAATCGAAGAGTAA
ATCAATGCATCAAGGACTTTTGTTCCTGCAGCTATCAGCCTGTTCACATCCCGTGTTGCAGTC
TTGTACATAAAAACCTTTATTTCACCAGGATAGTAAGAGTCATCCTTCAGATCAGCATTTTTT
ATAAATTTCTCAAGGTGCGACCTAATCGCTTCTTCATCAATTTTAAAACGGCTTTGTTGGTTC
AAAAAACTCGTTAATTGCTCCTCAGTTTCCAGCACAGTATCCTTTCTATTCACTACTATTTCA
TCTTGTGGATTTGATCCTTGCCCTTCTTCCAAGTTAATTTGTGGTGGTATTATCCCAGATTTAA
TTGGTGCCACATCATTCCCAACCATTATCACACTTTGCTTACTTCNCGTGAGCGTCTGAGCGA
AGATGAAGTGATGGGAAATCAGGAGGCTGAAGTGGCAACGGCAAGGCTGAAGCGGCGACG
GCGAAGAAGAAGATGTCGGGGATTGAAGATGTGATGCGCCGATTTGGGGAATTGAGAATAG
ATTGAGAATCCTTGTGTTGGTGCCCCAATTTTTCTTCACTTTTTTACTTTTTCGTATTTTGTCAT
TTTCTCTCCCTCTCCCACCCAATCCAGCCGCCTCCCACTGTACTGCTCGACTGGCGGTAATCA
TCTCACCCCCCACACCTTGTTTATCTTCAGGTGAGCTCCTTTCATGGTGGATTTCTTGGGACA
GAATACTAAGTAAGGGTGAGACTGGTTACCGTCGGTCCAGAGCAGTACAGTGGGAGGC
>Seq34 [organism=Cyrtomium ophiovirus] RNA1 genomic sequence
TTAAATTCTTGTATATTCTACTGTTTCAATTGACAGTGCTGTCAAGATTGTTATGCACATATA
TTATATTTACAAGGACCATCTCAGAAGATGGAGAAATTAGTCTTCGTAACTGTAATCCTCAA
AATCATCCCAGTCATCCATCACATCTAGGAAATTTATCTCACCGAGTGCTATCTTTCTTTCTA
TCTCTTCATTTTCTGTTATCGACTCCTTTGCTGTAAAGAAACCTTCTATTACTTTCTCTTCTGT
AAAGAAAGATAATTCTCTACTTATAGAGAAATCAATTGACTCAGCTTCTATTTTCCCTTGCAC
AAACGAGAAGAAATTGTCTGAACTCTGCCACAATTCAGACAATAGCTTCTCTGCAGTTTGGT
ACTTATTAATAAGTTCAATGACCTCTTTTGAGTCAGGTTTAATGCCAGGGTCAAGACCAATA

TGTTCTTTTAGATATTCTGTTACATCTCCTCTCTTGTGGGATATATGTACTCCAGATGATAATC
GTCTGATTCTATTTATTAATAGCTTATAATTCATTATATTTACTCTATTTGTTTTAGTCTTAAT
TTGCATTAGATTCCTAAGCTTTCTAGAATTTAGACCCGATATAGCAATTAAACCTTCTCTTGT
TCTTCCTGATTTCCATGCTAAAACTGCCTGTCTCATAGTAGTATAGAGTGTGTCCATAGTCAA
CCCAATACCTTTGGTTCTTAATGATTCTATAGATATTTGTTTTACCATTATATCAGATGATATT
TTACCCTTCCCAGCGAGTGATGCCAACATTCCATATACTTCTTTAGTGTGATCGTTTAACCTC
ATACCCATAAGTTGCTGCACATATTTGCTCAAATCTTCTGCTGACATATTTTCAAATCCTGTC
ACCCATCTCGTGGGAAATATCTCTGATTGTTTAAGAGTCACAAGGTATCTTGTGTTTCCAGTT
GTCATATATTCCTTCAGTATGTCTGAGGCGTAATCATAATTGGAATTTTCCATGGAGTGTTCT
GATACAATAAATTCAAAGTCAACCGTCGTCAAGCCTTCATATATATTATTTTGATTTCTTAAT
GGATAGGCCATAAGTGTTCTTAAGTCGATCGATGACTTTATACTATCTTTGAGTCCTTGGCTA
ACAGATATATAATGATCATCTGCTCCGGGAACCAACAATTCTTTCATTATTTGATCGTCTGAT
ATGAATTCTTCTGTGTGGTCTTTTACTTTATAATGCCTTGTAGGAATTAGTCTTTCTGCTGATT
TTAGGGTATCAAGATCAAGTAACTTCTGGTATAATATTGATTCCTGAAACCCTGTTTTGTCTC
TCTCCACTTTGGTTACTTTATGTCTCTTGTTACCATTTATATATAGGTAAAGAATCCCAGGAG
AATTACGCTCAGGAATATACATAATTGCTTCACATTTTGAAATCATCAATATTGTTGCTTCAT
TTAGATGTTCTGTTATAGTGTTCATTCTAATAATAATCCTTTTCTTCATTAAGATTAATTCTGA
GATAGCTTTATTCATAAGCTGTTTCTTTCCTGTGATGTGTGACACATCAAATAACATTACATC
ATGATCACAATAATCTATGTATGATGAATGTTCAAATGCATTCCAACCTTCTTTTTTCGTTAC
CATTCCAGCTATTCTATTCACCAGATTATAACCATCATTCCTGGATATAGAAGTGTGTGATAT
ATTCATATCTTCCATTGCTACGTGGAAATCACCTCTTCCTGCACAAAAATCGACAATTCTATC
TGATTTCTTGATTTTGTGTGACTTGACTAATATATTCAAAATGCCTTTAGCGGATGGATATAC
ATCAGATTTAGTTGGACTAGAATATTGTAACGGAGTGGCAGCCAGTGAACAAATATATTCCA
TTGCTTTTATTCCTGCTCTTAATGCATTTAAGTCACTAAGATCATGGTAACCTATTGGTACAG
ATGTGTAATTCACCAATCCATATTCGATCGACCTATCAACAGTGATATAACTGAGATTTTCTT
TATTGAAATATTCATATAATTTTACTTGTTCAAATATCATGTTGTCAAGTTCATCTGCTAACT
CTAATGCTGCAAATTCTACTTCATCAATACTGTATGATAATGTAGGAAAAGCATTTAAAAGA
ATCTGTGATACTACTTTATCTTTTATATAGAAATGCCCTTGTCTCCTAGATAATGCTCTGAAG
CACCTAACTGAATTTGACATTGTTGCTTCATGATTTACAGACAACCTTTGCTTATTGAATTCA
TCTGGTTGGATCACAAATACCAAACCACAGGCTATAATGAATACTCTTAACAATTCATTCCT
ATAAGACATGGAAAACCCAGCAGTCATCAATTCTCGGGACCACATTTCACCATCTTTGTCTT
TCTTGAATAACTCCTTGAAGATCAAACTTCGTATTAATGAACCATTACTAGATTCTTTACCCA
TAACATGGCAAGATGAGTAGAATGTATAGAATCCATCAAAAAAGTCATCAAAGCTAATTTCT
TTCTTTTTAAGCTGAGGAACCATTGCCTCCAAATCATTCTTATTGATTATTTCAGGGCTTATT
ATATTCTGATCCGACATGTATTTATATACTTGCTTATAATTAGATTCCACATTTATTATGTCTA
AGGATCTGCCCATAGCAAATCCGATGAAATCTTTGGTAGATGTAGCCCCAGCTAGTAAATAC
TGTGTGTATGATTTATATATCGGTGCAGTATCTATTAATGATGAACTTTCCAATCTAATAGGA
TGTTGTAAAGCATGTGCTTTTCTGATACCCATCAAGGTGAAAGAAACATCTCTTATCTTATGG
CATATATCAAGGTTCAATGTCAACCCCCTGAGATTCTGTGGCGACACTTTGTTATAACCAAG
AATAACTCTTGTACTAACCATCAAGTATTGTAAATTCAGGTTATTATCTTCACCACCTAGTGC

ACCATTCAATCTTTCTAACCCAGCCAGTTCATATCTTCCTGTCCAAACCTGTGAAGTAAGATC
ACCTGTCTTTGGATTGAAGCCACCTGCTGTAGATCTATGGAAATAGCGACCACCAATTGGTG
CTACTGTGTAAGGTTCTAGATCAGCGTATCGGGCTTCTGTAAATGTGGACAGTGTGCAATCA
CAAGCGCTGATGAGACAACGTTGCTCCAGATCAGATAGGGTACTGTATTTCACTAGCTCAAA
TATGAGCCACTTTGTATATCGGGTTATTTCGAATGCCTGAAACACAAGTTTGGACCTGAATA
ATGATTCTATGCCTAATTCCCTATCAAATTTGGGTGAATTTATAGAAGAAGGAGATGGAGGA
TTTCGTTTAGGACCATTTTTTGTGATCGTTTCTTTTCCTCTCATTATAGGTTCAAACAGTGAGT
GTAATGAATCAGTATGTACTTGACCTAATAGAGGAACCTCTTCAATTGGTAGAAATATTAAC
TCTATGCCTTCACCTTTGAGTGATTTAGTATGCATATTCCTGAAACGTATTAGCTCATTATAA
TTGATTATGTCCTCATTCTTTGTCAACTTTACATTGATATGGTGATTTGATTCCCAGACGTTTT
GCCAAGTTCTTGCATAATAACCTTTACCTACTATGCTCTTTAGTGTTGCGGATGAATCCACTT
TAGAAATTATATCTTCTAAAAAAGAGAATACTGATTCTTCATAGAATTTGCTTGCTATTCTAT
AAGAGAAACAATTAGCAAAGCAATGTACTATTTCTGCCATGAAATTGTCTATATCCTTCCTA
GTTAATGAATTAAGAAATTCTTTGAGTTCATGATTTTTGCACTTATGAGGTAAGGCAGTACTG
AGCATTTTAGTCAATTGCATTGTAGTATCTTTATAACCACCAGTTAAAGGCACTGGATTCAA
AATATAAGAACAAGCTTCATCATTGTAATTAATTAATCTAGATAATAGTTTCTTGTTCTCATG
GTCATGCAAAGATTGAACGTAAGTGATACGTTTGACATTGGATATTGAATAGCCTGAGATAA
CTGTATCTGGTATATTCGTAAGTGAATATCCATACATACAATGAGGAAGGTAAAAAAATGTC
AGAACCCATTTCAGTATAAGGGGATTTCTGCATGCAGATGATAAAACAATATTTCATTATC
TAAGTCTTTCGGGAATAAGTCCCACATCATTGGATCTTCCACTGTGCTATAATCCATGACTGC
AATTGATTTTTTGTGCATCATCAATTTATCTGTGCTATGCAGTACCAATCCCTTGTATGTGGG
ATTAAACTCTAATTTAAGCTCCTTAAGAACATGCATTATATCCTTGCCTACTACACAGAGTGT
TCTAAAATTGCGTATAATGACTTGTAAACGGTGATTATCAGATCTTAAACAAGAAGATGTAT
AACCTGAATCAATGTTTCTGACTAATTCTAAATTGTCATGCCACAAACCACCAGAGTTAGGA
CTTATAGAAAACATTTTCTTGTACGTATTTTCAATAGGTAGATCTTTATAATAGTGATTTTTC
AACATACTGCACCTTATGTCTGTGATCTGTGTTTGTGATAATTTAATCAACAATCCCATCTTC
AGCCCACTATCTTGTATTCTGTGTATCATTTGATCCATCTTTTCTGTTGTGAAATTAACAACTT
TGAATGCAGCACATGCATCATCAGAATAGGTTGTGCATATACTTTTCCTTATATTCATATCTA
TGAACATCAATTCCATCATTAATTGAGATTGTATCCCCCATAATGCACCAAGCCAGCCTTCG
ATGGCACCTGTTTGTCCATGTGAGTAATATATATAGTCAGTATAGGTATTCTCTGAGATAATT
TTTATATTGTTGAAAACATGTATAACCGCCATGAATTCATTATATTCAGCTGTACCTCGTTCA
TAGCCATACATGTTGGCTATTATATCCATTATAAAGCTTGTATTGTCAGGAGTTTGTGATGTG
TTATGACCAGAATAATCCAAGAACACTACATAAGTATCTTTTTCTCTGAGGAGTGAACTCAT
ATCATGCATTATCTTCTGTCGCTGATCTTCATTCATTGTCATCATCTGACCTGGTAATAATTTC
ATAGCCCTTTTAACCATATCCATTACCTTGCTGATTTGAGTCTTATAAGCAAAAGATGCTATA
CCATAAAATCGAGGATCTTCTTTAAGTTCTTTTTCTTTCTCAACTAATCTCACGATGTCAAAC
TTTGAGGAGATCGGGACAGATTTCCTATCATAAATGTCACACACCCTATTATTCGTACTACTA
GTGTAAGGCAATGATTTGGGTAAGACATTCCAGTCTTCACAATCAAAGTAGTACTTTAGTTC
CTTAACAGAATTATATCCATTTGAATGATAGTCATCAGTCGTACAAGCCTTATCAATGACCC
GTGAGTGAATATTTCCTTCATTACCTATCTCTAACATTTTATTTAGTTTGACATTGAAGTAAA

GATTGTAATCAAACACTAGTTCCTTGTTTATAGTTCCTCCACATACCATGATGTCTAGTTCAT
ATTTCAGTTCCGGTGACAGATTGCTAATGTTCGGCATAGTCTTGTTCTTTTTGATATAATTAG
TTATCATCTTCATCTTGAAGAGAGCCGCAAGTCGTTCAATGTGATATTTGTCAACAATTTTCT
TACGGTTAGTCCTCTGAGTATATCTTTCCAAACCCCCATTATGATCTACTTGAGCGTATATTA
ATGATTTGTAGATACTACACATTTTTATGCATGTTTCAGGTTTGCTTTCTTCAGCTATCTTGAA
CAACTTAGTAGCAAAGTCTTTGTCAATTGATATTGTGTCATAAAGATTATCTAGCAAAGGAA
GATGTGAACATTTACCTTTAAAATCAGCCATGAGTAAGCAGGTTGTTTCATAAAGTGCAGCT
AATTTGACTCTCTTACTCATGTTATAGTGCAAGTGTACTTCTTGAATATAGTCAATGAATCTT
ATTTCTTCAGATGAGGCATGATCTCTTATCACATTCAACGTGAAGAAATTGTCCGCCATTGTC
ATAAGATAATCGATATAACTTCCTGGACCGAATGACACTACACCATCATATTCAATGGCCGA
AATGGTGCGTGATTTATACACTTTAAACTGCTCTTTACCCACTTCAATCACTGCTAACCACTG
CCCATAAGATCCTAATGAGATTTTAATTCGTTTTCCTTCGTGAAATGCACCAGAATCCTTCAT
TGTTCTAGTCCTATTCTTAATGACATTGCTTGCTAGAGCTTTTACTTTAAAGTTGCATGCTTCA
AACAAGTCAATTACCCAAGATGCCTTTAAGGTGGTGCTTGATAATTCATTATCTAGCTTTGA
GTTGTCAGCCTTGTTAGTGTACATCATGAGTAGATTATTATTTTCACAACGTTCTAGTGCAGA
TGCAAATTTCCAGTATTTACTAGATTTTACAGATACCTGAGCCAACTGTAGCAGTGTGTTATG
CATTGTTATAAGAGATTTCATAGATGAAGTGTATTCAAGATCTCGATTAGAAAAATATGACC
TGAATAAATGCAAATCCAGCTGCCCAGTTTTAGACCATTTTTCAGGATACATTGAACTATTA
AGGAAAAAGTCCTGGTTCTCTTTCGAAAATGTAACAAAAGGGAAATCCAACTTCACGATTGG
TTCAGGTGGAAGTTGCTTTGTTGGTCTAATATGTTCAATGGAAGGAATTAAGTATTTGAACTC
TTGCTTAAAAGACAATTTTGGTCTCGGTGGTTCAAATTTGCTCTCTTCTTTCCGTGAATGCAT
CAGATTCATATGAGATGTTTGTAGGCGCTCACATAATTCATACAAATACGGATATTCAGCTT
CGGACAATCCGAGTGAATGGATGTAATTATAAAATGGCACAAATCGATCTTTATCTTCTGTC
TTTCCTGCAGGTTCATCAGCGACTTGCTCGATGTCCGATAAGTCCGTATCATCGTTTGTCTTT
CGTTCATCTTCCGAGCTTTCGGTTGTGTCTGTGTACCAACTTCCACTATCTTCACTTTCATCAG
TATCACTTGATCCATCCATCACTTCATCCTGATCATCAGGAGGAATTTTCTCTTTCCATATGG
GAAGATCTAATACATCTTCATCGGTTATATCAGCCCAAGATATCTCTGACTTGTACTCCATGG
TGATGATCACCATTTTGTTTAAAACACTAGTTATGCAAATGTGTTAAATCAACCCATAGATG
AAATTATGGTCAATATCGTGGCAAAGCCAATAGTCATCTGGTAAATCATTTGGATCAGCAAT
GATTCTAATTAAAATAGGACGCAAATCCTTTCCAATGATTCTCCATTGATCAATGTGGTCTGT
AGATTCTGTAACATAAAGTATATGATTCCGCTTAGCATCGGTTTTATGATGTGCCATATATGT
GTATCCATTATGACGAATCCCAGTGGTTGACTTGACAAATGCAGCATAGAATCTGGATTTGT
CATAATCTATAACGCCTGTATTACGTTGACTCAATTGTCTGAAGTACATGTTACTATCACAAA
TTTGCAGTTTATT

>Seq35 [organism=Cyrtomium ophiovirus] RNA2 genomic sequence

GAAATGACATCATCCAATTCAGCAGGTCAGTTCTTGGGATCCTTGTACTTATTTCATATATAG
TAGAATCCTCACATGAACTCGACTTGACAATATAAATTTCAGATTTATCATCGTAATTTATAT
CAGAGCAGACCATGATCGGAAAATTATCTATATCCATTATAATTTTGCAAGTGGAGTCTTCG
GTGAATAGTATCTGAGTCATGTTCATGTTCAAATTTATTTAAAACAAGTTACGAAATATAGA
AGACTTACATCCATAATTATTAGTACCCAATCATGTTGATTATTTCAAATAACTTATTATCAT

TAATGAAACTTCTTACTACTAATTGATCATCTTTCTTGAGTATCCATTCATGACCTATAAACT
TTGATGAGTCAATGTTATTATGGATTAATAATCTCGATAATGATATTATATTCACATTAATGT
CATGAGTAACAAATACATCCTTAAGCATTATTGCTGATTGTTTGTATTCAACTAAGTATTCTG
GGTATAGATGCCCCTTCACTTTACCATTGATGGTGTCAAACTCTTGATCCGTTAATCCATTTG
GAGCATATACCAAATGATGTGATGCCCCAGAATCAATGAACCACTTGCCTATCATACTATCA
ACAACACGATCAAGTGTCATAAATACAAATCCTTGAGTCTTAGAGCTATCACCCTTGGTTTC
AACTTGTTTAAACTTACATGTATCCATACGTTTTTCTCTATTTTCTTCAATGTTACGAATCATA
TTCATGGATTTTGTAACATCAATCTTTTCCGATGATCTTAGCTGTTCAAGTATATTCTTGTTTT
TCATGTGTTTAGCAGGCTCAAAATTCGCAATGTAGATACTCTGAATAGCAATGTCTTTGAAG
AGTTTAGGTGTTCTTAAAGTTTCAATATGGGCAGTTACGTATAAGACCCCAAATTCTCCTTCC
TTTAACGACACATTAGCCACTGTCTTTATCTTTATCTGAGAAAAGTCTTTTCTCGTGACACAG
TATGACATCGTGAAAATGTGAAGAGACATCATGTTTGATGCGCATGAGATTTCCTGAATAAC
AGATTCTGATCTTTTCATGCGATTGTCACTCATTGTTATAATTAGTTCATCAGTCTTGTTGTTG
GTAATATACAAAGGCACAAACAACATCTCTAATTGAGCAATTTTCACAAAAGGCGGGGTTAT
TTTCAACTTCATAAAGATTTTATCTATACGGTCATAAAGGCTAGGAAGTGAATATTCACTAA
CTTTGTTATTGGCTTTCACAGGAAATGTCAATGGAATTCCAATTGTATGGATACCTGGATTTG
TGAAGTTTCTTAAATCACATGTAATGTCTCGCAGTCCATCTTCTAAATCACCTGGACCTTCGT
AATTAATCAGCATCTCATCATTGATATCTTCAAGAAGTGGATCTGCACTAATAGCATGTTGC
GTTACAGCTCTCCTTGAATTCACAGAATTTGCTCTGATGAGACTTAGATTTTCCATGCTGTAG
CTAGTATCGCTGTTTTCAAGAATTTCAGTTTCTAATAACTGATCGATTATTAAATATAAACCT
TAATTATGCTTACCCTTGCATTGCCACTGTACTCGGCGATATGGAGAGCTTTCCTCAAAAACA
AACAGTGACGTACTGAACCGTAAGGACGGAACTCCTTCTGTCACTCGCCATTGTTTTTATTTA
AAACAAGACAAGAAGACAATGTAACCATTCGACCCTTATTCAGATCTACCATCACTATCATA
ACCACAATAGTGATACTCAGACCCACTCCCGATGACTTCATCTATACGACGGAATGACCAAG
CTATAGGTTCTGATTCAACAAAGAGCTTGTTGTTAATCAGAGACACATGGTTACCTTCTTCAA
ATAATTTATATCTCTTATCA
>Seq36 [organism=Cyrtomium ophiovirus] RNA3 genomic sequence
ATTACCAGGATTAATAATTAAAACTTTGAAGTCTTTGCCAGCTGAGTTGGGGTCGATAAAAC
TTACCATCTGATTTATTTAAAACAGATGCACTTAGTGCTCATAAAACAGGAAGACAGCAGTC
ATTTCTTACCAGAAGCAGACACGATCTCTTTAATCAATTCAACTGACAAAGAGGTAAAATCA
TAATCACGATTCTGGAGAGAAGGCCAAGTACGATTACCAGGGTCCAGAGGATTATCCATCCC
AACCAAAATTTCATCCATCAAGAAGGAGTTCAACTTTTTGTTAGTCACAAGCTCATATAATT
GTTTACGTCCCTTCTCAGTCAAACAATATATAAGTGCATTAGTTGCCTTTGCAGTGAATCGTT
GAGGAATTTTCCTATCCATGTTCAACGGATGGAATTTAAGGAGTATATCAAAATCGCCAGAT
TTTCCTTTCAATAAGTCCCGAATCTCTAGCGCCATTTTCAGCTTTTCTTTCTCTTTGAAATCTT
CCTCAGACATTTTATCTTCACTCTTCTTGGGAACTTTGGTATCTAGTCCAGCTGTCAAATCTG
CATAACGGAGGACTTTACTCCCAGCCACGGTTAAACGAATACGTGCATAATACTTGTCAGGA
ATATGATTCTTTAACTCCAATTTTAGGAGGATATGAGCTGGGAATTTTCTACAAGGAGCAGA
TGAGAGGGCATCAGCAAGGTCACTCTCATTATTTATAGTAGTATCATTCAATAGAGTATTTG
CTACAAGCCCTTGCAACTTTCTTCCTTCACTTTCTTTATTTTTAGACGGAAGAAAACCACGAT

TAAAGATCATACAAAGTCCTGCAATGAATATGTTGCGACTGACTGTAAGTGCAACTGCGCCT
TCACTCATGCGTATGCTATTCAACCTTGTTATTGTAAACATGAATTGTGCATCCTCACGATCA
TGGTAGAGGGCATAAAGGATTGCATCAAGAACACGAGTACCTGATATGATAAGATTTGTTA
CATTGATAGGCGCATTCTTCACTAACTGAATTTCAACCTCCCCAGGCAAATATGTATCATTTA
CCGGTATAGTCTTAACAAATTCAGACACAATACGTGACAATCCTTCTTCATCATGAGAAAAA
GTAGCAGATTTGAATCGCTCCTCAAATTCTGCACCAACAACCATAATTGGTTTTCTAGATGTT
CCACTGCTTTCTGCTTCATCCTCTTGTTCCTTCTTTCCTTGTCGGCGTCTTTCATCCCCATTTGA
TCCTTTACTATCATCATCTGAATGTTCTTCATCAACCTCTTTTCTTTCCTTCTTTGGCTTCTCTT
CTTCTTTCTTTTCCTCTTCTGATTCATGACTTGATGAGTCCTTGGCATCTCCTTTATTCTTTTTA
GGAGGACTCTGAGATCTTGCTTTCATTATTTTCAGTTCCCCGTCAGTAACTGTGAATGATGTA
TCAGGATGAGTGTCTCGATTTGATTTGATGAATTCCATCACATGTTTAACGAGACCCCCATTT
GACGCGATAAAGCCCCATTCGCGCAACAGAGCCGCTTCTTTCTCACTCGCCTGACCATCATT
GAAATTCGCGATCATGGAACGCAGAGACTTCAGAGAACCAGCAGCAGATTGAGACATCTTG
ACTACAAGATAACAAGATAACAAGCACGGATAACGAACTCCGATGATATAACTCGATAGCA
AAGAATTTCAGTTCTTAGTAACTGATCCGGCCCCATTCGCGCAACAGAGCCGCTTCTTT
>Seq37 [organism=Datura ophiovirus] RNA1 genomic sequence
GTCAAACATGGCTTTTAAATTTTTGGCTGCACTTTGTGCTCTGTGCAATGAGCAAATGACTAC
ATCAAGACCCCATTAAATAAAAGGAAAGAACAATCAAAAGACATAAGAAAAAGCAAATTG
CTCATCTTAATCAATCATAGCAATAATCATCATCTTCCTCAAATTCATCTGATGTTTTGCTTAT
TTTTATCATCTCCTCAAGTGTCATATTCCTATTAATTTCATATTCTTCAACTGTGGTTCCTATT
AATGATTGAGTTAATTCTGTCAATAATGAACTTTTGTCTTCTATCTTCTCAGAAAATGATTCT
TCTTCTTTATTATTTTTCACACTACTTACTAAGACATCAAATAGCTCATTCTTCGATGATTTTT
CAAAAAATTCCGAGACAGATTTAAAACTCTCTATAAACCTTTTCAATTCCAGCTCCTCGATTG
TGTTAGAATTCCATTCAAATGTATTACTATCAGTTAAAGATTTATTGCAAGTCACTGAAAGA
GCCATATCGTGGTGTATTCTCCTCAATCTGTTAATTATCAGTTTATAATTCAAACAATCATAT
CTCTTATTCTTTGACTTATTTTTGACTAATTCATTTAAGTTGAGCTTTTTAATGCCAGATGTTT
CTTTTAATCCATCAAGTATTCTTCCTGATTTATACGCTCTGACTGCTAAATTTGCAATCCTATA
AATCCTAGTCATATTGTCTGTGCCATCCAATTTCATCTTTGATATGTTAAATGACTTTATAATT
GTGTCAGAATTAAAAACAGTATTCGATCTCACTGAAGCCTCATAAATGAATTTCCACGCCAT
TCTGCTGGATTCTTTTATTCTTGAATTGGCTATTGCTTCCACTAATCTGAAAAATTCATGTTCC
TCTAAATTTTCAAATCCTGTTAAGAATTCTGTATTGCCAAAGAATTGCAAGTAATGTGGCAG
TACTATCTCTTCATCTCCTCTTTTAGACTTGACAAGTATTTCTTTTTCTGGATTTTCTTCACAA
GATCCAGAGTAAATTATACGGGGTTTCCATGTTTTCAGTTTTACCCTATAAAAATCTTCTTTT
TCTATATTGTTTGATAACAATGAGCACGGCACAATCAACAACTCCTCTAATCCGTTTTCGTCT
TCCATTATTTTATCAAGAGATTTAGGAACTTCTACAAAATCTGGACAATGCTCTATGAGCATT
TCAGATAGCCTTTCGTCGGATATTGCTTCTTCTGTGTGATCTTTCACACGTTCGATCCCTTTGT
TGAATAATGATGTATCTTTTAATTCCATGATCCCCTTAGTCATAATTTCTGAAATTATAGAAT
CTTTAAAACTCTTTCTTTTAATTTTTTCTTGATCAAGTTCTTTGATTTCTTCTTTTCCATTTATA
GTTAAGTAAATGTAACCTGGCGATTCTATAGAAGGCATCACTATCCTCATTTCTTTATCCTTG
ACAATATTTATAAATTCTTCACCCAAATATTTCATTATTGAGTTCAACCTCAATATGATCTTT

CTTTCAAACACTATTAAATCATTAATCATTTTCATTAAATTTTCTTTTGTGCTGCTGTAATGAG
AAATATCTAACAGATAAACGTCGTGGTCTAAATACTTTGAATAAAAACTTTTTTCTGTCACAT
CCACTTTAGCTTTCACTTCTGTCATGCCTCTTATTCTATTTATTAAATTGAAACCATCATTTCT
TGAGACAGATCTATGTTGTATTTTTAACTCATTCAGTGCTAAATGTGAATCACCTCTCCCAGC
GAACAGATCTATTACTTTGTCATTCTCCTTTATGAATCCTGATGATTTCAACAGAGCTAGCAG
AGATATCATTGAAGGATATACATCTGATAAAGTAGGTGATGAAACATTCTTTGGTTTACAAT
TCATGCTGCATATCATCTCAAAACTCTTTACAGCTGATTTCAACATTTTAGTGTCTTCAAACT
CCTCCTTCCTGAATGGTAAAGATGAATAATAAACTACACCTACTTCGTGGTCAAAATCAGCC
TTCACATATGATTTAATCTCTTCAGAATAATAATTGTTCAATTGATATTGTCTAAATTCTTTCC
CATTTAATTCTTGATGGATTGACTTTGCACATGTGATGAGTTCAGGCACAGTAAAACCTGCT
GAAGGAAGAGCCATTAGCAACATTTTAGATATTCTTTTATCACGAATATGTAGGTGAGCTTT
GTCAGCTTTGATTCGTTTTAAATTTGTTATTGAATTTGAGACGGTCTTTTCCTCATTTATTTGC
AATATGAAACTACAACCTTTGATGTGTGTTAACCTATATGACAAAGATGTTGCTACTATAAA
TAATCTCATTAAGATTTCTCTATACTCTAGAGAATAGCCTTGCCTTATTATTTCTGATGACCA
GACTTTACCAGTGGACTTCGTTCTTTTAAATAACTCATCATACAACAAGCTTCTGATAACACT
CCTAGTTGGAGTCTCATTTTCTATTATATTCAAGCTTGTATAGAATTTGTAAAATTCATCAAA
ATAATGATCTTTATTAGTGAAATCCCTGATTTCTGGTGCTATTTTTTCTAGTATATCCATTGGG
ATCAACTCAGGGGATATTATGCATTGATCTATCATGTATTTATGCACTGATCTGAAAGAGTTT
TGTCTCATCATCAATTCGTGAGGCACCGCGTCTGCATTATTTATAAAATGTCCCTCGAGTTCT
TCACCTGTCGATAAAAAATAAGAGAGGTTCTTATATATTTTACTTTTGGATTTTATTTTATGT
AAATTGTTGATCTTTTCTCCTTTGTTATAAAAGTTTATTATTAATTCTGCGGGTTTCAACTTTA
GGTTAAATGAAACCTCCTTTATAAAGGGCCATATGTCAGAACAAATTGTTACTAATTTGCTTT
CATGATTCTTTATGTTACAATAAGATACTATGCTCTTGATTACTAAAATTAAATATTGTATAT
TAATATTGTTGTCTTCACCACCTGTTAGATCAATTAATTTGTCTATCCCAGCCACTTCAACCA
GATTGGTTCCTTCGTTTGAAGACAAGTCTCCTGTTCTTTCTCTGAAACCAGAACTTTTTGCTCT
GTGAAAATATCTTCCTCCTCTTGGGGAAACAACATGTTCAGTTAAGTCGTTGAACTTTGCATC
TGAAAAAGTGCTCAGTGTCAAATCACATATGGCTTTTAATGAATCAGCAGTATTATTGCTGA
GCTCAGAGTATTTCTCTAATTCCATCATAATCCACTTTGTATATCTGACTAGTTCATAGGCCA
TGAATATCAATTTATTTTGAAACATTCCTTCTATTTCTAAGTCTCTATCAAACTTTGCTGAGTT
GATATATGTTTTTATAGGTGGACTCTTATTTTTGCCTCTGTTATTATTGCTAGTTCGACTTATA
GCTCTCAATTGCCCGTTTACTTCATCTACTTCCAATTTGCCAAGAATAGGTATCTCTTCAATA
TCTAAGAAATCAATGATCAACTCTTTTTCTTCTGCACCTAATTTGTATGTCTTTCTCAAATTGT
TTCTTGCTTCTATGAGTGATTTATAACCTAAAGAACGTGGTGACCCCCAATTAATGTTTGTTT
GGTATTCGTAATTTATCATATGATTCTTTGTCCACGCCTGATTCCACAATTTGTTCATCTTATA
TTTGCCTAGGACGAATGAAAATGTGGTGGAATTGTCTATCTTTGAATAGATGTTTTCAAAGA
AACAAAAAATAGAAGATTCATAAAACTTGCCTACTATTCTGTATGAAAAGCTTTTCTTGAAA
CTTGTTACTAATTCTGCTTTATATTTTTCTTCGTCAATTTGTTTTGTAGCTTCGAATATAGCCA
GCAACTCTTCATTTTCTATTTTTTGTTCTAAATTTTTCTTCAAAAAATCTTTTAACAATGTCCC
TGTGTCAAATCTTCCTCCCGATAACGGCAGTGGGTCTGTAATATACTGATAAGCATTAGTGG
AAAGCCTAATTAGAGAATAAAATCTTACTTTGTCCATTGGATTTGCTATTATACTATCAATAA

ATGCTATTCTCTTGACACTAGAAATGGAGTAACCTGAAAGGGAAGCATCGATAATAGGAGT
ACACGCAAATCCAAACATTGCATAAGGAGCATACATCAGAATTAAAGTATTAGATAATACTT
TTATATCATCTTTGTGAAACAAATAAAAACCATTTATCACATCTTCTTCATCTGGAACCTCCT
TGCATGTCAATCCTTCAGATTCCTGGAGAATGTTGATGGCTTCTTTTAAGCTATCTAACTCTA
GTTTTGCAGATTGCTTTATAGCTGATTCGAGCAATTTTTGTCTAGGATCTTTCTCAAGTCCAA
AAAAATCAACAAATCTCATTATTTCTTTTTTCAACAACATTGTAAATTTATAATTCCTTAATA
TGGTCTGGACTTTGTGATGACTGGATCGTGCACAAGATGATGTATATCCACTGTCTATTATTT
TTATCTGTTCTAATTCATCTCCCCATATGTATGATGAATTAGGGGAAATACCCATCATTCTTT
TATAATTATTTTCTATGGGTGTATCCTTGAAATAATGATTTTTTAGCATAGAACATCTTCCAT
TTGTCACTTGTGTTTGTGATAGTTTTACTAGCAATCCCATTCTTCTTGAATATGTTTTTAAGTG
CAATATAATGTCATTTAATTTTTCTTGTGTCATTGATTCTTCTATAAATACACCACATGAATC
ATCACTGTAGGTTGTGCACTTAAATGAAGAAAATTTTAAGTCTCTTAGCATGTCTTCCATCAT
CAATTGAGATTGAATTCCCCATAGAGGACCCAACCACCCTTCTATTGCTCCCTTTTGATGTTT
AGAGTAATAGACATTGTCTGAATACAAATTCTCAAAAATGACTTCCATTGTATTGAAAATAT
CAAGAACTTGGGTCACATGCCTCCTTTCTTCACCTTCAAAACCATACATATCACAGATTTCTT
CTAAAATAAATAGATTATTTTCTGGCCGTTGTGAAGTGTTATGTCCTGAGTAGTCTAGAAAA
ATGGAATAAGAATCCTTTTCTTCCAACATCATTGACATTTCATACATCACATTGCGTCTCTGG
TCTTCGGTCATGGTCATCATCTGGCCTGGTATCAACTTCATTGCTCTCTTGACAAGTTCCATA
ATAGTGGATATGTACAATTTGATCTTAAAAGAGGCTACTCCGTAGAACCTAGCAGCTGTTTT
TAATTCTTTTTCTTTCTCAACTAATCTAACGATAGTGTATTTTTTCATCTTTGTTAAACTTTGG
TCTTTACGATCGACAATCTTGGTTAACCTTTGAGCTGTTGTTGAATTTTCAATCTTTACATTTC
TTACCAGTTCAGTAAGGTTATTGTTCTTAATGAAATAAATTAATTCTTTGACTGAATTGCCAG
ATGAGTCATACTCATCCTTAGTACAAGCTTTGTCAATTATTCTTGAAATGATATTAGTTTCTT
GGCCCAGCTCAAGCATCTTCCCTAATCTTACACCCCTATAATTATTTTCATTAAATGTTATTT
CTTTTGAGTAACCTCCTCCTGCTGCCATTAAGGACAATTCTTGCACCAAGTCATCTGGACAAC
AAAGTAATTTAGGTACTATTCCATATTTTTTGATATATGTAGTTATAACTTTCATTTTGAACA
TGTCTCTCAAGTGTTCTACCAAATATGGGTCAACATCGTGGTTTCTATTTGTTCTTTGAGTGT
ATTTCGACAGACCTTTATTTTCATCAACTTCTGCATAAACTATCGTTTTGCAAATGGATGACA
TCTTTATACACATTTGAGGTTCAGACATCATGCATATTTCTACAAGTTTTTTGGTTTTATAAG
GAGATTCTAATGTTGAGTCATATAGATTATCAAGTAAGGGCAAGATTGAAGAATTCATTTTG
TAGTCAGCCATCAATAAACAAGTTGTCTCGTAGAATGCCGTCATGTTTGTCCTCTTAGGATTT
TTAAGCTCCAACAATGATTTCACGTACATTATGAAGTCTTGTTCTCTTTCCCTTTCATTTTGTT
CTAGGTCATAGTTCAATTCACTTATCCAGTCTAGTGTGTTAGAATTTTCTGCAACCGTTAACA
AATAATTCAACATATTTCCATCACCAACCAAGATTTGTTTGTCATAAATAATCGATGTAATTG
TGCTGGACACAAATATGTAAATCTCTTCTATTTGTGTTTCTTTCCCAACATCTTTGTGACTAC
GTATTTCTAATTTTGTTACAGAACTTCCGTCTTTATTTGAAGTTGTGTTGATCCTTCTATTTTC
ATGAAAATTTACTGAAATTGATGAATCCTTCTTTTTGTTTAATTTGGAATAAGATATTTTTAT
TTTCAAATTGATTGTTTCAAGCACAGACAAAATCCAAGAACTTTTAAGTGTGTTCATTTTGGG
TTCTACTTTGAAATTGTTTTTAAAAGGGTAATTACCAGTTGTTCTATCACCTGTCATCATATTT
CTCATTTTTGATGCTTCATAATTCTCTAAGTTAGAAGCAAAATTGATGAAAGTCGTGTAATGT

GGAGATGCGTGAGCAACTTCTCTCATCTTTAAGTAAAACTCAATAATTGAGTCCTGACTGGA
CATAATCCTTTTCGCATCCTCACCAAATTCTGATACAAATCTTCTAAATTGCATTAGGTCTAT
CTTATTCTTTGTGTTGAATTTTTCAGGATACAATTTTGAACTCACAAACAATTGAAGGTTTCT
TTCTGAAAAACTTCTGTACGGAGAACATATTTTTGAGATTAATGTGGGTTTATCCACATAATC
TTTTTCCCTCTGGCTCGGTATCCATTCTACTAAATGATTGAATTTTTGTTCAAAACCCACATCT
GTCAAGTCAGGTTCCTCTTTCTTTTCGGTTGCTCTGCTTTCTAGCATTTGAAATAGTTCATCAA
AGTTTTCCACGAATATTTTGTAAAGATGTGAGTCTTCCATCCTGTACTGTTTCATTTTGTTGA
ACAATTCAAGTACAGATTTTTCTGTGCTCGTCGTTTTATCTGATATTGATTTCATAGATATCC
ATCTATCCACATAGTATTTGTCTTGCAGAAGTGATTCTTCTATGTCAGCCCAAATCATTTTTG
GAGCAGTCATGTTTTTGTGGTCGTTAGAAAAGTACGGTGTTATTTTCATTAAAGCGATTTTAT
GAAACTGAATCAATTGAAAATGCAAGTTTAATTTTATTCTCATTACAACAAATGTAATGAAT
TGATTTATTGATCTCGATTTGAGAAGGACTCAATTTAATAAGACTTGATAAATACGGAGAAT
TGAATGAGGCTAAGGATAATTTGCAACAGCTCAAAGTTGATATTAATTTAAAATAAATTGAA
AAGAAGATGAAATTTAGCTTAATTTCGGTCATTAAGCTAATCTATGGTGATAGAAGTAAGAC
AATCAAAGTATTCAGATTCTTGACTGCTTGTGTCATCTTCTTCACGCTCAATTTCAACTATTG
AATTTCCACTAATGCAAAATTCTTCCTTATTTGATGTCATCTTGTGAAATATTGATTCTGTCTT
TCTTAATATTTCGCAATGAGACTTCTTCCTAAAGAAGTCTTTCTCATAACCTGAAAAAGAATT
GTCGAATAGTTTAAACAAAATGCTATCTTTATCTCTACAACTTATGAATATGGCATCACAGCT
TAAATTTGGAGAAAGTTCATTGATTATTAGATCATCAAGAGAATTGCACAGGAATATTTCAC
ATTTTTCAATGACAAATGATGTGAAGAATGGTTCTGAGTTTGAGTAAACAACGTAGGCTTTC
ATCCTTACATCCATTACTTGTTTAGTTTCATCTGGTATTAAGTTGCTGATGATTTGATGACCC
ATCACTCCTTTGTCACTCCAGTCTTCTTTTGACAAGAAGCTATTTTCTACGATTCTGAATGATT
CAAAGATCATTTTATCATTTAAGACCCCCATTCTTTCAAGTTCCTGTTTAGCTATTCGTGAGT
TTACTCTTGTTAACTTGCAAGCCATGATTTTTAATATCAAATATCGATT

>Seq38 [organism=Datura ophiovirus] RNA2 genomic sequence

ATACGTAGACTGATCTTTCGATAATTTTAAATCGATTGTATATAATACAATTTGCGTGTGTGC
TACACGGCTTTTAGAATTTGGGTGCATGCCAGTTAAAGTCTGCATACTCGACTGGAACCTAT
TAAGATTCACATATCTTTTATTTAAAACGATACTTGTTCACCCCAAATTACATGATGCTAAAT
ATTTCAACAACTACTCATACTTATGCTTCATTAATTCATACTAAAAGTACTTCGTTTTAACTT
AATAATACTACCATAATCCAACACTTATCAGAGTTTATCATTGTTGTAATACTCTGTCGCAGC
TTCAACAGCATAGCAGAGACCCCCTTCAAATTCATGACTATATTTCAACTTATAATTATTGAG
AAAATCAGAAACAAACTCTTTTGAAACAAAGTAATTTTCATCCTGGATCCACATCCTACCAG
ATTCGTTTTCATCACATTTTAAGATAATCTTATTATCCAACATTAGATAACAAGTGCCATCCT
CAGATTTCATTCTATTAATCACACCATCTTCCTTCAGTTTAGAATAAGAAACTATGTTTTCCT
TCATACATTGTTCATTGAATAAACCACAATTTTTCATTGAAATTTCTCTCTCATGATATTTGTA
AAGAATTGTTCCTTCTTCTATCATCAGCTTTTCAACGCCTCCACAATAATCAATTGATTTATT
AGGCATAGCATCACCATGAAAGAAAAGATGAGTGTGCGATGCAGTGTCTAAACAAGTCAAC
CTCTTATCTCCTTCAGGTCTCTCAAATGTTCTGCTACTTTTAGTGAACATAGCATTCTTATTAT
TTTGTTTCACTGACATCAATTCAGATATGCTTTTCCTGCCAGCATCAATGATGTTATTAATGT
TGCTATCATCAGAACTTGATGATTCCTGGATGTATTCTATTTTTTTGCAATTCTTCTTTCTCTC

TCGTGCCCTTAATGTTTCCAATCTTCTAAACTCTTTCTTTTTCTCCTCAAGTTCTTTCTTCACAT
CCGAACTGATTTTAGAGAACATTGTTTTTGATTTATGTCCTTGGGTTTCATCATCGGGTCAA
GATATATCAATTTAGGTACACCTTTCTTTGTCTTTATGGGTATTTCCTCTTCATGAGTGAAAA
AGAACATGTTAATCGCACCATATGATCTGCCCTCTATAGGGATGCTTTCAACCGCCATCTTCA
CGAGAATGTTGTTTAGGTCTTTTGCAGCAACGAAGAAGTCCATTGAGAGTTCTATTAAAGCC
ATCTCATGACTTTCAAAGCTCACTTTTTCAATCACAGGATCCAGTCCAGCGTTTTCTTTACCC
ATGTCTTCAAGAGATATAGTTATCACACCAGATTGACCATCACTCTCATTTCTTTGAAATAAT
GGTATGTAGAGAATCTGAACTTTTTCGAACCGCACAAAGTTTCTACTCGGCATCACTTTGTTG
AAGAGACTTTTCAAACCACCAATCACAAGTCTTTTGGTGTGTTCCTCTTTCTTCAATTTCATA
GTGATGGAAGATACAGCTGTTTTGATTATGCCTTTTTGCACATTAGCGTAGTCCATGATAACA
TCTTCTATAGATGGATCAATCTCAGTCATATCAGAACTTAGGTGTATTATGTTCATTTTACCA
TTGTAGAACCCTTCATTCAGCATATCTGACGCATGAGCTCGAGATATGCAGACTTCTTTTCCT
CTCCTTGAAATTTGCCTATCCATAGAGTCACAATTCCTGACAGATATTGCTCTGATTACAGAT
TACAAATACGTAGACTGATCTTTC

>Seq39 [organism=Datura ophiovirus] RNA3 genomic sequence

TGTGCTGGTTGTGACCCTTTATTTAAAACGAAAGGACACGTAATCTGTAGATCACAAACATG
CGCGCCACAACTTATTTATTCAAAGATCCCTTTTAGCATAGCAACAGACATGTCGCTGTAGT
CTGCAGTGTCATCATTAAGAACTTCGTGAACAACTTTATTGCCATGTGTTTCACCGAAAAAA
TCTTTATCTCGCATAAAACCAGAGTTGTTGTCGGAAAGTATCTTTTTATGAAGATCCTTTCGT
CCTTCTACACTGAGCGTCTCAAGTATCGCTCTGGTGAGTTTTTTCGTGAAGTTTTGAACTACT
GGCCTTTTCGGATGAAGAGGATGCATTTTGATTTGTTTTTTGACATCTCCTTGTAAAGACTTT
ATGTACTCGACAAGGTTGGATGCATGTTCCAATCTTTTGTTTCGATCCATTATCATCATCAAA
ACTTCTGCTGAGTCATTTTTAGTTGGTTTCAGGGGCTCTGCCTTACGGAACCCAGATGCACAT
ACAGCGTATCTAACTGCTCTGTTGCCTGCTATAGCCAATTTGCATCTTGATGCAATTGCCACG
GGAAGTATGTTCAAGTCAATATTGAGGAAGACATTCTGAGGAAATTTCCTCGTGCTGGTGCG
TGAAAGGTCTCCTGCTATGCTGCCCATTGTCACTCTGTTGGAATTGTAAACATAATCTTTGAT
GAACTTCGGCACAGTTTTATTCTCAGCAGCATTCGGCAATGATCCTTGTACATAAACCAAGA
TCATTCCGGCAAGCAATGCTTGTTTGCCAAGCATTATATTTCTGCTCACATCAGTCGAAGTCA
ACTTTTCTATTTTCATCATTCTGAAGATTCCAACTGGTTCTTTGTCAGGCTCAATCATAGAGC
AAAACACAATTGCATCAAGAATCTTCGTGCCTGCTCCCACCAATTCAGAAACACTGCAATCA
GGGCTCTTATAGTCTGTATCCTTGAAATATTTGATCACTAAGTCATTCTCCTGATAAGTTTCA
GATTCAGTGCCGATAGTCTTGATGAACTTCTCAAGATGCTCTTGAATTCTTTTAGCATCAACA
ATGAACGATGATTTTTCCAGAGCTTTCTCAAATTCATCATGATTTGTTATTTTCATTTCTTCTT
TGTCCTCAAGATCTTCAAATGACTCAGAACCTTCTTCTTCTGATTCATCATCTTTCAGAGTTTT
TTCTGGTTCTGTCACTTCTTTGCTTGTGTGGCCCTTGCTTGAAGAACCAGTCTTCCTCAGTTCA
GCCAGAACACTGTCAGCATCTTCACCTAGAGAGAGTTGAAGATGGCTTTGTTCTTTTGCAGA
AATCCTACCTGTAAGAACACTTTTCCTACTTGGGGCTGAAGTGGTTTTTGTCCAATTTGTTGT
CTTTTGCTCATACGGACAAATCTGACCATCAATCACAGAAAACATCTTTTTGTCATCTAGACA
TTTCTTCAGACGGAACATGTTAGTTGTTATTTGTTCACAGTTGGGACCTATTCCTGCAGCATC
GAGTAATGCTTCTTCTTCAGGTGTAGGTGTGGCACCCTTGTTGTTCACCCTGTCAGCAAGGGG

GAATATTTCCTCAGGATTGAAAACTCTGTCTTTTCCAGCCATTATGGTTAAACGTAATCTCGG
TTCGTAATTTGTCGAATTACAGATTTTGAGTGGCAGATCACAGACAGCAGTGCATGCTCATA
CGGAC
>Seq40 [organism=Epifagus ophiovirus] RNA3 partial sequence
AGCAACCATCTTACATCATATGATAGCCTAACATCCATAAATGGCAAACTTGAGTGGGATTA
CGCTTCCGAGTTTCTACACGCCTGCGATTTCTTCCCCAAACGGAACCACAATACACAATGAA
AATGGAAACAAGGTACACGACGACACGGAGTCCCAATTTTTCTTCCTTCTTTGATTAGGGCG
GACGGAAGTTCTACCTTCTTCAAACAGCGGACGGAGAAGAAGCACGTCGTCTAAAAAAGAG
AGAGGAGGGATATGGACTATAAACTAATATACCCAATTTTTATTTAAAGCGAAATGCAATA
CACTAATGAACCATGAAACGCAGGCAATAGAGGTTTTCCTAATTTTAATACATAATGATTCA
GCAGCACATTTTTAGGTCACCTTATAGATCTCCTTCAATTGACTCACTGTGATATCATTATAG
TCTGCATCATCTCTTCCTANAAATTGGCCAAACCCTCTCCTCGTGTTCGCCTCAATCACACCA
TAAAAGTTTACATCATTCTTAAACGCTTTCACATTATCAGTATCCACTTTCAATATCATCGAC
TTTCTACCTTCTGAGCTTAACGATTCCAAAACTGCCCTTGTGAGTTTAAGAGTCAAATTCTCG
ATTGTCGGCTTCTCAGGAGGACAAAGCGGGTGCATATTGATCTGAGCAGCACAGTTTCCTTT
TAAACTTCTCAGAAATTCTGCTATTTCTCTTGTTCTTTTCATTTTATCATTACCAACATGATCA
GGATCAGCAACTGCTTCAAAAAACCCAGCAAAGACAGCATATCTTATTGCCTCATTACCAGC
AATATATAACTTGCATCTTGTAGAAACTGGTGTTGGAAAACTATCCGGAAATTTTGATGTAG
GCGCTGAACTTAATTGCTCACCAATAGATATCATACTTTCCACATTTCCTTTGTATATGAAAT
TCTTCACAAATTTAGGAACAGGGCTTGCATTAGGAACAGGGCTTGCATAAGTACCTCTGTTT
GGGGGGAGTCCTCCCTCCATATAACAAAACACAATTGCAGCTTGGATTGCTCTTGCCGCATC
AACGGAATTTTTGGAAACGGTGCTTGCTGCGATCTCTGTTATAACAGACACATCAAAAATTA
TTTCTGCTTCAACAGTGTCTTCTGTTGATGCGTATAGAATCGCATCAAGAACTTTCGTCCCAG
CTGCTATTAATCTTTGTACATCTCTGGTCACTGTTCTGAAGCATTTGATTTTANNATCTTCACC
AGGATAATAGTCAACGTATGATGATTGCTTCAGCAATTCATTAAACTCTTCTTCATTTTCCAT
CGCTTTCTCAGTCACAGCTGGAACGATTGCTATTGCTCCTCCACCTTGTTGGGAGTCATATGA
ACTAGCAACGGCCTCCTCGGTCCTCGCT
>Seq41 [organism=Erigeron ophiovirus] RNA3 genomic sequence
CCTTGGTCTATAACATTTCAATACTCCAACAGTTTTTCCAGATTAAATTTCTTGCAGGTAGT
TAATTGAATCTGTTGTTGAAAAATACCTGTTTAAATAGGAAATCTTAAAATACATTTTAAAA
TAATAAAAAAGGTAAAGAAAACGGGATACAACAACGCCGTCATATAAAAGACCATAACATC
ATAAACCTGCAAACACACCTTAACAATAACCTACCGTAACCGAAATCAAGAAACCCCATAG
ACAATCTTAAGTGTAAGAACAGATAGCTCAGCAAAATCCGCTTCAGCATTGCTCAAAACATC
CCAATATCTTACTCCATTCCTGACAACACCGTACAGATTTTCATCCTTCTTGAATGCTTCCCT
CTTGTCTCTGTCGATTTTTGCAACCATTTCTTGTCTCCCAGCCGGGCTCAACGTGTGCAAAAT
GGCATTAGTCAATTTCAGAGAAAAATTCTTAATTGTGGGTCTATTTGGAGACTGTGGATGCA
CTCTCAACTGAGCTTTCATATTCCCTATCAAAGTTGCCAAAAAGTCGGTTATTCCTTTTGCCA
GTTTCAACCTGTTGTTATCCTCAAATTTTTTCGTTACAGTAGCCAGATCATCTCCATTTTTGAT
CTCATTCTCTTGCCTTTTTTCACAGCTTGCTACATAACGAGCATATCTTACTGCTTTATTCCCG
GACACATATAACTTACACCTGGTTGCAAGTGGCACAGGAAGTATGTTTACATCAATGTTCAA

CAACACTTTTGCTGGAAACTTACTTGTTGGCGCAACAGATAAATTTTTTCCAATACTTGTCAA
GCTATCGATGGGTTCCCCGGTGTAGATTTCCTTGTTCAAAAAAAGAGGAACGCTTCGTTTAT
CTTCACCTGATCTTGATGGAAGATAGCCTTGGACAAACAAAAGTACAATCGCAGATAGAAA
TGCTCGTTTAGACTTGACAATATTTTGCGCAACCTCTTCGTTTGTCAAATCAGTAACTTGCAC
AAACGAAAAAATCCTTCTTGAACTTTTATCGTTACCTAACAAGCTATACAAAATGGCATCAA
GTACTTTTGTTCCTGCAGATACCAACCTGATCACATCAGGAACCAACTCTCCAGAATACTTTA
TTTCCACCTCACCAGCTTTATAAAATTCTTCCTCTTGTCTTAAACTCTTGAAAAACTTTTCCAA
GTGCTTAATGATTTTTTCCTCATCAATTACGTAAGAGTTCTCGTTGATGATTCTTTCTAGTTCT
TCTACCCTTGTTATTTTCACCTCCCCCGTGCTCTGACTTTGACGGGATTGAGTATTGTTGTTGT
TGCTTGTTATTTTCATTCCGTTAACTTCGTACTTCTTGCCAATGTCATTATATTCCCCTTCAGT
CTCAGAATCTTGCTTTTCTTCATGTTCTTTATTCATTATTTGATTTTGAGGATGATATGAGATA
GAAAAAGAGATGTGTATATGTTTGTAGGTTGAATCTGTTTGTGGACCACTAGATTTTCTATCT
TTAAATAAAGGAGTGTTGTGGGGGGATGGTTTCAATACTTCAACTTCCAAGTTCCTAATTTCC
AGTTTCAATATTTCAACTTCCAAGTTCCTCATTTCCAATATCAAAAAAAGCATTCAATTACAA
TAAACTTTAGAATAACAAAAAGGAGTATTATTTAGCTCACACGTAAAATTTACTCTTCCTAT
ATAAAGTTTATATTATTTATTACTTTAAAATATCTACTAAAGATATATATATACAAAACAACT
GTATATATGTTTATTTTCAATGCTAAACATAAGGGTAGTGCTATTCATTCATATAACAAAAGT
TAGCCGTATGCAGGTG
>Seq42 [organism=Erythranthe ophiovirus] RNA1 genomic sequence
AACATATTTTGGTCTTTCTATTCTCTAGAAAAGGTCGCAATGACCGTAGAAGTGAACTAATT
CTCCTGTCAATTTGAACTACTCATCACAGTAATCATCTTCATCCATTGCCTCATACCATGCAT
CTTCTCCTGCTTCTTCAATTGCCATGTCCACAGTAGTAAGTTGGTTTTTCTAATGTCCTCTTC
AGTCACGTCTAAAAATCCTATAAAGTCAATTCTCTTCTCAAAGTCTTCAACATCAATTTGAAT
TGTTTCCAGTGATATATCCTTGTTTAATCTCTCTATCCCTGATGAAAGAATAGAAAATATGTC
AGAATCATTATCTTCTTCTAAGTATGACATTATATCTTTGTATGATTTTATTTTTTCTTCAAGT
TCTTTTCTTTCTATCTCATCCATACTTTTCAAATGTTTGCTCCACAAATTTTCATACTCTTTCCT
TGGTATTCCTGCCCAACAAACTGGATCAGATATTATTTTACTTAAATACAATATTCTGTTCAT
AATGAGTTTGTAATTTAAAATGTTATACTTTGTTGTTTTGTCTTTGTGTTTGAGTATTGTGCCT
CTTCTTAAAGAATTTATTTCTCCTGCTCTTAGTAGTCCCATTTCTACTCTGTTAGTCTTGAAAG
CTAATACTGCTTGAGATGCTATCTCATATATATGATTTATTGACCTATTTTTCACGTTATTTAT
TTTTTGTACATTAATTAATTCTGATAGAATTTCTGTGTTTATTTCACTTTCTTCTTTTACCACTT
CTAACATTAGTTTCCAACATTCTTTAGTTACGTTGTTGTTGAAGTTGCCTTCTAAGACATTTTT
TGTCAGAATAACAAAATCATCAATTTTCATTTTTTCTGCCCCCCTTATCATTACTAAGTTATC
CTTCATAAATCTTTGATGAACAATGATGCTTTCTTCTTTGTCATCCCTCCCATCTAGTTTCCCA
ATCATGTACATACTGTTTGCACCTTCATCTCCTTTTGATAATATATTTTTGATTGGAAATAGC
AAATTCTTCCTAAATGACTGAACAGTTGAATTATCTATATAACAATACAAATGTGACTTGAA
ATCGTCATAAGATTTTATTTCATGTTTGAGATCAGGATGTACATGAACGTAATCAGGATCTTC
ATCTATCATCATTTCTAAAAGAGTATTATCTGAAATTATTTCATGAGTATGATCTTTTGTTTTC
AATCCTGACTTACTGATTACTTCAGATATTTTTGTTTTTCCAATACTGTTTGTTAATGCAGAA
GATAATAAAGACTTGTGATATCCTCTTACATCGCTCTCATTCTTTTCTTCCACCTCTTTTCTGA

AATCTATTGTTAAGAAGATGTACCCAGGTGAATTATTATCAGGTATGTAAATCCTTTTCTCAT
AACCTGATATTTCTGCTAAAACACCATCATTAAAAAATTTGAATAGCCCATTCATTCTCAGTA
TTACTCTCTTTTTATTGATTTTTAAATCTCCTATTATGTTACTTAGACAATCAGTCTTATTTGT
GATATGAGAAATGTCCATTAAAATAATATCATGGTCAAAATATTGAATGTAATCTTCTCTTTT
GAAACAGTTGAAACTAACTTTCTTGCTTTTCATTCCGGGTATCCTTTGAATCAAATTATATCC
ATCTTCCCTGGAAAGTGTCAAATGATTTATGTTTTCTTCCATCATAGCTAAATGAAAATCTCC
TCTACCTCCACACAATTCAACAATTCTATCATTAGACTCTATAAAGTTATTGCTCTTCAATTC
TGTTATCAAGCCTTTAGCACTAGGATATACAGAAGACATTGTAGGAGAAGAAATATTTTTTG
GGTTGACACTTAGCTGGCAGCACATTTCAAATCCTTTTATTGCTGAATTTATACCCAGTTTGT
CATCTAAGTCTATTGATTCGATCAGTATGGAGTTGTAATTTACTTTGCCATATATATATGTTT
CATTTTTTTCTATTTCGTTTAAAAATAGTTTCATATAATAATCACCCATTCTGAATTGTTCAAA
TACTTTTCCGTCTATTTCATCATGAATTTCATCACTTGCATTACAAATTTCATCATAACTATAC
CCCAATAATGGAAATGAATTTAAAATTATTTCTGTTATCCTCTTATCCTTGATATGAAAATGA
CACTCATTTTCTCTTAACATAGAATAATTTTTAACTGCATTTTGAGATGTCCTTTTCCTATTTA
TATTTAGTTTTATAAATCCATTACCTATATCTTCTAATCTATATATCATTGATGTCGCTATTAT
GAACAATTTTATTAAGGCTCTTTTATACATTAGAGAAAATCCTTTAATAGCTATTTCACTTAC
CCACATTTTTGCTTTATCTTGACTATTTATTTTGAATAATTCCTCATAAAGCATACTCCTTACT
ACAGCTCTTGAAGGAGTCTCTTTTCCTATAATATTTGTTGATTTGTAATACTTGTAAAATAAA
TCAAAGAACGAATCCCTATTTACATTTAATTCTTCACAAGGAACTAATTGATTGAAGATCTC
ATCAGAAATCAGATCAGGACTTACCATCATTTGATCTTCCATATAAGAAAAGACTTTTCGAA
ACGAAGACTCCATTGTAATCATTTTTTCTTTCATCGTGTGTACATGATTTACAAATTTTCCTTT
TAAATCTTCATCTGTGGATATGAAACTTGAATAATTGTAATATAACTTACCTCTACTCTTTAT
TCTTTCTTTAGTGGCAAAATTCATTCTAACTATATTGTCCAATCTCCCAATTGGATTTTCTAAT
CTTGTTAGTTTAAAGGAAACATCTCTTAATTCACCCATAATGTCATCAGTAACTCCTAGTGGT
ATTAACTCATTAGGACTGGGTCTTAATATTCCTAAAACTGTTTTTACATATATCATCAAATAT
TGCAAATTTAGGTTGTTGTCTGCTCCACCAGATGCTGTAAGTAATTGATCAACACCTGCTATG
TCATATCTGGATGAAACTTGATTTGAAGATAAGTCTCCTGTTTTTGGATTGAAACCAGCTGTA
AGTGCTCTGTGAAAATACCTACCACCTTTTGGACAAACAACGTTTTCTTCAATATCCTTGTAT
TTTGCATCTGAAAAGGTGCTTAATGTTTTATCACATGTCATTATTAGAATTTTCTCATCTTCTT
CATCCATTCCACCTGAAAATTTTTCTTGTTCCATTATAAGCCACTTTGTGTATCTAACCAAGT
CATATGCTTTGAATATAAGTTTATGTTCAAACATTCCTTCTATTCCCATTTCTCTATCAAATTT
AACTACATTAATAAAAGTTTTTGCAGGAGGATTATTCTTTCTTCCTTTTTCGGTCAAATTAGT
TGGTCCTTTATAAACAGGTTGTATTAAGTTCTGATATGGTAAATGCTTTATTTTTCCCATCAA
AGGGATCTCTTCTATTCCAAGGAACTTTATTTTTATTTCTTGATCTTTGTTTATTAATTTCTTTT
TCATATCTCTTTCCATTATAAGATCACTGAAACTTATCACTGGCATTGTATTTTTCTTTACCTT
TAATTTATGATTTACACTCCAAGCTTTGTCAACTAATTCCATCATCTTTTTACCTCCTAAAAG
CATTTTCATTGTTGTTGCATTATCTACTTTGGAAATTATTTCGTTTATATAAGAGTATAGACTT
TCTTCATAAAACTTTGAAACAATTCTTGAAGAAAAACAATTTCTAAAAGTAAATACAAGTTC
TTTGCGAAATTCTTTTTCATTTTCATCATCACCCATGTTGATAAATCTTAACAATTCAGGGTTT
TTTACCATTCTTTTTAATGACTTTTTAATTTCATCCTTTGTAAATAATTTAGTATCTTTTCTTCC

TCCTACCAAGGGAAATGAATTATCAATGTAAGAGCAGGCATTTCCAGAAAACATAACTAAA
CTTCCAAAAATTCTTTTTCTTTCTCCTGTTAATAAACTTTGAATATAAATTATTCTTTTCACAT
TAGATAAGCTATATCCTGAAATTAAGCTATCTGGCATTGGGGTCATTGCATAACCGTAAGTA
GTGTATGTACCATAAAAAATTGAAATAAAAATTTCTTTTACAGGAAGGTTATTTACATGGTA
ATTAAAGAATTGAATTATGTCAGATCTTTTAGAAGGAATTAATATTTTTGAACTATTCTTATT
GTTTGAAAATTGCCTTAAAGACGACTTCATCATTTCTTTGTAGTTACTTAGAGTTTTGATATA
TCTAGGATCAAATTCTTTTTCTTCCATTAATTCAGAAGTTATTTTCATTATTGAACTGCCCACT
AATTTAACTCCCCGAAAATTTCTCAGCAGTGTTTGTATAGTAATATCTTTGGACCTTGAACAC
GCTGATGTGTAACCTGAGTCTATAAGGGTTGAACTTTCAAGTTGGTCCCCTAACATTTTAGG
ACCATTCGGTGAAATTGAAAAAAGTTTCTTGAAAGTCATATCAACAGGGCTTTGTTTGTAGT
AGTGTTCCTTTAACATTGAGCATCTTCCATTTGTTACTTGTGTTTGTGACAGTTTAACAAGTA
ATCCCATTTTTCTCCCGTAATCTTGAACATTTTTTATCAAATTATTCAACTTGTTTTCATCCAT
TTCTTTTTGAATAAAAACTCCACATGAATCATCAGAATATGTAGTTCCTATAAAATCCTTTAT
TCCCATTTGTGAAAACATGTCTTCTAGCATTAGTTGAGATTGTATTCCCCAAAGACTACCCAA
CCAGCCTTCTATAGCCCCATATTGCCCATGAGAATAGTAAACAAAATCACTCCAAGTATCCT
CATTATAAACATGGATGTTTGAAAAAAGATAGCAAATGGAAGTAATCTCCTCATATTCTTTG
TCACCTTCATAAAAACCATACATATTCGCAATTTCTTCCAATATAAATAGAGTATTTTCAGGC
CTTTGAGAAGTGTTATGACCAGAGTAATCCAAAAATAAAGAATAACAATCCTTATCTTCCAA
TTTTAAGGACATTTTATACATTATATCTCTTCTTTCATCATCAGTCATGGTCATCATTTGCCCA
GGCAAAAGCTTCATTGCTCTCTTTACCAACTCCATCATGCTACTTAGCCACAGTTTTAACTTG
AATGAGGCTATTCCAAAAAATCTTGCAGCTACCTTTTGTTCCTTTTCCTTTTCTGCAAGTCTA
ACTCCGTACTTTCTTGGAATATTTCTTAATTCATTCGCTTCTCTATGCAACACTTTAACAATCC
TATCATTGCTTTTATACTTACTTTTATCTATATATATAGTATCATCTTCCATTAGTGACATATC
ATTCTGTATGTAAAATTGAATTTCCTTTTCAGAATTTCCTTCTGGATCATATAATTCCTTTGTG
CATGCTTTATCTATAATCCTTGACTGAATATTCATTTCAATGCCAGGTTCTAAAAATTTCCGT
AGTTTTACTGTCTCATAATTGTTTATCTCGTTTACTATGCTTCCATTGTAATTTCCTTTTGCAG
CTTTTAGTTCCAAATGAGATATCAGATCTTCGGAAACCCCTGTTAGCTCTGGCACCCTTCCAT
GCTTTGAAATATAAGAATTGATCAATCTCATCTTAAGCAATGATCTTAACCTCTCAACAAGT
GGATTACTCACAGGATGATCCCTATTTGTTCTCATGGTATATTTTTCCAGCCCCCTTTTTGGAT
CAGTTTCCGCGATTATTAGTGTTTTTGCCAAACTGGACATTCTTATGCAAGTTTCACCATCTG
ATCTGTTGCATGCCATTAGGAGTTTTTCTGTCATTTCAATACTGATACTCATAGATTCCATGA
CATTGTCAAGAATAGGCATAGCAGCAGATGAATTCTTGTAATCTGACATTAATAGACAAGTT
GCTTCGAAAGTTCCTGCAAGACCCATTCTTTTTTCAGAACTCATACTTAGCAATTCTTTGTAG
TAATTTATGAACATTTCTTCACTATAAGTAGCATAAACATCAGAATAAACATCCATTGTTAA
CAAATTATCTGCAATAAAGACTGAATTTTGCAAAAGCTCAGGTCTTCCTACATGGACAACAA
CACTGTCAGTAAAGAAATATGCAGATATTGTTGATGACATAAATATGTATAATGAAGTTTCC
TCATTTTCGAACTCAACATGAGTTATCGATGCTCCTATACGGTAGTAAGAAGTGTTTATTCTT
TTCCCTTCATAAAAATCAACTGATTTAGAAAATTCTCTTCCCTTTTCTATTGCACTAGCGTTC
ATCATTATGTTCTTATTTATTGTTTCAAAGATATCTAAAATCCAATAGGTCCTCATTGTGTTTT
CAGACAAAGTAGTTTCTTTTTTCTTAGACATATCAGTTGATAACTCTTTGTCCAGAAACATTA

AGCTTAACTTTGACTTTTCCATGTTCATTATGGATTCTCCAAATTTCCAGAATATATTTGATTC
AGAAGAAGGTCTTGAATAGGATCTTAACTTAGAAACAAGAACATAAGAAGAATCTTGGGTA
GATTTGTATGTTTTAAAGAAAGCATCTTCTTTCCCAATAAAAAATTCCCTGAATATCATAAGG
TCTATTCTGTTTTTTTGTCTCCATTTCTTTGGATAGTCATTTCCTTTTAACATTTGAAGGTTTTC
AGGAGAGATGATCTTATGAGGAGAGTCTATCTTTGTTATTAGAACAGGTATGCCAACTTTTG
GGTCTTTCATCAAAGAAGTAGCATCAACTTTCTCTAAATCAACAATCATTTGAATATAGTCA
ACAGTGACTTCTTTTGCTTCCATTTCCTTTTCTTTTTTAATTGATTTTTCCAAGTCTTCAAAATC
CTTCTTATACCGAGAAAAAAGAAAGAAAATGTTAGGGTAGTTTGAAATATTGATAGAATTG
AAAGCCATTTTCTTGATATCAGTAGAAGACAATAATTAGTTTAAAGTATCATTAAAGGTTTT
ATTAAAACATAAAAAGATGAAAGAAAACAAGTGGAATCAAAAAACAGAAATTAAACTGAT
TTACTCCCTGTGATGATGTCACTTTCATGTCCTGACTTCCCAGACATTTTGTTCTTGTAGGTTA
TGATCATCTTGTTTATTTTCTTCCATCTTTTATTTATCTCATCATTCTGCACATTAGTAGTTAA
AATGTATTGTTTTGGGATCTTAATTGCACCAGGCCTGATTATCATTATCTCTTTAAGGACTGA
CTGAAAGACTATATCTGCTTTAGTTGCAATTATGGTATAGTCATTAACAGCAACTTGGATATT
ATTACAGATAAACACTTGATAGTCCTCAACATCTAGCATTTCATGAATTTCATTCTCTGATTC
TATTATGATGATACCTTTTGGTGACAATGGTTCTCCCATTATTTTATCAGAAACAATCCATGA
TCCAAAAAGAAATCTAGTAACAACTTTGTAAGGCAATTTGATACTCATAAGCCTTATGAGCC
TGCTTCCGTTTCTCATTTCTAGTTCTTCATCCAATTTCATAACAATGGGAGAATACTTCATTTC
AGTAAGGTAATTGAATGAAGAACATCTAACACATTCTTTTGAGTTGCCATGACCTGGAAAAA
TTGAGTCCATGTTTTTACAATAAGGAAAATCAGATGAGTTCTTGTAAAATTTGTTTATGTAAA
AAAC
>Seq43 [organism=Erythranthe ophiovirus] RNA2 genomic sequence
GCGGGTGATAATTTTCTTTAAATTATCCAGTTCCTCATGTGAATGTTAGCCCATTCGCTAGAC
TGATAGCCAGCTCCTGACACTGCCTACCACTGACCAAGATGATCAGATTCACTGAGAAAGGG
TGCAATACCCGTAGAAGTGAAAATAGTAATTTAATACTATTTCAGTGAAGAGTTTTATTAAA
ACATAAAAGCTAACCTATAATCAAGTCTTCTTTGATCATGCCTAGTGAAAACATCATCTTCCC
ATTTTCAATGTTCTCAAGGTGGAACATGACATTATCATCTTTCATTGCCATTGATTGCCCACT
TGAGTTTGTCTTGATACAGTTTATAATTCCAGATTCTACTAACCTTGAATAAGATATGATTGG
TCTAGCATATGAACTTCCACAGCATATATTTTTCAACCTTACCCAATAATTTCCCAACTTGAT
CTCTGTTACATTATTTATTATGAAATAATTGATTGAATTATTATGTGGACTCTCTTCTAATGTG
TTCATGTCTCCTTGAAAAGAAGGGTTGTAGATCCAATGATTTGTTGCACTAGTGTCTAGTAAT
ATTTCATTGCTAAGTACAAAAGGAATTGATCTAGGCAATAATTCTTTTATATCTTCTTTTGTT
AAGCAGTTTTCTTGCATTTCAGAACATGCACTGTTTTCAGACACTTTGTTTTCAGTGAAAATT
CCTTTTATTCTTTCACTTTCTTCTTGGTTATATTTAATCATTTTTATTTTCATCTGCTCTCGTTTT
TCTTTTACATCATCTTGTACTCTTTTACACAATGATTTAATAGATGATGTCTTATTAATATCGA
TTGGAGATTCGACATTGTCTATCATCAAGGTAATAGATTTTCTTGGTTGTATCATTGCAGGGC
TAAAATCTTTATGTATGTAAAAACCAATTAAGAGAGAAGCATAAGATCTTCCAACTATAGGT
ACATTAAATGCATTATAAGATAATTCAATAAATTTGATATCTTCAGACCTGGTAAAGAAAGA
CATTGACATTTCTGACATACTCATTGAATTTAATGGGACGCTAACTGATGAAAGTACCTGAC
TTTCTTTGGAAGCAATTGAAGTATCTCTTAAAGAGAAAGTAATTTCTTCGCCTTTTCCTAGGC

TGCTATCGAATAATGGAATGTACAAAATTTTCACTTTGTCTATTCTATAATATGGATACTCTT
TTCCAGCAGTCTTTTTCATTATAGATACTATATTCTGAAGTGTTGACAATTTCACTTTCTGAG
AACCTGATTCTTTTGTAAGTTTCAGGGAGACAGGAGAGATGACTGATTCTATTTCTCCTTTTG
TAATTTGAGTAAATTCAGCTGCCATGCACTCAATTTCTTGAGATATTTCACTATCACCTTTGA
GTGACACCAGAGTATGTATTCCTTCTCTCATCATTTCTCTTGTTGCTCTTTTAAATGAAACAG
AACCTTCCCATACATCTGATGTGCTTCTCCTACTATTTTGTCGAGTAAAAGAGTCCATTTTCA
ACTGAAAAAATTAAATAACAAGTTTTGAGAAAAAGGAATTTGATGAAATTTTTACCTTTTAA
AAAAAAGGAATTT
>Seq44 [organism=Erythranthe ophiovirus] RNA3 genomic sequence
CCCAGTTGATTGATTTTTTAAAACTTATCGTGTGCCATCCACCCCAGTTGATTGATTTTTTAGT
TAATCATGTTATAAGCGGCTCGGACTCCAGCCAGACTCAGATCACCAAAATCTGCTTCCTGG
TTTTCAAGAACAGAAAAGACTGGCAACTGATTGTTTCCCAGTTCTCCAAAAATGTTGACATC
CTTCCTGAAAGCTTCAATATTTTTCCGAATTATGTGATTGCGGAATTCAACCCTTCCATCCTC
TGATAAACTATAAATGATAGCACAAGTAAGTTTAAGAGTGAAATTCTTAATCGTCGGGCGAT
TCGGGTTCAGGGGATGCAATGCTTTCTGGGTTTCAACATTTGTGGAGATTGAGACAAGAAAG
TTCACTATCTGTAAGGCCAAATCTAGTTTGCGCCTTCTTTCCATGAAAGCCTCCATGGCATTC
ATATCAGTAGGGGTTGCCCCTATAGTGCCTTTCTCAAACTTGGCTGCTTCTCTTGCATACCTA
ACTGCCTTGTTGCCAGCAATTGACATCTTGCACCTAGATGCAACTGCAGTAGGGACGTGATT
GAGTTCCACCATCAAGAATGAGTAAGCCGGAAATTTCGCAGGGTCAGTACATGAAAGTGCT
TCACAAAACGCCTTTGTGGTCAGGTCTGTGTTCTTGAATATAGTCTCTCTGACAAATTTAGAC
AGATTCTTGTCAGTAGCACCTAGAGAAGGAAGAAATCCTTGATTGTAAATGCAGCAAAAAG
CACCTTTGATGGCCCTTTGTGCTGTCAAGAGATTGTCTGTGATCTCTAGGGATGAGACTTCAG
TTAATGGTTTTTCAGAGACTTCAAAGATCTGATTGTTATCAGTATTTTCTCTGTAAGTCATGT
ACAGAATTGCATCAAGAACAGCTGTTCCTGCAGCTATGACATTAGAAATACTTACTGGAACA
CCCTGAAATACTTTCACTTTCACTTCTCCATTGTTATATGATTCATCATTTTTGGGAATTGAAG
AAACGTAAGTTTCAAGTGCCACCCTTACATCATCACTTGTTATATGAACCACTTTGCTCCTGA
TGACTCCTACAACCTCATCAGGAGTCATCGGAGTTCTGTCCTGATAGTCTTGTCTGACTCTAG
GAACTTGAATTGTGACTCCTGATATGCCGGGTCTTGCAATTCTTGGTGGACTCTGTGAAGAA
CTGGATCCAAAAAGAAAAGGTGAAGGACTTACTGCTCCAGTAAATATCCCTGGTGAAGGAG
TATTATTTCCTTGACTTGTTGGCATGTTTGCCATATTCAGTATTTCAGCAGCTCTTCTTTCATT
GTTCAATTCATCCTCAGAGCAAACAATGTTGTTTCTGTTTAGATATATTATTGCATTGGGAGT
GTCTCTAGCCAATTCCCTGAGTCTGACCACATTTGCCTGATTTGGCCTGTTATCAACAAGCAG
AAAAAGGTTACTGCATGTTCTTTTTTCTGCCTCATCTTTCTGTTCCTTTTCAACAATTGCCAAC
AAACTTGAAGCAGTGTAGGATCCAGCCATTGTTATTCAGAAAAACCGAAAGAAAATAAAAC
GATGGAATTATAGACGCTTGAAAACTGAGCTATTAAAATATCTCGATTACGAAAAGCTTTGT
TGCTTCAAAAAAATTAATCCCCCCG
>Seq45 [organism=Gentiana ophiovirus] RNA1 genomic sequence
GCCTTACTAAGGACTTATTTGGCTCAACAATTGAGCATCATTGTTCTCTTGTTTATACTCCTA
GAGTTGAGTTAAGGTCTTTAACCTGCTTTCACAGCATCTCTTGGAACTGTCGTCAATCTCACA
TTGATTTTTTTGGAATTTTATTCTTATCACTTTGAGTTATTATTTTAATTCAATCACTTATTAG

CTCTATAATTCTAAATTGAAAATTCACTTTACTATAATTAATAATTTATTTATTATAATTAAT
AATCATCCTCTTCATAAACATCTTCATCTTCATCTTGATACATTTTAGCTCTTCTACACTCAT
TTTGGAGTTGATTTCTTCATTCATTTTGATTTCTTCTGCAGATCCAAACAAACTCGACATTATT
GTGGTATTCTCTTCATCTTCTTTAATTTCTTCCAACACCACAAGATCTTCATTGTCAATATTTT
TGAGGTTCTCTGTCATTGTTATGAAAAATTCATCCCTTTCCATGTTCGCGAAATATTCTTCTAT
ACTTTCATATTCTAATATCTTCTCTTCAATAATTTTCTCTTCCATCTTATTTTCCAAGTCATTTA
ATATCAATCCTTTGTATCTTCCTCTATCATTTGTTGGATAATTGAGAAAACCAAACTTTTTGT
GAATTAACCTTATTCTATTTATCAACAGTCGATAATCTAGTATATCATACCTATTATCCTTAC
TTTTGTTCTTCAATATATTATTGAAATTTATCTTCTTGAATCCCGAAACTTCCAATAGACCCCT
TATAATATCTCTATTCTTATAATAACTTACAGCCATTCCAGCAATATTATGTATTTTACTAGTT
GATTTCATTTTCTTTTTGTCCACTTTAAAATGCATCATAGTTTCCAATACATTTGTTTGACTTA
TATTTGATTTTTCTGTTCTTAACATATCTAATAATAATAACCAAAAGTCTATTCGATGTTTATC
ATACCTAAATGCTGAAATTGCACATTTGCTTAATAACTTAAACTCTTTAATTTCTAGATCATT
GTATCCTACTATTATACCCATATTCACTTCATTCAAAATTCTTCTTGTTACTACTATTTCTGAT
GGGTAATTATCTAGTTTTGATATCCTGTATAATATATTATCTGTCAACTCATCACCTTTATTGT
CAATAATTATCTTTGGCTTCCACACTTCTAATTTATTTTCAATTATTTGATCCAGTTCCTCTCT
TTCATTGGTTGTTAATTTATCTTTTGAGATAAGAGATATTGGTATCATAGATAAGTATTTTAT
ATTACTTTTGTCTTGCAATTCGGACTCCAAAGTTTTTGGGACTTCTATATAGTTATCACAATT
CGTCATCATATTGATTAGTTGAGCATCACTAATGTCTTCTAGTGTATGATCTTCTACTTCATA
AACTAAGTTGCTCATTGGATTAACTTTCACCATCTTCGCAGTGTTTTTGTCAACTGTTCGAG
GATTAGTGAAGATGTGAATTTCTTCTTAAGTCTTTCATATTTTTCTGTTGTTATCATTCTTTTT
TCTACTTCTTCATTGCTCATTTTTGTTCCTTGAAACATTAAATAACAGTATCCTGGGCTATCA
ATTTCTGGGATGAAGAATTCCATTCGATCACTTAAGAAAGCCAAATTTCTAAGTTCTTCTTTT
AATTTATTGAATATGCTGTTTATTCTGATTATTAATGTTAAACCAGAAGTGACAAAACTCATT
AGCATACTACTTAATTTTTCCTTCTCTCCTATGAAATGTGATATGTCACATATTACGACATTA
TGATCTAAGTATTTGGAATAATTATGATTATCAGTTACGTCATAATCTTGTTTTACTTCTAGC
ATTCCCATAGCTCGATTTATTATATTGTATCCATCATTTCTTGATATTGATGTATGTTTGATTC
CTAATTGTTTCATTGCTATATGAAAATCACCTCTTCCGCCGAATGGTTCAAAAACCTTTGTTC
TACTATTCAACACTCCTGAACTTTTAAGCATAGTTATCAATGAATAAGCACTTGGGAAAACA
TCAGACCTTGTCGGTGAAGATATCTTCCGTGGAGAACATGCTAAAGCAGATGTTATTTCGAA
TGTGCTGAGTGCCGCTTTGAGTGCTTCTAAGTCAAGAAAATCCCTTTGATGGACACTGAATG
ATTTGTATTCTACAGGTGACCATTCTTTATCAAATTTATCTTCAATATAATCATTTATTTGTTT
GTTATAATATGATAAAATTTGCTGTTGAAGGAATTTCTTACCATCGATTTCGTCCACTAATTC
AGAAGCTACTGATTTCATCTCCTCAATTTCAAAACCCAATAGGGGAAGGCCATTTATTATTA
TGTTGGAAATTTCTTTATCTTTTATGTAAAAGTGTGCTTTGCCTTTTCTTAATCTTCTGAAATT
TACAACTGTTGCATTGATTGTTCTATCTTTGCTTATCTTTATCATTGATTCACGATGTGTTAAA
TTCTCAATATTATACACCAAGCTGGTTGACAATATGAATAATCTCATGAGAGATGTTCTATA
ATGAAAAGAGTATCCCATTTCAGATACTTCTGATGCCCAATTTGTCCCATTAATATTGTGTCT
AAATAATTCTTTATATAATAAGGATCTTATAACCATTCTTGCTGGTGTCTCATTTTCCAGGAT
ATTCAATCCAGTATAATGAAGGTAAAAATCGTCAAAGAATTTGTTTCTATCCATTTGGTATCT

TTCAAAATTTGGGACTAGTTTTTAAGTGTATCATCATTTATTAATTGAGGTACTAATATTTC
TTTGTCTTTCATATATGACAAGACATTCCTATAACCTCCCACTTCTTCTATTAAACTATTAGGT
ATGTATTCAGCATGATTGATGAATTTACCTTTAATTTCATCATCGTAGGACACAAAATCAGA
AAAGGATTTATACAATTTTCCTTTATTCATTATTTTCTTTATGTTTGCCTCTTTCAATTCTCCCT
CAAATTCTGTCATGAACATTCTTGGTTTCATACCTTTGAAGTTGAATGATACATCTCTTAGCA
TTGCCTTTACATCTTCTTTTAATGATAATCTTATAATATCATTCTTTCCAAATCCAATTATTGA
TAGTTCAGCTTTCAAACTTGATAAGAGATATTGAACATTCAGGTTGTTATCTTCACCACCTGT
TCTTGAAATCAGTTGATCTATCCCACATAATTCAATGTGATTAGTTAATTCATTAGAGCTTAA
ATCCCCAGTTCTTGCTTTGAATCCCCCGCTTTGAGCTCTGTGGAAATATCTTCCTCCTCTTGG
AGCCACAACAAAATTTGATAGGTCTTCAAATCTAGTGTTTGTGAATGTGCTCAAGGTTACAT
TGCAAATTCCTTCTAAATTACTTATATCATCTTTTGATATTGATGAATATTTTTCACAATCCAT
TATCATCCATTTCACATATCTACACAGTTCATATGCATTGAAGATTAACTTATTTGAAAACAT
GCCTTCAATTTCCAAATCCCTATCAAATTTTACTTGATTCATGTGAGTTTTTAATGGTCTTTTG
TTTTTAATACCATCTTTTGTCATTACTTTGCTATTTTTCCAAATTGGAGTTATATCCGTGAAGA
TTGTTGTATAAGAGAAGCTACCTAATAGTGGAGGTTCTTCTATTTCTATGAACCTGAATTGAG
TTGTTTTTGTTTCTCCTCTCAGCTTATAGTTATATGACATTAACTGTCTGGTATTTATTAAACT
TGATATATTTACATTTTCTCCAATTAAGTTCCCTTGGTATTTGTATTCCATTTTGTAATTAGCA
TCCCATATTTTCCTCCACAAATTTTGGATTCTTTTCTTACCTAATATGAATGAAAAAGTAGTA
GTATTATCTATTTTAGAATAAATTTCTTCAAAATAGCTGAATATGGAACATTCAAAGAACTT
AGCTGTTATCCTGTAATTAAGGCAATTGGAAAAGGTTGTTATGAGTTCTGCTTCGAATTGCTC
TAATGATAGACTTTCCCTCCTGTTTACTATCTCTAACAAATTTTCATTCTTTATGTGTCTCTTC
AATTCTTTTTTCAATTGTTCTTTCAAAATAGTGTTTGTATCATATCTTCCCCCATCCAATGGGA
AAGATTCTTTCAAGTATCTATTGGCTTTATCACTCAATTTTATCATTTTCCATATTTTGGTCTT
TAGATCAGTATCTAGTATTGACAAAACATAATTGATTCTTTTCAAATTGCTTAAAGAATAAC
CTGATACAATTGCATCAGGAATTGAGGTTTGAGAATAACCATATACCGTATATGGTAAATAC
ATTATTGCCAATATTGCTTGTTTTACTCTATCATTGTCTTTGTTCATTTTATAAAATGCTTCTG
TAATGGTATTGATTGATGGAATTTCATCAGTATTTTCTAATTTTTCTTCTATATTCACCTTTCT
TAATAAAGAGGATGCATACCTAATATTCATATTTACAATTGAATAGAATCTAGGATCTAATT
CTATTTCTAAGAATTCACAGAACTTTATGATATCCCTAGAAGTATAATATAAGAATTTATAAT
TTCTTATCACTGTCTGTACAAAATGATCATCACTCCTTTGAGTTGCTGATGTGTATCCTGAAT
CTATATTCCTTACTTGTTCTATGACATCCCCCCATACCATTGAAGAATTAGCAGATATTACAG
GTATCTTTTTATAGTTGTTTTCTACTGCAACACCATTCAAGTAATGGTTCTTCAACATTGAAC
ATCTACCATTTGTTACTTGAGTCTGAGAGAGCTTGAGTAGGAGACCCATTTTCAATGCATAG
CTCTGCATGAATTGTATTATATCATTGAGTTTTTCAGCACTCAGGTCCTTTTCTTTGAAAACT
GCGCATGAATCGTCACTATAGGTAGTTGCAATTACATTTTCAAATTTAAGTTTTAACATCATG
TCATCCATCATTAATTGAGACTGTATTCCCCATAATGGTCCTAACCATCCCTCAATTGCTCCT
TTTTGTCCTTTAGAATGGTAGACAAAATCACTGAGACTTGTCTCATAGATTAAATCCATGTTA
GAAAAGACATTCAGTACTCCTGTCATATGTTCTCTTTCTTCGTATCCTTTATAATACCCAAAC
ATGTCACATATTTCTTCCATTATAAACATGTTATTTTCTGGTCTTTGAGATGTGTTATGACCTG
AATAATCTAAGAATATTGAGTAGGAATCTTCACCTTCTAAAAATGATGACATTTTATACATT

AGATCTCTTCTTTCATCTTCTGTCATGGTCATCATTTGATTTTTGACAAGTTTCATGGCTTTCT
TTACCATTTCCATGACTGTCGAGATATATATTTTTAGATTAAAAGATGCAACTCCAAAGAAC
CTTGCTGCAGTTTTTAGCTCTTTTTCTTTCTCTACTAATCTAACTATTTTATATTTTTCCATCTT
TTCTAGTTTCTGATTGTCTCTTGGGACAATGTTTATTTTTCTTTCAGCTTTTGATCTATCTTCA
AATTTTATTGATTCCATGGCGTTTTCATTTTCATTGGAATTGATGAAGTATATTAACTCCTTTA
CAGAATTATTCCCTGATGAATCATAATCATCTTTAGTGCAAGATTTATCGATCACTCTAGAAA
TGAGTGTTGATTCTTTTCCAGGTTCTAGCATTTTTCCAAGCCTGACATCTCCATATAAGCTTA
TGTCATTTATGATTTGATTGTTTTGTTTACCTTTTGCTGCCATTATTTCTAATTCTTGCTTTAGG
TTCTCTGGGCATTCACGTAATTCAGGGACTCTTCCAAATTTCTTCACATATGATGTTATTACA
TTCCTTCTAAATGCAAGTCTCAATGATTCAATTGTTACTTTATCCACAGGATGATTTCTATTA
GTTCTTTGTGAATATTTTGACAATCCTTTACTTGTGTCAACTTCTGCATAGATGAATGTCTTGC
CAATGCAAGACATTCTTATGCAGGTTTCGGCCTCTGATTCCACACAAATATTTATCAGTCTTC
TAGTCATTGCAACTGAAATTTCCATGGAATCAATGATATTATCTATTAAAGGTAACAGTGCT
GATTTGTATTTCATGTCTGCCATTAATAAGCATGTTGACTCATAGAGTCCTGCTAATCCGACG
CGTTTTTTTGCAGGTAAGAATATCAAATCTGTTACGTAATCAATGAACATATTGTCTTTTCGA
TATTTTGTCGGATTATTTCTTATCACATTTAGATCCTTGCTTATCTTCATGTTCATAACATTTT
CAGCTAGTGTCATTAGATAGTTTAAATGGTCCAGATTCCCCATACTCACTTTCCCATTTATAA
CCCAAGCAGTCATTGTATGAGAAATAAATATTTTGATTGATTCAATTTCTCCATCTAGATCTC
TCATGTCTATGTCGACAATTGAATGACCTGATTTGAATGTTTCAGCATTTATCCTTTTGTTCTC
ATAAAAATCAATACTTCTACTATTTATCCCATTTATCTTTTTTGAATAATCTATTTTAATTTTT
AGGTTTATCGTGTTCACTAGATCAAACACCCACATTGCTCTTGATGTATCTTCTGTTATTTCA
ACTTTGTCTTTTACTTCATCATATTTTGTTTTTAACTTATTCATCAATCTGATAGACACAGCAT
CTATCTCTTTCTCTTCAAGCTTAAGAGCAAAATCAATCAAAGTAGAGTGGAACAAAGATGTG
TTCCCTAGACTCAATAAGTATTGATAAAGGTGCGTCACTTCGTCTTGAGAGCATGTCACGTG
CTTATCAAAGAGATTGTTTTTCTTCAGAAATTGCCTCAGAAAATACAGATCCAATTTGTATCT
CCTTCCCCATTTTTCAGGGTACTTTTCCCCTTTAGTGAAATACTCTACCAGTTCAGGGCTATA
TGTCCTGAATGGTTGATCAATTTTTGAAATAAGTGCCGGTTTATCTACATAGTTCAGATCAAA
CTCTTTTGGTTCCCATATTTCTGAGTATATGAATCCTTGAGGAAATTCAATCATTTCCAGTTTT
GGTTCTTTCGCTTCTTCTTCTTTTTTTTGCTCGAAGATATTCTATAATTGTCTTCTCATGGTTTT
CAATGAAAACTTTCAGCCATTCATCTTTCTTCAACCCTGATACAGTCTTTTTGAGATTAGTTA
ACTCGTCATTTATTTTCATTTGATCCTCTTCACTTAAGTTGCTCATCACCCCAGATTCTTGTTG
AAATGTTTTCTTGTTCTCCATTGCTCAGTTTTGCTTGCTTTTCTTTATAAATTAATACAGAAAT
CCTTTTCTTTAATCAGATGATTTTATTTAAAACGATAATGCAGAATTCAATTTGTGATCATTA
AACAGAGTGATTTAGATTTGAAATTTAAATTTTATTGGAATTTTATTTGAAAATATTATCAAA
ATTTCAAAATCATTAAAATTCGGTTGAATCAATCACCTTTTAATTTGTCTTCATTTGTTTTACC
AGTCAATTCTATTTCTTGATTATTTTCATCCTCAACCGTAGACCTAGGAGTATAAAATTCTTC
ATCATCTATTATTATTTCTCTTATTATATTATAATTATCTTTTAATTCATTATAGTATTTTCTTG
TCTTGCTATCATACATCTGCAATATGTCATAATTTTCTTTGTTGCTTTGTTCAGGAACAAAAG
CATATTCTCCCAAATCTAAGTTTGAGTCATGAGATATTCTAATTCCATCTAGTCTTAGATTTA
CAAAATCATCACTCAAATAATTATCATCAAAATTGCTCATGATTTGTATTTCAATGCCTTCAA

TAAATACTGAGCTATTTCCCTCAGAATTCCTTATCACATAGCCCTTTTTCCTATTTATGAATTC
ATCTGTTCTGTTATTATTCAGTAACCAAATGCCCATTAACCCTTTATCAATGACACCGGGTAA
ATTGGGTTCAAATTCTTTGATTATCTTCATTGCACTTTTTATTTCTTCTATTTCTCTGTCTAATA
ATCTGTTAGAGATTATCACTCTATTCATTTTAATTCTAATCTACTAAGTATCTATTTTTCTTTG
>Seq46 [organism=Gentiana ophiovirus] RNA2 genomic sequence
CTTTTCAGACCACAGAACTACAGACCTGATCGTTCATAGATCTTTGGTTGTAGTCTACAGCCT
TGGCCACGTCAGGGCATCATGTTTTTCTTGTTTTATACTCGTAGAAATCGAGTTTCAAGAAGA
TCAATTCTTGATTTTAGACCTGTACAATTGAATTGTACTTTTAGATTTCTGGAATACAAACTT
GATCGTTCATAGATCTTCCGTTGTAACCATCAATCTGCCACATCAGGGCATTCTTTTTTTCTT
GTTTTAGGCTCGTAGAAATCGAGTTGAAAGAAAATCAAAGCTTGATTTTGATTCACAAGTTT
TATTTAAAGCGATGTTCTTACATATATTAAACAGAAGATTATTGATCGCTTTAACAAAATCA
AACAAACAAACAAACCAACCACCTCCAACTCAAACCATCATTCAATTGTCAACTTAGCCACT
TTTGACAGATTCCTTCTCCCAAGCTTCATCCTTCAACACCATCCTTCCCTCTCCAGTATCAAA
TGTGAGGATGATGACATCATCCTTGAACAAATAAGCAGACCTCCCTTCTTCCCTCATAGAAT
CAACAATGCCATCTTTCTTCAAAGCTAAGTAAGATATGAAATTAAAATCAGCTTCAGGGTCT
TCAAGAAAAACTGCTTCACTGAAAACATAAGTTATCTTATTGAGATCTACTCTGCCTGCTGTT
TTGCATCCCCAGACTTCTTTTTCTCTCCAGTTGAGAGTTGCTTGAAATCATGAGGAACATCC
AATTCATTAAGCACATGCAATTGAGAAGTTACATCCAACACAAAAGGTAGGTTCATGGGATT
CTTAAAAATCCTGAAATTGAATTTCAGAGATCTTTCAGTAACAGCAGGTACTAGAGTATGAA
CTTGAGATTCAGTTGGTTTGACCTCCATAAGATCTCTATGAGTTTCAGAGATTGGGGAGTTGT
CTTGAGCATAATCACTGAATTCAGATTCATATGCTCTCTCAACATCCCTATCTCTGTTAGCCA
TCTCCTCCTCTCTCATCTCATCCATCTCTCTCTCAACTTCCAGTTCTTCCATGCTTCTCCTCCTA
AAAGCAGCTCCTCCTCTAGGGCTAAAGATCTCCCTATTCCTCAGCAATTCGGATCTTGTTCTG
GCAATGGCTAACTCCCTAGGACCTCTGATCATTATTCCTTTATTCTTCTGGTCATATTCCCTCT
TATCTTCCAATTCCTTTTTCTTATACTGACTCCCTCTCAGTATTTGTTCCCTTTTAAGATCTTCT
CCTATCTGTCTCAGGATGGTCTTCCTATTTGTATCTTTCGGTCGCACTCTAGGTTCCATGCAA
ACAATCATTGTTGGTTTTTCCAACATCAGAAGAGGATTATAAGATTCATGAATGAAGAATCC
AATTGTCATAGCAGCATATGCTCTTTTGATGATTGGAATCCCTTCAGACTCAATTTCAACTAC
AACTTTGTCAACATTTTTCTTCGCTATAAAGAAATCCATAGACAGCTCAACAATAACCTCTTT
TCCAGCATTGAACTTCACAGATTGAATTGTCCTTTGAGCTGACCCCATTTCTGCTCCTTCATC
ACTCAGAGTCACAGTAATTTCACCATCACATTCCCCCCTATTAGCAAAAAGGGGAATATATA
GTATTTGGACTTTCTCAAAAGTGATGAAAGGGTAATCTTCAGAACCACCTATTTTGTTTACAG
CTGTTTTTATCGCATTATGCACTTTTTGCATTGATGTTAACTGCAACTTCTGCTTTTTGAATCC
ATCTTTCAAGTTGATAGTGATTGGAGAAAGACAGCTTGTTATCTTATCAGTTGCCATCTTAGC
AAAATCAGCTGCAAGAGATTCTATTTCATCAGACACATCATCATCTCCGCTTATATTGATCAC
TTTAGTCTTCCCTTTTGCTGCTCTGTTGCTCTCAGTTTGATCTATTCCAAGCGTCAACCTTCTT
GAACTTGCTGGCAGCAGAGCCAAATCCATTCTTTCAGTGTTTCCATAAAACCTAGAAGATGT
CGACATTGCTTTCTTCAACTGAATACTTGCGATTCTCAAGCAAAGTGAATCTGCGTTTCTCTT
C
>Seq47 [organism=Gentiana ophiovirus] RNA3 genomic sequence

AAAACGAAAGCGAGCAAAGCTCTAAATTATAGAAACATAAGACTTATTATTTAAGAAACAG
AATTAAACACCATAAATAGCTTTCAATCCAACAACAGAAATATCCATGAAATCAGCATCAAC
ATTCTCAATGAGAGGCCAATGAGCATCCTTCCCTTCCCCCATACCAAAGAAATTCACATCAG
ACTTGAACGAATTGTTCTGATTTTTCACAATCTTCTCTCTCATCTCCAACCTTCCCTTTAAACT
CAAACTTTCCAAGCAAGCTCTTGTTAACTTCAATGTTAAATTTTTCACAGTCGGCTTATCGGC
TGATAAAGGATGCATTCTAAGTTGTGCTTTAACATTTCCCACCAATGACCTCAGAAACTCAG
TTAATTCTCTTGCTCTCTCAACTCTATCTTTCTTCTTTACTGCCCTTGTTGCTTCATCAGCATCC
AATTTTCCAGCCACTCCAGGAATGACAGCCTGGTCAAAGTTTCCAATGTAATCTGCATATCT
GACAGCTTTGTTCCCAGCAATTGACAATTTGCATCTTGATGCTGTTGCTACATCAATCTTATT
AAGATCAATATTTAGGAATGCTTTCGCTGGGAACTTCTTTGGATCTGCAACGCTAAGATAAC
CAGCTATAGTGGACAACTTGTCTACTTCAGAGTTGTATAAGTTTCTGATGAATTTTGGAACTG
GTCTGCTATCAGAAAGATCTTTAGGAGGAAGGTTCCCTGTTATGAATACCAGGGCAGTAGCA
GATAAGATAGATTTCTTTCCAAGGGAGAGATTTTGAGCCATAACAGATGGCTTCAATTCTGT
AACCATTATTTTCTTGAATATTCCATTGACAACTTGATCATTTTTGTAGCTACAATACAAGAT
GGCATCAAGGACCATTGTACCTGCTGCAATGAGATGCTCCACCGAATTTTCATTATCATTAA
AGTATTTAATAATAATTTCCCCGTTAGAATAAGCTTTGTCTTCACTTGAGGCTGTTGATAAGA
ACTTTTCAAGATGTTCCCTTATGCTTTCTGCAGTGACTATTATTCCTGCTTTGTTGACATGCAG
CTTAAAAGTTTCACTGTCTTTAATCTTCTCTTCACCTACTGACTCGTAAGTCACATCGCTTTCA
TTAAAATTCTTTTCTTTTTGTCCGGAACCTTCTGTCTTTGGCCTATAAGATGCGTCCATACTGA
AACCTCCTGCTATTGCAACGACATATTCAGCACCTCCTCTCTCATACTTATCCAAACAACTCC
CAACTTTAATTAAATTCTGGGAATGGATAGTCTGATCTTCCTTAACTATACCGAATTTCTGCA
AAGTGTTGAGCTCATTTGGTGTTACACTGTTTTCATTTTTATTATATTTCTTGGCAATAGCATC
AAGCTCCACGATTTTGATATGATCACCTTCCATTACTGATCAATTTCAGTGATGAAGCTGATA
AGATTTATAAAACTATATTTCTTAGAT
>Seq48 [organism=Gymnadenia ophiovirus_den] RNA3 genomic sequence
ACCAGGATAACTTGTCAAACGATCCCATATGCTCCCCTGACAGTCCCAACAGACATATCAGA
AAAATCAGCATTAGGATCAACAAATATCGCATAAACCAGATTACCACTGGCATCAGTCTTTC
CATAAAAATTCTCATCCTTCTTAAACGCTTCTATCTTATCAGAGACAATCTTACCACGCATAA
TTTGGCGCCCAATCTGGCTTAGGGAGAATAAAACGGCGTGAGTAAGTTTCAGAGTAAAGTTC
TTAATGGTCGGCTTCTGAGGATTTAAGGGATGCATTTTCAATTGCGACTTCCAATCAGCAGA
AATGCTGCATAAGAAGTCCACAATTTCAGTGGCTGTTTCCCATTTTTTGTACTCCCTCATAGC
CCTCTGGTTCTGCAAATTGTCATTTATTCCCATTGTGTTCGGTACTGGAGCCCTTTCAAAACC
CTTTGCTAACATAGCATACCTAACAACCCTATTCCCTGCAATTGACATTTTACACCTTGAGGC
AACATCAGTAGGGAGCCTTTCAATATTTATATTGAGAAATACTTGAGAAGGGAATTTTTGTG
TGTTAGCAGAAGACAGCATTTTTGCCAAATCACTCGAAGTGAAATTTGCATTACCAAAAAGA
GTTACCTTCACAAACTGAGATAATCCCCTTTCGACACTTCCATCCATTGGCAGATATCCTTGA
TTGTAGATTGTAGCAAAAGCACCTTTTATAGCTTTGTGACTATTCGATATCCTTTCTACAGCT
TCTTTGGGATCAAAGTCTGTGTCACCCAATTCTTTCATAGAAAATATGCTCTGATTACTGTCC
AGACCCTGATAGGAGTAATACAGAACTGCATCCAAAATTTTTGTACCTGCAGCAACTAGATT
CTTGATTGATAGACCAACATTGGAATAGTAAAAGATTGTGATTTCTCCATTTAGGTATGGTTC

AGCATATTTTGGAATTGTGCTGACATAGTCTTCCAGAGTTGCTCTTATCAAATCTTCCTTGAT
ATCAATGCAGTGTGAGTCTAGAATTTCACTGAGCCTATCTAATGGTACTCTTCGTGTTTCACC
TATCAGTCCAGGTGTAGTATTATTCATCCTTGAACCTCCAGCATTATTGTTACTCTGATTATC
CATAACACTTTTCTCAGTCTGAGAGATTAAATTCCCATTAGCACTTAAGTAGATAATGTCATC
CGGGTGAGAAGACATCACTTGTTCCACCCATAGTATCTTCGCAGGATTTATCCCTCCATCAG
ATGACTGGATTCCAAGAATTTTGAAGTGATCATTTATTTCTCTATCTCTTGTTTCCTGATGAG
ATAGATCGAATATTTGCTTAGCAGTGTAAATTCCTGACATCGTTAAAACTAGATTTTGAGAA
ACAGAGAACGCAAGTTTAGGGGAATCTGAATTCGTTAATGCTGGATTACTTTTA
>Seq49 [organism=Gymnadenia ophiovirus_odo] RNA3 partial sequence
CAGTAAGTATAGCAAAAACCAGATTACCACTGGTATCAGTCTTCCCATAAAAATTCTCATCC
TTCTTAAACGCTTCGATCTTATCAGAGACAATCTTACCACGCATAATTTGGCGCCCAATCTGG
CTTAGGGAGAATAAAACGGCGTGAGTAAGTTTTAGGGTAAAGTTCTTAATGGTCGGCTTCTG
GGGATTTAAGGGATGCATTTTCAATTGAGACTTCCAATCAGCAGAAATGCTGCATAAGAAGT
CTACAATTCCAGTAGCTGTTTCCCATTTATTGTACTCCCTCATGGCTCTCTGGTTCTGCAGATT
ATCACTTATTCCCATTGTGTTCGGTATGGGAGCCCTTTCAAAACCCTTTGCTAACATTGCATA
CCTAACAACCCTATTTCCTGCAATTGACATTTTGCACCTTGAGGCAACATCAGTAGGGAGCC
TGTCAATGTTAATATTGAGAAACACCTGAGAAGGAAATTTTTGTGTATTAGCAGAAGACAGC
ATTTTTGCCAAATCACTAGAAGTAAAATTTGCATTTCCGAAAAGAGTTACTTTCACAAACTG
AGATAATCCTCTTTCAGCACCTCCATCCATTGGCAGATATCCCTGATTGTAGATTGTAGCGAA
AGCACCTTTTATAGCTTTGTGACTATTTGATATCCTTTCTACAGCTTCTTTTGGATCAAAATCT
GTGTCACCCAATTCTTTCATGGAAAATATGCTCTGATTACTGTCCAGACCCTGATAAGAGTA
ATACAGAACTGCATCCAAAATCTTTGTACCTGCAGCAACTAGATTCTTGATTGATAAACCGA
CATTAGAATAGTAAAAGATTGTTATTTCTCCATTTAGATATGGTTCAGCATACTTTGGAATGG
TGCTAACATATTCTTCCAGTGTTGCTTTTATCAAATCTTCCTTTATATCAATGCAGTGTGAGTC
AAGAATCTCACTGAGCCTATCTAATGGCACTCTTCGGGTTTCACCTATCAGTCCAGGTGTGGT
GTTATTCATTCTAGAACCTCCAGCAGCATTGTTACTCTGATTATCCACAACACTTTTTTCAGT
TTGAGATATTAAATTCCCATTGGCACTTAAGTAGATAATGTCATCCGGGTGAGAAGACATCA
CTTGTTCCACCCATAGTATCTTAGCAGGATTAATACCCCCATCAGATGACTGGATCCCAAGA
ATTTTGAAGTGATCATTTATTTCTCTGTCTCTTGTGTCCTGATGAGATAGATCGAATATTTGCT
TAGCAGTGTAAATTCCTGACATCGTTAAAACGAGATATTGGGAAACAGAGAACGCAAGTTT
AGGGGAATCAAAATTCGTTAAGGC
>Seq50 [organism=Holcus ophiovirus] RNA1 partial sequence
CTCGCAATGACTGTAGTATTGAACCTTATCACAAAGAACCTTATCACAAGACGAGAAGACA
AGAAAGGAAAACTTGACAGCAGAACAGTGTTCCCACCTTTAATAATCATCATCCTCGCAATC
TCCCCAGTGAGCGAATATCTCATCATCACCAAATTCCTTTTGTAAATCTTCAAATGTGCTCAA
GCGTTCATTCTCCTTTATGTCTTCTTCTGTTGCAAGGAAACCTAACTCCACAAACTTTTCTCTT
AATCTATCTTCATTCTGTGTTGCATTTTCCATTCTGTCTTTCATTGCTGCTACTGCACCACCCA
AATGGTCCATGAAGGAGAAATATTCCTTATCCAGATCTAGGAATAATCCATCCAATTGCTCA
GGAGTCATGTTAGAAACATCAATTCGAGGTAGTTGTACCCCTTCATTCGTTGAAGATAAGCT
GTTCCAGATGTCATTGAACAGTGAGTGTTTTATTCCAAGGAAATGAGGTTCATTTGGTAATTC

TTGAGAAATGAGCATGATGCGATTCATGTACAACTTATAATGTAGTATATTGTTTCTCGTCGT
CTTATCCTTATGGTCCAGTATAGACCTTTTTCTTACATTATCTAGGTTTGCAACACTTAGCAG
ACCCTCAATGATTTTTCCAGTCTTGAATGATAGTATTGCTTTTGAAGCAATTTCATAAGATGA
ATTAATTGTTCTATCTAGGTTTTTCTTGAATTTCTGAATATTTATCATCTCTAGGACTTTATCT
GAGTCTACCCTTTTCTCTTGAGATGATAGATTCAGTATCAATTTCCAACATTCTATGGTCATT
CTCTTATTGTGTTTGGTTGTCATTAATTCATCAATTAATTCAGTTAATTCTGTTGTGGTCATCC
CTTCTACTCCCCTTACTATGACAATGTTGTTGTCTGATATTAGTCTCTGGTCAACCTCTATTAT
ATCCTGCTTTTCCTTACGTATTTTCCTTCCTAGGCCAATTAAGTTGTTTGGAACATTTTCGTCT
TCTGAAGGTCCTTTTGATACCAGTCTTTTGTCAAGTTCTATTATTTTATCTTTCATTTTATCTTT
AAAAGCTTTTTTGACATAACCCAACATATTATCTTGAAAATCTTCTATAGATTTTATTTTGTT
CTTCATCTCAAAATTGACATGAACATATTCAGGATCTTCTTCGAGTAACATCTCGGTCAGAGT
TGCATCACTTATCACTTCTTCAGTCTGATCTGTTACTTTAACTGGTGGAACTGATATGACACT
TCTGAGAGAAACTCTCGAGACTGCGTTTATCAGAGCAGAAGACAATATGGACCTAGAGTAG
CCTTTTAAGTTCCCTGAATTTTCCTTTAGTCTAATATCTTTTGTGAAATCTATTGTTAAATAAA
CATAACCTGGTGATTCTAATGCAGGCATGTAAGCAGTCACCTCATTTCCTCTTATTTCTCTCA
ATATTTCATTGTTTATAAACTTGTTCATACCATTCAATCTGATTATCATCTTCTTTTTGCTCAA
GTGGCAATCCCCCACAATTCCACTTAAGCAATCTTTTTTCTCAGTGATGTGAGAGATATCAAT
CAAGATGATGCTGTGATTAAAATAACTTATGTAATCTGCTTGTCTGAAACAATTAAATCCTG
TCTTTTTTGATGTCATTCCTGGTATTCTCATGGCTAGATTATAACCATCTTCACGTGACAAGG
TGACGTGATCTATTCCTGCCTCGAGCATTGCTAAGTGGAAATCCCCCCTTCCCCCGCAGAGTT
CTACTATTGAGTCATCTTTAGTTATAAAACCCTTCCCTTTGAGTGATTCTAATAGTCCTTTCGC
TGATGGATAAACAGCAGACATTGTAGGAGAGGAAACCTTCTCAGGACTCACCATCAAGGAA
CAGGCCGTTTCAAAACCTTTGAGAGCAGATTCTATGCCTAAGTTGTCTACTAGATCATACCA
CATGATGTTCATTTCACTATATTCAACCTTCCCATATTTTATTCCATCCTTCTTCCTTGTCTCA
GATTTGTGCATGTCCAAATAATAAGTCCCTAGTCTCATTTGCTCAAATTCTATTCCAGAAATC
TCATCACAGAGCTCACTTGCACATATATGGACCTCAGAATAACTGTATCCTAAAGTAGGGAA
AGAATGCACAATCATCTCGGTGATTCTCTTATCCTTGATGTAGAATTGGCAAGTCCCTTCTCG
CATCTTCTCAAAATTAGACTTACTATTCATCACGGTTCGGCTCTTATTAACTTGTAACTTCAT
ACAGCTCTGCCTGGATTCAATCATTCTATATGACAATGATGTTGCCAGAATAAACAACCTCA
AGAGAACATCCTTGAAACCACTTGAATAGCCTCTCTTGACTAATTCACTTGCCCACAAGGTC
TTCCTTATTGACCTGTTGTTGACTTTAAATAGTTCTTCATAGAGTAAACCCCTTATCACTGAT
CGTGAAGGCGTTTCATTCCCAATCACATTTAGAGATTTGTAATATCTATAGAATTTATCAAAG
AAATCTATTCTGCCAGACTTTGCAACATCTTCTCCAACTAACTGTTCAAATACGGGCTCAGA
AATTAATTCAGGACTGATTATCATTTGGTCTATCATGTACGAATGGACTGCTCTGAAAGCAC
TCTCTTGCTCTATGTATTCTTCACGAGTTGTGTCAACATGGTTGATAAATTTACCACTCAAAT
TTTCATTCATTTCTATGAAAGTGGAGTAATTGTAGTACATTTTCCCCCTTGTTCTTATTTTTTC
TCGTGAGGCTATATTGTACAGTTGTTTTGGCTGTTCAATCATATTTAAATCAGTCAGTGAGAA
AGTGACATCTTTTATGTGCTGGAAAATGTCATTGGTAAGAGTTAAAGAGACGAGTGACTTTG
GCTCTGGTCGAAGCAGAGAGAACGCAATTTTCACATATATCATCAAGTATTGAACATTGAGA
TTATTGTCAGAACCACCTGTTTTAGCTAGTAACTGATCAACTCCTGTGATGTCATAATTGTTT

GATATATGGTTGGAAGATAGGTCACCGGNNNNNNNNNNNNNNNNNNNNNNNNNNNNNNNNNNNNN
NNNNNNNNNNNNNNNNNNNNNNNNNNNNNNNNNNNNNNNNNNNNNNNNNNNNNNNNNNNNNNNN
NNNNNNNNNNNNNNNNNNNNNNNNNNNNNNNNNNNNNNNNNNNNNNNNNNNNNNNNNNNNNNNN
NNNNNNNNNNNNNNNNNNNNNNNNNNNNNNNNNNNNNNNNNNNNNNNNNNNNNNNNNNNNNNNN
NNNNNNNNNNNNNNNNNNNNNNNNNNNNNNNNNNNNNNNNNNNNNNNNNNNNNNNNNNNNNNNN
NNNNNNNNNNNNNNNNNNNNNNNNNNNNNNNNNNNNNNNNNNNNNNNNNNNNNNNNNNNNNNNN
NNNNNNNNNNNNNNNNNNNNNNNNNNNNNNNNNNNNNNNNNNNNNNNNNNNNNNNNNNNNNNNN
NNNNNNNNNNNNNNNNNNNNNNNNNNNNNNNNNNNNNNNNNNNNNNNNNNNNNNNNNNNNNNNN
NNNNNNNNNNNNNNNNNNNNNNNNNNNNNNNNNNNNNNNNNNNNNNNNNNNNNNNNNNNNNNNN
NNNNNNNNNNNNNNNNNNNNNNNNNNNNNNNNNNNNNNNNNNNNNNNNNNNNNNNNNNNNNNNN
NNNNNNNNNNNNNNNNNNNNNNNNNNNNNNNNNNNNNNNNNNNNNNNNNNNNNNNNNNNNNNNN
NNNNNNNNNNNNNNNNNNNNNNNNNNNNNNNNNNNNNNNNNNNNNNNNNNNNNNNNNNNNNNNN
NNNTATTCTTGTCGAGAATGAATTCTTGAAGACATTGATTAGCCTGCATTTGTATTCTAACTC
ATCTTCTTCTTGAGACAGATTCATTAAACGTAATAGCTCTGGGTTCTTCACAAGCCTTTCGAG
CTTTGTCTTGATAACTGGCCTCACAAGTAAAGCAGTGTCCTTTCTTCCACCCACTAAAGGAA
AAGAGTTGTCGATGTAGCTGTGAGCATTCGAGGAAAGATTTATAGTTCCTCCTAATAGTTTG
TTTGCCCCTGGTGATAGTATTCCCTGTAAATAGCTAATCCGTTTCACATTAGATAAGCTATAC
CCGGATATCAATACATCTGGCATAGGTGTCAATGAGTATCCGTACATGGCATAAGGACTATA
CATTATCATTAGTGTCAGGTCTTTTACTAAGGCACTGTCTCTATGGAACCTGAAAAATTCCAT
GTACCTAGACCTTGATGCGGGTATGAACACTTTTTCTGTTTTTCCGTATTTAAGCTCTCTCAA
AGCAATTTTGGCACTAATTTCGTAATTATTGACACTTGATTGGTATCTTTTGTCTAGACCATC
TGGTGCCAGGTTCTCAATCATTTTCTTGACTGAGCTAGAAATGACTTTTATCGAACGTAAATT
CCTCAGCAAAGTCTGCACCGTTATATCAGACGAACGTGTACAGCTAGATGTATAACCAGAGT
CTATCAGCTTAGTTTGCTCTGACTCATCTCCCATCACTTTAGGGCCATTGGGACTGATCGACA
TCATTCTTTTAATGCACATTTCTATTTGTTTCCCGTTGTAATAGTGTTCCTTCAACATGGAACA
TCTTCCATTTGTAACCTGAGTCTGAGATAGCTTCACAAGCAGTCCCATATCTTCTCCGTACTT
TTGTACGTTTCTTATGATGCCATTGAGTTTTTCTACATCTAGATCTGCTTGTGTGAACACCCC
ACATGAATCGTCAGAATAAGTTGTGCCAATGTAATCTTTCACCCCAAGTTGGATAAACATGT
CTTCAAGCATCAGCTGAGATTGTATCCCCCATAGGCCCCCAAGCCATCCCTCTATCGCTCCTA
ATTGACCTTGAGACAACAGAACCATGTCACTCCAAGTGTCCTCGACTATTATATTTATGTTGC
AAAAGACATAAGATAATGAAGTGATTTCTTCATATTCTTGCATTCCTTCATAATAACCATAC
ATATTGGCGATCTCCTCGAGAATGAAGTTGGTATTTTCTGGGCGTTGCGAAGTATTATGTCCA
GAATAGTCTAAGAAAAGAGAGTAAGAGTCTTTCATTAACAACTTTTCAGACATGGTATGCAT
AGTTTCTCTTCTTTCGTCATCAGTCATTGTCATCATTTGGCCAGGCAGCAGTTTCATTGCTCTC
TTGACCATTTCCATTATTGAACTTATCCACAGTTTCAATTTGAATGAGGCGATGCCATAAAAT
CTCGCTGCTGTTTTTTGTTCTCTTTCTTTCTCGGAAAGTCTCACATTAAAATACTTGTTCATGC
TGTTACTTCCTATTTCTCCTATCTTTTTCCTGTTCTCAACATATACTTTTCTATCCATTTCTCTG
TATTTGGTCTTATCGATGCCCATCGGGTCAGCAAACACTTCTTCCATGTTGTTGGTGATATAG
TACTGGATCTCTTTCTCTGAATTGTTCACACAGTCATAGTTGTCTTTTGTACAGGCCTTATCA
ATGATCCTAGATTGTAGGTTCATTTCTTCTCCAGGCTCTAAGAACTTGCCTAGGATCACATCA

TCATATCTGCTTATTTCTGAGATCATGTGGTTGAGATAATTTCCTCCTGCTGCTTTCATTTCAA
GTTGTGCTCCCAAATCTTCTGGTACTTTTATTAACGATGGAACTTTACCATGCTTTTTTATGTG
ACTAGTTATTACTCTCTGCCTAAAAAGTGNNNNNNNNNNNNNNNNNNNNNNNNNNNNNNN
NNNNNNNNNNNNNNNNNNNNNNNNNNNNNNNNNNNNNNCTCCTTTTGAGGGTTTAGTGCC
AGCAATAATCAGTGTTTTCCCAATACATGACATTTTGATGCAAGTTTCTGGCTTTGCCTCCAA
ACATATCTCCACTAGTTTTTCGGTCAGTCTACTTGAAACTTTGACACTCTCTGCTATATTGTC
AAGTATAGGCATGCTGGCAGACATTGTTTTTTCATCTGACATCAAGAGACAAGTTGTTTCAT
ACAAACTTCCCATTCCTATCCTTATGTTCTCTTCTTCATTCAGAAGTCCAACATAATAATCAA
CGAATTTTTTTTCACGTTCACAGAGTGACTGTCTTATCATTGATAGTGTGAATAGATTGTCAA
TCACATAAAGACTATATTGCAACATGTCCGGTCTGCCTATGTGGACTTTGTTACTTTTGCTCC
AATAGTAACCACTGATGGTTGATGAAATGAACACCATGATGTAATCATTGTCTCCTTCAAAT
TCGATCTTGGAGATGCTAGCACCAATTTTAAAATACCTTGTGTTTATCCTTTTGCCTTCATAG
AATTCAATGGTGTCATCTATTTCTTTGCCGTTTGTAGTAGCGCTAAATTGCATCATTATGTTCT
TATTCAAATGTTCGAAGATATCCAACACCCAATAATTCTTTGCACTGTCTTCAGTGATTTCAT
ATTCAGAGTCTTGTTTGATATTCTTATTCCCTAATAAGGACTTCGAAAGAGAGGACAATTCGT
GCTCCATCAGGGCACCACTATACTTGTAAATATTCTCTGACTCTTGTGATGCTTTTGCTTTTA
CATTTTTAAGTTGATTGATCAGCTCGATGGAGGATTCCTGGCTTGAAACCAAGGAATAACTC
CCAAGTTCAGTTGAAGCAGTCTGCAAGAAGATTCTCCTCGTTAACATTAAGTCAATTCTGCT
CTTTACCATCCATTTCTCAGGGTACTTTGAAGATGATATCATCTCAAGGTTCTCGGGTGTTAT
TTCCTTGTATGGGCANNNNNNNNNNNNNNNNNNNNNNNNNNNNNNNNNNNNNNNNNNNNN
NNNNNNNNNNNNNNNNNNNNNNNNNNNNNNNNNNNNNNNNNNNNNNNNNNNNNNNNNN
NNNNNNNNNNNNNNNNNNNNNNNNNNNNNNNNNNNNNNNNNNNNNNNNNNNNNNNNNN
NNNNNNNNNNNNNNNNNNNNNNNNNNNNNNNNNNNNNNNNNNNNNNNNNNNNNNNNNN
NNNNNNNNNNNNNNNNNNNNNNNNNNNNNNNNNNNNNNNNNNNNNNNNNNNNNNNNNN
NNNNNNNNNNNNNNNNNNNNNNNNNNNNNNNNNNNTTTTTATTTAAAACTTATGAAAGAT
CTACTTCTTATTATGAATCAATCATGACATTAAATCATTCGACCTTTTCCCTTATTAAATCATT
CGACCTTTTCCCTTATTATTCTTAGCATTGTTGAAATTAGTGATGTTGTTTGATTTAGTCTTTT
CATCATCTTTGTCTAGCATTGCAGAGTACTTTGGTATCTTAAAATTGTGGTGAATCTCATAAA
GATTATCTTCATAATAGATCATCATTTCTTCTTTTTCGAGGCGGAAAAAGAAAGAGGATAGA
ACAAGGTCAAAACCCTTTGCTAAAACAATTTCTATATTCTTGTAAGAATCCACATCTATCAA
ATCATTGACTGACTGTATGATTATGATTGCTTTTTTGTTGAAAGGAGATGACATTTCTTTCTT
GTCAAGTAGCCATGAACCTACAAAGAACCTAAAAAATTGTCGGATAGCTAGTTTTATATTCAT
TGAGACATCTTAACACTTTTTTATTCCTTTCGTCTAAAGAATTGTTATCACTCTTAATAAGTC
GAAGACTTAGATTCAAGCGGGATTCTAGTTGAGCTTCCATGATCTTGTTCAAGAAGGTTGAT
TTAAAATTTTAAAGGTCTGATGCCACAAGGTTATTGAGATC
>Seq51 [organism=Holcus ophiovirus] RNA2 genomic sequence
AATGCACTTCCTCAAATTAAAACCTTCACAAAAGCTCAAACATGGCTTTGTTCAGGGTTCCA
AAACGAAGAACGTGTAAAACTGAAGTTAATCAGCGGGCTGCGATAGCCGTAAGGAAATACT
GGTGTGTATTTCAAGTGTCCCTTTTATTTAAAACTTAAGTACACGCATCACGAAAATACAAT
AAATTATCACACGACATATATATATACGACAATCTTATTAATATGTTACATTTTACTATTATT

AAATTACTACTTTAAATACATTATTGTATATTGGAGACATATCATCTCTATCATTCCTTATTTC
TCGACTAAGACAACAGGGCTCAAATTATTGGATTTCTTGAACACCATATAATTACCACTGCA
ATCAAGCTCAAATACAATCTTGTCGTCTTTCAGCAAGACTGCTACATCATCACTCTCCGACTT
CAAACAATCCACAATTCCTCCTTTGACTAACTTCTGGTATGAAATCAAAGGAAAATCATCCT
TATTCGCATAAAGGACCTCCTTCATTCTAACCCAATGAGACCCTAGCTTCACCTCCATCCCAT
TCATGGCAAAATGTGTCTCTCCACGTCTTCCTGGTTTGTTTCCAACTCTTTCCTTAAATCCCAC
CAGACCAGGGTTGTAGAACCAGTGATCAGGTGCTCCTGTGTCAAGTATTATGTGATCCCCCT
TGTGAATTGGGACCTTTCTTGGGAGAAGATCAAGAACATCTTTCTCCCTTATAGGCTTGTAGT
TATTTTCTACTTCAGCTCCTGAGTCGTATCCCCCAGAAGTCTCCTCATCCTCTTCCAGTGATTC
TATCTTGATGTTGTTGGTGAATCTCATACTACTATTTCGTTCCTCCTCTTCATCATCATACTTC
AGTTGAGCAGCCTCAAAAGAGCCTTTTTTTGCTTCAATTCTTTTATTCACAGTCTCGACCAAG
TTCTTAATAGAAGAAGTTTTGTTGATGTCTTTCGGAAGTTCTATATTATCGATCAAGAGGACA
ACAGGATTCTTCTTTTTGATCGTCATGGGAACGTATTCCCTGTGTATGTAGAATGATAAGCAA
ACAAACGCGAATGCCCTTCCCCTAACAGGCACCTTTTGTGTCTTGTAACAGAACTCTATCAT
GCTCAAGTGAGCTTTTGGGATAAAATAGGAAGAAGAGAGTTCAACCATGCTCATCTTGTTCA
AAGGACAAGAAGTCTTAGAAACAGATTTTGACTTCAGCCCAACTTTTATTGAGCTGTCATTG
ATAGAGAATGTTATCTTCTCACCTGTTGCCTCTTCTTCGGGGAACAGAGGCAAGTACAATAT
CTTCAACCTATCCACTCTATAGAATGGATAATTGTGCTTTGGGAGTGAGGCAACAAGTGATT
GCATTGTCCCCAGTTTGACTTTCTTTGCACCTTCTTTCTCAGTGAGTTTTATCATCATAGGAGA
AACTATGGAGTTTATGTCTCCTGTTATGGCATCCTTGAATTCTGCAGCCATGCATTCTATTTC
AGGAGACATGGCTGTGTCTGAGCTCAGTGATGCTAATACAGTTATTCCTTCCTCAACCATCC
CTTCAGTAGCTTTAGCAAATGAAACACTTGGAATCATTGCAAGCTCTTTACCCTTTCTTCCAT
CTCTCTTTAATACCCCCGAGTTCGCCGCTTGACCAATCTGAACTTGCTTGGCCATTGGAGTTA
GGTTGGCGTTCAACTGGATTTTTAGAAAGTTCTTCTCCTTGTACTCTAGGCTTTTGTTTTCAGA
AAATTTTTGCATC
>Seq52 [organism=Holcus ophiovirus] RNA3 genomic sequence
TTGGCAGTCTCTAAGACTTACACTCCTTAACTCAAAAAGTTCTTGAAACTTAAGACCTTATTC
TCTTGTGTCCGCATTTCTTTCTTATCCTCACACCTCTTTAAGTTGATAACCCATAAGCAATAC
GGATCGCATCTACTGACAGCTCGGAGAAGTCCGCTTCTTTGTTCATCAGGACATTGTGTGTC
ACCTCATGTATTTGGTTGATTGATCCATAGAAATTAGGGTCACGTTTGAAAGATTCTATCTTT
CCTTCATCAATAGTCTTTCTGAGATCATCTCTTCCTTTTGGGGATAGTGAATATATGATAGCA
CAAGTCATCTGTAGAGTGAAATTCTTCCTTGTAGGTCTGCTTGCTGATTTAGGGTGCATCCTC
TTTTGTGCTTCAAAATTTGATCCCAGTGAGCTCAATAAATCAGTTATTGACTTTGCTTTGTTT
AACTTCTCAACTTCAGCTGAAAATCTTAGTATTTCAGCCTGATCGCCACTCCTAGGTGCTGTG
ACGTTATTCTTTTCAAACCTCTGTGCTAACATCGCATATCGTATTGCTTTGTTCCCAGCAACA
GACATCTTGCACCTCGAAGCCACTTCATTAGGTAACTGAGTCAACTCTATTCCCAGGAACAC
TTCAGCTGGGAACAAACTTGGATCTGCAGATGAAAGCATATCAACAAGCACGTTGCTGCTGA
AGCCCTGATAGCCCTTGAACAGAGTTTCTCTGACAAATTTTGGTAGTTGTTTTTCCTCAGTTG
ATTTCGATGGAAGTTCTCCCTGAGTGTAAATCAGACAAAATGCAGCTTTTATTGCCTTGTTTC
CATTCTCAATCTTTTTTGAGATATCTGTTGCATTGACAGTCCCAGGCTTCAGTTCAGTTGTAG

CAAAAATAAAATTATGTTCTGAAGAGTCCTTATAAGCCATGTACATAACAGCATCGAGTATC
CTCGTCCCTGCAGATAATAGGCTGCTTATAGAAAGAGCAACCCCTTTGAACCAAAATACTAA
AATATCACCTGTTTTATAAGTCTCAGCAGTCTTAGGTAAATTCACAATATAGTTTTCAAGGAT
ACTTTTGATCTTTCCAGCATCCATTGAGAGGCTCTTTATCTGTATCATTTCATTGACTACTTCA
GGTGAAAATTCAACCTCTTTGCCTTTCTCACTTGCAACTACAGTAGTCCCTGAACCAACCTTT
TGCATCGTGCTCGAGCTAACAGTAGTGCTGGTGATGAATTCAAGTGTTTTGTCTGATTTGACA
TACACCATTGCATTTGGGCTCTCCTTCTCCATCTCCTTCATCTTGTTCATCACCTTTGTGTTAA
CTCTTTCATCCTCTCTCAATATTTGTAGTGATATCAGGAGTTGTTTGTCATCTTCTGTGTACTC
AAGTTTCCTTGCAACCTCCATGACTTGGCTAACAGTCTTAGTTCCTGACATTTTGGCTTGAGA
CTTTCTGAGTTGAGTTCTTTCGGTTTTGTTAAGTTTGAGATTTTGCTC
>Seq53 [organism=Holcus ophiovirus] RNA4 genomic sequence
CTCATTCTTTAGAAGAAACACGTGCTGCCTTCCCTTTACACCATATCCTAATTTGATAAAATC
GCCAGGGCGGCAAGGTAGTCAGGCTCTCCCCTAGACCTGCAGACATAATCTACTGACCCAGT
TCGCCTGCTAGGATGTCCAACTTACCGCGATGGACCCCATCACAAGTAGAGCTCTCTTTTTG
GAAGAAGTAACTTCACTTCATGTCTGGCCAGCGTACTCGCTACTTTGAAAGCTTTCTGATAA
ACCAGTTCAATCCATGTTGCTAAATCTTCACATTTCTTGTTTATTTAAAACAAATAATCAAAT
AAATATCATTTCTTTCTAATGCTGTATTTTCAACAATTATTGCAGTTTTCAATGAAAAATCAT
TATAAAAAACGACAGAATTAAAAGAAGATATCACCATCGTAATCTTAGTACAGAAAAGTAT
CTTATCACTGCAGCTTCTTTCGCCCTTATTCTTCCTACTTCTCTCTCGATTGGTCTTTGGGGGT
ATAAGTAGGATAATATAGTGTCATCAAACTCAGAGGTAGTCTTATAAAGACGTAACATAGTC
GAAATCTCTATAATCAGCTTGTCAAATACATCATCCAGTACAAAAATTTCTCCATTGTGTTTC
TTCACAATATTGCAACACCCTCTAGGAAATGCTAAATCATATGTGTTCATCACTTTGTCTCTT
GAAAACTGAGTCAAGAAGTGATCTTCTTTTATCCCCATCTTGTTCCATGTGAATGTTATTAGT
CCAATCAAGTCCAAAGCTGCACTGTTGTCCTTCCCTTTCATACCATAGTGGTCATCATCTATT
TTCGAATCAAGGCTAGTAATGTATCTATCCTTCATGTATTCTCTTGGGGTTGTTCTTCTAGGA
TGCACCTGTAGGAAGTAATCAAGCCCACTTTCGAGGCTTCTGATTATGCTATCTTTTATTGGG
GATATGAAAAATTCTTTCTCATGCAGTGCAAAACTTTGTTTCATCTCATTTGAGTAGTGGTTT
AACAGGTTACTTTCTTTTAACTCAAATGAAACAGAGTCACAAGACAACTCAACTAATGAAGG
AACAATCAATTCTTTTATAATCAATCCCTCAAGCACTTTCAACTTCTCATTGAACATTTCTAT
GATCTTCATTCTACTCTCTGAATCCAATGAAATTGTTCCTGTCATACTGCTGCTGAAGTAGTC
AAACTTTTTATCGATAAATTCAATCCTGTTCCTCAGAAAGATTATTTCTTGCTCATTTAGTTC
ATTGATACCATTGAATGGTTTGAAAGCGATGTTTATAATCTCCTTGACGTGAGGAATGATTG
ATAGTGCTCTACAGAGAACCGGCTCAATAAACATGGCCCATCTTATCATGAAGCTCATATCG
CTGTCAAGCTTTGATGGTTTGCTATTTTTAATCTTGTTGATCATAAAGTTTTTGTATC
>Seq54 [organism=Lepidozia ophiovirus_tri] RNA1 genomic sequence
GCGATTGCAAAATTTGGAAAGAGCAAGAAATATGGCACTGAAATATGAGGAATTTGAAGAT
GATGGAATTAAATTTAATTAAAAACTGTTCAATTTTTATCAGCACCGCTGTTAACCGTGTACA
AAGTGCACTTGAAAAAGTGCAATCCAGTTTCAGGATCATACTTACCAATCTTCATCAAATTC
CATTCCATCATCTTCAAATTCCTCATATTTCAGTGCCATATTTCTTGCTCTTTCCAAATTTTGC
AATCGCTCTTGTTCTTCATCCTGGAACTCATCATATGTTGTCGTCAACATATAAGATTGTTCA

ATCTCTTGATTTTTCTTCACATTTTCATCCCAATCCTTGAAAAGCCCTTTAATAAAGGTATCTG
TTTTTATGGGTTCATCTATGGTGATTTGTTCTACACTTTTATCTAAGTCCATTGAATTAATAAA
ATTGAAGAAATTATCATTGTCCGTATTTGATGCTTCAATAAACTCTTGAATAGTTTTAAATTG
AGTGTAATGTTCTCTGAATTTTTGCAAATCTTCATCATTCATCCTTCCAGCTTTAACTTCCGTT
TTCCAAATGTCTCTCACAAAAGATTCGAGATCTTGATTGTTCGCTGTTATGTTGATGTCTGTG
ACTTTCTTTACAATGCGGTTAATCCTGTTTAAGATTAATTTGTAATTAATGATGTTGTTTCTGT
TGTTTTTTGTCTTCACTCGCATGATGGTGCTAAGATTCACTCTTTCAAGACCAGAGATAGCTA
TAAGACCTTCTTTGGTTCTTCCGGACTTCCAAGAATTTATTACCTGAGTAGTGGTTGTAAACA
GATTATCAATGGTTCTTCCGACACCTGAATTGCGTAGGGATTCAATGGATATCATGGGAATG
ATATCATCAGAACTGATTTTATCATAATCCTTTGCTAGGTCTAACAAAAACATCCACAAATC
CTTTGTAATCGGTAATGTATTCTGATCTTGTATGTGCTGAACAACCATGGTTAGATCAGAGA
ATTTCATCTCCTCAAATCCTGTTATTACAACTTTTGGTAATATGTCTGCTTTTTCATTTATTAC
TTCTAATCTAGTCTCTCCCGTTCTTCTATAATGCCTCATGCAGGACACGAACGAACCAAATCC
AATATCAAGATCAGATGTGATGTAATCGAAATATTCAGTTTTTATCCCGGGTATGTGTTGCA
AACCCTCAGCACTTGTGCAGAGTAATGCTCTTATGTCATCAGAATTTCTCACATTGTTTTTA
GGGATTGAGGGACCTCAACAATCGAATCCTGAGTATGACTCATTAATGAATCTAATAATTCA
TCATCTGTGATGAATTCTTCTGTATGATCCACTACGTCTTTTCCAATATGACTCAGCATGGGT
TTGTGTAAGAATCCCATGTTTCCTTGAGACAAGTATTCTAGTATCAGGTTACCAGCAAACCCT
TTATTGCTGCGTTTTCTGGAAACAATTTTCCCTCCTCTTTCTTTTGCCTCCAGTGTGAGATATA
AGACCCCACTGCTATTCTTTTCTGGTATTAAGATTTCAATGTCACAGTGTTGACCCATGACAT
CAATCAGTTTTTGAGTTATGATTGAAGATACTGCATTCCATCTGAGAACAATCTTTTTCTTGA
TAGCAATCATTTCTGATATGCCCTTAGTTAATAAGTCCTTCTTTCCAGTAAAGTGTGATACAT
CCAAGAGAATAATGGAGTGGTCCATGTAATCCATAAAATTTTCTCTGGAGAAAACATTCCAA
GAAGGTTTCTTCTCTTTCATCCCCTTGATTCTATTTATTAGGTTGAATCCATCATTTCGGGAGA
CTGATGTATGGTTCACTTTTGCTTCTGTAAGTGCCAAATGGAAGTCTCCTCTTCCAGCACAAA
GATCTGCGACTGTGTCTTTTTCAGTCAAAAACTGTGAACCCTGCAGACACTTTATAACTCCTT
TTGCCGCAGGGTATATGTCAGATTTGGTGGGAGATGAAAGGTTTTCAACGGTACAAGACAAC
GCACAAATGTATTCAAATGCCTTGATTGCGGCGACACATGCATCCTGATCTTTCAAGTCGTA
CATATCAATACCGAAAGAATAGTAATTGACCAAACCATAAACTTTTGATTCATCCTTCTCGA
TATATGAAGATCTCTCTGTTTTAAAATAATCATAAAGTTTATATTGCTCAAAAACCTTATGAT
TTAGTTCCATCGATATTTCATCTGAGCACTTTTCAACTTCAATGACCGTATAAGACATAGTCG
GAAATCCATAAATCAGGATTTGAGAGATAGCCCTGTCCTTTATGTGAATATGATGAGTTCGG
TTTAATATTGCTTTAAAGCAACGAACACTATTGATCTTGGTGGCGACTGTATTTATATGCAAC
GTACTTTTGCCATTATTTGTCTTGAGTGTGTATATGAGGCCACAAGCAATGATAAAAAGTCTC
AATAAATTGTGTCTATAATCCATGGAAAAACCTTTTGTAGTCATTTCCTTAGACCACACTCTT
CCCTGACTGTCATGTTTAAAGAGTTCTTTGTATAACAATCCTCTTATCATGGAAGCACTTGGT
GACTCTCTTCCTATGATGTTCAATGAAGAATAGAAATCATAGAATTCTTTAAAGAATTGTTCC
TTTGTGGTGAAGTTGTCGTAGTTTAATAATAATTGTTTTAACACGTCATCTGATATGACTTCC
GGTGTTATTATGTTTTGATCTGCCATGTATGAAAAGACTTGTCGAAAAGTAGTCTCTTCTGCA
ATCAATTTGAGCTCGGATCCTGAGGCAAATGATATAAACTCTTCTTCATTGTCTGGTTTCGAA

GACAAAAATTGAGAATATGCTTTGTATAATGTCCTGCTTGTGTTCATATCCTTTAAGGGTATC
TTTGTGTTCATCATCATCTCTTCAGCATACTCTATGCCGGTGATATTGAATGTGACATCTTTG
ATTGATGGTATTATTGATGCATCAAATGAATAGCCTTTAAGCTCTTTAGGTCGCGTTTTTAGA
TAACCCAATATTATCTTGATTGTGGTCATGACGTATTGAATGTTGATGTTATTGTCCTCACCA
TGAAAATATGATTGCATGGATTCAATCCCATTGAATTCATATCGACCAGACCAATAATGAGA
AGTTAAATCTCCGGTCCTGGATTTAAATCCGCCTGAATTAGCTCTGTGAAAATATCTGCCTCC
AACGGGAGAAACAACACCTGATTCTAGATCTGTGTACCGGGCATCGGTGAACGTTGAAAGA
GTCATATCACATACTGAGATTAATGAAGTCGCTTCAAGCTTAGAGACTTTGGAGAATTTGTT
TAGCTCCATCAAAATCCACTTGGTATATCTCACTATTTCCAGAGCTTGGAAAACTAATTTGGA
CTGAAACATACCTGCGATTCCTGCCTGTCGATCAAATTTGGGTGCATTAACTGCAGTCTTCAT
TGGTGGAAGTTTCTTTGGCCCTGTTTTGGTCATAGTTTCAGGTCCCCTCATAATTACACTGAA
GAGGTCAAAACCAGGTCTTATTTCGATAGCACCAAGTGTTGGTATTTCTTCGATTTTCAAGA
ATGACATTTCAACCTCTTCTAATTGTATCCTCTTTTTATTCACTGTATGGAAAGTGTATTTCTT
GATTTTGTCCCGGTATAGAACAAGGTCATGATAGTTCATTGTATTGTGTTCTCCAAATTCCAT
TTGCAGGTTGTGATTGGACTTCCAAGCCTTATTCCAGATTTTGGTATGCAATCTTTTGCCGAT
CATCATTAGAAATGTTGAAGTGTTATCGACTTTGTTGCAAAGATTGGTGATGAAACTGAATA
CAGATTCTTCAAAATACTTACTTGCTATTCTATTTGACATGCAATACTTGAAAGAGTGAACA
AGTCTTAGCATCATTTCTTCTTCATCTTTTCTGTTGTCTGCATTAAACAAATCTTTCAAATCCT
TGTTTTTGATGAATTTTGGTAAATGCTGTCTAAGCATGTCTTTCAGCATTGTTCCAGTATCAA
ATCGACCACCAGTTAAGGGAAACGCAGCGCTCACATACCTGGTTGCTTCATCAGATAGCTTA
ATAAGTCTTCCAATCTGTTCACGTAGATTGTCATTCAAGAAAGATCGAACGTAGGTTATTCTT
TTTATTCCTGATATAGAAAAACCACTCGTCACAGAATCAGGAATGGATGACAGTGAAAAAC
CGTACATTGTGTAAGGGAGATAAAAAAAGGCCAAAAAAAGAGATAAGAGATCTTCATTCTT
GAGATGCATGTGAATAAATAAGAGGTCGTACATTTCCAGTTTAGGAAACTTGCCCCAATTTG
TCAAATCTTCGTCAGATTTGATCATCGACATAAATTCATCAAGTTGCATCTCTTTTAATCCAC
CTTTCACCTTATCGAATCGATGTCGGTATAGACCTGCAAATCGGGAGTCCATGTTCCACTCCA
ACTGCATAGATAATTGTATCAAATCTTTAAAGATCACAGTGAGGGCACGATAGTTTCTCAGT
CTTACTTGAAGCATGTGATTGTCAGATTTTTCACATGCTGCTGTGAATCCTGAATCTATTAAT
TTTACACATTCGAGATTATCACCCCACAAGCGAGAACTGTTCTGGCTGATACCCATCATTTTC
TTGTAATTGTGCTCAATCGGATTATCTCTCACGTAATGGACCTTTAGCATGCTGCAGCGAGA
ATTCGTAACTTGTGTTTGTGATAACTTAACTATCAATCCCATTCTCATGCTAAAATTTTGAAT
GTCTTCAACCATGCTATCTAATTTTTGAGGGGTGGCATCTTTCATTATGAACACAGCACATGA
GTCATCACTGTAAGTGGTTGCAATCTGACGATTTATCTTGAAGTCAGACAACATTGCTTCAA
GCATAAGCTGCGATTGAATTCCCCACAGTGTTCCAAGCCACCCTTCAATAGCACCTTTCTGCC
CGTGAGAATGGTAAACGTTATCTGAATAGGTGTTTTCAGAAATTACCTCGATATTGTTGAAT
GCATCTACAATCGAAACGAATTCTTTGTATTCTTCAGACCCATTAACGAAACCATACATGTT
AGCAATTTCTTCCATGATGAAAGATGTGTTCTCTGGTCTTTGTGAAGTATTATGTCCAGAGTA
ATCAAGGAACAATGAATAAGCATTGTCTTCTCTTAACATTGAGGACATTCTATACATGATGT
CACGCCTTTGATCTTCAGTCATTGTCATCATCTGACCTGGCATCATTTTCATTGCTCTTTTTGC
CATTTCCATTATCTTAGACATCCAGGTTTTGAGAGAAAATGATGCTATGCCATAAAATCTCG

GTGCAATCTTCTGTTCCTTTTCTTTCTCGACCAGTCTAACAATGATATGAGATTTGGATATAT
TTATTTCTTTTCTGTCCTTAATTTCCAATTTTCTCAAATTATTTTGTCTCTCAAAAGTGAAGAG
ATCAGAGGGCTTGTCCAAGGTTTCCTTTTGGAGGTAATAGATCAATTCTTTGACTGAATTCAC
TCCATTATCATGATAAGTATCTGTCGTGCAGGCCTTGTCTATAATGCGACTCTCCAGATTCAT
CTCATTCCCTGGTTCAAGCATCTTCCCAAGTTTTACAGTCTTGTAGTATGAAGAATCATGTGT
GATTTGTTTGTTGTATTTCCCTCCGCTAGCCATCACATCTAGCTCGTATTGAAGATCCGCAGG
CAACATTGTAAGTTCAGGCATTTTGTTGTTCTTCTTAATGTAACTTGTAACCATCTTCATTTTA
AACATTGACCTCAGACGCTCTATGACATTTTGATCGACAGGGTGATCACGATTTGTGCGTTG
AGTATATTTCCTCAGCCCTTCTTCATGAGATACTTGAGCATAATGGAGAGTCTTTCCAATGCA
AGACATCTTTACACAAGTGGATTGTGTAGATGACATAATGATCTTGTAAATTTGTTCTGCATG
TTGGGGTGATGCAGATACAGTCTCGTATATATTATCCAAGACTGGCATTCTGGAACAGGTGG
ATTTCAGATCACTGCCTAATAGACAATTTGATTCATAAAGAGAAGCCAGGGTTGCTCTCTTTT
TGGTATTGCTGATTTTGTTCAATGATAAAAGGTAATCAACAAATGGTCTCTCTTCTTTGGATG
CATGATCTCTGATGACATTCAATGTTAATCCATGGTCTGCAAGTGTCATTAGATAGTCGATGC
CGCTGCCAGGCATCAAGTTGGTGAAGTTCCCATCTGATATCGCTGATATCGTCCTTGACTTAT
ACAACTCAATCTTATGACCGCCCATGATAATCATTCTTGCATGAATCTGTCCATGGGGAAAA
ACTGTTACTTCAACCCTCTTTCCTTCGGTAAATAACCCTGAGGATTCTGTTGACAAACCCTGC
ATTTTCTTGCTTGCATCAATTTTCACTTTATAGTTGATTGATTCTATGAAATCAAGAACCCAT
GCTGCCTTCAATGTTGATTCAGTGAGATCAACATTGTGATCAGTATCCAATGATATATTCCTG
TAGTTTTGTGACAATTTGTTGGATTCAAATTGTTCAAGCCTAGCTGACATTTTCCACAGAGTA
GATGCCTTCACTGAAGCAGTCGCAGCTGATAACAGCTGATTATAATTATCAATGACTGACCA
TTGACTTGATATACACTCAAGATTCCATTGTTTGATAAGAGTTCTCAAGATCATTAGATCTGT
ACGTTGCTTATTGGACCATTTCTCTGGATATTTTGAAGTTTCGGAAAAGAATCGTAAATTCTC
TTCAGAGAATGTGACAAAGGGAAAATTGATTTTATCGATTTGTTCTGGAGGGGCCTGATTAA
CCTTGGGAGGACGAAGATCAATATCTTCAGAGAAATGATTGAACATTTGAGGGAATTCAAA
GGTCTCTTTGTCTGGCTCAAGCCTCGCTTCACTGGACTGACTCTTAATAAGCAAATCATGAGA
CCCTCCAAATTCTTCTGCAATCATGTATAGTGTTGGATGATGATCTGAATTTGCGCCGAGGCT
GTTCAGATGTTCGATGCAATGCTTTTTGCTTAGCTCTGCATCTTCTTCATGATCCTCGATTATA
GGGGTTTGGTATTCATTATCGGAAACATCAGAATCAGAATACTGGAATTGCTCATCAAGAAA
TAGGGTATCGAGTGGAGTTAAGTTGAATCCTGTATCATTGGCGTCTGATTCATCTGCCCAAG
ACACTGACTTAAATTTCTCCATGATTTTATGTTCACATTGCTATTTTATTTAAAACGATTTCTT
ATAAAGATCACTGTGACACCGTATAAACAGTGTCATCAACTTTTAAGAAAATCTGATTTTCT
TGCGAAGTCAGATCTTCCAAGACCGTAGTGAGAGAATTAGTTAACACAAAATTAGTGTTGTA
TTTGTCAAGATATATCTGAGTTTTGTGCACCTTGGTTACCAAAACTACATGATGAGTGAAAA
CTCGATCACTGGTTACTGATATCAGAGGTATGTATTTCAAAATTTTTGCTGGGGTAATGGAA
ACAGACATGACTTTTGCACATGCCTGAATATAATCTAAATTCTGTTTCAGCTTGATTGGTGCA
TGCTGACACGGTTGGAGTATAGCCATTTGTCAAAGTATGAGTTTTGTAAAAC
>Seq55 [organism=Lepidozia ophiovirus_tri] RNA2 genomic sequence
TAGTAAAAACCCACGAAAACCCTTGCAGGCATGGTACCGAGGGCTAAAAAGCTTGTCGGCC
ATTGATCGTATCACCCTGTCGATTTTTCTTAAAACGAAGAACTGACAAGGTTCATCATCACTC

AACGGTCATAAAAACAATCCCACGCGGCATCACAACTACTGTAATTATCGGTATACTCAGAC
CAGTCATATCCTTCATCGTCATTATAGTAATCATCGTCTGCATAAAAACCAAACAAAACATA
GTATCTTTCAAATTCATCCAACTTGATATACTCACATCGACACAAGTCAACCGAATTGCACT
GCGGTGTAACGTTACAATAGGTGAAACAGTCATGGCTGATAGACATGATGTTAGCGTCATCC
ATAATTGTCAACTGATCTTATACAAATTTCCCTTCGTGAGCTTAGCACTGATCTTCACCTTAT
TTTTCAGGGTGATTAACCATTCCGTCGGGTTAAGTTCCATGCTTGCTATGTTGCCGGATCTGT
GTAATGATCCAAAAGATAACAGATTGTATGTCATGTCTTCTGACTCAAAAACATCACTTAAT
TGGGTTAACACTCCTCCTATGCTAGCATAGTACTGCTTGTGCATGTATCCTGTTATTTTCTCA
CCATTTGCTAAGGTGATTTCATCTTTTCCTGAAATCTTCTTGGACTGTATTACATGATGAGAT
GCTCCAGAATCAATGTACCAGGTCGAGAAATCATGATCCTCATCATTCCTCAGTGTAGTAGC
CATCATCACGATCACTGGAGGTGCCATTTGTGGTTTTTCTTCATGGTGAGGTTCCATGTAATG
ACTGTTGGTAATTCTGGGGACACCTTCTGGGTCTTTTATTCCATCAAACTTGAACCGTGATTG
CTGTTTTTGGACTTCATTGTTCCTGTTCATTATCTGTCTTTTTGCACGTTCTTCTCTCTTTTTTA
GCTCGTCTTCTGTTCCTGCTTTGATTGCTTGCAGTACATTTTTGTAGTTGAGATCTTTTGGCAC
TTGGCAGTCTTCCAAGTAATAACCAATTGGTTCACGAGTTACTCGAGTAAAGGCCTGTGAAC
CCGATTCAAGCTTAAAGAACAGGAATAGCATGCCGTAATCTCGACCTTGCACTGGAATGTTG
GAAGCTTCTGCGTTTATCTGAATGTTCTGAGCATCTTTCTGTTTGATGAAATAATTCATGGAT
AATTGGAATCTGCACATGTATGATGCATTCAAGGAAACAGTTTGAAGCAATTGTCTTTTTTG
AGGACACAGTGAAACGTCTCTCAGGCTCAATTTCAGTTTAGGCTCTCGAGTTTCTTCAAAAC
CGGCTGATTGTCGAGGGAACATGGGAAGATATATTCCATCAACGGCATGAAAACTAACAAA
TTGGTTTCCACTTACTCCAGGAAGACTTTGTTTGACCGTGTTCCATGCCTTCTGGAGATTACC
GATTTTCAAGGTCTTGTTTTCTCGTAGGTCCGTGAGCTTCATCGACATGACATGGCTGCTAGA
ATAAACCGTGTTCGCTGTGAGATTTGCGAAATCTTGCACCATATCAGTGATCCCTTGTTCCTC
AAGCTCTGGGTCACTTAAAGTCATGAATCCCTGAAACCCGGAATCTAGCAATTCCTCTGTCC
CTTTCTCAAATCCAACATGTGCAGTGATCTCCTTTGTTCCGTTGTACTGTTTTCCTTTACCAAT
CATATCGAAAACAGGCTTCCTCAGAGATTCAGAGCGATCCAACCCTCTTGAACTCATGCCAG
ATAGATCCATTGCCAAGAGTGATTTTGTGATCAAAGCACTGACTGCAACTTTGATTCACTTG
CTGTATCAAGATTCTCGATGATCTCTTGTTCACTTTAAGTTTGTGTGATTACATTAGT
>Seq56 [organism=Lepidozia ophiovirus_tri] RNA3 genomic sequence
AAATCACAAGTGATCACGGGATCATTATATGATAAAATTAACTCTTCCGTCAAGGCTTTTAT
TAAAGCGAACACTTGAAAAAGTCATCCACATCGAGTTGTTCAATCAAAACACTTGTTGACCG
TCTCGCGTGTTAACCCCATGAAGTTTCCATTCTCATCGTTGAGAATCGGCCAAGTTCGCTGAT
TGAGAGCATCATAAGACCCCATGATATTCTTATCCTTCAGGAACGATCGCATGTCCTTGTCCT
GAAAATGCTTTACCAAAGCTAATCTCCCACTCTCAGTCAAACTGTGAAGAATAGCAGAGGTC
ATCTTCATCGTGAAGCCAACCGGAACGCTCTTGTTCGGATTCAGAGGGTGTAATTTTAACTG
AGCTTGAAAATTTCCACTTCTTGAGCGAAGAGCATCCACCAATTCATGAGCTTTAACGATTTT
TTGGTGATTCGCCAATTCATCCGCTGATAACTTATCATTTGCATCAACCTTTGATGAAGATTG
TTCAAACAAAGATGCGTGCTCAGCGTACTTAACCACGCGATTCCCTGCTACTGCAAGTTTCA
TTCTCGATACATACTGTGATGGAAGTCCATCAATATTGACATTGAGCAGAGACCGAGCAGGA
AATTTGCGGGTAGTAGTACAAGAGAGCAATTGAGCGACTTCAGACTCAGTAGTCATTTCATC

ATGAAACAAATTTTCTCGGAGAAGCTTGATCAAAGGCTTTTGATTTGTTTCATCCCCAGAAT
ATGTAGGCATGTTACCTCTGTTGTAGATCAGAACAAGAGCGGCGATAAAAGCCTTTCTTGCA
TAATCAACAGCAGCAGAGACTTCCTCCATACTGATCTCGTCTATTTCTTCAACATTGAACATA
TATAAAGCTGATTGATCTTCATGATAGGATGAATATAACACTGCATCCAGAACTAACGTGCC
AGCAGCTACCAAATTGTGCACTGATACTTTAGCACCCTTTAATACTCTCACATGAACCTCTCC
CATGTTATATTTCTCATCTTCTGGTATTGTTTGAGCAAATTCATTCATTTTTTCCACCAAGGTT
TTAATTTCCATCTTGAATGCCGATGATGCAAGCATTGCTTCGATGTTCGCAGCGGTTACCATG
GATCCAGAACTTTGTCCCTTTTCTGGCTTCCCCATTCTGGTGACAAAGGTTTTTACGGTACTC
TCTTGATCCTTCTTCGGAGAAGTAGAGCGCTGGGCAATAGTTTCAGAAATCTTTTCAAGCTTT
GACTGAGCCTGTCCGGTCGGCAAGATTTCGACAGTTCCCAAGCCAGTGTTTCGGATCAGTTT
TCCTTTGTTGTCTATTGTCACAGATATGTCTCCATATGCCGAAATCAAGTCGTTGAGCTCCTT
TTTCTTCTCCGGCTTGATACTGTCGTCATTTTTCAGCATACCGAACTGCTTCAGGATTTCCTTC
TCCCCATCAGTAGCTTCGCCCTGGGACAATGAGCGAAGAGTCTTGATGAGAATTCCAGACTG
AATAGAGCCGGCCATTTCTCACAGTAGAAAGTTTCTTTTGTGTAAATCACAAGTGATCACGG
TGATAACTTTAAGTTTG
>Seq57 [organism=Lepidozia ophiovirus_pli] RNA1 genomic sequence
TTAAAAACTGTTCAATTTTTATCAGCACCGCTGTTAACCGTGTAAAAAGTGCACTTGAAAAA
GTGCAATCCAGTTTCAGGATCATACTTACCAATCTTCGTCAAAATCCATTCCATCATCTTCGA
ATTCCTCATATTTCAGTGCCATATTCCTTGCCCTTTCCAAATTTTGCAATCGCTCCTGTTCTTC
GTCCTTGAATTCATCATATGTTGTTGTCAACATATAAGATTGTTCAATCTCTTGATTTTTCTTC
ACGTTTTCATCCCAATCCTTAAATAACCCTTTGATAAAGGCATCTGTTTTGATAGGTTCATCT
ATTGTAATTTGTTCTACATTTTTATCCAGGTCCATTGAATTAATAAAATTAAAGAAATTATCA
TTGTCTGCATTCGATGCTTCAATAAATTCTTGAATAGTTTTAAACTGCGCATAGTGCTCTCTG
AATTTTTGCAGATCTTCATCATTCATCCTTCCAGCTTTAACTTCCGTTTTCCAAATATCCTTCA
CAAAAGACTCGAGATCTTGATTGTTTGCTGTTATGTTGATGTCTGTGACTTTCTTGACAATGC
GATTAATCCTGTTTAAGATTAATTTGTAATTAATGATATTGTTTCTATTGTTTTTCGTCTTCAC
TCTCATGATGGTGCTGAGATTTACCCTTTCAAGACCTGAGATAGCTATGAGACCCTCTTTGGT
TCTTCCGGACTTCCAAGAGTTTATTACCTGAGTCGTGGTTGTAAACAGATTATCAATGGTTCT
TCCGACACCTGAATTACGTAGAGATTCAATGGATATCATAGGAATGATATCATCAGAACTGA
TCTTATCATAATCCTTTGCTAGGTCTAACAAAAACATCCATAAATCCTTTGTAATCGGTAATG
TATTCTGATCTTGTATATGTTGAACAACCATAGTTAGATCAGAGAATTTCATCTCTTCAAATC
CTGTTATTACAATCTTTGGCAAAATATCCGCTTTCTCATTTATCACTTCTAATCTAGTTTCTCC
TGTTCTTCTATAATGCTTCATACAAGATACAAATGAACCAAATCCAATGTCAAGATCAGATG
TGATGTAGTCAAGGTATTCAGTTTTAATTCCGGGTATGTGTTGCAAACCCTCAGCACTTGTGC
AGAGTAATGCTCTTATGTCATCAGAATTCCTTACATTGTTTTTAAGGGACTGAGGAACCTCA
ACAATCGAGTCCTGGGTGTGACTCATCAATGAATCTAACAATTCATCATCTGTTATGAATTCT
TCTGTATGATCAACAACGTCTTTCCCAATATGACTCAGCATGGGTTTATGTAGGAAGCCTAT
GTTTCCTTGTGACAAGTATTCTAATATCAGGTTACCAGCAAATCCTTTATTGCTACGCTTTCT
GGAAACAATCTTTCCTTCCTTTTCTTTTGCTTCCAGTGTGAGGTACAAGACTCCACTGCTGTT
CTTTTCTGGTATTAAGAGTTCAATGTTGCAGTGTTGACCCATGACATCAATCAGTTTTTGAGT

TATGATTGATGATACTGCATTCCACCTGAGAACAATCTTTTTCTTGATTGCAATCATTTCTGA
TATGCCTTTAGTTAGCAGGTCCTTCTTTCCGGTAAAGTGCGATACATCCAGAAGAATAATTG
AGTGATCCATGTAATCCATAAAATTTCTCTGGAGAAAACATTCCAAGAAGGTTTCTTCTCTT
TCATTCCCTTGATTCTATTTATTAGGTTGAATCCATCATTCCGAGAGACTGATGTATGGTTCA
CTTTTGCTTCCGTTAATGCTAAGTGGAAGTCTCCTCTTCCGGCACAAAGATCTGCGACTGTGT
CCTTTTCAGTCAAAAACTGGGAACCCTGTAGACACTTGATAACTCCTCTTGCTGCAGGGTAT
ATGTCAGATTTGGTGGGAGATGAAAGGTTTTCAACGGTGCAAGACAATGCACAAATGTATTC
GAAGGCCTTGATTGCAGCAACACATGCATCTTGATCTTTTAAGTCATACATATCGATACCGA
AAGAATAATAATTGACTGAGCCATAAACTTTCGATTCATCTTTTTCAATGTATGAGGATCTCT
CTGTTTTGAAATAATCATAAAGTTTATACTGCTCAAAAACTTTATGATTCAATTCCATCGATA
TTTCATCTGAGCATTTTTCAACTTCAATGACCGTGTAAGACATAGTCGGGAAACCATATATC
AAGATTTGCGAGATAGCCCTGTCCTTTATATGGATATGATGAGTTCGGTTCAATATTGCTTTA
AAGCAACGAACACTATTGATCTTAGTAGCAACTGTGTTTATGTGCAACGTACTTCTACCATT
GTTTGTCTTAAGTGTGTATATGAGGCCGCAGGCAATAATAAAAAGTCTCAGTAAATTGTGTC
TGTAGTCCATGGAAAAACCTTTTGTAGTCATTTCTTTTGACCACACTCGTCCTTGACTGTCAT
GTTTAAAGAGTTCTTTGTACAGTAATCCTCGTATCATGGAAGCACTTGGTGACTCCCTTCCTA
TGATATTTAATGAAGAATAGAAATCATAGAATTCCTTGAAGAATTGTTCTTTTGTAGTGAAG
TTGTCGTAGTTTAAAAGCAATTGTCTCAACACATCATCTGATATGACTTCCGGTGTTATTATG
TTCTGATCTGCCATGTATGAAAAGACTTGTCGAAAAGTAGTCTCTTCTGCAATCAATTTCAGT
TCGGATCCTGAAGCGAATGATATGAATTCTTCTTCATTATCTGGTTTAGAAGCCAAGAATTG
AGAATATGCTTTGTATAATGTCCTGCTGGTGTTCATGTCTTTTAGGGGTATTTTAGTGTTCAT
CTCCATCTCTTCAGCATACTCTATACCGGTGATATTGAATGTTACATCTTTAATTGATGGTAT
TATTGATGCATCAAATGAATACCCTTTGAGCTCTTTAGGTCGCGTTTTCAGATAACCCAGGAT
TATCTTGATAGTAGTCATGACGTATTGAATGTTGATGTTATTGTCTTCACCATGAAAGTAAGA
CTGCATAGATTCAATTCCATTGAATTCATATCGACCAGACCAATAATGAGAAGTTAAATCTC
CAGTCCTAGATTTGAAACCACCGGAATTAGCCCTGTGAAAATATCTGCCTCCAACGGGAGAG
ACAACACCGGATTCTAGATCTGTGTATCTTGCATCAGTGAATGTCGAAAGAGTCATATCACA
TATTGAGATTAATGAAGTTGCTTCGAGCTTAGAGACTTTAGAGAATTTGTTCAACTCCATCA
AAATCCACTTAGTATATCGCACAATTTCGAGAGCTTGAAAAACTAATTTGGATTGAAACATA
CCTGCGATTCCTGATTGTCGATCAAATTTGGGTGCATTAACTGCAGTTTTCATTGGAGGAAGT
TTCTTCGGACCCGTTTTGGTCATGGTTTCAGGTCCTCTCATAATCACACTAAAAAGGTCAAAA
CCGGGTCGTATTTCAATAGCACCGAGTGTTGGTATTTCCTCAATCTTCAAGAATGACATTTCA
ACTTCTTCTAATTGTATCCTCTTTTTATTCACTGTGCGGAATGTATATTTCTTGATCTTGTCTC
GGTATAGAACAAGGTCATGATAGTTCATTGTGTTGTGTTCCCCGAATTCCATTTGCAAGTTGT
GATTGGATTTCCAAGCTTTATTCCAGATTTTGGTGTGTAATCTTTTGCCGATCATCATTAGGA
ATGTTGAAGTATTGTCAACTTTGTTGCAAAGATTGGTGATGAAGCTGAATACAGATTCCTCA
AAATACTTACTTGCTATTCTATTTGACATGCAATACTTGAAGGAATGAACAAGTCTCAACAT
CATTTCTTCTTCATCTTTCCTGTTGTCAGCATTAAACAAATCTTTTAAGTCTTTGTTTTTGATG
AATTTTGGTAAATGCTGTCTGAGCATGTCTTTGAGCATGGTTCCAGTGTCAAATCGACCACC
AGTTAAGGGAAACGCAGCACTCACATACCGTGTTGCTTCATCAGATAGCTTGATAAGCCTTC

CAATCTGTTCGCGTAGACTATCGTTCAAGAAAGATCGAACATAGGTGATCCTTTTAATTCCT
GATATAGAAAAACCACTTGTTACAGAATCAGGAATGGATGACAATGAAAAGCCATACATTG
TGTAGGGAAGATAAAGAAAGGCTAAAAACAGAGATAAGAGGTCTTCATTCTTAAGGTGCAG
GTGAATAAACAGAAGGTCATACATTTCCAGTTTAGGAAATTTACCCCAATTTGTTAAATCCT
CATCAGATTTGATTGTGGATAGAAATTCATCAAGCTGCATCTCCTTTAATCCACTTTTTACTT
TATCGAATCGATGGCGATACAAGCCTGCAAATCGAGAATCCATGTTCCACTCCAACTGCATC
GATAATTGTATCAAATCTTTAAAAATCACAGTGAGTGCACGATAATTCCTCAGTCTTACTTG
AAGCATGTGATTGTCAGATTTTTCACATGCTGCTGTGAAACCTGAGTCAATTAATTTCACACA
TTCGAGATTATCACCCCACAGGCGAGAACTGTTTTGACTGATACCCATCATTTTCTTGTAATT
GTGTTCGATCGGATTATCTCTCACATAATGGACCTTTAGCATACTGCAGCGAGAATTAGTTA
CCTGTGTTTGTGATAACTTGACTATCAATCCCATTCTCATGCTGAAATTCTGAATGTCTTCAA
CCATACTGTCTAATTTCTGAGGAGTGGCATCTTTCATTATGAACACAGCACATGAATCATCA
CTGTAAGTGGTTGCAATCTGGCGATTTATCTTGAAATCAGATAACATTGCTTCAAGCATGAG
CTGCGATTGAATTCCCCATAGCGTTCCCAGCCACCCTTCAATAGCACCCTTCTGTCCATGAGA
ATGATAAACGTTATCTGAATAAGTGTTCTCGGAGATCACTTCGATGTTGTTAAATGCATCTAC
AATCGAAACGAATTCTTTGTATTCTTCAGATCCATTAACAAAGCCGTACATGTTTGCTATCTC
TTCCATGATGAAAGATGTGTTCTCTGGTCTTTGTGAAGTGTTGTGTCCAGAATAATCAAGGA
ACAATGAGTAAGCATTTTCTTCTCTTAACATTGAAGACATCCTATACATGATATCACGCCTTT
GATCTTCAGTCATCGTCATCATCTGACCTGGCATCATTTTCATTGCTCTTTTTGCCATTTCCAT
TATTTTTGACATCCAAGTTTTGAGAGAAAATGATGCTATCCCATAAAATCTTGGTGCTATCTT
CTGTTCCTTTTCCTTCTCTACCAGTCTAACAATAGTGTGAGATTTGGATATACTGATTTCTTTC
CTGTCCTTAATTTCCAGTTTCCTCAAATTATTTTGTCTCTCAAAAGTAAAGAGATCAGAGGGC
TTGTCCAGAGCTTCTTTTTGTAGGTAATAAATCAGTTCTTTGACCGAATTCACTCCATTATCA
TGATAAGTATCTGTCGTACAAGCCTTATCTATAATGCGACTCTCCAGATTCATCTCGTTTCCT
GGTTCAAGCATCTTACCAAGTTTCACGGTCTTGTAATATGAAGAATCATGTGTAATTTGCTTG
TTGTATTTACCTCCGCTGGCCATAACATCTAACTCGTATTGAAGATCCGCAGGCAACATTGA
AAGTTCAGGCATCTTGTTGTTCTTTTTGATGTAATTTGTGACCATCTTCATTTTAAACATAGA
CCTTAGACGCTCTATGACATTCTGATCAACAGGGTGGTCACGATTTGTACGTTGAGTGTATTT
TCTCAACCCTTCTTCATGAGATACTTGGGCATAATGGAGAGTCTTTCCAATGCAGGACATCTT
TACACAAGTGGACTGGGTTGATGACATAATGATCTTGTAAATTTGTTCTGCGTGTTGAGGTG
ATGCAGACACAGTCTCATATATGTTATCCAAGACCGGCATTCTGGAACATATGGATTTCAAG
TCACTACCTAATAAACAGTTTGATTCATAAAGAGAGGCCAGGGTTGCTCTCTTTTTAGTATTG
CTGATTTTGTTCAATGACAAAAGGTAATCAACAAATGGTCTTTCCTCTTTAGATGCATGGTCT
CTGATAACATTCAATGTTAATCCATGATCCGCAAGTGTCATAAGATAGTCGATACCACTGCC
AGGCATCAGATTGGTGAAATTTCCATCTGATATCGCTGATATCGTCCTTGACTTATACAACTC
AATCTTGTGACCGCCCATGACAATCATTCTTGCATGAATCTGCCCATGAGGGAAAACTGTTA
CCTCGACCCTCTTTCCTTCGGTGAAGAGCCCTGAGGATTCTGTTGGCAAACCTTGCATTTTCT
TGCTTGCATCAATCTTTACTTTATAATTGATTGATTCTATGAAATCAAGAACCCATGCGGCCT
TCAATGTTGTTTCAGTGAGATCTATATTGTGATCAATGTCCAATGATATATTCCTGTAGTTTT
GTGACAATTTATTGGACTCAAATTGCTCAAGCCTAGCTGACATCTTCCACAGGGTAGATGCT

TTTACTGAAGCAGTTGCAGATGACAAAAGCTGATTATAGTTGTCAATGACTGACCATTGACT
TGATATACACTCAAGATTCCATTGTTTAATGAGCGTTCTCAAGATCATTAGATCTGTGCGTTG
CTTATTGGACCATTTCTCCGGATATTTTGAAGTTTCAGAGAAGAACTTCAAATTCTCTTCAGA
GAATGTGACAAAAGGGAAATTGATTTTATCGATTTGTTCTGGAGGAGCTTGGTTGACTTTAG
GAGGACGAAGATCAATATCCTCAGAGAAATGATTGAACATTTGAGGGAATTCGAAGGTTTC
TTTATCTGGCTCAAGCCTCGCTTCATTGGACTGGCTCTTAATAAGCAAATCATGAGACCCTCC
AAACTCTTCTGCTATCATGTACAGTGTTGGATGATGATCTGAATTTGCGCCGAGGTTGTTCAG
ATATTCAATACAGTGCTTCTTGCTTAGCTCTGCATCTTCTTCATGCTCTTCGATTAATGGGGA
TTGATATTCATTATCTGAAGCATCAGAATCGGAATACTGGAATTGCTCGTCAAGAAAGAGAG
TATCAAGTGGGGTTAAGTTGAATCCTGTTTCATTATCATCTGACTCATCTGCCCAAGACACTG
ATTTCAATTTCTCCATGATTTTGTGTTCACATTGCTTTTTATTTAAAACGATTTCTTATAAAC
TTCACTGTGATACCATATAAACAGTATCATCAACTTTTAGGAAAATTTGATTTTCCTGTGAGG
TTAGATCTTCCAAGACTGTAGTTAGAGAGTTAGTTAACACAAAATTAGTGTTATATTTATTAA
GGTATATCTGAGTTTTGTGAACCTTAGTTACCAAAACCACGTGATGAGTGACAACCCGATCA
CTGGTCATTGATATTAGAGGGATGTATTTTAAAATTTTTGCTGGGGTAATGGAAACAGACAT
GACTTTTGCACATGTCTGAATATATTCTAAATTCTGTTTCATCTTGATCGGTGTATGCTGACA
CGGTTGGAGTATAGCCATTTGTCA
>Seq58 [organism=Lepidozia ophiovirus_pli] RNA2 genomic sequence
AAGCCAACCAAATTGCACTGCGGTGTAACGTTACAATAGGTAAAACAGTCATGGCTGATAG
ACATGATGTTTGCGTCATCCATAATTGTCAACTGATCTTATACAGATTTCCTTTCGTGAGTTT
AGCACTGATCTTCACTTTATTTTTCAGGGTTATTAACCATTCTGTCGGGTTAAGCTCCATGCT
TGCTATGTTGCCAGACCTGTGCAGTGATCCAAAAGACAACAAATTGTATGTCATTTCTTCTG
ACTCAAAGACATCACTCAATTGGGTTAGCACTCCTCCTATGCTGGCATAGTATTGTTTGTGCA
TGTATCCAGTTATTTTCTCGCCATTTGCTAATGTGATTTCGTCTTTTCCAGAAATCTTCTTGGA
CTGTATCACATGATGAGATGCTCCTGAATCGATGTACCAGGTTGAGAAGTCATGCTCCTCGT
CATTTTTCAGTGTAGTAGCCATCATCACAATAACTGGAGGAGCCATTTGGGGTTTGTCTTCAT
GATGAGGCTCCATGTAATGTGTATTAGTGATTCTGGGAACACCTTCTGGATCCCTGATTCCCT
CAAATTTGAACCGTGATTGTTGTTTTTGAACTTCATTATTTCTGTTCATTATTTGCTTTTTTGC
TCGTTCTTCTCTTTTCTTCAGTTCATCTTCTGTTCCTGCTTTGATTGCTTGCAGAACATTCTTGT
AGTTAAGATCTTTAGGCACTTGACAGTCTTCAAGGTAATAACCAATTGGTTCACGAGTCACT
CGAGTAAATGCCTGTGATCCTGATTCAAGCTTGAAAAACAGGAATAGCATGCCGTAATCTCG
ACCTTGTACTGGGATGTTGGAGGCTTCTGCGTTGATTTGAATGTTCTGTGCATCTTTCTGCTT
GATGAAATAATTCATAGATAGTTGGAATCTGCACATGTATGATGCATTCAAAGAAACGGTCT
GAAGCAATTGTCTTTTTTGAGGACACAATGAAACGTCTCTCAAGCTCAATTTCAGTTTGGGCT
CTCGAGTTTCTTCAAAACCGGCTGACTGACGAGGGAACATGGGAAGATATACCCCATCGAC
AGCATGAAAGCTAACAAACTGATTTCCACTCACTCCAGGAAGACTTTGTTTAACCGTATTCC
ATGCTTTCTGGAGATTGCCAATTTTCAGGGTTTTGTTTTCTCGTAAGTCAGTGAGCTTCATCG
ACATAACATGGCTGCTAGAATAAACTGTGTTTGCAGTGAGATTTGCGAAATCCTGCACCATA
TCAGTGATTCCTTGTTCTTCAAGCTCGGGATCACTTAAAGTCATGAATCCCTGAAACCCGGA
ATCTAGCAACTCCTCTGTTCCTTTCTCAAATCCAACATGTGCAGTGATCTCTTTTGTCCCGTTG

TACTGTTTTCCTTTACTAATCATATCGAAAACAGGCTTTCGCAGGGATTCGGCGCGATCCAAC
CCTCGTGATTCCATACCAGATAGATCCATTGCCAAGAGTGATTTTGTGATCAAAG
>Seq59 [organism=Lepidozia ophiovirus_pli] RNA3 genomic sequence
TGAAAATGGATCATTATATGATAAAATTAACTCTTCCGTCAAGGCTTTTATTAAAGCGAACA
CTTGAAAAAGTCATCCACATCGAATTGTTCAGTCAAAACACTTGTTAACCGTCTCGCGTGTT
AACCCCATGAAGTTTCCATTTTCATCATTGAGTATCGGCCAAGTTCGCTGATTGAGAGCATC
ATAAGAACCCATGATATTCTTGTCCTTCAAGAACGACCGCATGTCCTTGTCCTGAAAATGTTT
TACCAAAGCTAATCTTCCATTCTCAGTCAGACTGTGTAAAATAGCAGAGGTCATCTTCATCG
TGAAGCCAACCGGAACGCTCTTGTTCGGATTCAAAGGGTGTAATTTTAACTGAGCTTGAAAA
TTTCCACTTCTTGAGCGAAGAGCATCCACCAGTTCATGAGCTTTAACAATCTTTTGATGACTT
GCTAACTCATCTGCGGACAATTTGTCATTTGCGTCAATCTTTGATGAAGATTGTTCAAACAAA
GATGCATGTTCAGCGTACTTAACCACACGATTACCTGCAACTGCAAGTTTCATTCTCGAGAC
ATACTGCGATGGAAGACCATCAATATTGACATTAAGCAGAGACCGAGCTGGAAACTTGCGG
GTTGTAGTGCAAGAAAGCAATTGTGCTACTTCAGATTCAGTAGTCATTTCATCATGAAACAA
ATTTTCTCGGAGAAGTTTGATCAAGGGCTTTTGATTTGTTTCATCTCCAGAATATGTGGGCAT
GTTACCTCTGTTGTATATCAGAACAAGAACAGCGATAAAAGCCTTTCGAGCGTAATCAACTG
AAGCAGAGACCTCTTCCATACTGATTTCATCTATCTCTTCTACATTGAACATATATAAAGCTG
ATTGATCTTCATGATAAGATGAATACAACGCGGCATCCAGAACTAACGTACCAGCAGCAAC
CAAGTTGTGCACTGAAACTTTGGCACCCTTCAACACTCTCACATGAACTTCCCCCATGTTGTA
TTTCTCATCTTCTGGTATTGTCTGAGCGAACTCATTCATTTCTCCACCAAGGTTTTAATTTCC
ATCTTGAATGCCGATGATGCAAGCATTGCTTCGATGTTCGCAGCGGTTACCATGGATCCAGA
ATTTTGTCCCTTCTCTGGCTTTCCCATTCTAGTGACCAAGGTCTTCACGGTACTCTCGTGATCT
TTCTTCGGAGAAGTAGAGCGCTGAGCAATAGTTTCAGAAATCTTCTCAAGCTTTGACTGAGC
CTGTCCAGTCGGCAAGATTTCGACAGTTCCTAAACCTGTGTTTCGAATCAGTTTTCCCTTGTT
GTCTATTGTCACAGATATGTCTCCATATGCAGAGATTAAGTCGTTGAGTTCTTTCTTCTTTTCC
GGCTTGATACTGTCATCGTTTTTCAACATACCGAACTGCTTCAGGATTTCCTTCTCCCCATCA
GTAGCTTCGCCCTGGGACAATGAGCGAAGAGTCTTGATGAGAATTCCAGACTGAATAGAGC
CGGCCATTTCTCACAGTGGAAAATTTCTTTTGAGTAAATCACAAGTGATCACGGTG
>Seq60 [organism=Lepidozia ophiovirus_sela] RNA1 genomic sequence
GTTTATATCAGCACGACTGATATTCATGCCAATATAGCACTTGAAAAAGTGCAATCCAGTAT
CAGGATCATACTCACCAATCTTCATCGTAATCCATGCCATCATCATCGAAGTCACCGTACGC
TGATGCAATATTTTTAGCCCTCTCAAAGTCTGCTATTCTTACCTGTTCATCATCTTGGAATTCT
TCATATGTTGTTGTTAATGTGTAAGACTGTTCAATCTCCATATTCTTTTTGACATTTTCATCCC
AGTCTGCAAATAATCCTTTTATAAACATATCAGTTTTCTTCGGTGCATCAAAACATACCTCTT
CTGTGGTTTTGTCAAGACTCATTGAATTTATAAAATTGAAAAAATTATCATCGTCTGTGTTTG
CTGCTTCGATGAAATCTTTCATAGTCTTAAATTGAGAATAATGTTCTCTGAATCTTTGTAAGT
CCTCATCATTCATTCTTCCAGTTTTCGTCTCTGTCTTCCATATTTCTCTAACAAAGCTTTCAAG
ATCTTGATTATTTGACGTAATTCTTATATCAGTGACCTTCTTGACTATCCTATTTATTCGATTA
AGAATTAACTTGTAATTTATGATATTATTCCTATTGTTTTTTGTCTTCACTCTCATTATAGTGC
TGAGATTGACTCTCTCCAAACCAGATATAGCGATTAAACCCTCCTTTGTTCTGCCAGATTTCC

AAGAATTAATGACCTGTGCTGTGGTGGTGAAAAGGTTGTCAATTGTCCTGCCTATACCAGAA
TTGCGTAGTGACTCAATCGAAATCATAGGTATTATGTCATCGGAATTGACTTTATCACAATCT
TTTGCCAAATCCAACAAGAACATCCACAAATCTTTAGTCACTGGAAGCGTATTTTGATCTTG
CACGTGTTGGACTATTAAAGTTAGATCGGAGAACTTGATTCCTTCAAACCCAGTTACCACTA
ATTTTGGTAAACTATCGGCTTTCTCATTGATTACCTCTAATCTTGTTTCTCCAGTTCTGCCATA
ATGTCTCATACACGAAAGAAATGATCCGAAACCCATATCTAAATCAGATGTGATGTATTCAA
AATATTCCGTTTTAATACCAGGTATATCCTGAATTCCCTCTGCACTGGTACATAACAATGCTC
GTATATCATCAGAATTTCTGACGTTATTTTTAAGTGACTGAGGAACTTCTACGATTGAGTCAG
GAATGTGACTCATTAAAGAATCAAGTAATTCATCGTCTGTAATGAATTCTTCTGTATGATCA
ACGACATTCTTACCCACATGACTCAACATAGGCTTGTGTAGAAAACCTATATTGCCTTGAGA
TAGATATTCTAAGATTAGATTGCCTGAAAATCCTTTATTGTTACGTTTTCGAGATACAAGAAT
GTCTTCAGCAGCCTTTGGAGCAAGTGTCAAGTAAAGAACTCCACTGCTGTTTTTCTCCGGAA
TCAGAATGTTTATTTTGCAATGTTGTCCCATGACATCTATAAGTTTTGGAGATAATATAGAGG
AGATAGCATTCCAGCGCAAGATGATTTTCTTCTTAATGGCAATCATCTCAGATATTCCTTTAG
TGAGTAGATCTTTCTTTCCGGTAAAATGAGATACATCAAGCAATATGATTGAATGATTCATA
TAGTCCATGAAGTTATCTCTGGAAAAAGCATTCCATGAAGGCTTCTTTTCTTTCATCCCTTTA
ATCCTGTTTATTAAATTGAAACCATCATTGCGTGACACGGAAACGTGTTCAATAGAATTCTCT
GTCAGAGCGATGTGATAATCACCTCTTCCGGCGCAAAGGTCAGCAATAGTATCTTTATCTGA
TATAAACTGTGAACCCTTGAGGCAATTGATCACTCCCAGTGCTGCGGGGTATATATCAGACT
TCGTCGGGGATGATAAGTTCTCTACGGAACAAGAGAGTGCGCAAATGTATTCGAAAGCCTTT
ATTGCTGCAATACAGGCATCTTGATCTTTCAAATCGTCTTTTGTTATGTTGTGAGAATAGTAA
CTGACAATTCCATAATCCTTCGTTTCATCTTTTTCGATATATGAGGATCTCTCCATCTTGAAGT
AATCATAAAGCTTATATTGTTCAAACACTTTATGATTAAGCTCCATTGATACTTCATCAGAAC
ACTTCTCAACTTCATTCACTGTGTAAGACATTGTTGGAAAACCGTAAATGAGAATTTGGGAT
ATTGCTCTGTCCTTTATATGAATATGATGTGTTCTATTCAGAATTGCCTTGAAACATCTAACA
CTATTGATTTTAGTTGCAATGGTATTGACATGAAGTGTACTCTTTCCATTATTTGTCTTCAAG
GTGTATATGAGCCCACAAGCTATTATGAAAAGACGGAGAAGATTGTGTCGATAATCCATTGA
AAATCCTTTAGTCGTAATCTCTTTGGACCATACTCTCCCTTGACTATCATGTTTAAATAATTCT
TTATATAATAGCCCTCGTATCATCGAAGCACTTGGAGATTCTCTCCCAATAATGTTGAGCGAT
GAATAAAATTCATAAAACTCCTTGAAAAACTTCTCTTTAGTAGTAAAATTTTCGTAATTTACG
AGCAATTGTTGAAGGACATCATCTGAGATGACTTCAGGAGTGATTATATTCTGATCGGCCAT
GTATGAAAATACTTGTCGGAAGGTGGTCTCCTCTGCAATAAGTTTCAATTCTGATCCAGACG
CGAACGATATGAAGTCGTCTTCACTATTTGGTTTTGATGATAAGAATTGTGAATATGCTTTGT
ATAGAGTTGTACTTGTGTTCATATCCCTGAGAGGAATTTTATTGTTCATGACCATTTCATCAG
CAAAGTCTATTCCTGTCAAGTTGAAAGTAACGTCCCTGATAGCTGGGATGATGGAAGTATCA
AATGAATAACCTTTCAATTCTCTTGGTCTTGTCTTCAAGTAACCCAGTATGATTTTAATTGTC
GTCATTACATATTGAATATTAATGTTGTTATCCTCACCATGGAAGTAGGACTGCATAGAATCT
ATACCATTAAATTCATATCTACCAGACCAATAATGTGAAGTTAAATCACCTGTTCTTGATTTA
AATCCACCGGAGCTTGCTCGGTGAAAATATCTACCTCCCACAGGGGAAACAACCCCTGATTC
AAGATCCTTGTAACGTGCATCAGTGAAGGTTGATAATGTAAGATTGCAAACGGAAATCAAA

GCATTTGCTTCAAGCGTAGAAACAGAAGAGAACTTATTCAACTCCATCAAAATCCATTTTGT
GTATCTTACAATCTCAAGTGCTTGGAATACTAGCTTGGACTGGAACATGCCTGCAATTCCTG
CTTGACGATCAAACTTGGGGGCATTTATTGCAGTTTTCATAGGTGGTAATTTTTTGGGTCCTG
TTTTTGTCATGGTCTCGGGTCCTCTCATTATAACACTGAAGAGATCGAAACCTGGACGTATTT
CAATCGCTCCGAGAGTAGGAATTTCTTCAATTTTGAGAAAAGACATTTCAACTTCCTCTAATT
GGATTTTCCTTCGGCTGTCCTGACGATATAGAAATTTCTTCACATTATCACGATACACAAGAA
TGTCATTGTAATCCATTGACGAATGATTTCCAAATTCCAGCTTCAGGTTATGATTTGATTTCC
AAGCTTTGTTCCAGATTTTGGCATGAAGATATTTCCCAATCATCATCAAAAAGGTGGATGTA
TTGTCAACCTTGTTACAAAGATTGGTGATGAAACTGAAAACAGATTCCTCAAAGTATTTGCT
TGCTATTCTGTTTGACATACAATACTTGAAAGAGTGAACTAGACTGTTCATCATGCTCTCTTC
ATCACGGCGGTTGTCAGCATCAAAGAGCTCCTTAAGTTCTCTATTTTTAATGAATTTAGGAA
GATGTTGTCGTAATAGATCTTTCAAGAGCGTGCCTGTGTCAAAGCGTCCTCCTGTGAGTGGA
AAAGCAGCACTTACATACCTGGTGGCTTCATCTGAAAGTTTAATCAGTTTGCCAATTTGGGC
ACGAATGTTGAGATTTAAGAACGATCTAACATATGTAATTCGCTTGATTCCCGAAATTGAAA
AACCGCTAGTCACAGAATCTGGAATTGATGATAGGGAAAATCCATACATTGTGTAAGGTAA
ATATAGGAATGCAAGGAATATAGATATAAGATCTTCATTATCCATATGCAGATGAATGAATA
ACAAATCATACATTTCTAATTTGGGAAATTTACCCCAGTGTGTTAAATCTTCATCACTCTTGA
TTAGGGAAGCAAATTCGTCGAGTTGCATTTCCTTCAAACCTCCCTTTACCTTATCAAATCTAT
GACGATAAAGACCAGCAAATCGGGTATCCATGTTCCACTCCAATTGAAGAGACAAGTTTATC
AAATCCTTGAACACAACTGTTAAAGCTCGATAATTTCTTAGTCTAACTTGCAACATATGATTG
TCAGACTTTTCACATGAAGCAGTGAACCCTGAGTCGATCAGTTTCACGCATTCTAGATTGTCC
CCCCATAGTCTGGAGCTATTTTGACTAATCCCCATCATCTTTTTATAGTTGTGTTCAATGGGA
TTGTCTCTCACATAATGAACTTTGAGCATGCTGCACCTTGAATTCGTCACTTGAGTTTGGGAT
AACTTAACGATTAACCCCATCTTCATGCTGAAGTTCTGGATGTCCTCTACCATTCCATCTAGC
TTTTGAGGCGTTGCATCCTTCATAATGAACACAGCACAGGAATCATCACTGTAAGTCGTTGC
TATCTGACGATTTATCTTGAAGTCAGATAACATAGCTTCTAGCATAAGCTGTGATTGGATTCC
CCACAAAGTCCCAAGCCATCCTTCTATCGCACCTTTCTGGCCATGCGAATGATAAACATTAT
CTGAATATGTGTTTTCTGAAATAACTTCTATATTGTTGAAGGCATCAACTATAGATACAAACT
CTCGAAATTCTTCAGATTCGTTCGAGAAACCATACATGTTAGCGATCTCTTCCATGATAAATG
ATGTGTTTTCTGGCCTTTGTGAAGTGTTATGTCCTGAGTAATCTAGGAAGAGCGAATAGGCG
TCTGCTTCTCTAAGCATTGAAGACATGCGATACATGATATCTCTTCTTTGATCTTCTGTCATG
GTCATCATTTGTCCAGGCATCATCTTCATGGCTCGTTTAGCCATCTCCATGATCTTTGACATC
CATGTTTTCAATGAAAAGGAAGCAATGCCATAAAACCTTGGTGCTATTTTCTGTTCTTTCTCC
TTTTCAACCAACCTCACAATGATATGTGATTTATTTATTAATATGTCTTTCCTGTCCTTGATTT
CTAATGATCTTGTATTATCCAGTCTTTCGAATTTAAACAAGTCAGATGGTTTATCCAGTTTTT
CCTTTTGAAGATAATAGATCAATTCCTTCACTGAGTTCACGCCATTATCGTGATAATTGTCAG
TGGTACATGCTTTATCAATAACCCTGCTTTCAAGATTCATCTCATTGCCCGGCTCTAAGATTT
TACCAAGTCTGACTGCCTTATAATAAGAGGCATCATGAGTGATTTGCTTGTTGTACTTGCCTC
CGCTGGACATGACATCTAGTTCATACTGTAAATCTGCTGGCACCATTGATAATTCTGGCATCT
TATTGTTCTTCTTTATATAACTGGTGACCATCTTCATTCGAAACATGGATCTTAGTCTTTCGAT

CACATTTTGATCGACAGGATGATCACGGTTGGTACGCTGTGTGTATTTCCGCAAGCCTTCCTC
GTGTGATACCTGCGCGTAATGTAATGTTTTTCCTATGCAAGACATTTTTACACATGTCGACTG
AGTCGAAGACATTATGATTTGATATATCTTTTCAGCGTGATAAGGGGAAGCTGAAACAGTAT
CATAGATATTATCGAGCACTGGCATCCGAGAACATGTGGATTTCAGATCACTCCCTAGGAGA
CAATTAGATTCATATAAAGAAGCAAGTGTTGCCCTTTTCTTTGTATTGCTAATCTTGTTCAAT
GACAGTAAGTAATCCACAAAAGGCTTCTCTTCTCTTGAAGCATGATCTCTTATAATGTTTAAA
GTGAAACCATGATCAGCCAAAGTCATAAGGTAATCTATACCACTACCCGGCATAAGATTTAT
GAAATTACCATCGGATATCGCCGATAGAGAACGTGACTTGAATATCTCTATCTTATGTCCTC
CCATGACAATCATTCTTGCGTGAATTTGTCCATGTGGGAACACCGTCACCTCAACTCTTTTAA
CTTCAGTGAATAGTCCCGTTGATTCTGTAGGCAAACCTTGATGTTTTTGCTTGCATCAATTT
TGGCTTTATAATTGATAGATTCGATGAAGTCCAATACCCAGGCTGCTTTCAGGGTTTGCTCTG
TCAGATCAACATTATTATCTTTGTCGATTGATGAATTTCGATAGTTTTGGGATAGCTTGTTGG
ATTCAAAATTCTCCAATCTGGATGACATTTTCCAAAGTGTTGCTGACTTGAGTGAAGGAAGG
GCAACTGTAAGTAATTGATTGTAAGTATCAATTACAGACCATTGACTCGAAATGCACTCTGT
GTTCCAGTGCTTAATGAGAGTGCGTAGGATCATTAAATCAGTCCGCTGTTTATTAGACCATTT
ATCAGGGTATTGTGTAGACTGCGAGAAAAATGTGAGATTATCTTCCGAAAATGTAACAAATG
GAAAATTAATTTTATCAATCTGCTCCGGAGGAACTTGATTAACTTTCGGCACGCGTAAGTCA
ATAAATTCAGTGAAGTGGTTGAAGGATTGTGGAAACTCAAATACTTCTTTGTCTGGTTCGAG
TCTTGCTTCACTTGATTGACTCTTGATCAGGAGATCATGTGAGCCTCCGAACTCATCGGCGAT
CATGTACAATGTGGGATGTAAGTCCATACTTGCTCCAAGTGAGTTCAAATGCTCAACACAGT
GCTTCTTACTCAACTCAGAGTCTTCCTCGTGTTCTTCTATGATAGGGGTTTGGTATTCATTGTC
GGAAACATCAGAATCGGAATACTGGAATTGCTCATCAAGGAAAAGTGTGTCTAAAGGAGGC
ATATGAAGTGGAGCTTCCATATCTTCTGATACATCTGCCCATGATACTGACTTTAGACTCTCC
ATGATTGTTAGTTCAAGTTTTATTTAAAGCGAAAACGATAATATTACTTCTTAACCATATAAA
CATTGCATCCAATCTTCAAATAGATTCGATTATCTTCTGACATTAAATCATCCAGAACTGCTG
TGAGAGAATTTGTGAGCACAAGATTTGTATTGTAATTGTTAAGGTAAATTTGTTTCCTGTGAA
CCTTGGTCACTAGAATAGCGTGATGGGTCATCATTCTATCTTTACCAACAGATATCATAGGT
ATATGTTTCAAGATCTTTGCGGGAGTTATCGATGCTGACATCACTTTTGAGCATGCTTGTAGA
TAATCCAGGCTCAGATGCAATTTGATCGGGGCATTAAGACATGGTTTTAGTATTGCCATTTTG
TCACGTTTAAGTTTTAGTTAAGATTAATTAAATTTAACTAAAAAATGTTCTGTTTATATCAGC
ACGACTGATATTCATGCCAATATAGC

>Seq61 [organism=Lepidozia ophiovirus_sela] RNA2 genomic sequence

TTGTAACAAACCCACGAAAACCCTTGAAGGCATGGTACCGAGGGCTAAAAAGCTTGTCGGC
CATTGATCGTATCACCGCGCCTTTTTTTATTAAAGCGATTACTTAATCAGGTCCACAGTGTAT
TAAAAATCATGAAAACAGTCCCATGCAGCATCCAAGCTTTCATAATTATCGGTATACTCGGA
CCAATCATACCATTCATCATCGCTTTCTTGATTCCATACCATCTCACCAGGAAACAACACTGC
ATATCTAGAAAGATTGTCCAACTTGAGAAAATCACACCTACATAAGCCAACCCCGTTGCAAG
ACGGTGTAACGTTACAATATGTGAAACAATCATGGTCAATACACATGATGTTTGCGCAGTCC
ATAATTGTCAACTGATCTTATACAAATTGCCCTTCGTGAGCTTAGCACAGATCTTCACCTTGT
TTTTGAGAGTGATCACCCATCCTTCTGAGTTAATCTCCATGCTATCAATATTGCCTGATCTGT

GCAATGATCCAAAAGACAGCAGATTATAAGTCATATTCTCAGATTCGAAGACATCACTTAAC
TGTGTCAACACACCTCCGATGCTCGCATAGTATTGCTTATGCATGTAACCTGTGATCTCTTCA
CCATTTGCTAAGGTGATTTTGTCATTACCTGAGATCTTCTTTGATTGTATCACATGGTGAGAA
GCCCCAGAATCAATATACCAGGTCGAGAAGTCATTTGGCTCTTCATTCTTGAGGGTAGTAGC
CATCATCACAATCACTGGTGGTGCCATCTGGGGCTCCTCATCATACTTGGAACCTAGGTACG
GAGAGTTTGTAATCATAGGAACCCCTCCTTTGTCTTTGATTCCTTCAAATTTATACCGTGATT
GTTGTTTCTGAATTTCGTTGTTTCTATCCCTGATTTGCTTCTTAGCGCGGTTTTCTCTCTTTTTC
AGTTCTTCCTCTGTCCCTGCTCTGACAGCTTGCAAGACATTCTTATAGTTGAGATCTTTGGGA
ATTTGACAGTCTTCTAAGTAATAACCAATCGGTTCTCTAGTCACTCTTGTGAATGCTTGAGAT
CCCGATTCGAGCTTAAAGAATAGAAATAACATGCCGTAATCTCGTCCTTGAACAGGAATATT
TGTTGCTTCAGCATTAATCTGGATGTTCTGTGCATCTTTTTGTTTGATGAAGTAGTTCATCGA
CAACTGGAATCGACACATGTATGATGCATTCAGTGAAACTGTTTGAAGAAGTTGCCTAGCTT
GAGGATATAGCGAGACATCAGTCAAGCTCAATTTTAGCTTGGGCTCATGTTTCTCAGCGAAG
TTTGCAGATTGCCTTGGGAACATTGGAAGATAAATACCATCAACCGCATGGAAGCTCACAAA
CTGGTTGTTACTCACCCCAGGGAGACTTTGCCTGACAACATTCCAGGCTTTCTGGAGGTTTCC
TATCTTCAAGGTGCGATTTTCTCTCAAGTCTGTAAGTTTCATGGACATTACGTGGCTGCTGGA
ATACACTGTATTCGCAGTAAGGTTTGCAAAATCTTGAACCATGTCGGTAATCCCTTGTTCCTC
GAGTTCAGGATCACTTAATGTCATGAACCCTCTAAACCCGGATTCCAGCAACTCTTCCGTAC
CCTTCTCAAATCCAACGTGCGAAGTGATTTCTTTTGTTCCGTTGTACTTGTTTCCTTTGCTGAT
TACATCGAAAACAGGCTTACGAGATGATTCAGTTTTTCCATACCCAGTTGATTCCATATTTGA
GAGATCCATGATCAATGAGTTCCCTTGATGTAGATGACGGCGTGTGAAGATGAAGCCCTGAT
GGTGCTGAGGTCCACAATGATTACTTGTTCACTTTAAGTTTGTATAAAAACTTAT
>Seq62 [organism=Lepidozia ophiovirus_sela] RNA3 genomic sequence
ATTGACTGAAGATTTTTCTTAAATCGAAACCGTGTAAAATTCACACAAACAATGGTCAACCG
TCAAAACACTTGTTCACAGTATCTTTCGTTAACCCCATGAAGTTGCCATTCTCATCATTGAGT
ATAGGCCAAGTTCTGTTATTGAGCGCATCATAAGAACCCATGATATTCTTGTCCTTCAGGAA
AGATCGCATGTCCTTGTCCTGGAAATGCTTTACCAACGCTAATCTCCCAGCCTCAGTCAAACT
GTGAAGAATTGCCGATGTCATCTTCATAGTAAAACCAACAGGAACACTCTTGTTCGGATTCA
AAGGGTGTAATTTCAATTGAGCATGAAAGTTTCCACTCCTGGAGCGGAGAGCGTCAACTAGT
TCGTGTGCCTTCACAATTTTCTGGTGAGCTGCCAATTCATCTGCTGATAACTTTGCATTTGCA
TCGATCTTCGATGATGATTGTGCAAAGAGAGATGCATGCTCTGCATACTTGACAACTCGATT
ACCTGCAACTGCGAGTTTCATTCGAGAAACATATTGAGGAGGGAGTCCATCTATATTCACAT
CCAGGAGGGAGCGGGCAGGAAATTTCCGAGTCGTAGTGCAAGAAAGATACTTTGCTACTTC
AGATTCAGTAGTCAATTCATCATGATATAAATTTTCACGAAGTAACTTGATGAGTGGTTTCTG
GTTTGACTCATCACTTGTATAGGTTGGCATACTTCCTCGGTTATAGATCAAAACAAGTGCAG
CAATGAAAGCCTTCCTTGCATGATCAACCGACACCGAGACCTCCTCCATGCTGATTTCCTCG
AGCTCTTCTATCGCAAACATATAGAGAGCAGCTTGATCTTCATGATAAGAAGAATACAGAAC
AGCATCCAAGACCAGTGTTCCTGCAGCAACCAGGTTGTGCACGGAAACTTTGGCTCCCTTCA
AAAGGCGTATTGAAACCTCACCCATATTGTATTTTTCATCCTCGGGAATGGTTTTGGCAAATT
CATTCATTTTCTCTACCAAGATTTTAATTTCCATCTTGAACGCCGATGAAGCAAGCATTGCTT

CAATATTCTCGGCGGTTACCATGGATCCAGAGCTTTGTCCCTTTTCGGGTTTACCCATTCTAG
TGATTATGGGTTTCACAGTTTTTTCGCGTTCTTTTTCAGGTGATGTTGTGCGTTGAGCAATAG
TTTCAGAAATCTTCTCTAACTTTGATTGAGCTTGTCCAGCCTTCATGACATCAACAGTTTCAA
CGCCAACATTCCGTACCAGTTTTCCTTTGGAATCTATCGTCACTGATATGTCACCAAAGTCCG
AGATCAACTTCTCGAGCTCCTTTTTCTTCTCTGGTTTGATGCTGTCATCATTTTTCAGCATGCC
AAACTGCTTCAGGATTTCCTTCTCACCATCACTCGCTTCTCCTTGGGACAATGACTTAAGAGT
CTTGGTGAGAATACCAGATTGGATCGAGCCAGACATTTTGAGAGCTGAAAGATTCACTCTTA
GCGATACACGCGAGCACAAGTTGATAACTTTAAGTTTG
>Seq63 [organism=Lithospermum ophiovirus] RNA2 partial sequence
TTTCATTATTTTAACATTTATTCTGATAATATTTATTCTTATAATATTGATTATTATAATATTG
ATTATTATAATATATTTACCTATATCACATTATACTTGATCCCAAGGCCCTGTGCTTCCTGAT
TTTCAATTTCTTTCTTAAATGCACTATTTTTCAACCACATTCTGCCTGGTTGAGATCCATCAAA
CTGCATGACCACAACATCATTCTTAAGCAAAAAAACATCATCTTCTTCTGTTAATATACTGTC
TACAACTCCATCTTCCTTCAATTTTGAATATGAAATCAAATGAGATCCAAGACGTTCATCATT
GTAGACCCATGCTTCTTTGAAAAAGAAATCAGATTCTTCAGTGACAAAATTTACCCTTGCTTT
TTCAGCATAAAATTTGTCTATGCCACCAAAGTTCTCCATCAAAGTTTCTTCTTTAGCATTCTT
AAAGAAAAGGTAATGTTCAGCTGATCCAGTGTCCAACATTGTTTTAAGGTCACCAGGTAAAA
TAGTGATTGTCCTCATACCCTTATTTGCAATCCTTCCCATCATTGCATTTCCAACCAATGAAA
TGCTCTTTCTCCCAACTTCAATCATCTCATTGTTATCCTCAGAACTAGAAGTCATACCATCTTT
GATAAAGTCTATCTTTTTCATTGTTCTTCTTGCCTCTGAATCATATTTCTTTTTCTTCTTCTTCA
ACTCCATCCTTTTGTTTTTCACCTCTTCCGAGATCTTATCCCCAAGTCCTTTAAAGACTGAATT
TTTATTTATGTCTCGTGGGTTTTTAAGAGAATCTATGTAAATTAAAGTTGATTGTTTTTTCTCT
AATCTCAATGGTGAATGTTCTTCATGGACAAAAAATGCAATGAAAATTGCACCGTATGCTCT
GCCTTCCACTGGCAGTTTATCTACAGACATTTCAAGACAGATTTTTTCAATATCTTTTTTAGC
GATAAAATGATTCATAGAAAGCTCCACAAGGGCCATTTCGTCTGCTTCAAATGTAACAGATT
GTATTATTGGATCATTTCCAGCAAGTTCCTTACCTTCATCAGTTAATGATACTGTCATTTTCCC
GTGATCAGATTCTTGTCTATCAAATAGAGGAGTGTACATTATTTGAATTTTTTCAAATCTCAC
AAATGGCCTGTCCTCTGTATTCCTTGATATTTTAGAAATAATATTCTTCAGCTTCTTCAAATTT
GTGATCTTTAGCCTTTTTCTATGATCTTCTTTCTTCAAGGTGAGAGTCATCGGACTAATCACA
GAATTGATTGTACCTTTCTGCACATTTGAGAAATCAGCTGCAATACATTCTATTCCTTCAGGT
AAATCACCAGTTTCGTCTTTAAGCGATATGACCCTGATTTGGTTCACATTATTCAGATCATCA
ACAATGTTTTGAGCAGACAATTTCCCAACTCTCGTAACATCAGGATTACGAATAGATGATGA
TTTGATAACTTCAGGAGTCTTGAAGGATGATGATGCAATCAGAG
>Seq64 [organism=Lithospermum ophiovirus] RNA3 genomic sequence
ATCGTCTTTTATTTAAAACAATAATAGTTGCAATACACACACCATAATTTGTATTTTTCAACA
ATTTATTTATTTATTCATTTATTTATTTATTTATCTAGCCATTAATAAAATGATCTTTACTATT
ATTATTGATCATTTTATCATCATTTTCACTATCATTCTACATATTGTTTAAACTCACACCACTC
CATAGATCCCTTTCAAAGTATTCACTGACATATCAGAAAAGTCTGCATCTGGATTGTCTAAA
ATCCTCCATACTCGAACTCCTGCAATATCTTCTCCTGCGAAATTGGAATCATTGAGGAAAGA
AGAATTTTTAGCACTTTCCATCTTGTTGAATAAAGATATCCTCCCTTCAACACTCAAGCTCTC

TATTATTGCTCGAGTGATTTTCAACAAAAACCCTTTCACCACAGGTTTATTTGGTGATAAGGG
ATGCATTCTCAATTGTGCTGTAGTGTTTCCACTTAATCCTCTCATGAAATCTGATATTGCTATT
GCCAGTTGAATCCTATTATTCATGGCTGTGAGTTTTGCAACTTCTGGACCTTTCATTTCTTCAC
CTATGATGAATAATTCCTCTTTCATGCATGTCCCTGCAAAAATTGCATACCTAATTCCTTTAT
TCCCTGCAATGCTCAACTTGGCCCTTGTTGCAACAGGGACTGGAAATCTGTTGATATCAACA
TTCAAGAAAACTCTTGCAGGAAATTTAGAAGTACTAGCAGATGATAGATTCTTTCCAATGGC
ATTTAGATTACCTATATCTCCCTTATAAATGTTCTCTTTGATGAATTTTGGGACTGGTTTCTTA
TCATCATCAAAGTTTTTCGGTAAAGATCCTTGAATATAGACCAGAACTGCAGCTGCTTGAAT
GGCTCTTTTTGCATCTTGAACATTTTTCCCAACTGTCATGGATGTCAGATTATCCACAGCCCT
TTCATTGAACATTCCTTGAGCTTCAGGATTATCTTTCATAGACACAAAAAGCAATGCATCTA
ATATCTTTGTTCCAGCACCTACCAATGATTGTATTTCATAAGTTTTTTGTTTATAATAAAAGA
TGCTTATCTCCTTTGTCAAGTATCCTTCATCTTCCGTAGTTACAGACTCCAAGAATTTAAGAA
GGTGTGCTTCAATAGATTTTGTGTCGATCTGATAAGAATCTTTCTTCACATTTTCCAAAAAAG
AATCAATTGATTGAATTCTTTCTCCTCCAACATTCATAGGAGCTTTTTCAGGATTTTTAGATA
CTATTCCAGAATCAACTTCTTCTTTTTGTTTAGAGCTTTCAGCGCTCTTTTTATCATCTGAACT
CTGTTTTCCTTCAGGAATGAACTTTGGAACTCCATTCATGATTTTGACTATTCCACCCTCCAT
CTGAGTCTCCTTGTTCATGATTCTTGCTCTATTGTCACTAGGTGCATGACCACTTCCGAAACA
TTCAAATTCTTTCAATATGTTTATCTTACTTGTTTCCATGTCTTTTTTGCTCTCATATTCTTTAC
AGAGTGTTGCGAGTTCTTTCAAGCTGATTGTCTTGGATTCAGAAGTCATTTTTAAAAATAAA
AATCTAAGATTGCTGATTGCCAATTTTCTCAAAATCAAATTCGATCGTTGCGTAAACTAAAA
ATATCCCCTTCAGGAATGAACTTTGGAACTCCATTCATGATTTTGACTATTCCAC
>Seq65 [organism=Lupinus ophiovirus] RNA3 genomic sequence
GTTATATTTAATATTTAAGATGGTGAGAAAATAAAGAACAAATCTATATTAATTTTCTTTTAT
GATTGCTTAGCCTTGACTGCTCTATATTTCCCCTGTGAAACAAGTTTGTGTAGACTGCAATTA
AATTCTTAACTGTGATTGGTTTACATTGTCACTATTTCAGGAATGAGACATGTTTGAGCTGAG
ATGACTCAATGGGAAGGACAGAAAATATGTGATCAAAACCTGTTGGAACATTTTTATAAAA
CAGGTATTTTTGATGCAGCCATAAGATGAACAACAAAACCAAATACAAAACAGAATAGATC
CCCTTATGATCTGGATTAATCACAAGACACTCATTCTGTCTTATACATCCCCTTAAGAACATC
AAGAGATATGTCAGAGAAATCTGCTTCATTATCCTCCAATACGGGCCAAACACGAACATTGT
CCTTCAGTTCTCCATACAAGTTAACATCTTTCTTGAAAGCATTATTGTTTTCCCGTTCTATCTT
TGCAGCTAACTCTTCTCTTCCTGAGCTTGTCAGTGATTCAATCACTGCTCTAGTCAGTTTTGCT
AACAGCTTTTTCATTTTTGGTCGATCTTTTGACAGAGGATGCATTCTTAGTTGTGCCTTAATA
TTACCTGCTAGTGATCTAAGGTAATCAGTGATTTCAATTGCTTGCTTCAGTCGTTGATTACTA
CTACACATTATGTCGTCACTCCCGATCGGTTTCTTTTCACTCTCTCTGGTACAAGAGGCTGCA
TACCAAGCATATCTTACTGCTTTATTACCAGCAATACTGAATTTGCACCTAGCAAACAGAGG
TGTGGGTAGTATTGCTATGTCAACTTTGAGGAAAAGATGTGCTGGAAATTTCCTAGTGCTAG
CAACAGAAAGGTCTTTTCCTATCTCAACAAGAGAATTAAGTTTTCCAGAATATAAATGGGAG
TTTAATAATTTGGGCACAGGTCTATCGTCCGAATCTGAATTAGGCGGCAACACTCCTTGTAT
GAAGACAAGGGCTAATGCAGCTTGTAAGGCCTTTTTACCTCTTGTTATGTTTTGAGCTACGAC
AAGTGGAGTTACTTCACTGATAGCTATGAGGTTAAAGATCCTCTGAGAGCTTCCATCTCCAT

GCAATATACAGAAAAGCAATGCGTCGAGCGCTTTCGTTCCAGCTGAGATTAACAGGTTCATA
TCTCGTGTAGCTTGGTTTCCACAGTATTTGATGGTCACTTCATTAGCTACATATGCTTCCTCTT
CCTGTTTTATTGAAAAAAGGAACTTTTCAAGATGATGTAGTATCTTCTCTGTATCAAGAGAGT
ACGACGTCTCTTTCAGCATTTCCTCAAATTGTTTCGAACTCCGTATTACTCGTTCTTCCCTTTC
CTCACTTGCTTGTCCTGAACCACCTGATTCTTCTTTTACCTTANNCTTCCACTCATCATCACAC
TTCCGAATTAAACTTAAACGAATCGAATCGAGCACGTTCGTCGCTAATGGAGATCGATTTCG
GAATCGAACCCTANNACCAGAGAGAGTTTTCGGTATGGATTCAGATCCTGTGATTCTCTCTC
TCTCTAGAATCTGGAAAATGGAGTTAGGATATGGGNGGAATTTCAAACAGTGATAAAAACT
ACGACTAAAAAACGTGCATTNGATTGTTTGAATAAATTTGACATGAAAAGGCACAAACATTT
GATTTCCAAAACAAGTCTGGATCTGAAAATCATGTATCATCAACTACTAGAATCGGAGTTCC
ACGCCTTCCATTATATGCAACCGCAGAACTACACCAAGTTATAACACCTTGTTAGGAAGCTA
CTGGAAAATATTACTAACAAAGAA

>Seq66 [organism=Osteospermum ophiovirus] RNA1 genomic sequence

TTCCGATCTGATTTCTTATCCCATGATCTACGATCATGTTCAGGCGCAATGCCATGTTTATTA
TTATTCTCCCTTCTAAACATTCCCAATTTCAACTTTTGTCGATTATGTTATTTGCTTCTATTTG
GCTGTTCAGATCAAATGTTATGCGATCAGCAGTAGTGTAGTTTTGTATCACTAGTCCGTTCCT
AGTTGTTGTCATTTTCTTTCCTCTATTGTGATCTTGTTTGTTACATTTGATTTTCTAGATCTTTC
CTTCCCATTATGATTATTTCGTGTGGTTCAACTACTTTATGATCATTAGCTAATTGTCCTACGG
TTATTAGTTGATTATGTTACTTAATTATTTTATTTGTATTTTACGTGATTTGATTTATTCTAACT
TCACAGATTGTTTAATGATATTTTGTTTCCTTGTTTTTTTCTTTCTTTTGCCTTCTTTTTGTTGT
CAGGCCATTTATCTTTTGATCTTTTATTCAAAATCTTCCCAGCAATCTTCCCCTAATTCTTTTC
TCATTTCATCGGTTGTCATGTTCTCATTCCTTTCTATTTCTGATTTCGTTGCACCAAGTAATAA
TGCAATTTCATTGTCAATTTTCCCAATTATTTCTGCTTTCTCCATTTCTTCAATTCCTAACACT
TCAGAGTCATCATGTGACTCTTGATTTTCTGGTTCCTGATGTATTCTCCTTTCGGTTAATTCTT
CTGTTATTGCGTTGAATAGCTCATTTCCAAACCTATCCGATAAGTATTCTTCCAATGTGTCAA
ATTCCCTGAGTGCTTTGATCTCATTTTCAATCCTTCTTTCTATTTCGTCTGATTCTATTCTTTTT
TCTTTGTTTCTTGACAACCTCAGCGTCTCAATGGACCTGTTTTTACTTTTTTTGTTTAATTTGA
GTGAGCCTAATTCGGATAATAGTTCCTTCACGTCTAGTGGCTTTGCAATCAATTCAAGAAAT
AATCTTTCATGCAATCCAATCATCCTGTTGAGCAATAATTTGAAATTTAGACAATCATACCTG
TGTGATCTTGTCTTGTTTTCCATTAAGCTTTTCATTGACCCTTTCCGTATTCCCGCAAACTGCA
AGAACACTCCTTCAACATTCCTTGACAGGTATGATCTTTTCACTATCTTGGCTATTTTTATCAT
CCTGTCAAATGAATGATATTTGTCCCTGAATGAAACTCCGGTTTCTATGCTTGAAAAGAGTC
CCTCTAAATCTGAATTCTCTCCTTCTTGTCTTTTAAGTCCTAAAAGAATCGTTTTCAATGATTG
ATAACTATCACGTCTGATTATTCTTTGATCTAATAAATGCTCTATTATGTATATTAAGTCTGTT
ACTCTTCCGTCTTTTAGTCCAACCATGTACAACCCTGAAGTGTATTGGAAGCTCGAGTTCACT
TCAATCTCAGGTGATTTGTTATTGTTTTTGTAAAACTCTTCTTTAATTTTCTTGAAGATAGATT
TATTAGGTATATTTTTCTCATCTTTTATAATAAACACGGGCCAGGATAATTCCATTAATTCCA
AATGTTTTTCTTTTAATTCCCTCTGCTCCGTTGCACCATTACCATTCACACTTCTCTTCTTGTCT
ATGTAAAACTCTTCCACTTCAGTGATGATTAGTTCTTTGATTTTGTTTCCTTTCATTAATTTCT
TCTCCATTTCGGCTTCTAAGAATTTGTCATCGTTGTTATCCTCCAACAATATGTTAAGTAATG

TTTCATCTGATATTATTTCCATAGTGTGATCCATCACATCCGTTGAGCTCTTGTTGAACAGAG
AATCCTTTCTCATCTTGTATATTTCATTTGTTAACTTTTCAGCTATGATTGATTCAGTAAACGA
AGTTTTCTCTTCTCTTATTTTTTCATTTTTATTTTTTCTAGGTTTTTTCCTACCATCTGATTTTTC
TATCATAACATACATGTACCCTGGTGATTCCACTGAGGGCAGGAGCAATTTTCCTGTTCCGCT
ATTGCACAGGTTCTGAAGGAAGGATAAATCCTGATTTATAATCGAATTGATTCTAATTACTA
TCATCTTATCATTGTCAAACAATTCTTCTATCATGTTTCTCAATGTTGTTTGGCTCCCTGTGAA
ATGAGACAGATCCATCAAAAAGACATCATGATCTAAGTATTGACCATAATTTTCATTTAGTG
TCATATCCACTCCTCCTTTGATCTCTATCATTCCTGGCACTCTGTTTATGAGGTTATAGCCATC
GTTTCTTGATATTGATGTGTGACACAAACTCAATTGAGTCATGGCAAAGTGGAAGTCTCCTC
TTCCTCCGAACACATCCATTATTTTTAGCTTACTTATTTCTCTTTCAAGAAAGTCAAAAGTC
CCATTGCTGACGGATACACATCTGACATCGTTGGTGATGATACGTGTTGTGGTTTACAGTGG
AGAGTGCATATCATTTCATAACTTTTTATTGCTGCGCTTGTCATCTTGCTGTCTTTGAACATTT
TGTCGTCTATATCTATTTCTTCGTAGCGCACTGCACCGAAATCTTTGTTGTCAAATTCATTCGT
TATGTAATGTTTAATGTCCTTATCATAATATTGAACTAACATTTCTGTTTCAAAAACTCTATT
ATTGAATCTTTTATATATTTCGTCTATGCAATCAAATAGTTCACTCATTCTGTATCCACTTGTA
GGAAAAGCCATCATGATCAAGTCTGATACTCTCTTATCCCTAATATGGAAATGTGCTTGATT
CTTTTTTAACCTAGTTAAATTATTTACTCCATTCTGTTTTGTGTTATCTCTTTTGATTATCATTT
TCATGCACCTTGGATTTATTTCTTCTAGTCTAAACACCAAGCATGTAGATATTATGAATATCT
TCATCAGCGTTTTTCTGTAATTCACTGAGTAACCATACTCTTCAATTTCCTTTATCCAATGATC
ATTGTCTTCCTTATTGCCTATTAGTTCGGAATTGATTATACTTCTCATTACTGTTTTTGTGTAA
CTTTCATTCCCTATGATGTTTAATCCCTTGTAGAATTTGTAGAACTTATTTAAGTAATTGTCTC
TATTTATGCTCATCTTAACTTCAGGGGCAATTTTCAACAACACGTCGTCTGGTATCATTTCGG
GTGATATAATCTCTTGATCTTCCATGTATTTGTGGAGACTTCTGAATGATGAGATCTCATCTA
TTTTCTCATTTGGCATGACAGACTTGTGGTCAACAAATTTTCCTTTGATTTCTTGATCACATG
ATATAAAGTATGAGTAATTTTTGTACAACCTACCTTTTTGTTTTATCCTCTCCGTACTTAATTC
AGTTGGTATTTTTCCCTTTAATTGAATTGGGTTGATCTCATCCTTTATCTTTATTTCTTCTAAG
TTGAATGTCACATCTTTTGCATGTCTGACTATGTCGTCGTATAACATCAATGGTCCCATCTCA
TTATCTGTTGCATCAAAGTATCCCATCATCACTTTTATGTACACCACTACTAATGACAAATTG
ATGTTATTGTCGACTCCCCCTGTATATTCTAATAGATTGTTCAATCCAGTTATTTCATACCTGT
TTGTTTTGATGTTTGATGTTAAGTCTCCTGTTTTAGGATTGAACCCACCTGACAGTGCTCTGT
GAAAATATCTGCCTCCTTTCGGGCATACCACACTCTCAATCACATCACTAATTTTACAATCAG
AGTATGTGTTTATAGTAACTTCTGTTATTTTCATTAAAGATTCCTTGACATGATCAGACACCT
TTCCAAATTTTTCCATGTCCATTATCATCCATTTGACATACCGTACTAACTCATATATCAAGA
AGATGAGCTTATTCGAAAACATTCCTTCTATTTCTATGTCTCTGTCAAATTTTGTCAGGTTCA
TGTGTGTTTTTGCTGGCCCTGCAAGTCTGAATTTGTCTCGATTTTTCCCTGTTCTGACTACACC
TGCTTTCCGTATCGGGTTGATTAACCCTAACCTATCATTAATTTCTAAAGTTCCTATAATAGG
TAATTCTTCAATTTTTAAAAACTCCATTTCAATCGCTTCCCATTCTCCATTATCATTCAATATT
TTCTTTTCTGCTGTGTCTCTTTGATCTACTATCCAGTTAAAGCCGAAGTTGCAACCTCTTATAT
CATTTGATTTGTAATTAATCATGTGATTTGCATTCCATGCTTTGTTCCATAGCTTGTTCATTTT
TCTTTTCCCTAAAAAGAAGGATAATGTTGTAGAATTGTCGATTTTTGAATAGATGTCTTCAAC

ATAAGCAAATATTGAGCATTCAAGAAATTTGCTAACGATTCTATGAGAGAAACAAAATTGG
AATGTTGACAAAAGTTCATGTTTAAATGTTCTTTCATCTTCTTTTGTCCCTAGCTCTAACAACT
CTTTTAGTTGTTTGTTTGTCACCTTCTTTGACAGGATTTTCTTCAATTCATCTTTCAATAATGA
TTTTGTATCATGTCTTCCTCCTTCTAGCGGAAAAGGTTCTGTAATGTAACACGTTGCTTTGGA
AGAAAAGTTTATTAGACTAAATGTCTTCTTGTTGAATTTGTGAGACAAAAGGCTGTTGACAT
ATTCTATCCGTTTGACATTAGAATGTGAGAATCCTGAGATCATAGCATCTGGTAAGCTTGTC
ATTGCATAACCATACATAGTGTATGGCAAATACATCATCATGATTGTTGCTTCAATTAATTCA
TCATTTTCCATGTTGAAATGGTAAAATGAAGTCAACATATCTGATTCTAGAGAGTTCCTGTTT
GAATCTAGTTGATCTCTTAATTCTTCTGCTTTTCCTTGTTGTTCTTTGTTTTGTTTTATTTTTCC
AAGGCATAGCTCAGCATATTTTCTCATTGCTGAAATATTGCTAATGAATGATGTATTTAAATC
TACATCTAAGAATTGACTCCATCTTAAAACTTCTTTTTCAATCACCGTCATTGCTCTGTAGTT
CCTTATCACAATCTGTTTTCTAAGATTGTCGGTCCTTAACGTCGATGATGTGAATCCGCTGTC
TATCGTCATCATTCTTTCCAAATCATCACCCCATAAAACTTCACTGTTGGGGCTTATAGCTAG
TACTCTTTTGTAAGACATGTCTATAGGTCTATCTTTGAAGTAATGATTTTTCAACATTGAGCA
TCTGCCGTTTGTTACTTGGGTTTGAGACAGTTTCACTAAGAGTCCCATTTTCAGAGCGTACGC
TTGAACGAATTTTATAATATTGTTCAAACTTTTTCATCTAATTCTTTTTAACAAAAACTCCA
CATGAATCGTCACTGTAAGTGGTCCCTATGAATTTTGAGAAGTTTAGTGATCTCATCATGTCT
TCCATCATTAGTTGCGACTGAATTCCCCATAAAGGGCCAAACCAACCTTCTATTGCTCCTTTC
TGATATCTTGATATCATTACATTATCTGAGAACAACTCTTCATACAGAATCACTATATCTGAG
AATAAATAAACTATCATTGTCATGCGTTCCCTTTCTTTTGAACCTTCTTCAAAACCATACATA
TCAGATATTTCTTCCATTATAAACAAATTATTTTCTGGTCTTTGTGATGTGTTGTGCCCAGAA
TAATCAAGAAACAATGAATAAGAGTCATCTTCATCTAGTAATTTCGACATTTGATGCATCAA
TTGTCTTCTTTCATCCTCTGTCATTGTCATCATTTGACCTGGTATCAATTTCATTGCTCTTTTTA
TCATTTCCATCAGCACGGAGATGTATATTTTCAATTTAAAACTTGCCACTCCGTAGAATCGAG
CTTTTGTTTTTAATTCCTTTTCCTTTTCGACGAGTCTAACCATTTTGTATTTTTCGATTGATCCA
ATGTTTTCTTCATCTTTTCTATCAAATACCCCACATTCTCTTTCGAATTCCTTTTTAAACTTGTT
GATAGATAGGCTGTCTATTAAGTCGGGGATATCATTTTTCGTGATATAATATATCAATTCTTT
GACAGAATTTCCTGAAAAGTCATATTCATCTTTAGTGCATGCTTTATCTATTACTCTCGATGC
CACGTTTACTTCTTGGCCTGGTTGTAACATTTGTCCTAGCCTTATATTTTTGTAATTAACTTGG
TTTGATACAATATCAGAGTTATAACTTCCTCCAGAAGCCATAAGATTTAATTCTTGCTCTAAT
GTTCCTGACGCTCCTCTTAATGGGGGGACTATTCCATATTTTTTCACATAATTCGTCACCACT
TTCATTCTGAAAAGCCATCTCAAATTTTCTACAGCTTCTTCTGAAACACTGTGTTTCCGGTTG
GTCCTTTGTACATATTTTTCCAATCCTCCGTTTGTGTCAACTTCAGCGAAAGTGTTGATTTTTC
CCAGTCCTGACATTTTAATGCATGTCTCTCCATCTGATTCTCTGCATATGTTTACCAATTTTTC
TGTCATCTCTCTGTCTATCTTTAATGATTCAATTATTGCATCCATCACCGGCAATGATGAAGA
TTGGACTTTGGTGTCTGCCATCATCAAGCATAAAGTTTCATATGACGCAGCTAATTCTGTTCT
TTTAGGATTATCTACCAGTGATAGTGATTTAACATAATCTATGAATTTGTTTTCTTCAGTTTTT
GCTTCGCATTTCCTGTCAAAATTCATATCTCTAACAATGTTTATTGTGCATTCATTCTCAATTA
CAGTAAAGAGATATTTGATCAGTGTGTTGTCACCAATGTAGTAAAAGGGGCCATAATTAAGA
CAATGGAAAGTGTGGCTTATGAAAAGTTTAAACTTAAAAATATCATTCAAGTCATCGTTATT

TTTCCTTATTTTTATGCTGGTGACACTTGAGCCATCAGAATTGATCATGCATTTGATTTTTCTG
CCCTCATGCCATAGATCCTTCGATTTGTATACCCTTTTATTGCTGAGACTAGAATAACTCAGT
TTAATTTTATGATTCACGACTTCAAATAAGTTTAACATCCACATTGATTGTAAAGTCTCCGTG
CTTAATTCTGTTCCTTTGATCGGTTTCTTCTTTTTGTTACACTTCAACAGATTGATTCTATTTTT
ATGTTCTTCAAATTCTAGCACTTTTTCTCCAAACCTGAACAATGTCTCCATTCTTATTGAAGG
TATTGAATAATCCTTAAGAATTCGATAAAATTCAAGCATTTTATCCTGATTGCCTTGTTCTAT
GATTTTCTTTTCACCAAGGAATGACCGCAATCTCTTGAGATCAATCCTCGATCGCATTCCAAA
TTTCTCCGGATACATTCCTGTAGTCTCAACCAATTTTCTGATATTGTTTTCATTAATTGGCTTG
AACGGTGAATCTATTCTTGAGATCAGCGCAGGCCGATCAATATATAAGTCAGCCAGATATTC
CTTTGGCCATTGCTCATAATGATTGATCAAACCCATGTACCCTAGTTTGATCTTTTTTGGTTCT
AGAGATTCTTCTTTCTCTCGAGACATTTCTAATGAGACGTAATTATCTCTTCCTGATTTAATC
ACTTCTAATACGTTTACACAATTTTCTGTCTCCAGTGTTTTTATCAGGTCCTCAAACTCTTTTT
GCTTTTCTATTGTTGTTGTTAAGTTTTCTTCTTCTGAAGAGTCACCTTCAGTTTGATCAATACT
ATATGTCACAACATTGCTCGACAGCGTGATTCCATCTTCGAATTTTTCTTCAACTTCAATGCT
TTTCTCTTGAGTCCACTCCATATTTCTTCTCTGCCTCTATTTCTTCCTAATTTCAACAAGAAGG
TTGTTCTGGTCGTTGACGTTTCAATTGAGTTTTATTAAAACGAATAGAATTGACGGATCATAG
TAGTTGGTAGTTTAAACCCTTGTTATTGTGATTTTAAGCTATTCTGTAAATCTCCTCTTATAAT
CGCAAAGAGTCCTGTACTTAAGCTTAACCGGTTTTCTTTCAACCAGATTATCTCTTATCCACC
TCTTGATGGCATCATATTTCTCTTCAACATATGTCGCATTTTGTCTTTGCATCGTTTTTTCCGA
TTGTTCCACTTCTTTCTTTAAATTTGTCAAGGTTTCCTCATGACAAACTCATCTTTTCTGTCG
ATTAAGTTGAACTCTTTTCTTATCATAAACTTCAATTGATCATTAGATGAATTTTTAAGGGTA
TCAGCTAGCTTTCCTGAAATGGTTATATCTTGCCTGATTGTATTGATCATTATTTGATCAACC
ACTGTCAAATAGTCATAATCCTCCCATAAGACATTTGTAGTGTTTATCATCATTTTGTCAGCT
CTGATTTGCATTCCTTCAAATTCATGTCCTGGTTTTGATTGGGTTCTGTATTTTTCCATTCTCT
CAAAAGCATTCTTTCCAATAATCTCATCATTGCACGTTAACGCCCATATTCCTATAGCCTCCT
TGCAAACTATTATATCATCTTTTAGGGTTGTCTTGTTTTCTATGTCTATTTTTCTTGTTATTATA
GAACACATCTCTTCCATTTTCAATTTAAATTCATTGTCTTGAATCTTTTTAATTTGAATTATTC
TATCCATGCAATTCGGGAC
>Seq67 [organism=Osteospermum ophiovirus] RNA2 genomic sequence
ATTGATCACCAATATTTTATTAAAACGAGTAAACGCACACCAGTAATCTCGTTACAGTTTAA
ATCCACATTAGTTTAAATATGTTTCAATGATTTGGATTTTTAATCATTTTGCTGACGCGATCA
CTTCCATCAAGTACACATTTGCACTTTCCTGACCACCGTTTGTTACTTCATTCCATTTGTCATC
TTTCAACCACATCCTCCCAGGGTCACAAGTGTCAAATATCATGACTATTTCATCATCTTTTTT
CAAGAAAAGAGAATCTCCTGCTGTTTCCATTGCATCAACCAGCTTGTCTTCTACCAGCTTGG
AATATGATAAAAGATTGCTTCCCATCCTTAGATCATTGAATCCGTAAGCAAAACTTATATTG
AACCAATTTCCAAAAGCTTTAAGTTCAACAGACATCTCTTCCACTGAAGCCTTATCTACACC
ACCAAAATGATCCACTGTTGTATTTGGCCTGAGTGTAGGGACATACAAATAATGATCTGCTG
ATCCAGTATCAAGCATTGTCAGCAGACCATTTCCTCCTTCAATCCTCACTGTTCTTCTAGGTT
GCTTCAATGGATTAGGTGCAGATCTTTGATCCTCAAGATACTTGGACAAAACAGATACACTT
TTCCGTCCTTCTTCAATCAAATCACTTGGATCAACTGATGACCCAGATGAAGTAGATGATCTC

TCCATCTTGAAATTCCTTTTACTTTTCCTCATTTCTTTTGACCTTTCCAATTCTCTTCGTTTGAA
TTCCAATCTTTTTTCATCTATATTTTTTGTTACATTATCTCCAATCTTCTTGAAGACTGATGAT
CTAGTTATGTCCTTTGGCCTATTCACTGAGTCGATGTATAAAACAGTAGATGGTCTTGTTTTC
GATTTTATCGGGATAGATTCCTCAAATGTGAAAAAAGCAACATTGAGAGCCCCATATGATCT
TCCTTCAATTGGCACTCCTTTCGCAGTTATGTTAATGCTGATTTTGTCCATCTCCTCTTTTCTT
ACAAAAAAATCCCTGGACAATTCAACAATTGCCATTTCAGATGCATCAAAGATCAACCTCTT
AATTATTGGGTCCTTTCCTGCGTCCTCTTTTCCAGAATCTTGAAGTGATATGCTTATTTCCCCA
TTTTCTCCCTCTTCCTGTGAAAACAAAGGAATGTACATGAATTGTATTTTCTCGAACTTTATG
AACGGTTTTCCTTCCTTACCACCTAGTTTTTTCATGGCCATCACTATTTTGTCCATGGTTGCCA
TCTTAAGCTTCTTGTTATGCTCAGCCTCTTTGAGTTTGAGAGTCAAGGGCACTACAGCAGTCT
CAACAAGTCCCTTGTGTATATTGGCGAAGTCCATTGTGATTTCTTCAATTCCTTCAGGGAGGT
CCGAGTTTCCATCTTTTAAGCTTATCACATTTAAGGATCCCTTTTTTATGAAGTTATTACCCAC
TGAAGTAGCTGAGATTCTGGCTGGCTTGGATGTCATACCGTCAAATCTGTTGACTGCGAGCA
CACCTGCGGCTGATTTGTAGCTAGAGCTTGATTTTACAGACATTTTCGCTGATATCGATTGAT
CTTTTGAACTCTAGGTACTAAAAAAAGTATCGGTACATACGTAGTGTGTACTTTGGTTAACT
GGACAATCTGTCTGATTCCAGAAGGCGCAATGCCATGGGTGGAATTCTCGTCAGCGGATCAT
ACT
>Seq68 [organism=Osteospermum ophiovirus] RNA3 genomic sequence
GATGGAAGTGTCAGTGTCCTGAACCAATCGGACCACGTTTATTTTCATCATTGTGGAAAGTG
CTTTTAGGATCCTGGACATCTCACGCAATTTTATATATGTTCTTAATTCCTTCAACACTTATAC
CGCTGAAATCTGCTTCTGCGTTTTTGACTATTGGCCACTCTCTGTCTTTTCCTTCACCGTATCC
GAAGAAGTTGACATCTCTTTTGAACGCTTCATTTTTGTCAGCAATTATTTTTCTGCCATTGCT
TTTCTTCCACTTTTGCTCAGAGAGAACACTATCGCACAGGTCAGCTTTAAAGTGAAGTTCTTG
ATAGTCGGTTTAGAATCTGACAAAGGATGCATTACCAGCTGATTTTTGTAACAACCATCCAA
GGAAGCCAATGCATTTTTAATCCCTTTCGCGAGGGATATTTTCCTATTATTTTCCATTATTAA
TGGAATAGCTGACCTTGGCGTTTCATCATCAATTTCTACTTCGTTTACTTCTTCAAAACCACT
TGCAAACACAGCATACCGTATTGCCCTATTTCCTGCTACATTCAGCTTGCATCTTGAGGCTAC
GGGTGTTGGCAGCAGATCAATGTTCATCTGCAATAACACTTTTGCAGGAAACTTTGCAGTGC
TTGTGCAACTGAGCTCTTTCCCAATTTGTTCCATGCTTGCAGATTCTCCTTCATACAATTTATC
GATTATGAAGTTTGGCACTTTCCTTCCTTCTGCAGTCTTACTTCCTGGAAGACTTCCTTGTAC
ATACACCAAAACGATTCCTGACTGTAATGCTCTTTTTGCTTCCCTAACATTGATCACAATTTC
CTTTTCAGATATACCTTCTTTTATTTCTTCTTCAATGAATAATCCTTGATTGACTGGATCATCT
CTCATGCTGCAAAACACCAGAGCGTCCAATATCTTTGTTCCAGCTGCAACCAACATTGTGAC
ATCATCATTTCTATTATTGAAGTATTTCACTAGCAGATCCCCCTGTCTGTAAGACTCATCTTC
TAGTGTGATCGTTTTAACAAAAGCTTCCAATATTTCTTTTAACTTAGATATTTCGAGGACATA
ACTTTGGGACTTGATGGTTGATTCAATCTCATTTTTCTTGGTTATCTTCTTATCAGACAATCTG
CTTGTGTTAATCCTTTTCCTTGGTTCTTTTTCTTCACTCTCTTTCTTTTTTCCATCTCCACTGCT
GCTCCCTGACATCTTCATTATCTTTCCCTCTACTAGAGTGACCATCTGTTCTTTGTTCTCTTTC
ACCCAATTAGATACCAATGTGCAGTTAGAGGACATATAACTTCCATTCTGCTTTAATAGCCC
ATATTTCAACAAAAGTTCTTTGATCTCATCTCTAGCATTCTCCTTATCGCTGACATGCTCCTC

AAAGGCTGCATGGAGTTCGGAAACTTTGATTTTGGATTCCATGGTGTCTTAATGCTTGAAAA
ACTTGCAAAATGGTGCTTG
>Seq69 [organism=Phalaenopsis ophiovirus] RNA2 genomic sequence
ACCATTTTCACAAATAAGAAAGACACACATAACACATCATCACATCACCATTTTAATATATA
ATTATAAATTGATTATCATCCAAGGACTACTTGGGAAGTATCGTCATTATTATCAAAAACAA
GATAACCTCCCTTCTTAGAAAGTGTATAAATAACCCTCTCATCCTTTAATAATATAGATTTTT
CATTATCTAATGATCTAAGACAGTCAACAATTTTATCTTTGACCAACTGATTAAAAGATATC
AAGGGTTGGTGTCTGGCTGAACAATAGAGAACATCATTCAATTTTGTCCAGTGCCTACCTAA
TTTGACTTCAACTGGCCATGGCAATATAACTTGCCGTACACTCTTAAACATTGAACTTTCACT
TTCTGAAACGTGGGATCCTCCTATGAAATTTGGGTTATAAAGATGATGTTGAGGAGCACCAC
TGTCCAAAATATGATTATCCTTAAGCATGTATGGTAAGTCACGAGGAAGATGATTATGAATT
TCTTTTTCAGTAATAGTTTTAGGAGGAGTAAAATTTTCTATTGTTGATTCACTATCCTCAGAC
TTCTTCCTAAAGTGTTTTCGTACTTTAGCTTCATTATATTTAATTTCAACATCATCATTAGGTG
AAGCATCTCTTTCTTCCTCAAGCTTCTCTATATCTTCTTCAAGTAACTGTTCAATATCATTTTT
ATTTGGTGCATCAATATAGATGTCATTGTTCCTGAGCTCTTTCATCCTAAAGTAGTCATGCTT
TCTCTGTTCGAACCTCTCTTTCTTGTTCCGTATATCTGTGTTAACTTTGTCAACCAGCTTTTTG
ATACTGCTTGTCTTGTTAATATCAATAGGCGCGTCTATTTCATTTAGTAAAAGTGTCAAGGGT
GCTCTTATCTTCATACTAGCAGGAGCAAAATCCCTATGAATATAGAAAGCAACCATAACAAA
AGCATATGATCTACCTCTGACAGGTATTTCTTCAGCAGTGTAATTGAGAGTAATGTTCTCTAT
CTCTTTCATCTTAGTAAAAAAACTCATAGACATCTCACTCATTGACATGACGTTCAAGGGAA
GATCAACTCTTGATATCACTTGTTTGTTATTTTCGATAACTGAATTGTCTTTTAATGAGAATCT
AATGATTTTTCCCTCAGCTATTCTTCGATCAAACAACGGCATGTAAAGGATTTTTACTTTATC
TATGCGACAAAATGGATATTCTTTTCCTCCAATGTTTTTGAGAGAAGATGTTATCTTTTGGAG
AGTTGTTAATTCAACTTTTTGAGAACCCTGACCATCCTTTAATTTTAAAGAGAAAGGAGAAA
CTATTGACTCTATTTCTCCGCGGGTTACATTTGCGAATTCTGCTGCCATGTCCTCCACATCAC
CATTGATATCACCCTCTCCTCCCAAGGAAACAATAGTATGTGCACCATCATTCAATAAATGG
TCATTTGCTCTTCTAAAAGAAGCCATCTTGTAAGAAGTCAACGATCTATTATTACTGGACGAT
TCATCATTGTAATATTCTAATGTGTTGCAGTGTCTCTCCATTGTTATTTCACGTGGTTCTTTTG
TGCTCTGATCCGAACTGCACCAAGCAACAAAAGCACAGCGTCTCCGAATCACTCTAATCCAA
ATGACCAAGTGAGACAGATTACTGTATCACAGACTGGTTGTTGACACTTAAGCAATTCAACG
GCAAGCAGATGCAC
>Seq70 [organism=Phalaenopsis ophiovirus] RNA3 genomic sequence
GCGTGTTACAACCTTATCACCAAGTTAAAATTTTCATTTATTAACCTGTCATCACATCAGCAA
ATCACTTGCCATATGCAGCCTTGATATTAGTGATGGACAGGCTAAGGAAATCTGCATCGGTA
TTATCCAGGATGGGAAATATTAGCTCATTTTCATTGTTCATGACTCCAAAGAAATTTGTATCT
CTTTTGAAGGCTTCAATCTTCTTATCTATTATGGTGTCATGAAGGTCCTGTCTACCCTTAGCA
GTGAGAGAATAAATGATTGCATGTGTAAGTTGCAGTGTAAAATTTTTCAATACTACTTTTTG
AGAGTTCAAAGGATGCATCCGTTTTTGTGCTTCCCAGTCAGATGACAATGAACTCAAACAGT
CAACTATATCGCATGCGGAAGATATTTTTCTATTAATTGCTCTGCCCACAGACCCTCCGATAT
CTTCAGCATCCGGGTCTCTTCTTTCGAAAGACTTTGCAAGTACTGCATATCTAATTGCTCTAT

TCCCCGCAATAGACATTTTGCACCTGGAAGCGACTTCAGTTGGTAAACTATCAATGGATATT
TGTAAGAAAACGGAAGCAGGGAACTTCGAAGGGTTTGTAGAAGAAAGTCGTTCGCAGAACA
TCAGTTCAGTAAGATTGTTGTCACCAAATATTGTCTCTTTAACAAATCGAGACATTCCCCTAT
TCAACTGTCCTAATGAGGGCAAATAGCCTTGATTATAAACAGTCGTGAAAGCAGCTTTAATT
GCTTTCTGCCCATAGTTTATATTTTCAGCTATCTCCAATTTATTCACCTCATGATCCATCTTTT
CTACATCGAAGATGAAGTTATGTTCATCTGATTCATGATAGCAGCAGTAAAGTATGCTATCA
AGAACCTTGCTCCCTGCAGCAAGAAGACTTGATACACTTATATCAATGTCTTTGAACATGTA
AATTTTCACTTCTCCTGGTAAATAGGTTTCACCTGATTTCGGCAATGTCTTGATATATTCCTC
AAGTACCTTTTTGATATTTTCCAAATTTAGGTTCAACGATTTTCCTTCTATTAGTCCTTGTAAT
TCAGATTTTTCAACAAGTGTTTTGTTGTATACTGTCCGTGCTGTGATAGTTGGAACCTGAGAT
GAACTTCCCACCTGAGTAGTTGTACCTAACACGATTTTACCTTCCGCTGACAAGAATACAAT
GTCATTTGGGTTTGTTATGTTCAACTCTCTGAGTTTGATGACATTTTCCTTGTTAGGCAAGTTA
CTGCTTGTGTATAGACCAACGTCTTTCAGAATAGTCAACTCATTCGCCGACTTCTTCTCTTTG
TTACACAGAGACAGCAAATGTGATGCAGAATATGCTCCTGACATTATGAACAGTAGTACTTC
TTGACAAACCAGCAAGTGAACAAGCAAAAAAACTGAAACTTAAACAGGCTACAGAGAAACT
AGACAAGAGATTGCAAAGTGAGCAAGTAGTTTGCAGAGAGATA
>Seq71 [organism=Phalaenopsis ophiovirus] RNA4 genomic sequence
ACTCGCCAGGTTACTTGTCCTAAGTTAAACTACTCCTACTCCTACTGACTAACGGGTCGCATT
GACCGTAGTTTGGAGCATGGTTAGTGCTCAAGGGTGTTTTTTTATCATTTATCCAAGTTTTAT
TTAAAACCCTATTTCTCTGTAAAATACAAATAACCATCTATTTTATCTAGCTCAAACATTATC
TTTCTATCAAGTATAAGATAACACTTACTGTCAGAAACATATTTCATACCATTGATAAAACC
ATCTTCTACTAACTGAATATATGATATTATAGGTTCTGAATTGCAATCACCTATGCAAACATC
TTTAAGAGGAACCCATTTATTATTCAATTTAACACTTATTCCTTTTTCAATAATTATATATTTC
CTCTTCTTGTCATTCCTTGTATCCATCATTGGCATTCTTTTATTTGTTTCAAGGTTATTGTAAT
ACAACCAGTGATTCGGTGCTCCTGTGTCAAGGATATACCCTTTACCCTTTATGTGTGGGATAT
CTCTGGGCAATAAGTTTTTATAGTTTTTTTCACTTAATTTGAAGGGAATTTCATTTTTCCCCAT
GTTGATATCATTTTTTGAATTTTTTATCCCGAGTACAGAACTACCTTTTGATCTATTGTTCTTT
TCATGTATTTTATTTTTCATTAACTTAGGACAATTTGTGCAATATGATTTTATTGATTTCCCTG
ATGACCTGGAACTTGTTTCCTCCATATTAGATTCTTTAGTTTTAGATGATAATTTTGAACTAG
AGCCGATGGATTTTGAGTTCCCATTGATATCATAAATCTTCCTTGTTTTGTCAGATATGTTTG
ATTTACCTTTAAAATCTCTGCTGAGTAATGTTATTGTTCCATCCTTTATTGAACTGTATTGAG
AAGGAGAAGGATGATTGTTCAACCATGATTTTACATTATTGTCTGTGGAATTCATTTTGTTAC
TTTCAAATGATATTTTTGTACCATGCAGGTATGAGTTATTATGAGAACTTCTACCTTTTGTGG
GACATGATGAGCAATGACTTTTCCTCATACTGTTTTTACTCAAACATGATGAACAATGTGAT
ACAACCTTACCTGTGACACTTCGATCTGCCCATGTAACTTTCTTGGGTACAGATAAACTTGAT
GGATAGGGACAGCCTCCTGATGGGCAGTTTTGACCTTGGTAAGCATAACTACCATGAATTGG
GCATGTCGGGTCAACCATCCAGTTGTTCCCATTAGGATCTACACCAGAGATCGGAA
>Seq72 [organism=Primula ophiovirus] RNA2 genomic sequence
TTGTTTTTGGTCTGAAACTGACTACCGGTTTTGAAATAGGTTTTGTCCAACAGTGGCGGAGA
GATGAACTAAATGGAGTGATTGTTGACATGTTTGTTTGAGCACAATTATTACCTTAGACAAC

GGATCTCTTAGGAACCTTATTCAACACTCAAAAGCAACACACAATTTGATCTCTCACTTCCTA
GATCCCAGATCCAGAAAGCACAATTGCCCGTTTTCAACAACTTCCAACTCCCACACCAGTTT
ACTTTCCTTGTACAAATACCCTGAATCACCAGTTATTGTCAACCTGTCCACCAAATCATCCTC
TTTGAGCCTGTCAAAAGATATGACATTTCTTCCTATATTGTCCTTCACCAGAAACACACTCGA
CAACACAACCACTTTGCCCCCTATTATCATTTCAAAGTTTGATTCAACCTTATAGCTGATCTT
CTCAAACCCTATCACATTATGCATGTCAACATTGGGGATTACATGTGGAGAATAAATGTAAT
GGTCCGGAGCTCCAGTGTCCAATATCATATTAGCAGCTCTGGAAGCCGTTCTAGGACCAATA
ATCGAAAGCCCACTTGTGCTCTGATGAGACTGAGACACAGTGCTTAACTCTTCCCATTCACC
AGAAGAATTCTCTTCTTCCTTGTCGTCTTCTTCTTCCCCGTCATCCTCTCCATCATCGATAGCC
ATATTTCTCTTAATCCTCTCAATCCTTCTCTCCTCACTTCTCCTCTCTTCATTAAACTTCGCTCT
ATTCATCTTAATTCTCTTTTGTACACTCGCTCCGAGTTGAGTGAACACTGTCTTTCCACCGAT
GTCCTTGGGGACTTCCATGTCATCAAGTATGAGAGACATGGGATCTTTTCTCTTGTACGTGCA
AGGATCTTGATCGTGCTGAACAAAAAAAGCTATCATCATTGATGCGTAAGATCTTTTATCAA
CATCAATTCCTTCTGCCTTGAACCTCAACCTGATGTATTTCATATCTTCTCTTTTTACTTTAAA
ATTCATAGACAATTCGCTTAGAGACATTACACCTCCAGGAATCTCAACGCCTGATATTCTCTC
GTTTTCACCTGACCTTGATTCGTCAATTAGTTCAATAGTCATTTTGCTCTTGTCTTTTCCATTG
TTGTGGAACAACGGACAATACATGATTTGAATCCTGTCAAATCTGACAAAAGGTTTTGCATC
TCCCATTAGACCAATCTTTTGAATGGCTCCAGTTATTGATTCCAACGTCCCGATAGATAAGCC
AGTTATTTCTTCCTCCTTTTTCAATCTGACCGTCAATGGAGTTATTACAGTCTTAATTTCTCCT
TTGGATATTTGGGTGAAATCTGCTGCTATGCATTCAATGTTTGCGTCCATTTCTCCTCCCTCA
GATAAAGAGATAATGTTGTGTACACCCTCTTTCATTAAATCGGCACTAGCACGTCTCGAACT
CAAGGTAGGAATTTTGCGGCTCGTGCTAACTATACTCTTGTCCTTTTTGGACTTCAATGACGA
CATCCTCTTAAAACTCGACATGTTGATTTCAAAAGAACACAAGATCTATTTTTCAG
>Seq73 [organism=Primula ophiovirus] RNA3 genomic sequence
AAAAAACATATCATGATTTCTTTAAAACGAGATTACACCTGAAGACGAGTTCACATACTCTA
TCTTCAACAATCGATCACTGTTTATTCTTCAACACGCAGACGCTTAAAAAAGGAGGAAAAGA
AATCTCAAGACATCATTCGGTCTCTTTCAGACACTTCCTTGAGCAACAGCGGCCACACCATA
GTAAGACCTGAGAGCTTCCACTGATACATCATTAAAATCTGCTTCTGTGTTCTCAAGGATGG
ACCACACGACTTCATCACCAACCTCTTCTCCGAAGAAATTTTTATCAATTTGGAAAGCATTGT
TTTTTGCATCGATTACTCTCCTTCTCATCGCCATTCTGCCATTGCTGGTCAACGACTCTATGAC
AGCTCTAGTAAGCTTTAAAGTCAGATTTTTGACAACAGGTCTCTTTGGGGCCATAGGATGCA
TTTTCGCCTGAGCAACATGATTCCCGTTTAGAGAGACAAGAAAGTTGCAGATTGCATGGCAC
TTATCTATCCTCTGATTTCTTGCCACAGCAGCGTCTATGTCTTCAACTCCTTGGAGAGATATT
TTGCTTCTTTTTTCCATATTGCATGCATAAGCTGCATAACGTATGGCCCTGTTCCCTGCGATA
GATAGTTTGCACCTAGAAGCAACGGCATGGGGAAGCAAATCCAGATTTAGCTCTAGGAAAA
TAGATGCCGGGAACTTGAGAGTAGAGGTTGAGGAAAGTTGAGCCGCTATTTCATTCCCTGTT
ATGGCCAGGTTTTTGTATATACCTTCTTTGACCAGTCTCGGGACCTGTTTTTTCTCATCTTGTC
CGACAGTGGGCAGGCTTCCTTGAGTCCAAAACAAGACAACTCCAGCTTTTATCGCTTTCTTT
GCATTGTCCACGTTCGTCGCCACAACTTTGGCTGTCAGAGATGTCACTTGTTTTAAAGAAAA
CATCTTCATCTCACCTTCATTCTCCCTGCAAGTCGCATATAACATAGCATCTAATATTCTAGT

TCCAGCTGAGATCAGAGGAACCAGTTCGTTAGAAGGTTCTTTGTCAAAGTGCCATATCACAA
TCTCACCCTTGGTGTAACCTTCTTCTTCCTTTGGAAGCAACGAGAGATAAGCTTCCAACTGAG
CTTTGAGTCTGATCAAATCTATATTGAAGGAATCATTCACACTCTCTTTCTTAGCTTCTTCAC
CTGTGGATATCACTTTGCTGCCCACGTACATTTCTTCTTCCATTTTTTCTTTGTTTTTATTTTGA
TTACCGCTTGAAGACTCTACGGCCTTCCTGACTGATTCAGATCTGTGTCCATCTTTTGACCAC
TCTGTGATTACGCCGTCTATTAGAGAGACAACAGAATCATCCTTCATCTCCTGCAGTTTGTGG
TAGTTCTCCACAAGCAAACCTTCATCTTCATCGATGAGGCCAAATTGTTTAAGTAGTCTAATG
TCTCCCACAGTCATTGATGCACTTTTTCTCCTTGCCATTGCATCCATCAATGAGATCTTGCTG
GTTTTAGACGCCATTTCTTTCAAGCTTGAAAAGTTTGGTTCCTTGAATTGCGAAATAGTGAAT
CTTTG
>Seq74 [organism=Pseudolycopodiella ophiovirus] RNA2 genomic sequence
TTGATGTCAACAATCGTAGTTTTGAAATAATACAATGTACTCCGTACTTGGCGAGCTTGGAG
ATCCCTGAGCCGCTCAGCTGATAAATAAAGTCCTGGACGAGACCAGGAGCCCAGGAAACAG
GGCCATCCTTTGAAGTACTACATTACTACCAATATCTCCAAGGTATCAGTAAATGATATTCTA
TGTAGACAATAGACATTTTAATTTTGATTATATATTCACCTTCCTTTTATATAAAACGATAAC
TTATCATAGTTAATCCTATATAAAGACCTTATAATAGCACGTTAAACAATCGAAACATAAGT
ATTTATTTTTCGTGATTATCGTATTCGTTATCATAGTAACTTATTCTGAATTGCAAGGTCTCTC
TATAACCCTCTTTCCTTTAGTGATAACTCCATGACGGCCTACTAACATATTAGCTTTATTCAT
ATAAAACTCTCGCCTCTTATCTCGGTTTCTAAAACTAGATATAGATTATTATCAGTTTTTTC
AGCGCATAATTTTAATTTGCCGGAAAGATAAAGATCAAAAGTTGATGTTTCTCATCTATAA
TTTCTGTTTTTATAGAATCAAAGAAACCTGATTGTTGTAGTGAACCAAGCGACAATATGTTTT
TCTTCACCTCGGATGTTTCAAACACATTGAGCAATTTGATGAAATCATTCTGTATATAAAACA
TTAATTCTTTATGCAAATACCCTTTGATTTTTTTGCCATCAGGTAAAATCATCTCATTACATTT
TGAAGATCTCTCAGACACAAGGATATGTTGTGAGGCCCCTGAATCTAGGTACCATTGTCTTA
TTTCTTCGCTTTCGCGAGCCACAGAAAAATCGGTATTCTCAATAGCCGATGATGAAATCATA
TTCAACTCTTGTTCAGATTTGTTCTGAATCATGTATTCGGAAACGTCTTTTTCTACCATCTTGC
TTGCCTTCTTTTGAACTTGTCTTTCTCTTTTCTCCAATTTCTTTTCAATAGACTTATTGAATTCT
TGAATTATATTTTTCACATTCAAGTTTTTAGGCATTTGTATGTCATCTAAGTAAATACATTTC
GGGATATTCTTTTCATATATTTTATTGATAAACTCATTATGGACATGAAACAATAATATTAAA
GTGCAAAAAGCCCTGCCCTTGACTGGAACATTAGAGAATCTATACTTTATTTTTATATGATCA
CTATTCTTATCCAACACAGAAAAATTCATACTTAATTCAGATAAGGCCATTCTGTTGCCCACA
AAGGTGACACCTTGGATTATCTGGTCTTTCTGATTAATTACAGAATCATCGATAAGAGTTATT
TCACCTTTCGAATTTGCACCAATGTCGCTATTAAAAGCAGGTAAGTATAAGACATTGATTTTT
TGATACCTGGTAAATTTATTCTTGTTACCAGGGACCATTTTCATGATGGTGTTCCATGTATTA
CTCAATGTTGTTAACTTCAAAGGTTTTGATCCTGATTTTTCGGTAACTTGTATTGTCATGATAT
GAACACCAGTGTAGATAGCATCTTCGACAAATTTATGAAAGTCTTCAGCCATATCTACCACC
TCTGAAGACAATGCATCATTGCTATCCAAAGATAGAAACCCCTTTGAAGTCATTACCAAACTC
ATCTTGGACAGATTTGTGAAAAGTTACACTTGTCTTAGCTTCTACCAAGTGCTTATGTGATGT
TGTGCTGAAGCGAGATGACGATGCATTTGAAGAAAATACATTTTCCATTTTTGCAATTTTGAT

TTTGTACTTTGTAGATTTATGATATTAGAAAGAAGTTAAGTTTATTTAAACTTATCGATCAAT
TATAT
>Seq75 [organism=Pseudolycopodiella ophiovirus] RNA3 genomic sequence
GTTATAATTAACTTATCGATCAATTATATTGATGTAAACAATTGTAGAAGAAAATTTATTTAA
ATCGAAACCGTAATACATGCGGTTTAGGAAGACTGTGAGTGTCAGCCCCACCTCACTTAGAC
TACTTTAAAGATGGAATCCAACATCTCAAGGGATACATCCATAAAGTTAACATTCTGATCGG
AAAGAATTTCCCAAGTCACCTCACCGGCCTGGTCAGTCGACCCATAGAAATTCAGATCTTTC
TGGAATGCAAGTATCTTCTTCGAGTCAATATGCTTTCTCATCGCGACACGGCCACTTTCGCTC
AATGTATGAAGTATCGCTTTTGTCATTTTCAGCGCAAACCCGACAGGAACTTTCTTATTCGGA
TTCAAAGGATGCAACATCATTTGGGCCTCGAAGTTGCCCTGTCTATCTTTCAATCCTTTGACC
AGTTTAAGAGCATGTTCCAAATTGCCTTTTTCAATTAGATCTTCCATTTTCGGGGGATCCGAT
ATAGGAGCTACAGCCTTGTCAAATCTGCTAGCTAACTCAGCGTATTTAATCACTTTGTTTCCA
GCCGGAGATAGTCGTATTCTAGAAAAGAATTTTGAAGGGAGATCTTTAATGTTGATCTTCAA
GAAAACAGAAGATGGAAATTTCATAGGATTGGAACAAGTAAGATCAAGAGCTACAGATTGT
TCAGTAGCATCCATACCGTACTCTAGATCTTCGCATATCAATTTAATCAGAGGTCTTTTACTA
GCCATGCCCACTTTAGCTGGCATACTACCTCGAGTGAAAATAAGGATTATCGTTGAAATAAA
TGCACATTTTGCTGTCTGCAAATTACTCATCACAGAATTGAATTCTAATTCTTGGATTGTTGT
AATTTCAAAAAGGTACTTTGCCTCTTCTTCCTGATGCATGGCCATATATATGATGGCGTCAAG
CACCAAAGTTCCAGCAGCGATCAAATTATGCACACTAACCGGAGAATCTTCGAGGATAGTG
ACTTTGACTTCACCTGCTTTATAGGTTTCACTTTCAGGGATTGATGTAATATAATCATTCATT
ACTTTAACAACTTTGTCAGCATCGAACTTAAACTTCGATTCCTGCATGACAGCATTTATATTC
ACGCTGTTGACCCATCCTGTTTTCGGAAGAGCCTTGGTTACCTTTTTCTTAGGATCTCCTCCTC
CTGACGAAGATGCCTTGTCTTCTTTTTCAGAGACCTTAGCAATGGCGCCTTGGAGTTCCTTCA
TTTTCTGCGACTGTCCCGTGGACATTTTCTCAACTGTGATACTGGGGTTACTTTCTTTTTCAGT
GATCTCGGGAGGCGTCATACTTACTATCTCAAAATCGTCGTTATCGTTCAATTGAATGGGCA
CATCACCATGGGCTTTAAACATCTGCTCCAACTTAGATAATCGAGACGATACGACTCTCTTA
TCAGCATTAATTAACTTAAACGCAGAAAGGAGAGGCGTGTCAGAGTCTGAACCTTCACCTGA
CCTAAGGCGCTTTGCCATCAACAGGATATCTTTCAGCATTGTCGTAGTCATTTTTGACGATAG
GCGCTATACCCTGAATTTGTGTAGGAAGA
>Seq76 [organism=Ranunculus white mottle virus_hele] RNA1 genomic sequence
TTTGAACCTTCGAACCCGGCGAGTCGCAATGACTGTAGTAGCAAACTTTTGAATCCTGTCAC
TTGTCCTATGAATTTTGATAGCCAAATATATAGTTAACTATGTTAGTATTCTTCATTATCATC
ACAAAACTCATTATCTCTAATCAGAGAAATCTTCTTCAATATCTGCCCATGCACCAATAATTT
CACTTTCTCCCATAAATTCTGTTAATTTCTCAAAAGTGTCTATACTTCTGTTAATCTCCATTTC
TTCCTCTGTGGCTCCTAGTCCTATATCTAGTGCACTTATGTCAGGTATGTTTGTTATACTTTCA
TCAAGTCTTCTAGAAAGATCTGATATTGCTCCATTAAAATGCCCTACAAAGGAAAAGTACTC
GGTATTTATTTCTTGAAAGAATTTTTCTAGCATCTTTTCATCATTCATTGATTGTGATAATCCT
CTCAGGTCCAATGATATCTCTTCTCTGCCTATGTTTGACCATAGCTCTTCGAATTTTCTATTAT
CTATTCCTAAAAAGTACGGTTCAGTATACATAGATCTGGAAGCCAACATTATTCTATTCATAT
AAAGTTTGTAATGTAAAATGTTTGTTCGTGTGGTTTTGTCTTTATGGTCCAAAATTGCTTTTC

GTTTAAGATTCCCCATATTAGCAACCACCAACAAACCTTCTATTATTCTTCCTGTTTTGAAAG
ATAGTATCGCTTTTGATGCTATTTCGTATGAGGAATTGATGGTTCTATCGATTGTTTTCTTGA
ACTTCTTAATGTTGATCATGTCTGTTATAGCATCAGAATCAATAACCTCTTCATTTCCTGACA
AATTTAAGATCAGTTTCCAGCATTCTATAGAATTCTTTTTATTGTTTTTAACATTCAAGATCTC
TTCCATAAACTTTATCAATTCATCATTATTCATATCCGTCACTCCCTTGACAATCACAAGATG
ATCTCTTATCACCTTGTTTCTAACTTCTATTGTATCACGATCATCTTCTCTGATCTTTCTCCCT
AGTTCGGTTAAGTTTTCAGGCAACTTCCTATCCTCTCTCCAGTTTCCGTTACAGATGATTCTTC
TGTCTAGTTCTTTTAATTTTTCTCTGTTTTTTCTGTAATATGTGTTTGAAACATATACGAGCAT
GTTATCTTTGAATTGATTCAATGATGTTATTTTATTCTTAATTTCCTGACTTACATGAACATAA
TCGGGATCTTCTTCTACCAGCATTTCTATTAGTTTATCATCATTAATAATTTCCATGGTTGGAT
CTTGAACTTGCTTGGGTGGAACTGTTATCACATTTTTCAAATCAACCTTTGATACTGCATTAA
CCAAAGATGAAGATATAATAGATCTATTATAGCCCTTTTTCTCACTAATGCTAGATGTGTTTA
TCATATCACTCTGTTTTGTGAAGTCTACTAGTAGATAAACATAACCAGGAGATTCTAATGCT
GGTAAACAGACTTTCAATTCAGATCCTCTCATTTCATCTAGCACTTTCTCATTTAGGAACTTG
TTTAATCCATTTAATCTCAATATCATTTTCTTCTTTGCAATTCTCGCATCCCCTATGATTCCAC
TCAAACAATCATGTTTGCTTGTAATATGAGAAATATCAAGCAGTATAATATTGTGATCAAAA
TAGCTTATATAATCTTGTTGTTTGAAACAGTTGAAACTAACCTTCTTGGATGTCATTCCTGGT
ATCCTCATGACTAAGTTGTAACCATCTTCTCTTGAAATTGTTGTGTGTTTTATATTGCTTTCCA
TCATCGCCAGATGAAAATCACCTCTTCCACCGCAAAGTTCAATAACACTTTCATCTTCTTTAA
TGAATCCATTCCCTTTCAATAATGATAATAGACCTTTTGCACTTGGATAAACAGATGACATC
GTTGGTGAAGATACCTTTTCAGGGCTCACCATTAATGAACATGCTGTTTCAAATCCTTTCAAT
GCAGAATCTATCCCTCTAGTATCTCTAAAATCTTCCTCTGAAACATTGATCTCAGTAAATTCT
ACTTGCCCGTAATGCACATTTTCTTTCTTTTCAGTATATGACTTGTGGAGTTTAAGGTAATAA
GAACCCAACCTCATCTGTTCAAACTCAATATTGCTGATCTCATCACAAAGAATACTTGCCCCT
GAGTGGATGTCTGAGTAGGTGTAACCCAAAGTAGGGAATGAATTTACAATCATTTCTGTAAT
TCTTTTATCCTTGATGTAAAATTGACAAACACCTTTCCTCATTTTCTCAAAATTGCTTTTGCTG
TTAATTATCGTTCGGTGCTTGTTTACTTGCAATCTGACAGTGTTGTTCTCTTGGTCTACCATCC
TGTATGATAGTGAAGTAGACAAAATGAACAATCTCATTAAGGCTTTCTTGAATCCGCTAGAA
TAACCAAACCTTGTCATTTCATCAGTCCATAGTTGTTTTCCTATTGAATTTTCGTTCACACTGA
ACATTTCTTCATAAAGAAGTCCTCTGATAACAGATCGTGAAGGAGTTTCATTGCCAATAACA
TTTAAAGACTTATAGTACCTGTAGAACTTATCAAAGAATGTCATCCTACCCTCCTTGAAGATT
TCTTCTCCTATTAGTCCTTTCAAGATATCATCTGATACTAATTCTGGACTAACTATCATTTGGT
CCACCATATAAGAGTGAACAGACCTAAACGCGCTTTCCTGATTCACAAAATATTCTTTAGTT
GTGGAAACATGGTTTATGAATTTATCTGCTATATCTTCATTCTTTTCAACAAAAGTAGAATAA
TTGTAATACATTTTTCCTCTTTCTTTGATCTTCTTCTTTGAAGCCATCTTATATAATTTCTTATC
GACCTTTATTTCGTTTAATCCTTTGATAACAAATGACACGTCCTTGATATGCTCAAGAATGTC
ATTAGTTAAAGTCAAAGGAACTAACTCCCAAGCACTTGGTTCTAAGAAAGCCAATCCTATTC
TGATATAAATCATTAAATATTGAAGGTTCAGATTGTTGTCCACTCCTCCTGTTTTTGCGATAA
GTTGATCAACTCCTGTTATGTCATAATTTCCGCTCAATAGATTGGAGGAAAGATCTCCAGTCT
TTGGATTGAATCCTCCTGTTAACGCTCTATGAAAATATCTTCCACTTTTGGGACAGACAACAT

TTTCTTCTATATCACTGTATTTGGCATCAGTAAATGTGTTCAAAACTGTGTCACAAATGGAAA
TCAAAGTTGTCTTATCAATTTCAGACATTCCTGATGAAAATTTCTCTTGTTCCATCATAAGCC
ATTTTACGTACCTAACCAAGTCATATGCCTTGAAGATTAACTTATGTTCGAACATTCCATCTA
CGCCTAATTCTCTGTCGAACTTAGCAGAGTTGTGGAAAGTTCTTTGTGGCGGCCTTGCTTTTC
TTCCTTCCTCTGTTAATTTAGTTGTGCCTTTGAATATCGGTTGGATCATACTTTTGAGATTTGA
GTGTACAACTTTTCCCATAATCGGTATTTCCTCTATTTCTAAGAATTTCAGTGATACAGATTC
TTTGTTTGGCAAGACCAATGCTTTGTTTCCTCTAGCAAACATCAAATCATCATAATTTATGAT
TGTGTTAGCATTAAGTTTTATTTTCATTCTAAAATTCGCCTGCCATGCCTTGTCAATCAACAT
CATCATTTTTCTTCCACCAAGAAGCATCTTCATTGTTGCTGCATTGTCTATCTTTGACAAGATT
TCTTGCACATACGCATATAAGCTTGATTCTAAGAACTTACCTGTGATTCTTGATGAGAAACA
ATTCTTAAATGCTGAAACCAATTGTCCTTTAAATTCCAATTCATCTTCTCGAGTATAGAGTGA
CAAGAAGTCTAATAATTCCTTGTTTGTAACAACTCTCGATAATATTCTTTCAATTTCTGGCCT
TACAAAAAGTGATGTGTCTTTTCTTCCCCCAATCATAGGAAATGGATTGTCTAGGTAACTGA
TAGCATTCTGTGAAAGATTGATTAAGGAACCCATCTTCTTGACGTTTTCAACTTTCAATATTC
CTTGGAGATAACTTATTCTTTTCACATTAGACAAACTATAGCCTGAAATCAATACGTCAGGC
AAAGGTGTTAGAGCAAACCCATACATTGTGTATGGAGAGTACAGAAACATAATAATGATGT
CCAAAACATCCTGATCATTTTGGTGGAACTGGAAAAATTCAATGTTTCTTGACCGAGGTGCA
GGTATAAACACTTTGTTATTCACATTCTTGTTGAGCCCTCTCAATGCTGATTTCATGCTGATC
TCATAGTTATGTTTGCTTGAAAGATATCTTTCATCAAGGTTATCTTGGAGTATTTCTTCAGTC
ATCTTTCTCAAAGAACTTGAAAGCAGTTTTATGATGCGGAAATTCCTAAGCAATGCCTGTGT
CCATATGGTTGAAGATCTAGTACAACTTGAGGTGAAACCCGAATCAATCAAAGTGCTAGATT
CTAGTTCATCACAAAGAATCTTAGGACCATTAGGGCTGATGGACATCATCTTTTTCAATGTC
ATTTCGATCGGGATACCCTTATAATAGTGTTCTTTCAACATAGAACATCTTCCATTTGTGACT
TGAGTTTGTGAAAGCTTAACAATGAGTCCCATATCCTCACCACATCTCTGAACATTCTTGATT
ATTCCGTTCAGTTTTTGTACATCCATAGACCTTTGAGTAAACACACCACATGAATCATCAGA
ATAGGTTGTTCCAATAAAATCCTTGACACCTAATTGCATAAACATATCTTCCAGCATCAACT
GAGATTGAATTCCCCATAGACCCCCTAACCAACCTTCAATTGCACCCAATTGTCCTTTTGAAT
AATAGATCATATCACTCCATGTATCTTCCACTATTAAATGGATGTTACTAAAGACATAAGGC
AGTGAAGTAAATTCTTCATATTCAAGCGATCCCTCAAAGTAACCGTACATATTGGCAATTTC
TTCCATTATAAAATTTGTATTTTCAGGCCTTTGAGACGTATTATGACCTGAATAATCCAAAAA
GAGGGAATATGTATCCTCCTCCAATAATTTTTCTGACATTTTAAACATAATTTCTCTTCTTTCA
TCATCTGTCATGGTCATCATTTGTCCAGGTAAAAGTTTCATTGCCCTTTTTACCATCTCCATTG
TTGAACTAATCCAGAGCTTTAACTTGAAAGAAGCAATGCCGTAAAATCTAGCAGCAATCTTT
TGTTCACGTTCCTTTTCTGAGAGTTTGACTAACATATATTTTTCTATGTTTTTCTGCAGACCTT
CAGACCTATGGATTACTTCAGCAATTCTTGAGGATGACCTGTATGCATTCTTTTTGATCCCCA
TTGGTTCAGAGAAGACATCTTCCATGTTATGAGTGATATAATAATGAATTTCTTTCTCAGAGT
TACTTTCTTGGGAATAATTTTCTTTTGAGCAAGCCTTATCTATTATTCTTGACTGCAGATTCAT
TTCTTCACCAGGCTCCAGAAACTTGCCTAATTTCACTTCATCATATCTGCTAGTTTCAGATAT
CATCTGATTTATATAATTACCTCCTGCTGCCTTCATTTCTAACTGAGCACCCAAATCTTCAGG
AACACTTACTAAGTTCGGGACTCTTCCGTGTTTTTAATGTAAGCAGAAATTACTCTTTGTTT

GAATAGAGACCTTAACCTCCTTACCGTGTTTTCATTAACTATGTTGTCTCGATTTGTCCGTTG
GGTATATCTACTAATCCCTTTCCCTGGTTTTGTGCTTGCAATGATCAATGTTTTCCCTATGGA
AGACATTCTGATACAAACCTCAGGCTCTGATTCTTGACAGATCTCAATGAGTTTTTCTGTATG
GTTGAGAGAAACATTAATCGATTCTACAACATTATCAAGAATTGGCATACTCGCTGAAAGAG
TTTTCATGTCAGAAAGAAGAAGACATGTTGTTTCATAAAGACCTCCCATTCCTATCCTGATGT
CTTCGGGTTCATACAACAGACTAGTGTAGTAATCCAAAAACTTCTTTTCAGATTCTGACATTT
GGTTACGAACGATGCTAAGTGACAATAGATTATCTGCTAAGTATAAGCTGTACTGTAGCATA
TCAGGTCTTCCCAAATACACTTGAGAACCATCGTCCCAATAATAAGCACAAATTGTAGAGGA
CAAAAAGATGATTAGATATCTTCCCTCCTTTTCAAACTCGACTTTTGAGATTGAGGCTCCTAT
GGGAAAATATCGAGTGTTCAACCTCTTTCCTTCATAATGTTCAATGCTTATGTCCAACTCACG
ATTGTTGTGTTTGGCACTGGCGCTCCTCAATATGTTCTTGTTTAGGTGCTCAAAAATATCCAA
GACCCAATAATTTTTTGCAGCATCTTCACTAATTTCATAAGACATTTTTGTTCTTTTCTTTGTT
CCTAGAAGTTGTTTTTCCAGGAGAGAGATTTCATGGTTTATTAGATTTCCGGAAAATTTCAAA
AATACTCCTGATTCACTAGAAGCAAGACTGAGTTTCTTAAGTCGTTTGATGAGATCTATCGA
ACCCTTCTGTCTGAGTTCAGGGTTTATAAGAGGAATACAATCGTCTCTTGCTTCCTTGAGGAA
AGTTTCTCTGAACAAAAACAATTCCAACATATTCTTTTCTGACCATTTTTTCGGAAATTGTGA
ACCTAAAACAATAGAAGCATTGTGTTGAGTGATTTCCTTATAAGGACTGTCAAGTTTTACTA
AAATACACGGTGTCCCTATCTTATTCATGTTTAAGAAAAAGTCTTTGCTGCAGTTTTTCAGGT
CAACAAGTTTATGTTCAAAATCAATTTTCACTTCATCTGCTTCTTTCTCTTTCTCGTTTTTAAT
TGTTTGAGTGAGTACATCAAAGTCATTGACAAATTTACTCATTACCTCCCTGGTCGATGGTGT
GTCACAATAATTGATATTTTTCTCCCTAAGATATGCTTCCATTTGATTTGCTCAATTTCTACTA
GTGTTATTGGTTCAGAGTTACTTTTTATTTAAAACGAAGAAAAACAAACTCAATTCATGATTT
GTTTCTTCTTGATTTCTTTGTACTTTTTGTCCCTGTTCTCTTTTATCTTCTTTTCCTCCAATGTTA
GTTTCAAACTATATTTGGGCGGTGTAACTTCATTCATTATAATGAAGATTCTTCTGTCTTTCA
TAAGCAAAATTAAGTCAGATTTATAAACTCTATTATAAAATGATTTCCATGCTTCTTTGAAAG
AGTTTGCCAAAGTAATCTCAATCCCCTTATGAATATCACCATCAACAATTTTCCCATGTGTTT
TGATGATAATTATTCCCTTCTTACAAAAAGGATTTTCGTTCTCTTCAAGTGTCAATAACCATG
AACCATAGAGAAACCTATTCTCTTCATTATAACTAGTTTTATACTCAACAAAATCAGCAAGA
ACTCTCTCATTTCTTTCTTTTATCTCTTGATCAAGATGCACTATTTCCTTGAGAACAAAACCCT
TTATTCCAGTCATTCGAATTTCATTAGTCATCCTGTTATTATTTTCTTGTTATATTCTGATAAG
ATTAACTTTTAATATGTGAATTTTCGAAAAGGTTTA
>Seq77 [organism=Ranunculus white mottle virus_hele] RNA2 genomic sequence
AAATTATTCTCTTTCACACTTGGAATGACCTCGCCGTCTATCGTGCTAATCCCTCATTGAGCG
AATACGAACACGGTGCGATGACTACGGCTCATTCCGTAAAAGAGTGAACCCGGACGGGCGG
GCCGCAATGGCCTTAGTAAAATGGATTAGAATAGGAATATAATTCCTACCTTCTCACCCAAT
GTGTTTATTTAAAACAAGAACACACCACACCGAGTACAAATGACAATACCACACACAAACA
AGCGATAATCAACAAACCAGAACCAAATCTACTTTCTATGAAGTCTAAAAGACACATTACCC
CATATAGGGTATCACTCAATGATCATTTTACTACTGGAACTATCGGAAGCATCAAAAACATA
ATAAGCTCCATCGTGAGTCAGACTGAACACGAGTTGATCATCGAGACATAGAGTGGCCTTGT
TGTTTGGAAGAGAATTAAATTGATTTATTATTCCTGATTCAGCTAACTTCCGATATGATATTA

GAGGTATATCATCTTTCTTACCATATAGGACCTCTTTCAGTCTCACCCAGTGAACGCCTAATT
TGATCTCAACCCCATTGAGACGATGGAAATTAGTGCCTTCAGCAAGATTATCACCTTGTGTA
ATCTCTTTTAGTTCAGCAAGGTCTGGATTGTAAAACCAGTGATCAGGGGCACCTGTATCGAG
GATGATTCGGTCACTGTTTGTGAACGGGAGTCTTCTTGGAAGGAAGCTTGGAATGTCAGAAT
TATAAAGCTGCAAGTCATCTGCTACAACTTTCTTTCCATCAAAGAAGCCAGAGTCTATCCTAT
TAGGTTTCTTTTCATTGGGTAAATCGAAAAGGATTGGAGCAAATCTCCTTTCTTCTTCTTCAT
CCTCTTTGTCTTTGTTCTTCAAAAATTTTCCTTTGTTCTTCTCTATCCGGGAATTCACTCTCTTC
ACAAGACCTTCTATACTTGATTTCTTTGTTATATCAACCGGGTGCTCTAGCTCATCAATCAAT
AGGACAATTGGTTTCTTCTGAGCGATAGTAACTGGTATGAAGTCTCTTTGAATATAGAAAGA
CAGACAAACAAACGCAAAAGCTCGATCCTTCACAGGAACTCCTTTTGCTTTGTACCCAAACT
CTATCACATTCAGCTTTTTCTTGTCAACAAAATATGATGAATGAAGTTCAATCATACTCATCT
TGTTCAGAGGAGCATCTGCTTTTGTGACAATCTTTGAACCATTTCTAATTGAACTGTCTCTGA
TAGAAAACGTGATTGCTTGATCTTGAGCCAGCTCTCCTGAAAATAAGGGAAGATAGAGGATT
TTCAGCCTGTCCACTCTGTAATAAGGATAATCCTTTCCAAACAAAACATTTGCAACAGTCTTT
AGTGTTCCCATAGTAACTTTCGTTGTATCTTCTTCACCTTTCAGTTTTATCTGCATCGGGGAG
ACAACTGATTGAATGTCACCTCTGATTGCTTCTTGGAACTCTGCAGCCATACATTCTACTTCT
GGACTCAGATTTTCTCCCTTGAGTGATGCTAATATAGAAACACCATCTTCTACCATATCCTCT
GCAGCTTTCCTAAAAGAGACAACAGGATGACTTGAGGTAGTTCTAGTTAATTCTCCTGTTCT
CTTCAAGATCCTGGAACTATTCCTTGAAACAAGGTTGGACTGAGATGCCATGGAATATTGAT
TGGAGATAACTTGCACTTTAAGAAGATTCGTCTCTTTATGAGCGGATAGGTTTCCTCTTGAAT
AGCTTAACATGTAC
>Seq78 [organism=Ranunculus white mottle virus_hele] RNA3 genomic sequence
TTTTTTTTTTTTTTTTTTTTATTTAAAGCTATCTTGTGCAACAGAGAACATTCCACTCGTAAT
GCCGTTCATTTCTAAAATCGAGAAGCATTGTCGCTCATGCAAACTTATTCTGCCAACTTTCAA
CCTTTTAAGTGGTCAAACCGTATGCTGCCTTCACAGCTTCCATGGATAACTCGGAGAAATCA
GCCTCTGGATTCGACAACACAGGAAAATGAATCACACCTAAACTGTTTCTTTCCCGTAAAT
GCTATCATCTCTTTTGAATGCTTCAATCTTCTGGTCATCTATCAACTTCCTCATCATTTCTCTT
CCTTCGGTTGAAAGAGCATATACGATCGCACATGTCATCTGGAGTGTGAAATTCTTCCGAGA
TGGTCTTTCGGGTGAAAGTGGGTGCATCCGTTTCTGAGCATCAAAGTTTGATCCTAAGGTAC
ATAGTGTGTTCACTATGGCTTTAGCTTTATCGAGTTTCTCATTCTTCTTTACATACTCAAGCAT
TTTTGTTGCATCCGTAGGAGCTGAAATCATGTCTTTGGGAAACTTCTGTGCAAAGATAGCAT
ATCTAATTGCTTTATTACCTGCAATAGACATCTTGCATCTCGAAGAAACTTCGGTAGGAACA
TTGTCAAGCGAGATTTTTAGGAAGACTGAGGCAGGAAACAAAGAAGGATCGGCAGAAGAG
AGATGAACACACAAATCTTTTGTCTCCATTTTCGCGTTATTGAATATTGTTTCTCTAATGAAC
TTTGAAAGGGTCCTTGATTCAGATGACTTCTTTGGCAAACCTCCTTGATTATACACAAGGCA
AAATGCTGCTTTGACTGCTTTATTGCCAAAATCAATGTTCTCAGAAATGGTTTTACTAGATAG
ATGAGAAGGCTTACTATTGTGAATGGTGAAAATGAATTGATGCTCAGCAGAATCCAAGTAG
GACAAGTATAATATAGCATCGAGAACTTTGGTTCCAGCAGACAAAAGAGAACTCACACTTA
ACTCGATACCTTCAAAAGTCTTGATCTGAATTTCACCTTCTTTGTAGGTCTCAGCACTTTTCG
GGATTGTTTTTACATAGTTCTCTAGAGTCAGTCTGATCTTTTCTTGATCTAACGAAAATTTCTT

TCCATCAATTATTTGTGAAGTGCTTCAGCAGAAAAGACTATGTCCGCAACATGGCTAACAG
ATTCAGTTGGTTTAGTTGTCGAACTTTCTAAGAACTTCAGTTTCCCTTCCTCATCAAGATACA
CTACCGCAAATGGTGTCTTTGCTTCAACAGCTCTTAACTGTTTAGTCTTCTCTTTGTTACCAAT
CTCATCATCATTGATCAAACTAAGGCTTTTGAGAATCTTCTTATCCTCAATATCCCAAGTTGA
CTTTCTCACGATAGCCAACACCTGTTCAACGGTATATTTGCCAGACATACTTTGCTAATTTTT
GAAAATTTCGAGATATAAGATCTTTAATTGTGAGTTCTTGAGTAAGTGAGACGTTTTGCT
>Seq79 [organism=Ranunculus white mottle virus_hele] RNA4 partial sequence
CTCTGTACACCTTGTCAAGTTAAGACGGGGTCAGAGAAGGGCCAGCGGAATGGAGGCCAAC
GGCAGCGTTTAAAAGAACGAACATGGTTAGAGGGAAAGAAATTACTTTAAACAGTTAACGG
CANNNNNNNNNNNNNNNNNNNNNNNNNNNNNNNNNNNNNNNNNNNNNNNNNNNNNNNNNN
NNNNNNNNNNNNNNNNNNNNNNNNNNNNNNNNNNNNNNNNNNNNNNNNNNNNNNNNNNNN
NNNNNNNNNNNNNNNNNNNNNNNNNNNNNNNNNNNNNNNNNNNNNNNNNNNNNNNNNNNN
NNTCATTAATTTGCTGTCAAATGCGCTTGTTGTCTTCAATACCCTCAGCATAATAGAAGACTG
GATTGTAACCTTGTCAAACATGGTTGTCAGTCTTTCAAAATTCTCTCTATGTGTTCTATCATG
ATAGATCTCAATCAATAGACAGTAAGTATATTTGATATTGTAATAATAGAACTGATACATGA
ACTTATCAGTATGTATTCCTAGATCATTAGAGAAATAATTCATAAAGGTTATTAGGGTAATA
CATTCCCGCTGATCAGGTGTTCTAATACACTTTATTCTTCTGTTGATGTCGTTCCCAGGATGC
CTGTGATTCTGAAGCAAGTTGTCAATTCTTCTTGGATTGTAATGGATCAGATAATCAATTCCT
TTCTCAAGATCTCTGATCAGACTCTCCTTGATTGGATTGTAGAAATAGACTGTTTCATGTAAA
GAGAGTGAGTGTTCCATTTCTTTCTTGAAGAAGTTTAGGAACCTGTTTGTTGCAATTGGAGTA
AAATTGGAATCTTTTTCCATAAATTCTTCTAATACTGGTTTTATAAAGTCAGCAAGGAGCTTT
TCTTCTAAAGTGTTTAAGTTCAGATTGTACTTTTCAATTATCATTCTTCTTTGTTTTGCATCAA
GAAGAAAGTTTTGATGCTCGGGTTCGCCTAGGTATTTGAATTTTTGGTCAATAAACTTGATCC
TATTGGTCAAGTTGTTTTGATCTAATGCATCGAGAATGTCTATCCAATTATGGGGCTTAAAAG
CAATGTTGATGATCTCCTTAATATGAGGTATGATAGCCATTGCCTTACAAACCAGAGGCTCC
AACATAACTACCCAATATGCAAAAATGTTCATTGTGCGATGGTAATATTCTTC
>Seq80 [organism=Rhododendron ophiovirus] RNA2 genomic sequence
CTGCTCATCCATTCTTAAGGGAATTATGATTCCATGATTTGCCAATCCCTACGCAACACGAA
GAATATCTAAAACGAAACCCCGAGAGGCGAAACCCTAACGATGTCACAGTCGATCTTTAGG
AAAAAGCCAAATTATAGAACTTTTTCTAAAGAACTGATAAATCGTTGGATGTCACCACCCAA
GGATGACCCGGTTTCCCAGGGGAGCGCATTGCTCCTGGAAAAGAGGCAAGCCTCAATTCAA
CTTTGCATTTTATTTAAAACGATTCTAGCCAAAATATAAAGACTAGTAATCGACGACACAAA
AGGTAAAATTCATTAAACAATTCAACAAAACCACACAGGACACAAAAGAAACACTCATACT
AGAAACCAGATAAACGAAATTATTTAAACTCGTGAAACACACTTAACTGTAGTGACATTTTG
AAGATTCAAAATACCAGTTTCCCAGCATCATCAACACTGAGTTCCCAACAAATCTCCCCATT
GAGATAAAGAAGACCCACCTCTTTTTGAATTGTCAGCTTATCCACCAGACCTTCCCTCTTTAA
CGCTGCAAGTGACACTAGATCAAATCCCATATCGGCAGTCGTGATTTCGGTTTCTGTGAGGA
TGACTTCATTCTTTCCGAGCCTGACGCGAACTCTTGGAAAAACTTTGTAAGGAAGATCGGAC
ACTCCACCGACATTCCTCATAACTTTGTCAGTCACTATATTTGGTTTGTAAAAGAGATGACCG
GGCGCACCTGTGTCCAGCATTTTCATGTTTGAATTCTCGATCGAAATCACAGCCTCAGAGGA

TGCAACTTTCGGTTTCATGAGAGAAGATAGATCAGCAGACATTTGAGACCGGTTATCTTCTT
CATCTATGCTTTCCTGATCAAACTCATCCTCTTCATTTTCATCCTTTATTGCTACACTCTTCAT
TTTCTTGTTCAAATATTTCTTCTCCTCCCTTTTTATCTTCTTAAAACTTGCTTTCTTTTTCATTA
CGGATGATTGGACAGCATCCCCCAATTGGGAAAATACAGTTTTTGAATTGATGTCTATTGGT
CTATCTAGTTCGTCCAAGTAAAGGGTTGTTGAACTCTTTTTCTTGAAACTTGCACTGTTGAAA
TCTTCGTGCACATAGAATATCACCATCATAGAACCATAATCTCTATTGGTGATCTTAATCCCT
TCTGCAATACATCTCAATTTTATCTTGTTCATGTCAGCAGCACGAACAAAATATTCCATGCTT
AACTCATTTAATGACATAGAGTCAGCTCGGATCAAAGTGGAATCGATTACTGATTCCTCATC
ATCATCATATCCCTCATCCACCAGTTGTATTTCGATTGTGCCTCCAGGTTGCCCTTTCTCTATG
ACATTGTAGTCAATCTCTCCTTGTGTCTTCTTTCCCAAATTGAAGTACAGGCATTGGATTTTG
TCAAACCGAACAAACGGTTTGTGCTGTCCAAGAGTTCCTCCGCTGATTTTCTTTGCTGCGTTG
AGAATTGATGATAGGCTACCAAGACTTAACCCTTTTTCTATCTCGTCCTTCTTCATCCTCAAC
GTCATTGGGCTCATCAATGTATGAATTTCCCCTTTGCTAATTGCTGCAAATTCAGCAGCCAGG
CATTCTATCTCAGGGGATACATCTCCCATGTCCTTCATAGATATCACAGTTTTGATTCCTGAC
TTCATCAAATCTTGAGTTGCTATTGCAGCAGAAAGTTTACCTATATTCTTAGCCTTCACTTTT
CCATTTGAGCTCTTAACAGCGCTAACCCTCTGAATCGACGACATGGTTATCGTAAGTCCTTGC
ACAGAGATCTC
>Seq81 [organism=Rhododendron ophiovirus] RNA3 genomic sequence
GACAGTTCAAACATGCTCTGCAAAACAGGTCACAGCATCTTGTAATAACCCTTCAGTGCTGC
CAGGCTAAGTGACCTAAAATCAGCCCGAGCATTTGTCAGAATGGGCCATACACGTTCTTCGC
CCATCACAACACCGTAATATCCAGTATCCAAAGTGAAAGCAGAGTTCCTTTCATCATCAACC
TTTCTCACTAATTGATACCTCCCCACCACAGACAAATTCTCTATTATCGCTCTCGTAAGCATC
AAAGAAAGATCCTTGACCACTGGTCGATCTTTCCGCTGTGGATGCATCCTGGCCTGTGCGTC
TGTGTTTCCCTGAAGACTAACCAAGAAATCAACAATGCTTCTTGCCAGATCTATCCTCTCATT
CCTCGTCATTGCCTCTTGAATATCAACTGCAGTCATCCCTTCAACCACAGTTCTTCTAGCTTG
CTTTTCACAATCAGCTGAATATATGGCATATCTCACAGGCCTATTTCCAGCGACAGACAATTT
GCACCTTGATGCGACACCTTGATCTAACTCGGAAATCTTTACATCCAAGAATACAGAACAAG
GAAATTTCAGTGTTGACGTTGAGGAGAGTTGCTGAGCAATCTGCAAATGATTCTCAACTTTT
CCTCCATATATGTAATTCCTAACAAATTTTGACAAAGACCTCGATTGTGTAACTTCTGACGGC
AGGCCACCTTGCGTCCACACTAGCACCAGAGCAGCTAGAATTGCCCTCCGTGCTTCCTCTAA
ATTCTTAGAAATGACCAAAGAGTTCAATTTTGAGACAGAGACAAAGTGGAACATGCCAACC
TTTGTTTCATCTTCAACCAATGCACAAAACAACAACGCATCCAAGACCTTCGTCCCTGCTGC
GATCAGCAGCTCGGGGTTTTGCTGAGCTGCCTGGTCGAAATGCATAATTTTAACATCTCCCTT
TTTATAACCTTCACCCTCCCTATGAGACTTCTTGAGAAACGTTTCCAGTTCTTCACGAATCTT
CTTCTCATCTAACGCGTAATTCGATTCACAAACCAAAAGCTGTGCTTCAGTAGAAGTTGATA
CAAATTCTTGGTTTATGTCATCTCCTATTTCATCTTTTTTTACTTCTTCAGATGTGCTGCCCTTC
AAGCTCACAAATGGAGTTCCAGTCGAATGTTTCTCTAATTTACCGGCAATCAAGGACACCAC
ATCCGAGTTCTCCAAGCTGACCAACAGTTGATAGTTATCAGCGCACAGACGGCCAGTCTTGT
CAGTTATTCCGTACTTCTCCAACAAGCTTATATCTTCTCCACTGGGAACACCTGCTTTCCTTCT

ATCATTTGCTTTGATCAAATGTTCCACCTGAATGCGATCCATAATGACGTGAACAAGTTCAA
ACGCAAGAAAATTAAGATCGGAAGAGCACAC
>Seq82 [organism=Silene ophiovirus] RNA1 partial sequence
TAGGTGACTAAATAGTCAAGTATTTGGATTTTAAGGAATTGATAATTTAACAATTTAATAAT
ATTCATCCTCATCATAATATTCTTCAGCTAACATTTCTAGTAATATATCACTATCATTAATTAT
CTCTTCCATTCTTTTCTTGTTAGATTCCATTTCTTCTGGACTGCATAAGAAATTTTCAAAAATG
TTTGTGTCAAGCGTTATCTCTAATTCTCCATTAGAGTTCAAAATATAGTGATTTTTCATTTTGT
CTCCCAATTCGTCAGATATACTGTCAAAAATTCCTATCCCTTCTCCTGAAAAATATTCTTCAG
TGCTTTTATATTTTTCAAGCAATTGAAGTATTTCTTTGTTTATGTTATTATTGTTGTCAAAAAC
TTGACCTTTGATTTCATTTTCTATTCTATTGAACCTGCTAACAAAATTTTCATTATTTTCTCTT
CTCTTTATGTATGAATAATTCAATAATTGTATAATGTTAATTACTCTGTTAGCTATCAATTTAT
AATTTACTATGTTATTTTTGTTAGTTCTTGTTTTCACTTTCATTAAATTTTGAAGTTTCTTTCCT
TCTAAATGAGATATAGTCATTAATCCTTCCATTAATTTCCCTTGAGCAACGGCATCACATACT
TGATGTGCAATTGTATACAAACTCTCCCTACCAGATGATATTCTCTGATATTTTAAATTCCAC
AAGTCAATTGTTTCTATGATATCAATACTTGAGATTTTATTTTTCCCATTAGTTATGTAAAAC
ATTTCCCAAATGTCTCGGGTCAGGTAATTTCTTTCATTTTCCAGAACAATTTGTGTTAAACTT
TTAAGTTCTTGTTCCTCTAAGTCTTTGTAACCCATAACAAATGTTCGAGGATATGATTCAAGT
TCTTCTTGAGTTACTAGGAATTCTTCTAGATTATTATTTAATGCATTATTTATTATCTCTCTCA
GATCGGATTTTTCTGTTTTGTTACCATCTGAAATGAGTTTTACATTTAGTATTTCTAATTTATT
TTTGATAAAATTCATCTTATTTGTAAACTTTGTTATAAAATTTAACTTCAAATCCTCTTTGCTC
TTTATTTCTCTAATGCTGTTGGGAACTATTATTTTGTTTTCTTCGACACTTTGCATTATGTTCA
CTAATTCTTTATTGCTTAGAAGTTCTTCTGTGTGATCAGATATTTCTTCTCCTTTGCTAAAATT
GCACTTGAAAGTCAAGTCTTTTGTTTTCATGGTTAATTTTTCTAGTATTATTGAGTCACTAAA
AGACTTGATTTCTTTTTCCTTGCCTTTATATTTTTCCATAGAATTTTTGGATGGATTTATTAAT
ATATAAATTAGCCCATTTGAATTAACTTCTGGTATTAATATGGATGTTGCATAATTTTTCCTG
CTAATCAATTCTATGAATGGTTCAGAAAATCTGTCTGATGTAGTGTTTAATCTTATAATTGTT
TTCATTTTTTGTTTTAACCCTTCCATCACCAAATTGCAAAAATCATCATCCTTTCCTGTAAAAT
GTGATATATCTAGTAATAAACAATCTCCTACTAAATATCTCATGTAATTTTCCTTTTTCAATC
CATCCCAATCTTTCTTTTTCTCTATCATTCCTACCATCCTATTAATTAAATTATAACCATCATT
CCTAGAAATGCTTTCATGTTCTATTTCTAATTCTGTCATTGCTAAGTGAAAATCTCCTCTTCCT
CCGAAAAGATCCACTACTTTTTTGACATTGTATTTTTCTTTCATTCTTTTCATTATGTTTATTG
CACTTGAATATACATCACTTTTAGTTGGTGATGAATACTTTTTAACAGAGAAATTTAATGAAC
AAATATATTCAAAACCTTTGATTGCTGCTCTCATTCCATCTTTATGTTGTAATTCTTTAATATT
TAAACTGTGTTCTTCGTAAGATGTTTCTTCATATTCAACTTGGTTGTCTTTTTCTATATATGAT
TTGTTTTGTTTTATGAAAAATTCTAATTGCCTAAATTGAGTAAATGTTTTCCCTTCTAGTTCGC
TGTATAAATTATCTACAGAACATTTAATGTCATCAAAATCATATGACATTAAAGGCATGCCA
TATATTATCATTTCGGCAATAGTTTTGTCTTTTATATGGAAGTGATGATCTCCAGCTCTCAGA
TTTTTTATGCTAAACATTGTGTTTCTTATTGTTCTCTCTTTGTTAATATGAATTTTAATTTCATT
GTCATTTTCAGTACTTATTGTGTATATCATTCCGCATGATATAACAAATAATCTTAATAATTC
TTTTCTATAATCTAAACTGAATCCTTTAGTTATGATATCATTACACCACATTTCTCCTTTTTCA

TCTTTTCGGAAAATTTCTCTGTTTAATAATCCTCTTATAACAGCTTTCTGAGGAGTTTCTTTTC
CGATGACATTCAATGATTTGAAAAATTCATAGAATTTGTCAAAAAATAAATCTTTACTAAGA
TATTTTTCTTTTAAATGTGGTATCAATTGCAACAAAATATTATCGTTAATTATTATGGGAGTT
ATTATATTTTGATCCTCCATGTATAGCTTTATAATTCTAAAATCACTTTCTTCAACTATCAACT
CCTCTGGTACCAATTCTTTTATGCCTATGTATCTCATTTTCAATTCTTCATCAGTGCTAATGAA
TGTAGAGTAATTTTTGTATATTGATTTTGCTTTTTCTAATTTTTCACTGTTGAATTTTATTCTGT
TTTGATAATTGTAACTTGGAATTTTTAAATCTGTTAATTCAAAAGTCACATCTTTTAAATATG
GTAATATTTCATAATCTAAAGAAAGCGGTTGCAACTCCTTTTCTTCAGCTTTTATTATACTTA
ATTGTATTTTACAGCTAACCATTAAATATTGCAAATTAAGATTGTTATCTTCTCCTCCTGTAC
ATGTCAGCAATTTTTCTATTCCTGTCATTTCGTATTTTCCAGATTTAATACTCAAAGATAAAT
CTCCTGTTTTTGGGTTGAATCCTCCTGTTATTGCTCTATGAAAATATCTTCCTCCTCGAGGTGC
TGTTATACCTTTTTCAAGTAGTTCATATTGTAAGTTAGAAAAAGTTTTTAAAGTCATGTTGCA
TGTTTCTATAAGAACTTTTTCTGTAAAGTAATTAATTTTCGAAAATTTTCCCAATTCATATAT
AATCCATTTAGTGTAACGACAGATATCAAATGCTTGTAATATCAATTTTGATTCAAACATGC
CTTCAATTCCAGGATCTCTATCAAATTTACCAACATTGATGGCTGCTTTTGCTGGTGCATTCC
TCACAAGACCTTTATCAGTCAATTTGTGAGTGCTTCTGATTACCGGTCTAAACAATTCATATT
CTTTATTTGTTTCGATCAGTCCCAATAGTGGTATTTCTTCAATTTCAATGAATTTTATTTTTAT
TTTTTGTCCTTTACTGTTAATTTTTTCAGTTTCATTTCTCAAATTTATCATTTTGCTATAGGAA
AGATTAGGAAATAAGATTTCATTTTTTATTATTTCTAACTTATGATTTCTCGTCCAACATTCAT
GCCACGCTCTAGAAAACTTAGATTTTCCTGCAAAGAAAACTAAAGTGCTTGAGTTTTCTACT
TTACTTAAGAAATAATCTATGTAACCAAAAATAGAGTCATCTTTATATTTACTGACAATCCTG
CTGCTAAAACTGTTTGAAAAAATTCTTACTATTTCATCTGAGAAATTGTTCTCATTTTCTTTGT
TTCCTTGTTTTATTCTTTCATTTATTTTTCTGTTCTTTGAAATTTTCGATATAATTGATTTTAAT
ATATCTTTCATTTTTGTACTTGTGTCAAACCTTCCTCCTGTTAATGGAAAAGGGTTATTAATA
TATGAATGTGAATTTTTTGAAAAATTTACTAACATTCCAAATTGTTTTCCTAAATGTTTTGGA
ATTATACTGTTCACATAACAAATTCTTTTTATATTTGATATAGAATATCCAGATATTAAAGCA
TCAGGCATGGGCGTTAAAGAATAGCCATACATGGTATAAGGTAAGTACATCATGCTGAGTAT
CATCTCCTTTACTAAATTATTTTCTAAGTGTGTTAACATAAAATAATTATCTTCATTGTTAAA
ATTTGGTAATTCTGCTCTGGAAAAATTTCCTGTTGATCTTATTTTCATTTTGCTTATTTCTATC
AATCTTTTATTGTTCTTTACTAACCCATTATATCTTTCATCTAATTTCACTTCGAAATTACTCA
CAAAATGCCTAACATTCCTAATAGATATGTCTAAGAATTTTGCGTTTCTCAAAATAGTCTGA
AGTCGATGATTCTCACCCATAGAAGAAGATGATGTGTATCCTCCATCAATTAGTCTTGTCATT
TCTAACTCATCACCCCATAAGTAACTAGATAAATTACTTATGCCCATCATTCTCTTATAAGTG
TTTTCAATTGGAGTGTCTTTCCAATAATGTGTTTTCAACATGGAGCACCTAATTGAGCTAACT
TGAGTTTGAGATAATTTGACTAGTAAACCCATGTTTGAAGCAAAATCTTGTACATCTCTTATT
ATTTGGTTCAATTTATATTCGTCAATTTCTTTTTCTATGAAAACTCCACATGAATCATCAGAG
TAGGTTGTGCATTTTTTACTCTTGAAGCCTAATCTTTGAAATAAACATTCCATCATCAATTGG
GATTGTATCCCCCATAGTGCACCCAACCAACCTTCTATTGCTCCTTTCTGTCCTTTAGAATAG
AACACTTCATCACTGTATGCATTTTCATATATTATATCTATGTTAGAAAACACATATAGCAAG
GATATGAAATTATCATACTTTTCTTGATCTTCATCTAAACCATACATAGCAGCGATCTCTTCC

ATCATGAATAAGTTATTTTCAGGTCTTTGTGAAGTATTATGACCTGAATAATCAAGAAACAA
GCTGTAAGCATTAGGATCTTTTAACATTATAGACATTTCATACAAAATTGTTTTCTGTCATC
ATCTGTCATAGTCATCATTTGTCCTGGTATTAATTTCATAGCTCTCTTAATCATTTCCATACTA
GATGATATCCATAATTTTAAATGGAAGCTAGCAATTCCAAAAAATCTTACTGCTTCTTTTTGC
TCTTTTTCTTTTCCTGCTATTCTTACTATCATATTATTTTTCATTGTTTTAAAAAAGGATCTTT
ACGATCAACGTTTTTTGTTGTCCTATTTCTGGAGAATTTAATGGAATTCTTATTATATCTTGA
GGGTCTTTGATTTCTTCTTGATTTAAATAATATATTAATTCTTTAATTGAGTTCCCATTCTCAT
CATAGTCATCTTTAGTGCAACTTTTATCTAGAATTCTAGTTTCTAATGATGTTTCTTGCCCTAA
TTCTAGCATTTTTCCTAATTTTACGTCAAAGAAGTTTTTATGATTTTTTACAAATTCTTTTTCA
TATACTCCTTTACTTGCCATAATTTCCAATTGATGCTTAAGAGTTTCTTCACACTCTTTCATTG
GGGGAACTTTTCCTACTTTTTTAATGTAACTGTTTATTACAATACATTTAAACATTGCCCTTA
GTTCTTCAATTGTTTCATTGTATACTGGATGGTTTCTGTTTGTCCTTTGAACATATTTACTTAA
ACCTTTATTCTTGTTAGTTTCTGCATAAATTAATGATTTGTACAGGCAACTCATCTTTAAAAC
AGTTGGTCCATCACTCTTGTCAATTATCCTCAATAGTTTTTCAGTGTAAGTTTGGTTTATATCG
CAAGATTCTATTAAGACATCTAAAGGAGGTAACTTTGAACATTTCCCTTTCATATCAGTCATC
ATTAGACAAAATGTTTCAAATGTTCCACCTAAACCACACCTTACTTTACGATCTTCTATCCTT
CCAATTTCTAATATGTAGTCTATAAATTCTTTTTCATTTTCATTACCGCTGTAATCTTTAATTA
CATTCAATGTGAATTGATGATCTACTATTGTTGTTAGATAATCTATTGCACTTCCCGTCCCGA
TGAAAGAATA
>Seq83 [organism=Silene ophiovirus] RNA2 genomic sequence
CTTGAACGTCAGATAGCCTTAAAGCTCATTCATTTGATATCTACATCTTGAATTTCACCTTAT
TTTTTAAAAAAACGAAAAAGCAACAACACACAACTGAAACAACACAAAATGCAAAATACAG
AATTGAAAATTACATAAATGAAATTACTCATCAACGCCTCTCACTACATACATCCCATTATT
ATTGTAACACTTGAATACTGCCTTCTTATTCTTCATTAAGACAATTGAGTTATTTTCTATTTTC
ATGTCATCTATCATCCCATTCTTCTTGAGAGAATTCAAAGAAAGCAGATTATGCTTTATTTCA
TCTGTGATAAATATGTCATTCATGCTGATGACATTATTACCTATGTTACAGTAAATTGGATAA
TCACAAACTTTCCCTAACACCTTCTTTCCATTACTCAATATCATTGAGTCTTTTGTTTCTTTTT
GGTTTATTCCAAATTTGACACTTTGTACATGTCTACTTGCTCCAGTGTCTAAATACCAAATGC
TATTTCCACTTGGAAAATCCAGCTCTGATATCATGTTAACACTTCCTTCTCCAATTTCAAAAG
TACTACTTATTTTTCCTTCTTCTTCGTTTTCTTTCACCATCAATCCTTCTTTCAATGATTCTAGT
CGATTGTTTTCAATTCTTTCTACCTTGTTATACCCAATTCTTCTTTGTTCACTTTCTGTTTTGAG
ACTACTTCCAATTCTTGTCAACACATTTTTCTTGTTAACATCTTTTGGAGCTTCAACATTATCC
ATATAAACGCAAATTCTTTCACTGGCTACAGTCCTTACAGGATTCATAGTCTTGTTTGTTTTG
AAGCAAATAAACATTGTTCCATAACTTCTTCCTATCACAGGAACTTCAGTTGGTTTCACAAC
AACTTTGATAAACTTAGCATCTTTAGTCTGAACTTTATAATCCATTCCAAACTCTGTATATGA
CATTCTGTGAATATCAGCTACAGTTTCTGCTTTCACTTGGTCTTTGATATTCTTCATTGATTTA
TCAACAAGTTTGAATACAATTTTTCCATTACTCTCACCGTTTACTGTAAAGAGAGGAATGTA
GAAGATTTGTATTTTATTAAATTGAATGAAAGGCTTCTTGTTGATCTTAAACAAGTCACCTAT
CTTTTCCAAATTTGATAATTCAAATCCTTCTTCATTTTCTCTTCCTTCCAATTTTATAGATATA
ACATGAACACTTGTCTTTATCTCATCAGTGGACATGTTTTGAAATTCAGAGGCCAAATCTTCA

ATTGCGTCAACATTCTCTATGCCAGGAAAAGCCATTATTCCCTTAACTCCTTCTTCACTCAAT
TCCTGAGTTGCTCGGCTGAATGAAATAGTTCCAGTTGCCTGAGATGAAGGATTTCTCAAAGT
GCCACTGCTGAGTAGAGGCTTGAGAGCAAGGTTCGACATAGCTAGATTGAGAGACGAGAGT
TCACAGGTGAAAACTTGATAAATATATGTTCAATTGAGTTGTCAACTGGCGTTAGATAATC
>Seq84 [organism=Silene ophiovirus] RNA3 genomic sequence
TTTTTTTTTTTTATTAAAACGAAAATTGAAAAACAAACAAAAGAAAGAAAGCACGTAGAAT
ATTGGAAGATGGATTTTTGAATTTTCAGTTCTTTTTCTTAAATATGTCTAACACTGCCTCCTTA
GAATACTTCATGAAATCAACCTCCTTATTATCTAATATAGGCCACTGTCTAACACCAACCGC
ATTGATAGTACCCACAATATTCGCGTCTTGAGCAAAAACCCCCAGCTTCTTAGTAATGATGA
TATCCACTAAGTTTTCCCTTCCCTTACTATCAAGCTGAAACATGATTGCTTTAATGGCCGAAA
GAACAAATCCTGGAGGAACATGCCTCTTCTTATTCAAAGGGTGAAGGTTTAATTGACCTTCT
ATATTTGCAGCTTGTTCCACTAAACAATCTGATATTTCTTTACTCTTTGCTAATATCAAATTGT
ATTCTATTCCTTTTACCATTTCTTCAGGTTTCATTTTTGTGATATCCCGGAGTTTTTCTTTGGA
ATGACCAGCAGCAAGGCATGCTAGCCTTACAAATTTGTTTCCAGCAATTGATAATTTGCATC
TACCTCCTACTTCTGCAGGTAATGATTCCAATTTCATTTTAAGTAATGCTTCTCCAGGAAATT
TTCTTAGATTTGATCTAGATAATAATTTTGCTAGATCACTTTCTGTATCAATTTTTAATTCTCT
GAATGAAAAATTTTCCAATACAAATTTCTGCAACGGCTTTTTAGATTCAATGGTTGGTAATCC
TCCTCTATTATAAACCATACAGATAGCAGCACAAAATCCACTCTTCCCAATTATAAGATTGT
CTTGTATCTCATTTTTTGGTATAGATTCTACCGTAACAACTTCAAAAATATCATTTATTTTTC
ATCATTATGAAATGCTTCATATAATGCAGCATCCAAAACACAAGTCCCTGCTGCAATGAGGA
ATTCCATTTTCACTTCTGCACCAACAAAAGTGGCAACTTTCACCTCTTTTGGGACATACCCTG
CCTCAATTTTACACTGAGGAGCGAAATCATTTAGTACACTGATTAATTTCTCAGGGCTAATTG
ATATAGATTCAATGTTAACCATCTGAGAGAACGATTCTATAGTGTTGATAACCTCAGAATGG
CCAGATGGAAAATCTCGCATACTGGAAAAACCTAATTTATTTTCCTCTGACGGGGGTACCTG
GTCTGCCGGCCGGATCCCCTTTGCCGTAAATTCTTTACCCAAGTCTTCAGCATCAATTTCCAA
GTTAGAATCCAGCCCCGCTAGAACTGTCACCTTGTTATCATCAATTGTATCGCTGTCTTTTAA
CATGTTAAAATCAGCTAAAACTTTTGCCATTGAAGCTGATGCATTCTTAGCAGAAAACTTGA
AGCTGTAGTCTATAAGGTCAGCAACTTTTACCTTGGGAGACGACATTTCAGCTTTAGTAGCG
TGCTTGATTGTAAGTTGTACTTTGCAAGTTTTTAATCTTAAATTGTTCAAGTAGATGAGCTAG
AGGTAGTTGTTTTTAAACTATCCCGC
>Seq85 [organism=Thymus ophiovirus] RNA2 genomic sequence
CGATAAACAAGGAATACATATGCTTCTGGACTTAAAATCCTTCTTTTAGTGATAATCTAGTC
AAGCTCCCACTTGATTTCAGACATCAAGTGCCTTGTCCAGAGACTGTGCATTTCTTACTGCAT
GTTCAATAACTGAACAACCACCATGTTCTTCACATGCAGCATATGATTCATCTTTGAGCCAC
ATTCTTCCTTCATTGCTTGCATCAAATCTCATTTTTATTACATCATCTTTCATCAAAAAGACTT
CATCCCCACTTGAGACCATTGCATCTACTATCTTGTCCCCTCTCAATTTTGAAAAAGACAATA
TGTTACATCCAAACCTAAGATCAGAGAAAAAGCTTACTTCACTGAACAGGATCAGATCATCA
CCAAGGCTAAAAAACATGGCTCCTTCTTCAATGTCCATTTTGTCGACACCGCCAAAACCCTC
AATATCGGTAGTAGGGTTAATTTTAGGCCTATAGTAAAAATGTTCTCCAGATCCAGTATCCA
ACATAGTTGAGAGCAGAGGATGCTTATATTGTTTATAAACATTCTTCATGGCTTTTGTTTTGG

GGGTTGCCAGCTGCGAGTTCTCAGCCTTACTGTCAGAAATATTCATCTCATTTAGGTACCTTC
CTAACATGTCAACACTCTTCCTTCCAGCTTCTATGAATGTCTCTTTAGAGTCAGTAGATGAAC
TACTGAAGCTTTTCCTGCTCTCAGTTTCCATTATGACATTTTTCATTTTCTTCTTTCCATCTCTT
TTTCCTTCTCTCACTGATTTAAGTTGTTCTTCTCTTTTTCTCTTGACCTCATCCGCAACAATTTC
ACTCACATTGCTTAAAACACTTCTTTTGTTAAGGTCTTTTGGAGCCTTACCACCATCAATATA
AATCAGAGTGCTTGGCTTGGGTTCGTAACTGAATGGAATCATTTCTTCATGGACAAAAAATG
CCACAAATAGTGAACCATAAGCCCTTCCAACAACAGGGGCTACACTTGCACAGATTTCTATC
GAAATTTCTGCAACATCCTTTTTTTCAACAAAATAGTTCATGGATAATTCCGCCAAAGCCATC
TGGTCCAATCTGATGTCCCTCTTCTGAATCAATCCGTCATATCCAGCCTTTTCCTTCCCATGAT
CTATCAAACTGACACTTAAAGTTTCATTCTCAAAATTCTTCTTATCTGAACCAAGCAATGGAA
GATAGAGCACTTGCACTTTCTCAAACCTGATAAAAGGGTATTTTTTCTTCTTCAAGTCTGGAA
TGAACCTTTTGATTTTGTCCAAAGTTGTTAATTGCAATTTTTTATTGTGCTCAGTTTTCTTGAG
ACTCACAGTGATGGGGGCAATTACAGACGTGATTATTCCCTTTTGTATATTTCCAAAATCAG
CAACAATGTCTTCTATTCCTTCAGGCAAATCACCATCTTGAGACAATGACACAACTGTTGTGT
CCGATAGCTTCAGTATCTCATCCTTGACTTGTTTGGCTGACATTCTCAATGATGAGCTTGAAC
TCTCTCTCATTGAACCTGAGTTGTTGAATTTTCTCAGTGAGCGGTTTCCGGATGTTGACATCT
TAAAAACAAGTTACGATTGATCAC
>Seq86 [organism=Thymus ophiovirus] RNA3 genomic sequence
ACTGTTTCAAAGAATTTATTTAAAACGAGTGAACGGAAAATAATATCATCGAGACTAATCAA
GAGCAGAGACAATTCAATGCTTTGAGTATGCAGAATAGACAGACTGTGAACTGAGTTCATTG
AAATCAGCATCTGGGTTCTCCAAGATATGCCATATCAAGGCACCTTCCTCTTCACTACCAAC
AAAGTTTGTATCACGGAGAAACGATGAATTGTTGTCTTTCTTTGCCTTTCTGTTGATTTCAGG
TCTCGCAGATGGAGCAACAGACTCAATGATTGCTCTAGTTAACTTCAGAGTGAAGTTCTGAA
TTACTGCTCTAGATTGAGAGAGAGGGTGCATGTTCAGTTGGGTTTTATAATCAGTTGCAATT
GTCCTCAGAGCTTTAACTATCTCTTTCGCTTTTGTTATTCTTCTGTTGTGCTCTTCATACTGTTT
GTAAAGCTCAATTCCTGCTCCGGCTGGTATTGCTCTTTCTTCTGCTTCCTTCAGGGATGCTGT
AAAGATAGAGTAACGAATAGCTCGATTTCCTGCTATAGACATCTTACATCTGGTTGAAATTG
CTATTGGAGTCCTGTCTAGGTCAATCTCAAGGAAAATCTTTGAAGGAAATTTTTCAGTATTTG
TGCTCGAGAGAAGTTCAGCGATAGCCTTTAAGCTACCTATTTCACCTTTAAATATCTTTTCCT
TGATGAATTTAGGAATTGGCTTTGTGTCATCAGACTTTGAAGGAAGTGAACCTTGAATAAAC
ACAAGGACAACTGCCGCTAACAATGCTCTCTTTGCATTTATTATGTTGTCACTAACAGAAGA
TGCAGTCAGTTTTGTGACTATCCTAGTAGTGAAAAGCCCAGCATTTTCTTCTCTTGTCCCTAT
TGCTTCATAAAGAAGAGCATCTAGCACTTTAGTCCCTGCGGCAATTAAGTTGTTCACATCCTT
TTTGTCACTGTAAATGTTTATTTTAATGTCCCCAGTCCGGTAAGCATCATCTTCCCATTTTATT
CCATAGAGGAATCTTGTCAAGTGTTCCTTGATTTCTTCAGCAGACACAGTATAAGACTGTTGT
TTCACTAAATTCTCTACTTCTTCAGTAGATAAAACCTTTCCTGTGTTCAAAACTGCATGTCCA
GGGTTAGGCTTGTCATGCTCCTCATCAGTGTATTCTTCTCCTTCTCCTTCCTCCACAGCACTGG
AAAAACTTGAACCCTTGTGGTGTTTCTCCTTTCCTGAAATGTAATCCTCAACTGTTTTGCTTTT
CGATGAAACCACATGGGAAACAACCAATTTTTTATTGATCATTATAAGATCAGCATTGGGTT
TTTCACTTTCATGTTTAGAAAGAGATATGAGAAATGCATTTTTCTCTTCATTGTTGTCCAGAT

TGACACCAAATTCTTTAAAAAACTTGTCAATAGCTTTTATCTCTCTGGGGTTACTGCTTACTT
CTCTCTTATCTGCGAGCTCTCTCAGCACGACCACAGGGACATCATTAGATTTAAGTGAAGTC
ATCTTGTGTTATTTGTTTAAAGTTCAAATTTGCAAATTGTTACTCGTTTAAAATAAATCCCCC
TCAACTGTTTTGCTTTTCG
>Seq87 [organism=Tulip mild mottle mosaic virus_chilo] RNA3 partial sequence
CCACCTTCTCACTACGCGATCTTCATCATTTCTTACCGTAAGCAGTCTTCACGGCATCGACTG
AAAGTTCAGAAAAGTCAGCTTCTGAATTCGTCAGAGTAGGAAAGACCGGATTACCTACAGC
ATTTAGCTTTCCAAATATATTCTCGTCTCTCTTGAAGGCTTCTATCTTCTTGTCTTCGATTGCT
TTTCTCATTTCTAGCCTTCCCTTGTAAGACAAAGATGTGACAATAGCAGAGGTCAGTTGCAG
AGTGAAATTCTTTCTTGATGATCTTTCAGGGGATAGTGGATGCATCCTCACTTGTGCTTGGAA
ATCAGAAGCTAAGGAACACAAAACATCAACAATTTTCTTAGCTTTCTCCAATTTCTCCTTTTT
CTGCATGTACTCAGCTCCTGCAGTTGCTGTCATCGGATCGGGAACGGGAACTTCATCCTTTTC
AAACTTTTGAGCTAATAGAGCATATCTTATTGCTTTGTTTCCAGCGATAGACATTTTACAACG
CGATGAGACTTCAGTTGGAAGGTTCTCAAGCGCAATTTTCAAAAAGACCTGACTTGGAAACA
AAGAAGGGTCTGCAGATGACAAGAGTGCACATAGATCATTTGACTTGAGATCCTTCTCTCTG
AAAATCGTCTCCCTTATGAATTTTGAAAGGGGTCTTTCTTCGTTTGTTTTTGAAGGTAATCCT
CCTTGATTATAAACAAGACAAAAAGCTGCTTTTATGGCTCGATTTCCTGTTTCGATTTTGGAG
ACTAAATCCTTGCTATCAACAAATTCAGGTGACAATGCTTTCACATCAAAGATGAAATTGTG
CTCGTTCGAGTCCTTATACGTACTGTATAGAATGGCGTCCAAAATTTTGGTCCCTGCAGAGA
GTAAACTGCTAACGCTGTACTCAACCCCCTTGAAGTACTTAATTTCGATTTCTTTTGGCTTAT
ATGTCTCAGCTGTCCGTGGTAGATTTTTCACGTAATCTTCCAAAACTTTTTGGATTTTGTCCTG
ATCGAGCGTGAGACTTTTACCTTCGATCAGCTCTTTTACTTTGGCTGCACTAAAAGCAATCTT
TTCACCGATTTCAACTTTTTTCTCTGCTTCCTTGCCCTTAGAGCTAGGTTCACCAGCCTTCTTG
GAAACTTCAAAAGACAGGGAACCGTCCTCGAGGACAAAAACGGTAGCGTTTGGATCTGCGG
ATGTGAACAGGGCCATCTTTGCAGGAACAGCCTTCTCATCCTTTGTCATGAGACCAAGAGAA
ATAAGAATCTTTGTCTCATCTTCAGAAGTTACGTCCTTTTGCAAGATATTCTGGATCTCAG
>Seq88 [organism=Triticum associated ophiovirus] RNA1 partial sequence
TCCAGGTTGTGATCCCTAACAGCTCAAATAACACTTCCTTGCTTCGTCCAAATTTGACACCTT
TCCAAGCACTTCTTATTAATTCATTGACATTGTCAAGGTGTAAATCACTTCCATTTATGTAAC
TGTTGACTTTGGAGTAGACCAAATTTTTGATAATGTATTCTGTATCCACTTCTTCTAATTGTA
GTTCTTCTGCGATCTGCAAAATTAACATCCAGCTCCACACAAGCTTCTTATTGGACATTCTAG
AATGTAAGTTCAGTACATATTGCAATAAGTCATTGAATGGTCCTTTGTACCCAGTAATCACA
ATATTTCTCGTCACTTGGTTACTGCCCACTTCTATCAAATCTTTCCCGGCAATGTAATCATCA
TATATTTCATACATTGGTCTATCGACAGATTCTGCCCCTGTTATTAATCGCATCGAGTCAGTT
ATGCACTGAGATGGGTGATCATAGGAATAATGTAATAATTGTCTTCTTAAATCACGTTCACT
CTTGATATACTGGTTGAGCATTGTTGGGATCTCAAATCTTTTCTGGACTGTTGCCTTATCAAC
AATTTCAAGGATTGACTCTTTGCTGATCATTTCTTCACTTGGATCTCTGATGAATTGTGGATT
GCTGAATTCTATATCTTTAGATAAACCATCTTGACATGTGCTGATTATTTTCCGACTGAATAC
TGAACTAATGAAGCCTTTTTCAGTGCTTATTTCTCCAATTTTTGTACGCTTAGTTGGTTTGAAC
CATAAGTAAATGAGGCCTATTGAATTAGAAGATGGAACCAGTGCATCACAAGTGAACTTTTT

CAACACAGTTAGGACCTCGTCTGGCATCTGATCAGCTACTGAGTTTAATCTTATGATGCATTC
TTTTCCGTTTTCTAAAATGTTGCTAGCCGCTTGCCACACATTCTTCCCTGTTTTGTGGCTCACA
TCGAAGAGAATTATGTCATGATTGTAATACTTTTCATACTCCATGCTTTGGAATGCATCAAAT
GTTTTCTTTTTTGCTATCATTCCTGGTGCACGCTCAAGCAAGTTGAATCCGTCATTCCTTGATA
ATGATGTGTGTTCTAATTGGTACTCTTGTAAAGCTAAGTGGAATTCTCCTCTCCCCCCGCACA
TGTCAGCAATTCGCTTATCTAAGGAAGATAGTTGCAGTAGCTCACATACTCCGAGTGCAGAT
GAATATGTTAGAGACCGTGTTGGTGATGAGAAGATTTCGATGTCTGCGCTTAGTCTGCATAC
AGTTTCAAGAAGCCGTATTGCTGCTCTAACACCAACTACATCTTGTAGACATTCCACATCAA
ATGGGACTGTATCATAATTTACTTTCTTCATTGATTCACCCATCATTGGTTTCATAAAGCCGC
AGTGTTGTGGGGTATAATAAGAATCAATTTTCCTTTGTTCAAAGCTCATTTTATTGTATTTGT
GACAAAAGAAATCTATCGCGTCTTCTATGTCCTTATAACTAGCACCGAGGGCTGGGAAACCA
TTTACTAATGTGTCTCCAATGGCCTGAGATCTGATGTATATGTGACCTTTTAGCTCTCTCATT
GATTTAAGAACAGTCTTTCCTTTGTGCTTGCTAGCTTTCTCTTTTATGAGTAATTTCAGGCTGC
TGTCTTCGTTTTGGACAACTTCGTAAACCAAAGAAGTTGCCAAGACGAATAGCTTCATCATA
GCATCCCGATATTCCAGTGAATAACCATGCCTTTCAATTTCTTGAATCCAATTCTTTTGATCG
CCTAGTCGGAATACTTCTTGGTATATGATGGATCTGATAATAGCCTGATTTGGAGTTTCTGTT
CCAATGATGTCATAGCCCTTATAGAAGTCGTAGAACTTATCCAAGAATTCAATGTGATTCAT
GTCACCGATCTCATACAGTGTCATAATATCCTTGATGAATGATGGAGCAATTCTATTTGGTGT
CATCAACCTTTGATCTTCCATGTATGAGAATAGATGGCGAAATGCTCCTTCTTCCTTCATTCT
ATATGTGCTTTTTGTCTCAACATCTTCAATGAAATCTTCTGAATTACTTGGAATAGCGGCAAG
GAATGATGAGTATTGTCTGTATATGCGCTTACCAGTTTCAAACAAGTTTTTGGGCACAAATG
TTTGCATTTGCATCTTGCTAACTGAAACTAAGTTCTTTAATTCAAAACTCACATCTTGTAGTA
GTGGTTCTAATTCTGGGATGAATGAATATCCATAGCATTCATGCTTATCTGCATTTAAGAGTG
ATAACTGTATTCTCATGCAGACATTTAAGTACTGTAAATTTAGGTTGTTATCTTCTCCTCCAT
ATTCTGACAATAGATGATCAAAGTTTGTGACTGTGTACAACTTTGATTTGTCTTGGGATGAG
AGATCTCCGCTGAATGCTTTGAAACCACCACCATCGGCACGATGGAATATTCTACCACCTGA
TGGCACTGCAATTATATCTCTTAAGTCATTGAGCCGAATATCAGTGAAGGTGCTAAGTGTTA
AGTCACATGCATCCGTTAGCCCAGTTGCAGTTGTTTTATCTTCCATTCCACTTCTGAATTGCT
CTGATCCCATCCACACTGTAAATCGACACACATCATAAGCAAGAAAAATGAGTTTGTGAGCA
AACATTCCCTCAATCCCTATTGTTCTGTCATATTTTGGTCTTGCGCCACTGGAATACAGGGGT
GGCCTAAAGTGTTCACCATCTCTAGTTAAGACTTTTTCAGTGAGTCTTGACAATTGAAATCTC
ACACTAAACGGGTTCCTCACAATGCATCCAAGAATTGGGATCTCTTCAATCTCAATGAACTT
GATCCCAGTGATTTGGCGTCGCATGTCTTGCATCTTGGCAAGTGATAAGTGACCAAGCTCAG
GGCGCATTAATGGCAGAATTGGTCGATGGTTGGTTTTCCATGCTTTTGAGTGAATGATGTTTA
GTTGTCGGTTACTGAGTAGATGTCGAAGAGTCACAGAATTATCGATTTTTGACACCCATGAT
CTGGCGTAACTGAAAATGCTACATTCATAATATTTTGATGTCCAGCGGTAGTGGAATGCNNN
NNNNNNNNNNNNNAGTAGTTTTTGCTTGTATTCCAATTCTTGTGGTGATCCAATCATTTTTAT
TGCTCTCAGCAGTTCTTCGTTTTTCACTTTATAAGGGAGAATTTTGATTAGTTCATCAGAGAG
GAGTGTGCTAGTGTCGAAGTAACCTCCTGTTAGCGGAAATGGGCTCATTAAATATGACTCTA
GGCTTGTCGAGGGTTGATATAAGCTATGGATTATGTAATGGTATTGTTTTGGGATTAAAGAG

TTCGCATAGTGTATCCGTTTCACATTGGAGACTGAGAAACCAGAAATCGCAGCATCTGGGAN
NNNNNNNNNNNNNNNNNNGTGCTCTTCCGATCTCATGAGTACTAAGGTCAGTCCAAGTACT
AGTTCGTTATCCTTGTGAAAATGCATGAATGATGCATTTAAAGATTCTGCATCTGGCAACTTT
TCCCATTTGTTTGGATTATACCCCATTGATATGATTTGGCCCAACTCACCCAAGTTGAGTTCC
TGTAACTGATTCTTTACTGTGTTCTGATGTATGATGAATCTGGGATCTATCTCATTTGTGATTT
GTAGGATTTTGTATCTAATGCTTTCAATGTGTAATGACAATAGTTGATAGTTCCTAATACAAG
TCTGCAACAAGTGATCATCTGATCCTTCGCTAGCTGATGTGTAGCATGAATCAGCAGTAGTA
ACGTCTGATAATTCTCCGCAGATTAGCCCATCAGTACTACTAGTCATTGACAGAATTCTTTTG
GCACTAGTGTCTATTAATTTACCCCGCCAGTAATGGTTTTTAACATTGTTGCCCGATTTTCT
GATACTTGAGTCTGTGAGAGCTTCAATATTAGTCCCATTTTTAATGCTGATGATTGGCAATTC
TCAACTATATTATCAAGTTCTTCTACTTGTCGATTGGGAATCCTCATGGTTGCACATACATCA
TCTGAGTAACTTGTTGCAATAGCTCTATATATCCCTTGATCAACAAGCATTTGTTCAATCATT
AGTTGTGATTGGATTCCCCATAGTGGACCAAACCATCCTTCAATTGCGCCGATTTGGAATGC
TGATAAGTATACAAAATCAGACCATAAGGTCTCTGAGAATATCGGACATGAGTGAAACACC
CAGGGCAATTTTAAGAAATCATCATTATCAGAACCTTCTAGTCCGAACATGTCACCTATTTCT
TCCATGAGAAATGATGTATTCTCTGGCCTTTGTGTGGTGTTGTGGCCTGAGTAGTCAAGAAA
TAAAGCAATTCCATGTGGATGATCTTGCATTTCACACATTTTTGTCATCATGTTTAATCTAGA
GTTTGAATCCATAGTCATCATTTGTCCAGGTATTAATTTGGCTGCCCTTTTGACCATTTCCAT
GGCTACTGATAACCTCAGCTTTAATGCAAAGCTTGCTATTCCGTAGAATCTTGGATCACTCTT
CATTTCTTTCTCTTTTTCCGCCAGCCTAATCCACAGTTTTGTAGCCTTCATTTCGGGGATGTCA
TCTCTTCGGAATACATTGACGAATCGATCTGGGTATTCCTCTATGAATGACTTGAAGTCCACC
TCAATATCAATCTCTTGGCTTTCAATGAACACAACCAGTTCCTTGATAGAGTTATGACCATTC
GGATCATAGTCATCAACAGTGCAGAGATCGGAAGAGNNNNNNNNNNNNCTTAAACAAGCTT
CTTTGTTAAAATTCAACATTTTTCCTAATTTCACTGTTGAGTAGTTGGTGGGGTTATATACTA
TACTTTTATCATATTTCTGTTTGTTTGCAAAGAGTTCTAAGTGAAACATTAGCTCGTCATTTG
CTTTGATGGGGGGTAATTTCTTATTTTTTGTTATATATGATGTCACTACGTGCATTTTGAATCT
CATCCTCAATTTTTCTATTTGTAGTTTATCTATTGGGAGGTCCCTTTCTGTCCTTTGCGTGTAT
CGAATCAATCCACCCTCGTGGCTAACAGCTGCAAAATGTGCATTTTTGCATATACCAGTCAG
CCAAATGCAGTTTTCTGCGCTAGTAATGTTTAAAAGTTGGATCGTTTGTTCACCCATGTCATT
GTCTATTTGATACATTTCTAGTAGAGCATCGAGGTATGGCATGTGGGCACATAATCCCTTCAT
GTCTGCTGCCATCAATGTATATGTCTCTAGACTAGCTAAAAACTTCACTTTTTTGTTATGATT
CCAGTTTGCCTTTTTGATTGATAGCACAAGTTCAATTAAGTTTCGTTGTTCAGCCCCACAATT
ATCACGGATTATGTGAAGGTTGCACATGATTTCTGAATAACTAAA
>Seq89 [organism=Triticum associated ophiovirus] RNA3 partial sequence
CGATCTTTATACCTCTGGTTATTTTTCTTAACACGAGACTGTTGACCACTTGATCGCACTACT
TGGTGTAATTCGTAATCCATTTCACAGTGAGTTCCTCTCCACGTAATTCAGGAGATTGGAGA
CCAGGGCAGGATAGGACTTGGCCAACCTCTTGATACAACATGGTATCCAACTTTAATGCACG
CATATTTTTGTCACGGATGAATGCCAGTGCTGCAGTCAGTTCCGTGTTGTCAAGGGAGCGGA
ATGCCATGACTGACAACTTCTGGCTGAGTTTCGCAGCGTGTCCATTGGTGAGGGGGAAGTCT
GGGTGAAACTTGCGCAACGTATCCCACTTGAGAGATAACACATAAGCTCGGATCTCACGAG

ATAGTGTAGCGACTGGAGTTTTGTTCTTGCCACCGTCGTGAGCCGCAGCAATCTGAAAGTAT
CGAACAAATTTCGTTCCTGCTGGGCTCAGTTCGATTCGGTTAGCTGTAACCTTGTCTAAATTG
TTGAGGTCGTCCAATAGGAATGTGGTCATTGGGAATTTACTGAGAGGTGCACTGCTGAGAAT
GCTGGCAAGCTGTGCCTCTGTCTTGACATTCAGGTTCGCCATGGTGCGGTTAACTATGTTTGC
ACTTAGGGGGCTAGATGTAGCATCACGTTTTGGGGCAAAACCTCTCGTCAAGTAAAATTCAC
AGAGTGCAAATATTGACTTGCCGGCCGTATTGCGGATGCTACCAGCTGTTCCACTATCTGTG
CTAAATGGACCACTGATTTTGAAGATCCGTTCAAAGTTGGGATCTTGACCGTGAGTAAAGAA
GATTAGTGCATCAATGACTCCAGTTCCAGCAGCACAAACATTAGTCAGGAGTTCCTCTGCAT
CCCAATCCATCATATCTTGGAACATGAAGATCTTGATTTCGCCGGCGAAGTAAGTGTCTTGC
TTTGGAATTGCTCCAAGAAGCTTATGGATGCAGGCTTTCAACAGATCACCGTCAAGCACTAG
GCCAGTCTTTCGGAGGTCTTCATAAGCCTGTACACCGGCTGCATCTCCAATCGGCACAACGG
CTCCAGTATTCAACATCACTGGTGCGGCCTGAGTAACAGCTGGCGCAGAAGCAAGATTCTGC
AGGGACAGGAAAGGGGAAGCCATGTTAATCGCTGAGAGATTGTCAGGAATTAGTCTTGAGA
ATAATTGCTCACG
>Seq90 [organism=Viola ophiovirus] RNA1 genomic sequence
TAGGGTCTTCACTTTCAAACAATTCTAATTAGAATTAACTAGAAGATTTGCCGAGTTTTTCAC
CTCCGCTTGGAACACAACAATGATTATAGGTCACAGCTGCATTCCTGTTTAATTAAAAATCC
AAATTAAAATATTTAATATTCATCGTATCTGAATAGGTTAGGGTCTTCACTTTCAAACAATTC
TAATTCTGCAAGTGTTTCTTCCAAAGTTTTTCTTTCTCCACATTTTTAATTTCATCATTTAGTT
GTAAAAATTTACTCATAATATCTGTTATTCTATCTTCTTCTTTTTGTAAATTATTTTCTTCTTCT
TCTTTAAAACTATCAATAAATGATTGATTGAACAAATCATTTAATGGATTTGATTTAAATTCA
TCCGAGTTCATATATTCTATAGCATCCTTATATTTTAATTCAAGATTTTTCAATTCTTCTTCTG
ATCTGATTCTATCCTCTTTTGAAGCTACTTCACCCCTTAGTTTTAAAATAAAATCGATAGTTT
CCAATTTTCCTGATTCTGTTTTATTACTTATCCTATAATCTTTGGCAAGTCTCATTACTCTATT
TAATATGAGTTTAAAATCCTTTATATTGTTTGTGTTGGTTCTTGTTTTTACTTTTAACAAACCG
GATAAGTTGTTTGTGTGAACACCAGAAATAATTCGCATTCCTAATTTTATTGATTTCTCTTC
CATAATTCAGTAACTAATTTAGTAGTTGTAAAAAGATTTGTCACTAATTTCCCTGTGCTATTT
TTCTTTATAGCTTCTATGCTTACTGAATCAATTAAGTCCTTGGAGCTTATCCCTCTTTCATCAT
TCATCAATGTGTAAAGTAAGGACCATGCTGATTTATGTCTCATAGGTATGTTCATTATGGAA
ACCTCTTGCAAAAAATTCAGCTAGTTCACTTTGTTTTGACTCCTTAAATCCATAGACTATATAA
GGAGGGAAGGATGACAACTTTTCACAATGTACTTCAAATTCAGTGCTACCTAATTCTTTATA
TTCCTTTATTATTTCTGAAAAATTCTTATGTCCTGATTCTCCCTTTGAAAGATATGTCAAGTTA
TCATATAAAAGGTATTTATCTGGTTTTTTCCAAGTTCCCACACATAGTAAATTTTCTAAGTCA
ACTTTATTATTAACACTTCTTTTTAAATCATCAGGGACAATTATTATTTCGTCTTTCATTGGTG
ATAACATCTCATTTAAAATATGGTTATCAGTTATGAATTCTTCCGTATGGTCTACAATGTTTA
ATCCAAATCTAGGCATAACTGGTTTGGACATTAATTCTAATGAATTTTTAACTAATTGTCGCA
ATAATAGTGAATTATTAAACCCACCTTTTTCAATATTCTCTGAAAGAAATGGTCCATCTAAAC
TAATCATATCATTTTCATTTCTTTCTAGAATAAGATAAATTATACCAAGTGAATTATAGTCTG
GCACTGCAATTTTTGATTTACATTTTTTAACCAGTTCCCAGAAGCTTCTTGGTGCATTACCGA
TTATGCTATTGATTCTTAGTATTACAACTTTATCATTGTTCAATAGATCAGTTATTGAATTTGT

TAATTTTGATTTATAGTCAGTTGCATGGGAGATATCTATTAAGAAAATGTTATGAAAATTTAT
TGAATTATAATTTTCCTTATCGAAGCAATTCCAATTTGCTTTCTTAGTTACTAATCCAGGCAT
TCTATTAATCATATTGAAACCATCAAGTCTAGAATATGATTTGTGGATTAAATTTGCTTCTTC
CAATGCTAAGTGAAAGTCACCTCTTCCCCCGCACACATCAGCAATATTATCATCCATGCTTA
AAATCCCTTCTTCTATAAGGGTCCTTATTAGTCCTTTCGCGGTTGGATATATATCTGATTTTGT
TGGTGAGGAAATTACAGATACATCACAAGCTATTGATGTAGCATATTCATAAGACCTTATAG
CTGATCTTAGTCCTTTTATGTCAGAAATATCTGATTCTTTCAAATCAAGTATTTTATAATTAA
CCTCTCCAAAGAATTTGTTTTCTTGTTTTTTAATCTCTTTTGGAATTTCATCTGTAAAAAACTC
ATAAAATCTTGCTTGTTCAAATTCCCTATGGTTTAATTCTTTGATCATATCTTTTGCACAGCAT
TCTATTTCTACAATTTTATAAGACATTAAAGGAAAACCATAAATTAATGTTCTTTGTATTGAT
TTGTCCTTAATATGAAAGTGATAATCTCCAGATTTCATTTTAGATAATGCTCTTTTTGAATTTT
CAATTGTTTTGTCAGAATTTATTATGAGTTTAAAGTTATTAGCCCCTGTTGTTTCAACTGAAT
ATACTAATCCACAAGCTATAATGAATAATCTTAATAAGTTGTTTCTGTACGTGAATGAAAAA
CCAGTTTTAACCATTTCTGCCATCCAATTTTCCCCAATTTCATCTTTTTTGAAGATTTCTTCAT
ACAACAATCCTCTTATTATAGCTCCATAATTACTTTCCTTCCCAATGACGTTAAGAGAAGAGT
AATAATTATAAAATTGATTAAAGAAATCATCCTTATCATAAAATGTTCTTTCTGAATTATCTA
TGATTTGAAATAGTAATTCATCGGCTATTATTTCTGGAGTTGTTATAAGCTGGTCCATCATAT
ATTTATAAACTATTTTAAAACTTGTCTCTGCTTTGATATCACTTAGTTTAAATCCTTCAGATAT
AGCAACAAATTGATCAGGGTCTTCGTCATCACAACCAATAAACTCTGAATATGTTTTATAGA
TTTTACCTGATTCCATAAAATATTTTTTAGATAATAAATTATTGGGTGTTATTTCTTCTTCTAA
TTTTTGGATTCCAGATAAATTAAAAGTTACATCTTTTATGTGATTAACTATTGTATCAGAGAG
AGTATAACTAGCTAAGGATTTTCTTGAAGTGTTCAATAAGCTTAGGATTATCCTCGTTGAGGT
AATTAAATACTGTATATTTAAATTATTATCTTCTCCACCTAAGAGATACTGCATTGTACTAAT
TCCTAGTATTTCATATTTTGAAGATACAAGATGGCTACTTAAATCACCTGTCTTAGGATTAAA
ATTACTTGAGTTTGCTCTATGGAAAAATCTTCCACCAATAGGAGGAGATATAAATTTTTCTA
AGTCTTCATATTTTGAATTAGAAAATGTTGATAATGTAAAATTACAAACCCTTATTAAACAG
AATTCTTCTCTTATTGTTATTTCAGAAAATTTTTTAATTTCCATTATTATCCATTTTGTAAATC
TTGTTATTTCTAATCCTTGAAATATTAATTTATGTTTGAATAATCCTGATATTCCACTATCTGA
ATCATATTTTGGCAAATTTAAAGACGTTCTAACAGGTGGAAGTCCCTTAACTCCTCTTTCTGT
AAGTATTTTATTTCCTCTAGTAGTTATTTCGAACATTTTATTCCAGGTAGTAGTTTCAATGTTC
CCCATAATTGGTACTTCTTCTATGTTTAGAAAATTCATAGATAATTTTTCACCTATTTCTCTTC
TTTCATCCATAAGACCATCAAAATTAAATAGATTCCTTTCTCTTTTGTTTTTTAAATTAAGTTT
GTTATTCTTTATAATTATTTTATGGTTAGCAGTCCAAGCTTTATTCCATAATTTGATGAATCTG
TACCCCATCATACATATAAAGGTGGTTGAATTATCTATTTTCTTTATAAATGTGTTAATATAC
CCATAAACTGATTCTTCATAATATTTAGATACTATTCTCGAAGAATAACACTCTAAAAATGG
TTCCATTATGTTTTTCTTGAATTTATTTTCCTTTTTGCTATCAACTAAATTAAAGATTTGTTTC
ATTTCTATATTTTTAATTCTTTTTGGAAGTACCCTTTTTAGTGAATCAGAAAGGAGTGTTGAG
GTATCATACCTTCCTCCTGTCATGGGAAATGGAGTACTTAGATAGCTTAATCTTTCCGTAGAT
AAATTAATTAATTTAGTTAATGAATATTGATCATTTTCATCTAATAACATTCTTAAGTAAGAT
AATCTTTTAACACTTGATTGAGAATATCCTGAAATTAAGGCATCTGGAATTGGGGTTAATGA

ATATCCATAACATGTATAAGGGGCATAAAAGAAATAAAGAATGTGGCTTAAAAAACTTTTA
TTTTTCAGGTTGAATGTTATAATTGTTGTTTCGGGATGTAAATCAGGTGAATTTGGAACATTC
TCTCTCTTTAATATTTCTTTGGCATTTAACCTTGAAAATTTCTTAACGTTATCATCTTTGTGTT
TTCTAAAAGTAATAGATCTATTATCTTCTGAATAATCTGTCTGTGAAATTAAATGAAGCAAA
TCTTTAATAATAACTTTTATTATCCTAAAATTTCTAATTACTGATTGCAAAAGATTTGAAGAT
GCTTTTTCTGAAGAAGACGTAAAACCAGAATCAGCTAATTTACATAACTCTAAATCGTCACC
AAATAGATAGGATGAATTTGTTGATATGCCAGTCATCTTTTTTAGATTGTGTTCTATAGGCAT
GTCGTTATAATAATGATTTTTAAGCATACTACTTCTAGATTTACTTATTTGAGTTTGACTCAG
TTTTACTATTAAACCCATTTTTAAACCAGATGTTTGTACCCTTTCAATCATAGCATTAAGAAC
TTTGGTTGATACATTTTTATATTTGAAAACAGCAAATGCATCATCTGAATAAGAAGTAGCTA
TATATTCACTTACCTTAAAATTCATGAACATGGATTCTAACATCAATTGTGACTGTATTCCCC
ATAATGGGCCTAACCAACCTTCTATTGCTCCCTTTTGTCCCTTTGAATAATAGACATAATCTG
AATACATTGACTCTCCAACTATATCAAGATTTTCAAAAACATTTAATATTGAACAAAACTTTT
TCAACTCTTCAGATTCTTGCTCGAATCCATATAAAGCCCCTATTTCTTCCATTATAAATAAAT
TATTTTCTCTTTTTTGAGATGTATTATGACCAGAATAATCTAAAAATAAAGAATAACTTCCTT
CATCTGTTAATAGACTAGTCATGTCATACATCTTAGTTCTTCTATCGTCTTCGTTCATAGTCAT
CATTTGTCCAGGAATAAGCTTCATTGCTCTTTTTACCATTTCCATAATTGAAGATATCCATAA
TTTTAAATGAAAAGACGCAACACCATAAAATCTTCCAGCACTTTTTTGTTCTTTTTCTTTTTCT
ATTATCCTGGTTAATGTATGTAATGTTTCTATTGTTAATTCATTCCTTTTTAAACACTTTATTA
TTCTTTCCCTTTCAACTTCTTCAAAATTGAATAAATCATTAGGGTCACTTATTTTCTCATGCTC
TATATAATATAAAAGTTCCTTAATTGAATTCATCCCACTTTGATTATAATTGTCAGTAGAACA
AGACTTATCAATTAGTCTGCTTTGAAGGTTGATTTCTTCTCCAACTTCCAAAGTTTTGCCAAA
AAGTATATCCTTGTAATTATTTGGATTATTTACAAACCTTTCATTATAATTTCCATCTGCAAC
CATTATTTCAAGTTCTTGCCTTAAGTCAGCATTTATAAACTTAAATTTTGGCATTTTTCTCATT
TTCTTAATACAGCCAAGTACCAATTTCATTCTTAATATATTTCTCAATTCTGTAATGTAGGAT
GCTGAAACTGGCAAGTCTCTATTTGTTCTCCGAGTATACTTTGAAATTCCCAAATCATAATCA
ACTTCAGCATATGTTAAAGTCTTATTTATGCATGAATACTTTATCATTGTTTCAGGGTCAGAA
TTGTAACAAATAGAAAAAATTTCTTCAGCTTTATCTGGGTTAATCTTTATTGTCTCAATTAAA
TTGTCTAAAACAGGCATTTGAGCACAAACATTTGTGTAATCACTCATCATTAAACAGTTAGT
TTCATACAGGGAAGCTAGAGCAGTTCTTTCAATAGAGTCTTTTATATCTAATAGTGATTCTAT
ATATTCAACAAATCTTTTTTCATTAAAAGGTATATTATCCTTAATAATTGAAAGTGTTAAGGA
ATGATCAGCTAGAGTCATTAAATAATCAACCATAGAACCTGGACCTACATTTAATTTATTTT
GAAAATAGACAAATGATAGTGTCCTCGATTTGTAAATATAGATTTCTTCTGGTTCAGATTCTA
TCTCTATTTTTGCCAAAACTTGTCCAAAAGGAAAAACTTTAACATTTATCCTTTTTCCTTCAT
GAAACATCATGCTTCCAATGCTTTTTACTGAGTGTTTTCTTTCATTAACGGATGTTTTAGCTTT
AAAATTGATAGTCTCAACCAAATCCAAAACCCATGATTTTCTTAATGTCTCCTCAGATAATTC
ATCTGATACAACATTTGGAATTTTTTCTTTCCCTGATGTATAGAATTTTCGAAGTGATGACAT
TTCATGTTCCATGAGAGAATGAGATAATTTCCAAAAGACAGAAGATCTATTACTGGGATTAC
TTTTTGATAATAGATATTTATAAAAATTTATCAATTCTGTCTGATTGGAAGTGACCTTATTAA
TGACAAATCCCTTTATGTTTTCTCGTAAAAGCATCAAGTCAATCCTTGCTTTATTATTCCATTT

TTCAGGGTAATTTTCATCATCAATGATAAACTTTAGATTCTCATTTGAAAGATTAGAGTATGG
AAAATCTAGCTTGATTATCTGATTGGGTGGCAATCTATTTTTCTTGGAAAGGACTTTTTGAT
CTCATCAGAAAAGATTTCAAACTTTTGAGGTATATGAATTTTTATTGGCTTTGCCTCAAAGTT
ATCTTCTCTCTTTATACTTTTTTGTAAAATAGAATAGGAAGTATTCAATTGAAATAATAATTT
CCCTATTATAAGATGTTTCTCAGGATTATAGCCATAATTTTTCATGTCTTCAATCAAGCTTTT
GTCTTCTGCAATAATTTCTTCATCTTCCTGATTCAGATTAAGTTCAAATTCTAAAATTTCCTCT
TCATCATCTACCTGATCTGACCACCTTTTGCCAAGATTTTGTGAAAACAATTCCATTGCGCTT
TTTATTGTGTAAATTTTATTTTATTTAAACCGATTCATTGATTAGTAAAAAATTACTTAGGAT
TTTCTGAAAACTAGAAATAGCTTTAATTTCATCATTTATTAAAAGGTCATCTGATGGTTTATC
AAATGTTTTTCTGAAAATGATCATATTATCATTAATTTTACAGATGTATTCCAGAGGATTATA
AAAAGAATAAATTGGAGTTTTATTATTTATCCTGTAACCAACAAATAGTTCCACTAGTGTTTC
TTTGTACCAGGTTTCTCTGTTTTTGGTCAATTTATCCTTACACCATACTATGAATTTTGGCTTA
GATATTATTTCTGGGCAATCCTTTATCCACCATATACCAAATGGAAATATGTCAATAATTTCC
ACACCTGATTCTTCTTTTATAGTTTTTATCATATCAGAAATATCATATTTAGGATTTACATAA
CTTTCCAAGTTAGAAATTATTGCAATTGGTTTCTCTTTATAGAAGTAATTGACTCTTCCAGGG
ATTTCCCGATTTATGATTTTCTTAATTGGTGAATTACTCATTTTATTTAATTCTTAATTTCAAC
CTATCCTATTTTTGTTATTTAACAAAATCCC
>Seq91 [organism=Viola ophiovirus] RNA2 genomic sequence
CCCGCCCTCTCAAACAAGGTCGTGAGAACCACGTTATTGCGATGCAACATTGCCACAGTGTT
ACAAAACAAAGAGACACTAGTCACACCCAATGATTATCGGTCACAGGGCATTAGGCTAGTC
TCTCCCGTGGACAATCAATACTGATAGTCAAATGTTATTTTATTTAAAACGATAGTCGGAAA
AGCGATTTCAATTAACTATAGATAATTTTTCTTCACTAAGAATTGTCATGAAGACCAGATCAT
CTCCGGAATAGTACATGGTCCTTTCATCTGACAATTTCATTGTGTCAATGATTCTATGGCTCT
GCAAAAGGCCAAGTGATAAGATGTTGATTTCAATTTCATCAGTCACAAATGCTTCTGGGAGG
CAAATGTTATGATCATTCAAATCAACAGTTACATTTTGCAATATAGTGCCCCTTATAACATTG
CCATTTGCAGTGTGGATGATCTTATCTTTCTTTTCAGCACTTCTGGCTATAACATGCAATGCA
GATCCAGAATCAATTATCCAAGAATCCCTTGACAAAGCTTCTAAATTAAATCCAATAGTATA
TAAAGGATTTTCTTCAGAGTTCTCATTTTTCTCATTTTCAGATTTAGAGCTTTCTCCCATTTCA
AGAATAACCTTCCCTTCTCGATTATCCCTTTCTTCCTTAACTCTTTCTTCAAGTTTAGACAATT
TTTTGTCCTTTATTGCCTTTAGTTCACCTGTCACTGTCCTGATTATACTCTTTGAGTTTAAAAG
GCTTGGTCTTGGGCAATTGTTGAGTAATAAACAAATTGGCTTGTTTGTCTCTACTTTAAGTCC
GATAGAATTTGCATCAATATGTGTCATGATACAAATTGAAGCATATGCCCTGTTGCAGACAG
GAACTTCTGTTGATTTGAACCTTAACACAATACATTCAGCATCTGAAGACAGTATTGTGTAA
CTCATACTCAAAAGACTAATAGCCATTTCAGTAGCAGGATGAATGACTTCTTGGATAACCCT
ATTTGTAGAAGTATCAAATAATTGAATTGTGATTTCAGGATTTAATCCCTCTACTGCTTCAAA
GTGTGGAATATATAAGATGTCAACCCTTTCATACCTTAAGTATTTTGGGGTTTTCTTAGTGAA
GGAAATTATACTTTTTAGCTGATCTTTCATCAAATGAAGCTTTATTTTCTTAGTTTCATTTTTC
TCTTTTAAGCTTGTGGCAATAGTGTAACTTGATGTCCAGATTTTATTCCGAACAAACCCTTCA
AAGTCACCAGCAAGATCAATAATTCTATCATCAGACTGATCAGGAATTTGGACAACAGTATT
AAAGCCCTTCTCAATCTCTTGAATTATTGGAGCATCAACATTAATTCTTCCTGTAGGTCTTGT

GCTGTTTGTTGGAATAATTAGTTCCAATTCATCTTTCCTGTTTAGAGAAATTGACCTTGTGAA
CTTTTTTACAGATTTCTTTTTCCTAAGATCAGACATGCTTCTTCTTGGAGCTTCCATTGAGTGA
GATTTCGAATTGATTCGATGCTTTAATTTCCAAAAGAGCTTGTTGTTTAAAAAACAATATCCC
>Seq92 [organism=Viola ophiovirus] RNA3 genomic sequence
TTTTATTTAAACCGAAATCACAAAAGACTGATTTGAATTAATCCTTATCAAACACTGCCTTAA
CAGAATCTACAGTCATTCCCATAAAGTCAGCATCATCATCATCCAAAACAGGCCAACTTGTC
TTTCCTGCAGCATCATCTGTTCCCATGAGGTCAGCATCCTTTGTGTAACTGTACATTTTCTTCT
CCCTGAAGTAATTTATCATATCTAGTTTTCCCTTGTTGGTTAGGGTGTACAGTATAGCACAGG
TGCATTTCCTAGAAAATCCCTTGGGTGCTGATCTTCTTGGATTCATTGGGTGGAGCTTCAGGC
AGGCCTTCCAGTTGTTCACATATGAAGCAAGTGTAGCAACAATATCATTTGCTTTTACCAAA
GCATTGTTCAGGTCAATTTGCTGCTTAGATACAGCTCTATCACCAAAATTTTGAATAGGAAC
CTTTTCAAATTTTGATGCATAAACTGCATAGCGCAATACTCTAGTTCCAGCAATTGATATTTT
GATTCTTGAGAATGCAGTACCAGGAAGACTTTCAAGATTCACCTTAAGTAGAAGATGTCCTG
GGAACTTCTTGGGACTAGTACTAGATAGCCTAGTAGCAATTTGCCCTTCAGTGCTCACTTTCC
CATTAAACAAATTCCTTATGAATTCGGGAAGTTCTCTCTTTTCAGCACCTTTTATAGTAGGAA
CAATCATGTTACCCCTTATCAGAACCAATATAACCAAGGAAAGAAAGCATTTCTGACCTATT
TCAATTCTCTTTTTTACCTTTTCAAGAGTAAGCTTTGAAATATCAGCAGGAACTTCTTTGAAA
ATACCAGCAGAATTGGGTTCAGAAGAGAGTGCAACATATAAAATAGCATCAAGCACTTTTG
TTCCAGCTGCAATCAATGAAGGAACATCTTTCTTTTCCTCATGGATAAAAACTACAATGTCCT
TTTCTCCATAGGATTCCTTGGCTGGCAGTGATTTTATGTAATTGTCCAGAGCAGCAATTACTT
TATCTTCATCAATATCAAATTGAGCACCAGCAACAGCAGCATCAAAATTATTAGCATCAACA
GGTGTAAAAATCTTTTGATTTTCACCTTCTTCAACTTCTTCATCACTTTCTATTTCAGTTTCAT
CACCTTCTTCTTCTTGCCTCTCATCATTATCAGAATCATTTGGTTCACTAGGAACAACCCCTTC
CAAATTTTCAACAGCTGAAGCAGAAGTAGCTCCCCTTATAGGATTTCCAGCTACTGTAGACT
TTGGAGAACCAACAACAACAACATCTCCATTATCCAGAAGATCAAGTCTGGAACTACTTGGC
TCAAATTTCTCTCCTTGAGAAATATACTTGTCCAAGGTTTTAGCAATCAACCTGTTATTGTTT
CCAATAAGACCATATGATCTTAACTCTTTTAAACCTTCAGGAGGTAAGACATTTTCCCTTAAC
TTCTTTAGTGAATTCTCAAGAGCACTAAAATAGATAGAGGACTTAATTGTCGGAGTTTGTTT
AGGAGGAACCATATTAAGGAAATTCGGAATTTTAGCTTTTAAAACTTGGGAATAAGAAGCTT
TTTGTTTTTT
>Seq93 [organism=Viola ophiovirus] RNA2 genomic sequence
CGCCACTTTCTCATGCTGCCTGATCGCAATGATCTCGAGTGAAGTGTTATCCTTAAATCATTT
GATGCTCCGCAATTGAATAACACCAAAATGATTTAAAGAAAATCACTTCAGGGGCAGCAGG
CTAGTCCAAGTTTCTTTAAAACGAACCCGTAACAAAATGAAGTGTGATCAAGAGTTAGTGTG
AGGTTTGATCCAAGTACCATTTGAGTCTTGAATCATAAACCACATTCTTCCTTCTTCAGTATT
ATCAATCCTGAATACTGTTTCATTCTCTTTTACCAAATATATAAAATCCCCAGTATCATCCAT
CCTAGTTATTAAGCCTTGTTTTTTAAGCTTACTATAGGAAATCCTATTTGGATCAACATTATC
CCCTACAAACACATCTTCTAATTTAACAACCTTTCCTCCAATTATAGTTGTTAATTCTTTAAC
CTCAGAATAAGATTTCCCGTCTTCAATTATTTTTCCTTTATGACCTCTCAGGTGGATATAATG
CTTTGAAGAAACAGTATCAAGTATGAATCCTTTTTCACTTTTATGATCACTTAAAGCCCATCT

TATGACTGATGAGTCATCACTGCCAACTGATTTCACATGTTCGTACTCTTCAAATTCGCCTCT
ATCCTCTGTAATCTCACTTGAAATAGATATTGCACTACTAGTGGCTTTATCCTCAAGGAACAT
GTTGTTTTGAAGTGATAATAACCTTTCTCTTTCAAGGTTCATTTTATTTTTCATTTTTTTGTCAT
TTTCTAATGACAATGCTTCCAACCTTTCCCCTATTTGTTTGAATATGGAACCCAATTTTACAT
CTTTGGGTTGGTCTGATGGATCAATATACATGATTTTTGAATTTTCATGTTTATAGCTTCCTGC
ACAATGAGATGAGTGAACAAAGAAAGCTATCATAACTGAAGCGAATGAACCAGATTTGGTT
GGTTCATTTTCTACTTTCATTAATAATTTTATTTTCTTTATATCTTTTCTAGGAACAAAGAAGG
ACATATTCATCTCTAAGAGAGACATCTCATTTATTGATGCACTTAGCTCAGCAATTGTTTGGT
TTTCTAAATCTCCATTCATCCTTTCATCAACCAGTATAAAATTGATTTTGTCATCTTTATTCTT
CCTGCCATCGAATAAAGGAAAATAAACCATTTGAATTTTGCTAAACCTCATGTAAGGTTTAT
CTTCTCTATATACTGCCTTCTTTATTCCTCTCCAAATGTTTGAGAGAGAATTTACTGTTAAAG
ATGCTTCACCTGTTTTACCTTTCAAATTAATTTCTATTGGAGAGAATGCAGAATCAATCCGAT
CTTGTGTTATTTTGGCAAATTCACCAGCCATACACTGAATTTCAGGAGATAAATCTTCATCGG
ATGACGAACCTACAATTATGATAGCTTCATCTTTTGCATCGTTAGTTCTTGTCTTTGATATAG
ATATCCTTGGATGCTGAGTCAGGGAAGTGCTTGACGTTGAATTCATACGTCTTAGAGAGCGG
TTATTTGCTGAAACTGAAACCATTTTCTTAATCAACAATAATACCACGCAATGATCTTAC
>Seq94 [organism=Viola ophiovirus] RNA3 genomic sequence
AACCGTTTTTGGCGTGAATGGAATTTTATTAAAACGATGACATGAGGAGCGGAATTAATTAT
GCAAGTTCTGGGGGAATCGTGAAATCTTTCCCATACATGTTCTTCAGTGTCTCAACAGTGATT
GAAGAAAAATCGCCTTCAGGCGTATTCAGCTCAGGGTAAACCCTCCTCCCTGGCTCTCCAGG
TTTTCCAACCAAAGTTTCATCTTGTAAGAAAGCAGTATTTTGTTTCTGCTTTAGATGGTTGTA
AAGATCAACTCTTCCCTTATCAGTAAGAGAATAGATGATCGCACAAGTTATTTTTCTTGTCAG
GCCAGGTGTTGTTGGCCTTTCAGGGCTCATGGGATGCATTTTTTTCATGGCTTCCAAATCAGT
TTGAACAGCCAACAGATACTCAAGAATACAAAAAGCTTGTTTCACCTTCTTCCTCTCAGCAT
TGTTTCTCAAATTCTCTGCTTCAGTACCTCCAGGAATAAAAGCTGGAATTCCTCTTCTTTCAA
ATCGTGAAGCACTTATTGCATACTTCACGATTTTGTTTCCAGCAACAGACAACTTGCACCTTG
ATGCATCATTCAACGGTAGTATATTCAGGTTGATTTCAAAGAAGATCTTTTGGCAAAACTTC
CTTGTTGAAGTACATGATAAGAGCTCTGGAAGATCTTGCATCTTAGTAACATTCAGCTCGAT
TAGATGTTTCAAAAGATAAGCAGGAATTGGCCTTGTATCAGTTGAATCAATGCTAGGAAGGA
AGCCTTGAATCCATACCATAATAGTACAAGCTCTTATCAATCCTCTTCCTTGTCTTATGTTAA
AAGACATATCTTCTTCAGCATATGACTCAATTTTGTGAATGTCAAACATTCCGTTTGACTTTT
CATCAGTCGCTAGAGAAACAAATAGTAGTGCATCCAACGGTTTTGTTCCTGCACCTAACAGC
TCACAGAGAGAAAATTCAGAATCTAAATCCCCAAACCTGAAAATTTTAACCTCATTAGCTGA
ATAGCCTTGACCATCAGCAGGTAAATTGTTGTAGAACTTTTCAAGTTCTGCTTTTACTCTGCT
AGCATCAACTTGAAAATTCTGACCAGATAAATTCGTCATCAATTCAGCACCAGACTTCATCA
CTTTAGATCCCTCACCTCTCTCATTGTTTTTCTTTGTTTCTTGGCCTCTTTTTCGTGGTTCAGGA
GGTGGTTTATTAGTTTCTTCTTGAATAGGACACTGTTCAAGACTTCCGGCCTCATCAACAGTA
AGTAGCCAATCTTTATCGCTGTTGGCCATTTCTTTGAGTACAACCAGATTGCTGGGATTAAGG
GCCCGATTATCATAGCAGATTTTGAATTCTTTGAGAAGGGACCACTCAGCATCGTCTAGGTT

CGCCTGGTCCTTTTCATACTTTTCATGGAGGGACTTTAATTCGCTAGTCCTGATTTTAGACAT
ACTTGAATCACCTGAATGATCAATAACTTAATGAGCAATAGAAAT
>Seq95 [organism=Xerochrysum ophiovirus_brac] RNA1 genomic sequence
GTTTATACATAATCCTTCTTCCAGATCGCAATGATCAGCAGTGTTACAACTAACTCTATTGAA
CTATACTACAATACATAATAAATTTACAAATAGATTACCATTCAAACTCATCCTCATAATCA
CATCCTTCATCTTCTAGGAGTTTTAAGTTTTCCTCATATGACCTATTTCTATTAGCTTCAATTT
CTTCTTGAGTGTTGTCTGTAAATGTAGGAATTGTATCAGGTTCAATAATTTCTTCAGACACAG
TATACATTTTTTCAGTTTCCTTTTGGACATTCGAAACTAACATAGAGAAAAAATCTGGTATTT
CATTTCCTTCATCATCGAATTCTTTGTTATTTTCCAAACTTTCAACAATTTTCATTAATTCTGT
AGAGCTCAATTCAGGTTCATCACATGCTGGAGTATCATCCATATCGGCCCATTTTTCATTTTC
AGTTGGATTTTTCCTATACCTTGGAAGAAGATTAAATTTTTCTTTCAGTAATCGTATCCTATT
GATTAACAATTTATAATCCATGATTTTATTCCTGGTTGTTTTCCCTTTGTTTTTCATTAATTCA
TTTAAACTCCCTCTCCTCATTCCTGTTAAAAGTATTAAATTTTCCATTGTTTTTCCAGTTTTAT
AAGAGACACAAGCTATTTTTGCAATTTTATGTGTTGCATCAATGGCTTTTATCTCTTCTTTCCT
CAATTCTGTTAAATTTATAATGTCTATTAATGAATTCAGAGAGACCTTTTTATCTGATTCCTTT
ACAATCTTAGTAAAAATTTCCCATAATAATTTGGCCTTTGCATAATTCAAACCATTAGTATCT
ATTTCTAGGTGAATTTCTTCCAATTCTGATTTAGTTAAATTATCAAAATTCTTAATTGCTTTTA
CAAAAGGAGTTGAAAGAATTTTATTATTAACTCTTACAAACACATCATCAGAATATAAATCA
TTATAAGTTTCAGTGAATAGACAATTATCATTTGATATGAATCTAGCTTCTCTGAATTCTACT
TTTTTACATAATGATGAATTATTTTCTATAAATTCTCCACTAAGAAATACCATTGAGTATTTT
TCTACTTCTTCTTTGGTTGAGAATTGTGAATGAAAAGATAGTGGTACATGATTTACATCACTA
TCAGGATCTAATATCATTTCACACAAGAAATCATCTGATACTGTCTCCATGGTATGATCTAGT
ACATTTTCTGGTTCTCTTGGAAATGGTTTTAGACCTACCATACATTTAATATTGTTAGTTAAT
CTTTCTAAAATTACAGAATCAGTAAATGTTTTTACTTTTAATGATCTTTCTAAAGTTTCACCT
GTTAAAGTCTTTTTTGGTTCTATAGTTAGGTAAATTACTCCAGGTGAAGACAATGTTGGAATC
CTTAAATTTAGTATAGCATTATTATCCAAAAAGTTTCTAAACTCTTGTGATTCAAATATTTCG
CATGTTGTATTAAATCTCATTATTATTTTTTTATCTTGTTTAATAACTAATTCTATTAATGAGA
TTAGTTTTTCTTTTGTTCCAGTTAAGTGTGAGATATCTAACAAAAATATATCATTTTCTAAAT
ATTTTACATATTCGAACTCATCAAGCGCATTCCAGGAAGCTTTCTTTTTTTCCATCCCTGTTAT
TCTATTTATAGTATTATAGCCATCTAATCTTGATACTGAATGATGATTAATATCCTTCTCAAT
CATAGCCAGGTGAAAATCACCTCTTCCTCCAAAAAGGTCAATCACTTTATCACCTAATTCTA
GATTTACAAATTTTTCATTGCACAGTAGGCTCATTAATCCCCTAGCCGATGGATAGATATCA
GATTTGGTTGGAGAAGATATATGTTTTGGTAAACAGTACATGGCAGCCATCATTTCATAACC
CTTGATAGCCGCTTTCATTGCTTTTGTATTTTCAAGGTCTTTTGGCTGTACAATGTGTGAATCA
AAGTCAATGAATCCATATTCTACATTATTGTCTTTCATTATTTCCTCAGACAATTGTTCTTTAT
AGTATTCAGTGATCCTTTCTTGTTTGAATTTCATTCCATGTAATGAGTCACATAATTCATCAC
ATGCTGATTCAAGCTCATCGAATGAATAAGATAACACAGGCATTGCATTTATTAACAAACTA
CATATCTTCTTGTCTTTTGTCCAAAAATGATGTTCCCCTTTCCTGATATTATTCAATGCATTTC
TTGAATTTATTTTTGTGAGTCGTCTATTTACTCGTATTATATTCTCTACTTCTGAAACGTCTTC
AACTTTAAAAATTAACCCACATGATATTACAAATAACTTTAAAAGTGAAATTCTGTAACTAA

CTGAGAAACCACTTAATGCTAATTCTTTTACCCAAGATTTTGAGTCATGTTCCCTATGAAACA
ATTCATGATTTATCAAAGACCTAGTTATTGCTTTCAATGGTGTCTCATTTCCTATTATATTCAA
GTCTTTATAATGATTATAAAATTCTTCAAAGAAATATTCTTTTGAAGCAAAATCACTTAAATT
GGGTATGATTTTTTGTAAAGTCGGTAAAGGAATTGTTTCTGGAGAAATTATCAATTGATCTTC
CATATAATTATACAGGTTAATAAATGAAGATTCATTTTTGATTATTTCGTCTGGTATGACCGC
ACTTTCTGATATGAATTTTCCAGCCAAACATTCATCTTTAGCAATAAAATTCGAATATGACTT
ATAAATAAATGCTTTTCTTTTTACATTTTCGAATGGTATTAGAGTCATTGCCTTTTTTGACATG
CCAATGTGTAAATGTCTTATTCCATGAATATTAAATGTGACATCTTTGATTTCTTCTAATATA
TCAGATCTAATACTCAATGTTCTGAGTTCTCTTCTAGCAGGTTGTAATAGTCCCATACAAACC
TTGACTATGGTGATAAGGTATTGTAAATTCAAGTTATTATCTTCTCCTCCGGTAGAGAATATT
AGTCCAGTTATACCTGACACGTCATATTGTCCACTAATCCTATTTGAACTTAGGTCTCCTGTC
CTTGGATTGAACCCTCCTGCGGAAGCCCGATGGAAATATCTTCCTCCTCTTGGACAAACTAC
ATAATCAACCATATCTTTGAATTTTACAGTTGAAAATGTTGATAATGTTAGGTCACAAACAC
TTACAAGGGCTTCCTTTTCTTCATTTGTCACTTCACTGAATCTTTCTAATTCCATTATTATCCA
CTTGGTATATCGAGTGAGCTCATATGCACTGAAAATTAATCTGGATTGGAACAAGCCTTGAA
CCCCTATATCCCTATCAAATTTAGCCAAGTTAATGTGTGATTTCATTGGTGGGTGTTTCTTAG
GTCCTGAACTAGTTAGCACAGTATCACCTTTGAATATTGGCAAAATATTGTGCGATAAAACT
CTGTGCTCTAAGGCTCCCAGTAAAGGGATTTCTTCTATGTTAATGAAATTTAACTTTACAGGA
ATTCTTTCTTCTAATTTTATATTCATTGTTTTTCTTTATTATTCCTTTCATCTACAATAGACCA
ATAAGAGACTAAATGCAAGCCAGATCTAACCATTTCTATCTTTCTATTCTTTTTCCAGACTTT
ATGCCATAATTTTCCCATCTCTTCCTTACTAACTAATTGCATAAAAGTGGATGAGTTTTCTAC
TTTGCTACAAATAGTTTCAACATAGCTGAAAATTGATTCTTCATAATATTTGCCAGCTATTCT
AGCAGAAAAACAATCCTTGAATGCAAACACTAATTTGTTTAGGAATTCTATTTTATTATTCTC
TTTTTTCATTTCAAATATTTTCTTTAGATTTTTATTTACAGTGTGATTTTCTAATATTCTTTCAA
CTTTATCAGCCAAAACTGTGCCTGTATCAAATCTTCCTCCTGTTAAAGGGTACTGATTTGTGA
CATAATGTTCTACTTTCGTATTAAAATTTATCAACTCACCTATGAAAGTCTTTCCTTGATATG
ATAATAAACTCTGAACATAAATGATCCTTTTTACGTTTGATAATGAATACCCAGATATATGT
GCATCAGGTATTGGGGTCATTGCGTAACCATACATTGTATAAGGTAAGTACATAAAAGATAG
TATAATTGACTTTACTTCATCTATTTCTCTATTAAAAACAAAAAAGTTAGCTATATTTTCATC
TCTTTTGTCCACTAGATCATTTATTTCATTTGTTTCAAGCTTTTCTTTTAATAATTCTTTTTGAT
GTGTAACACTGAAGCTTTTTAGTGACCTATACCTGTTATCTACATTTAAATAAGGTACTTGTT
CAACTAAATTTATTAATTCTTTTTTTGAGGTGCACAAGAATCTATAATTTCTAATTATTGTTTG
AATTTTTACACTATCAGACCTAGCTGAAGCTGATCCATATCCAGAATCGATGAACTTAACTA
ATTCTAATTCGTCCCCCCACATTGCACAAAGATTAGGTGATATTCCCATCATTCTTTTGTATG
TTGTATCTACAATAACATCTTTAAAATAATGTTCTTTCAACATACTGCATCTACTATTTGTCA
CTTGTGTTTGTGAAAGTTTTACTAATAAGCCCATTTTCAATCCTGACAATTGAACATGTTTAA
TTATTTCATTTAGTTTATTGTCTGTCATCTTTTCCATCTTAAACACAGCACAAGAATCATCAG
AATATGTTGTAGCTAAATACTTGGATAAAGCAAGTGAACTCAACATATCTTCTAACATAAGT
TGTGATTGAATTCCCCATAAAGGACCAAACCATCCTTCAATAGCACCTTTTTGACCTTTAGAA
TAATATATCAAATCAGTGAAAGGTTCATCATTAATGACAAAAATATTAGAAAACAAATATAC

AATTGAAATCATATTTTCATATTCTTCCATACCCTCAGTATAACCATACATGTTAGCTATTTC
TTCAATTATGAATAATGTATTTTCCGGCCTTTGTGAAGTATTGTGGCCAGAATAGTCTAAAAA
TAATGAATAACAATTATCATCGGATATCATTGTGCTCATTTTATACATTATATCCCTTCGTTC
ATCTTCAGACATAGTCATCATTTGTCCTGGTAATAATTTCATTGCTCTCTTAATTCTTTCCATC
ATTGATGATATCCACAGTTTTAATCTAAAGCTTGCTATTCCAAAAAATCGTGCAGCTGGTTTT
AATTCTTTTTCTTTTTCAGCTAGTCTCACAATGAAATATTTTCTCATCTTTTTTAATTCTTTATC
TTTTCTATCATATACTCTCACTACTCTATCTTTTTCACTATAATATTCTTCCTTGTTGACTTTCA
ATTCATCTGAAAAATCTCCATCAAAATCATTTTTAATATAATATATTAATTCTTTAATAGAGT
TTGTTTTTTCGTCATATTCATCTTTTGTGCAAGCTTTATCAATTATACGTGACTCTAAATTCAT
TTCATCACCTTCTTCTAACATTTTTCCTAGCCTTACATCTTTATAGTTTACAGGTTCAGAAACA
ATCAAATTATTATAATTTCCCCCAGCTGCCATTATTTGTAATTCTTGATCTAGAAATGAGTTT
GAATTTAATAAAAGTGGAACTCTACCATACTTTTTGATATAATTCCTAATAACATTCATCCTG
AATAGTCTTCTTAATTTTTCAATCTGATGTGAGTCAACAGGGTGATTTCTGTTTGTTCTTTGA
GTATACTTGGTTAAGCCTTGATCTTTGGAAACTTCAGCATAGATTGTAGTTTTCCCAATGCAA
GACATTTTAATGCAAGTCATACCATCTGATTCATCACACATCTTGATCAGTCTTTCTGTATAT
TCTGGTGATAATTCATATGAATCCAAAACATTGTCGAGAATTGGCAAAGCAGCCGCAGTTCC
TTTTGTATCAGCCATTAATAAACAAGTTGATTCATACAATGCTGCTAAACCATTTCTCACTCT
TGTTTCAACTATTTGAGTTATGGATAATATATAATCTATGAATTGTTTTTCCATTGGTTCTGCC
ATTTCCTTTATGAAATTCAAAGTAAACTCATGATCTATCATTGAGATAACATACCGAATCATC
GAACCATCTCCAATGTTAATTGTTCCATTTCCATATGAGAATGCAGAAAAAGTTGATGAAAT
GTACCAAGTTGTCACAATGCCTTCTCCTTCTATTTCTAAATCAATTATTGATTGACCAGTGCT
ATATACTTCTACTCTCACTCTTTTTCCTTCATTGAAATACAATTCACTTTCCCTTGTTTGATTTT
GCATTTTCATTGACATATTGATTTTTATTTTCCTATTCATGTTTTCAATGATGTCTAATAACCA
TGCTGATTTTATCACATCTTCAGAAAACTCTGTTTTCATATTGAAATTCTTCTTATTAAGTAGT
GATTTACTTAATTGATTTAATTCATGGTCATATAGATCAGCAGAAAAAGATAATAGTTTTCC
AGAAGTGAGTGAGGGCCTACTCAAAGATCGCAATTTATTCATTAACTTGATGCACATATGTT
GAGAAGAACCACAATTCAGTTCTCCATGTCTTTCCTTCATCTCTGCTAAATAACAATTCCTAA
AAAGCCACAAATCTTCTTTCATTTTCAGTCCCCATTTTTCTGGATACAAATGGGTTGATGTGA
ACATCTCTATATTTTCTTCAGAAAATGATTTAAAAGGAGAATTAATTTTCTCAAGCAGTATTG
GTTTTGTTTTGATGTTCCTTTCAATCCATTTGAATCCGACAGTGTGATTGAATTTCATCTCAAT
TTTAACTTCTTCCAATTCAGGTTCCATTTCAGCTTCTTGTTTTCTCAACGAGGTCAAGTCATGA
TGGAATTTCCCATACTTATTCAACACATTTTTAGTATGAATATGGGAATCATGAATAAGATTA
AATAGGGCGCTCATGATTACAATAATTGATAATAATAATAACTTGTTTGCCAGTATATAAAT
ATAAGCTTGTGGTTTATAAACCAAATGATTTTTATTAAAACGATGAGCATTTAATTACTGGA
ACTAGAGGAAGCCTTTCCCTTGATTTTCTTTTTATCCTTTTTACACAATCTACAAATTTTCTTC
TTAATTGGACTTCTTATTACTGTTCTTTTCTTGACTCCAAACATTTTCATGATGGATTTCAATT
CATAGTCAATCCTCCTGCAAGTTAGGTTGACATATTGAAGCTCAGGATTCTCCTCATCAGTGT
AATCAGATGTGATTAATTCCACTTTCCCTGAATGATAAAGGATAAACTGATTTTCCATACCC
ATCAACCGAGGTATTTTATTAAAGTCATCAACTGACCTGGCATAGACTATGCGAAGTTCTTC
AGAATCATCAGAGCAAGGGATTATGGTTTCATCACAGATAACTAATGCAATTTTACTCAAAA

AATCATTCGTCTTGAAAACCCATAATCCAAATAGTAAGACCATCTTTGTTTTTCTTTAAAAG
CATTTCTTTCTTTGGCTATCTCTAGTAGATTATTAAGTTCATTTGTTTTTCTTCGCACAAGGT
GATTTCAATCTCTTTTGTCCATTCAGGAAAGATGTGAGGGTCAACATAAGCTCTTTCTACTTT
CTTTAGACATTCAATCGACATGTTTTGATTAAAAACAAGAAGAAGTTTTTACTTTTTAAAGTA
AACCTGATTTCAATCTCTTTTGTCCATTCAGGAAAGATGTGAG
>Seq96 [organism=Xerochrysum ophiovirus_brac] RNA2 genomic sequence
CCGATCTCTGAATTCCAGTTCGGAGGAGGACGGGGGCCATGAGGGGTAGTACCAGGATCGC
AATGATCAGCAACATTGCTCTACAGATTTACATGGTGAGATGGTCGCATTGACTTGTATCCA
CCTATGTAAATCTAAGAGTCAACGGGGAATCTCCCTATTCCGAATACTTTAGTTTTTATTAAA
ACGAATACTAGGTGAAAATTACTCAATCATCCTCTTATAAACTCACCTGAGGCACTCTGTAC
CATGAACCACATTCTACCATCCGAAGTGTTATCAATTACAAATTTAATCAAGTCCCCTTTCAT
TAAGTGAATTGTGTCACCTTCATCAATCATCCTATCAATAATCCCCTGCTTTTTTAGTTGACT
ATATGAAATAAGATTTGGATTAACATTGTTTCCTACAAAAACATGGTTCAATTCAACCATTTC
TCCACACAAATCTAATACAAGGGAATTTATTTCAGTAACCCACATTCCACCTATTTTTGTTTT
ACCAGAATGCCCTTTTAGATGAATAAAATGAGATGGAGCACCAGTGTCCAGGATATATCCA
ATGTCACTTGAATAAGTTTTCTTGGACCACTCTTCAAGATTAATCACACAATGTGGAATTAAC
TCTTCCACTGACTCTTCTTTGATCTCACTAATTAATGAAGTTGAGTTTCTTCTAGGAATATCTG
AATTCCTTTCTGCTACATTTTCTAGCAACCCATCAGTGAGTATTTCTTCAGCTCTTTCTAACCG
ATCTAACTTCTTTTCAGTTAATAGAGAAGCCCTTTCAAGCTTCTTTCCAATGTTTCTCAGAAC
AGAACCTGATTTGATTGTTTTCGGGTGACTTGTTGGATCAATGTAAAACACTTTTGATCTTTC
TACTTCATAAGTTCCAGGAAAATAAGATGAATGTACGTAAAATCCTATCATTAAGGAAGCAA
ATGAACCAAACTTTGTTGGTTCATTATCAACTGTTGTTATTAACTTTATCTTTTTAATATCATC
TTTTGGAACAAAAAAAGACATGTTCATCTCTGACATTGACATTTCATTTATTGCAGCTGTAAT
ACTCGCAATTACTCTATTCTCTTTGCTTCCTTTCATCCTTTCATCAACCAAAGAGAATGTAAT
CATTTCTCCTTTATTCTTACGCTGATCAAACAGTGGACAGTAGAGAATTTGTACCTTGCTCAA
CCTCATATAGGGTTTATCAGCTACTTTTATTATTTCTTCAATCCAGACCAGACTTGTGCGAA
TGAACTAACAGTAAAAGATGCTTCCCCAGTTTTTCCTTTCAAGTTCAGCTCTATTGGTGAAAA
TGCTGAATTTATTCTACCTCTAGTCATTTTGCTAAATTCAGCTGCCATGCATTGAACTTCAGG
ATTCAATGGCTCATCTCCTGACAAGCTGATTATTGTTCCTTCGCCAGCAGGGAAAGTTTCACT
TATCGCCCTTTCAAGTGATATTCGTGTTTCCGATCGGTGGAGTTCCGTAGGAAGTGTTGTTGA
TCTCCTGAGTAATCGTATTCCAGAGTTGGCAGATTCCATCGAATTGTTTTCTGTTTAACAGCA
AGGAGA
>Seq97 [organism=Xerochrysum ophiovirus_brac] RNA3 genomic sequence
CCGATCTTGTAGCCATTGTTCCCTACTGACCTAGGAGTTTTATTAAAACGAAAGGCAGTGCA
ATAGAAATACTGCAAAGAAACTTACTTTGCATTGCCAGCAACCACAACAAACTCCTTCCCAT
ACATATTCTTCAAGGTATCAACTGTAATTGATGAAAAGTCACAAGATGAATCGGTCAAGTTT
TTATGGATCCTTGTTCCAGAGGGGCCAGTCATCCCAATCAAGTAGGAGTCCTTCAGAAAAGC
CTTATTGTCTTCCAGAGTCAGATTGTCAACAAGCTCAATCCTTCCTTTCATTGACAAAGAAAA
TACAATTGCAGTAGTTATTTTCCTTGTGAGGTTTGTTACTGATGGCTTGAATCCATTGAGTGG
GTGCATCCTGAGCATTGCTTCAAAATCAGTTTGAACAGAAAGAAGATAGTCTACAATGTTGT

AGGCTTTTTGCAGTTTAAGCCTTTCATTGTTTACCTTTTCAAGTTCTTCCATATTTTTTGGAAG
AGCAGGCATTGGGGCTCTCTCAAACTTTGAAGCAAGAACAGCATATTTCACTATCTTGTTTC
CTGCAACAGATAGTTTACACCTGGCACAATCAGCATCAGGAAGAGTAGCTGTATCAACTTTC
AAAAAGATTTTTTGAGGAAACTTCTTTGTTGAAGTGCTTGATAAAAGCTGAGGGATCTCCTT
CATCATCGTGTACTTTCCATCCAAAAGATGCTTTTGGATATAGCTTGGTATGGGTCGTTGGTC
TTTTTCATCAATGATTGGCAAACCACCTTGGACATAAACCATGACAGTACAAGCTCTCAACA
ATCCTTTCCCTTTCTTGATGTTTTTGGCCATAACACTTTCCTTCATCTTGTCAACAACATTAAC
ATGAAACATTCCATTTGATTTTTCGTTGTTACCTAACGCTTTGTAAAGTATGGCATCAAGAGG
AAGAGTACCAGCTCCAAGGTATTCAAACAAACTAACTTCTTCATTGGTGTCTGCAAATTTAA
ACACTTTTACTTCCATTGGTCCATAACCTTCTCCATCAGCAGGTAGTTCTTTGTAAAACTTGT
CAAGTTCTGCTTGAACTGCTTCAATATTCACTTCAAAAGAACGAGCAGCTAGTTTTGCCCTCA
ATTCTTCACCTTTGATTTCTTCATCATTTTCCTCTAGCTCTGGGATCCTTTCATCATCATCATC
CTTGTCTTTATTCACGTTGGAAGTGTTGGTGTCATCCCCTTCTTCAATATCCTTGTACATGAGT
TTTCCAGTGTCAGGGTCAAGAAACATAATATGATCCTTGCTAGGAGTGAGAGCGAGTTCATC
GAGAGTCCTGATATTGATTTTGATTTCATCCCTATCACTGTCAATTACTCCGAATTGTTCAAG
GGTTTTCCACTCTTCTTTGCTCAATTTTTGTCGGTGCTTTTTGAACTTGTTGAGAACTCTGTTA
AGGTGAGAAGCTTTGATAGTTGACATTTTGCAATAAGATGGAACTCAATAAAAAAGTATATA
ATTGAAAAATTAAACCCATCCCTATCACTGTCAATTACTCCGAATTGTTCAAGGGTTTTCCAC
TC
>Seq98 [organism=Xerochrysum ophiovirus_macra] RNA1 genomic sequence
TCTTCATAATCACATCCTTCTTCCAGATCGCAATGATCAACAGTGTTGCACTTAATACTACAA
TAAGTTACATTAAAATATATTACATAATATACATTAAATTGATTTACCATTCAAATTCATCTT
CATAATCACATCCTTCTTCTTCTAATAATTTTAGGTTTTCCTCATATGACCTATTCCTATTAGC
CTCTATTTCCTCTTGAGTGTTATCTGTAAATGTTGGAATTGTTTCAGGGTCAATAATTTCTTCA
GGTATATTATACATTGTTTCTGTTTCTTTCCTTACATTTGAAACTAACATTGAGAAAAAATCT
GGTATTGCATTCCCCTCATCATCAAATTCAACATTATTTTCTAAGCTTTCTACTATTTTCATTA
ATTCTGTTGAACTTAATTCAGGTTCATCACATGCAGGTGTGTCATCAATGTCAGCCCACCTTT
CAGTTGGATTTTTCCTATACCTAGGAATAAGGTTGAATTTTTCTTTCAATAATCTTATTCTATT
TATTAATAATTTGTAATCCATAATCTTATTCCTAGTTGTTTTACCTTTATGTTTCATTAACTCA
TTTAGACTACCCCTTCTCATTCCTGTCAGAAGAATTAAATTTTCCATTGCTTTGCCTGATTTAT
AAGAAACACAAGCTATTTTTGCTGTTTTGTGTGTTGCATCAATAGCTTTTATTTCTTCTTTTCT
TAATTCTGTTAAATTTATAATGTCTATCAATGAATTTAAAGAAACTTTTTTATCTGATTCTTTA
ACTATTTTAGTAAAAATTTCCCATAATAATTTAGTTTTAGCATAATTTAAACCATTTGTATCA
AGTTCTAGGTGAATTTCTTCTAATTCTGATTTGTTCAGGTTATCAAAAGATTTAATTGCCCCA
ACAAAAGGAGTCGAAAGAATCTTATTATTTACTCTTACAAATACATCATCAGAATATAAATC
ATTATAAGTTTCTGTAAATAAGCAATTATCATTTGATATAAATCTTGCTTCCCTAAATTCTAT
TTTTTCACAGATTCCTGGATTGTTCTCTATAAATTTTCCACTAATAAATGCCATTGAATACTTT
TCTACTTCTTCTTTGGTTGAAAAATGTGAATGGAAAGATAATGGTACATGATTTACGTCACTG
TCAGGATCTAATATCATTTCACATAAGAATTCATCTGATACTGTTTCCATGGTGTGGTCTAAT
ACATTCTCTGGTTTTCTGGGAAATGGTTTTAAGCCAACCATGCACTTAATATTATTAGTTAAT

CTCTCTAATATAACTGAGTCAGTAAATTTTCTTACTTTTAATGATCTTTCCAAATTATTATCAC
TTAATTCTTTTTTTGGTTCAATAGTTAAATAAATGACTCCTGGTGATGATAAAGTTGGAATCC
TTAAATTCAATATTGCATTGTTATCCAAAAATGTCCTAAGTTCTTCGGATTCAAATATTTCAC
ATGTAGTATTAAATCTCATGATAATCTTTTTATTTTGTTTGATGACTAATTCTATCAATGAAA
TTAATTTTTCTTTTGTTCCAGTTAAGTGTGAAATATCTAATAAAAATATATCATTTTCTAAAT
ATTTTACATATTCAAATTCATCAAGTGCATTCCAAGATGCTTTCTTCTTTTCCATACCTGATAT
TCTATTAATTGTATTGTAACCATCTAATCTTGACACTGAATGATGATTAATATCTTTTTCAAT
CATAGCTAAATGGAAGTCACCTCTTCCTCCAAATAAATCAATAATCTTATCATTGAGGTCCT
GATTTATGAACCTTTCATTACATAATAAATCAAGTAATCCTCTTGCTGAAGGATAGATATCTG
ACTTAGTAGGAGAAGATACATGTTTTGGTAGACAATACATTGCAGCCATCATTTCATATCCT
TTGATGGCAGCTTTCATTGCCTTTGTATTTTCTAAATCCTTTGGTTGTACTATATGTGAATCAA
AATCAATAAATCCATATTCAACATTGTTATTCTTCATTATTTCTTCTGATAATTGTTCTTTATA
ATACTCAGTTATCCGTTCTTGTTTAAATCTTCTTCCATGTAAAGAATCACATAATTCTTCACA
TGCTGACTCGAGTTCATCAAAAGAATAGGATAAAACAGGCATTGCATTAATCAATAAATTAC
AGATTTTCTTATCTTTAGTCCAAAAATGATGTTCACCTTTTTTAATGTTATTTAATGCATTTCT
TGAATTGATTTTTGTTAATCGTCTATTTACTCTTATTATGTTCTCTGTTTCAGAAACATCCTCT
ACCTTAAATATTAATCCGCATGAAATTACAAACAATTTTAACAATGAAATTCTATAACTAAC
TGAAAAACCACTTAGTGCCAATTCTTTGACCCAAGATTTAGAATCATGTTCTCTATTGAATAA
TTCATTATTAATTAGAGACCTAGTTATTGCTTTTAAAGGTGTTTCATTCCCTATAATATTTAA
ATCTTTGTAATGATTATAAAATTCTTCGAAAAAGTATTCTTTGGATGCAAAATCACTTAAATT
AGGAACAATCTTTTGTAGAGTTGTTAAGGGAATTGTTTCTGGAGAAATTATCAACTGATCTT
CCATATAATTATAAAGATTTATAAATGAGGATTCATTCTTAATAATTTCGTCTGGTATGACTG
CACTTTCTGAAATGAACTTTCCAGCTAGGCACTCATCTTTTGCAATGAAATTAGAATATGACT
TATAAATGAATGCTTTTCTTTTGACTTTCTCAAATGGTATTAATGTCATGGCTTTTTTTGACAT
TCCAATATGTAAATGTCTTATTCCATGGATGTTGAATGTCACATCTTTAATTTCCTCTAATAT
ATCAGATCTAATGCTCAAGGTCCTTAGTTCTCTTCTAGTTGGTTGTAATATTCCCATACAAAC
CTTTACTATAGTAATAAGATATTGTAAATTCAAGTTATTGTCTTCTCCACCTGTTGAAAAAAT
GAGTCCTGTTACTCCTGATACATCATATTGTCCACTTATCCTGTTTGAGCTTAAGTCACCAGT
CCTAGGGTTAAATCCTCCTGCAGAAGCCCTATGAAAGTATCTTCCTCCTCTTGGACAAACTA
CATAATCAACCATATCTTTAAATTTCACAGTTGAAAATGTTGACAATGTTAGGTCACAAACA
CTTATTAGTGCTTCCCTTTCTTCATTGGATATTTCACTAAATCTTTCTAATTCCATTATAATCC
ACTTAGTATACCTAGTAAGTTCGTATGCACTAAAGATTAATTTAGACTGAAATAATCCTTGA
ATACCTATATCTCTATCAAATTTTGCTAGATTTATATGTGATTTCATTGGTGGATGTTTTCTAG
GGCCAGCACTAGTCAAAACCGTATCTCCTTTGTATATAGGTAATATGTTATGTGATAGCACT
CTGTGTTCTAATGCACCTAATAACGGAATTTCTTCTATATTAATAAAATTTAATTTTATTGGA
ATTCTTTCTTCTAATTTTATATTCATTGTCTTTTCTTTATTATTTCTTTCATCTACAATGGACCA
ATAAGAGACTAAGATTAACCCGGATCTCACTATTTCAATTTTTCTATTCTTTTTCCATACTTTG
TGCCACAATTTACCCATTTCTTCTTTGCTTACTAATTGCATAAAAGTAGATGAATTTTCTACTT
TACTGCATATTGTTTCTACATAGCTGAAAATTGACTCTTCATAATATTTACCAGCAATTCTTG
CTGAAAAACAATCTTTGAATGCAAAAACTAATTTATTTAAAAATTCTATTTTATTATTTTCTT

TTTTCATTTCAAATATCTTTTTCAAATTTTTATTTACAGTGTGATTCTCCAATATCCTTTCTACT
TTATCTGCTAATACTGTTCCTGTATCAAATCTTCCTCCTGTTAATGGGTACTGATTAGTTACAT
AATGTTCTACTTTAGTATTAAAATTTATTAATTCACCTATAAAAGTCTTTCCTTGATAAGATA
ATAAACTCTGTACATAGATTATCCTTTTTACATTAGACAATGAGTACCCAGAAATATGAGCA
TCAGGAACTGGAGTCATTGCATAACCATACATTGTATATGGTAGATACATAAAAGATAATAT
CAGTGATTTCACTTCATCTATTTCTCTGTTAAATACAAAAAAGTTAACTATGTTCTCATCTCTT
TTATCGACTAAATCATTTATTTCTTTTTTGTCAAGTTTTTCTTTTAATAATTCTTTCTGGTGTGT
GGCACTAAAACTTTTCAATGATCTATATCTATTATCTACCTTAAGATATGGAATCTGTTCAAT
TAAGCTAATCATTTCTTTTTTTGATATACACAAAAATCTATAATTTCTAATAATTGTTTGAATT
TTTACACTATCAGACCTGGCAGATGCAGAACCATAGCCAGAATCAATGAATTTTATTAATTC
TAATTCATCACCCCACATGGCACATAAATTAGGTGATATTCCCATCATCCTTTTATATGTAGT
ATCTACAATGGTATCTTTAAAATAATGCTCTTTCAACATGCTGCATCTGCTGTTTGTTACTTG
GGTTTGTGAAAGCTTTACAAGTAAGCCCATCTTTAACCCAGATAATTGTACATGTTTTATTAT
TTCATTAAGTTTATTATCTGTCATGTTTTCCATTTTAAAAACTGCACAAGAATCATCAGAATA
AGTTGTGGCTAAATATTTAGAAAGAGCAAGAGAACTCAACATATCTTCTAGCATAAGTTGTG
ATTGAATTCCCCATAAAGGACCAAACCATCCTTCAATGGCGCCTTTTTGACCTTTAGAATAAT
ATATTAAATCAGTAAAAGGTTCATCATTAATGACAAAAATATTAGAAAACAAATATACAAT
AGAAATCATATTTTCATATTCTTCCATCCCTTCTGTGTATCCATACATATTTGCTATTTCTTCT
ATTATAAACAAGGTATTTTCTGGTCTTTGTGATGTGTTATGTCCAGAGTAATCTAAAAATAAT
GAATAACAATTATCATCGGATATCATGGTGCTCATTTTGTACATTATATCTCTTCTTTCATCTT
CAGACATAGTCATCATTTGTCCTGGTAATAATTTCATTGCTCTCTTAATCCTTTCCATCATAG
ATGATATCCACAATTTGAGTCTAAAACTAGCAATACCAAAAAAGCGTGCAGCAGGTTTTAAT
TCTTTTTCTTTCTCAGCCAGTCTCACAATGAAGTATTTCCTCATTCTTTTCAATTCCTCATCTTT
TCTATCATAAACTTTAACCACTCTAGCTTGATTGCTGTGATACTCTTCTTTATTTACTTTTAAT
TCACTTGAAAAATCTCCATCAAAGTTATTTTTAATGTAGTAAATCAATTCCTTAATAGAGTTT
GTTTTTTCATCGTAATCATCTTTGGTGCAAGCCTTGTCTATTATTCGAGACTCTAAATTCATTT
CATCACCTTCTTCCAGCATCTTTCCTAACCTTACATCTTTATAATTAACAGGCTCTGATACTA
CAAGGTTATTATAATTTCCTCCAGCTGCCATTATTTGTAATTCTTGATCTAAGAATGAATTTG
AATTTAATAGCAGTGGAACCCTTCCATATTTTTTAATATAATTTCGGATCACATTCATCCTGA
ATAATCTCCTTAGTTTTTCAATCTGGTGTAAATCGACAGGATGATTTCTATTAGTCCGTTGAG
TATACTTGGTTAAACCTTGGTCTTTGGAAACTTCAGCATAAATTGTTGTTTTACCAATACACG
ACATCTTAATGCATGTCATTCCGTCTGATTCATTACATATTTTAATCAATTTTTCAGTGTTTTC
TGGTGATAGTTCATATGAGTCTAAAACATTGTCAAGAATTGGAAGAGCAGCTGCAGTTCCTT
TTGTGTCAGCCATCAGTAGGCAAGTTGACTCATACAAACTAGCCAATCCTGTTCTTTTCTTTG
TTTCAACGATCTGAGTTATAGACAATATGTAATCTATAAATTGTTTTTCCATTGGGGTTGCCA
TTTCCTTGATGAAATTCAAAGTAAATTCATGATCAATTATTGATATAATATATCTAATCATAG
AACCATCTCCAATGTTAATCGTTCCATTGCCATATGAGAATGCAGAAAAAGTTGATGAAATG
TACCATGTTGTCACAATACCTTCTCCTTCAATTTCCAGATCAATAATTGACTGTCCAGTACTG
TACACTTCAACTCTTATTCGCTTTCCTTCATTGTAATACAATTCGCTTTCCCTAGTTTGATTTT
GCATCTTCATTGACATGTTAATCTTGATTTTTCTATTCATAGTTTCTATAATATCTAATAGCCA

TGCAGACTTTATCACCTCCTCAGAAAATTCAGTTTTCATGCAAAAATTCTTTTTATTGAGTAG
TGATTCACTTAGCTGATTTAGTTCATGATCATAAAGATCAGCAGAAAAAGATAACAATTTTC
CAGCAGTGAGCGAAGGCCTACTCAGGGATCTTAATTTGTTCATTAATTTTATGCACATGTGTT
GGGAAGAACCATAATTTAGTTCTCCATGCCTTTCTTTCATTTCTGCTAAGTAACAATTTCTAA
GTATCCACAGGTCTTCTTTCATTTTCAATCCCCATTTTTCAGGGTATAAGTGAGTTTCTGTGA
ACATTTCAATGTTGTTGTCAGAGAATGATTTAAAGGGAGAGTTGATTTTTTCTAGCAATATTG
GTTTTGTTTTAATGTTCCTTTCAACCCATCGGAATCCAACAGTGTGATTAAATTTCATTTCAA
TCTTGACTTCCTCTGAACTAGGTTCCATCTCAGCTTCTTGCTTCCTTAATGACATTAAATCATA
ATGAAATTTCCCATATTTGTTCAGAATACTCTTGGTGTGAAAATGAGAATCATGAATGAGGT
TAAGTAGGGCGCTCATGATTACAATTTGAAGTAGCAATAATAATTTAAAATCCAGTTCTTAA
AATTAAGCTTATGGTTTGATAAACCAAATGATTTTTATTAAAACGAGAAACAATTAATTACT
AGAACTAGAAGAAGCTTTGCCCTTAAGTTTCTTCTTATCTTTTTTGCATAACCTACAAATTTT
CTTTTTAATGGGGCTTCGAATCACTGTCCTCTTCTTAACTCCAAACATCTTCATAATCGATTTC
AACTCATAATCAATTCTTCTACAAGTCAGATTAACATATTGGAGTTCTGGATTTTCTTCATCA
GTGTAATCAGATGTAATCAACTCAACTTTCCCTGAGTGATAAAGAATGAATTGATTTTCCAT
GCCCATTAGACGAGGTATCTTATTAAAGTCATCAACTGATCTAGCGTAAACTATCCTGAGTT
CTTCCGAGTCATCAGAACATGGAATTATTGTATCGTCGCAGATTACTAGTGCAATTTTACTCA
AATAATCATTTGTTTTGAATATCCACAATCCAAACAATAGAACCATTTTTGTATTTTCTTTGA
AGGCATTCCTTTCTTTAGCTATTTCTAGTAAATTGTTAAGCTCAGTTGTTTTTCTTCACAAAG
AGTTATTTCAATTTCTTTTGTCCATTCTGGGAATATATGAGGATCAACATAAATTCTTTCAAC
TTTCTTTAGACATTCAATTGACATTTTTTGATTGAAAATGAGAAAAAGTTTTTACTTTTTAAA
GTAAACCCACAGAGTTATCTCAATTTCTTTTGTCCATTCTGGGAATATATGAG
>Seq99 [organism=Xerochrysum ophiovirus_macra] RNA2 genomic sequence
TTTAACAATTAAACTGAATTCCAGTTCGGCGGAGGACAGGGGCGACTTTCCCACTCAAGGGA
TGGTACCAGGATCGCAATGATCAGCAACGTTGCTCTATAGATCTACATGGTGAGATGGTCGC
ATTGACTTGTATCCACTCATGATGATCTAAGAGACAACGGGGAATCTCCTTATTCCGAATAA
TTTAGTTTTTATTAAAACGAATACAAGGTGAGAATTGTAAACTTATCCTCTTCTAAGCTCACC
AGAGGGACCTTGTTTCATGAACCACATTCTACCATCCGATGTGTTGTCAATGACAAATTTAA
TTAAGTCTCCTTTCATTAAGTGAATAGTATCACCTTCATCAATCATCTTATCAATTATATTCTG
TTTTTTAAGTTGACTATATGAAATTAGATTTGGATTAACATTATTTCCGACAAACACATTGTT
CAGTTCAATTATTTCTCCACACAGATCCAATACTAATGAATTTATTTCAGTAACCCACATTTC
ACCTACTTTTGTTTTTCCTGAATGCCCTTTTAAGTGAATGAAGTGAAATGGAGCACCTGTATC
CAATATATAACCTATATCACTTGAATACACTTTTTTAGACCACTCTTCAAGATCAATCACACA
ATGTGGAATTAATTCTTCCATAGATTCCTTATCAATTTCATTAATTGTCGAAATTGAATTTCTT
CTTGGGACACTTGAATTTCTTTCAGCTACATTTTCTAACAAACCGTCTACAAATATTTCTTCA
GCTCTTTCTAACCGGTCTAATTTTTTCTCAGTTAATAAAGAAGCTTTTTCAAGCTTTTTTCCAA
TGTTCCTAAGCACAGATCCTGATTTAATTGTTTTAGGGTGACTTGTTGGATCAATATAAAACA
CTTTCGATCTTTCTATTTCATATGTTCCAGGAAAATAAGATGAATGAACATAAAAACCTATC
ATCATGGAAGCAAATGAACCAAATTTTGTTGGTTCATTATCAACTGTTGTTATTAATTTGATC
TTTTTAATATCAACTTTTGGAACAAAAAATGACATATTCATCTCAGACATTGACATTTCATTT

ATTGCGGCAGTAATACTAGCAATCACTTTGTTCTCCTTACTGCCCTTCATCCTTTCATCAATT
AGAGAGAATGTAATCATTTCTCCCTTATTTTTCGTTGGTCAAATAGAGGACAGTAAAGAAT
TTGTACTTTACTCAATCTCATATAAGGCTTATCAGCAACTTTAATTATTTTTTCAATCCAGAC
CAAACTTGAGCTAAAGAACTAACAGTAAATGATGCTTCACCTACTCTTCCTTTCAAGTTTAG
CTCTACAGGAGAAAATGCTGAATTTATTCTACCACAAGTCATTTTGCTAAATTCAGCAGCCA
TACATTGCACTTCAGGACTTAATGGCTCATCTCCTGAAAGACTGATTATTGTTCCTTCTCCAG
CAGGAAAAGTTTCATTTATCGCCTTTTCTAATGATATCCTTGTTTCTGATCGTTGAAGTTCCG
TGGGCAATGTTGTTGACCTCCTGAGTAGACGTATTTCAGAGTTGACAGATTCCATCGAATTG
TTTTCTGTTTAACAGCAAAGACGTTTTTCCTTTCAAAAGTAAACCATCGTTCCTTCTCCAGCA
GGGAAAGTTTCAT
>Seq100 [organism=Xerochrysum ophiovirus_macra] RNA3 genomic sequence
ATTTATGATGTAGCCATTGTTCCCTACTGAACCCGGAGTTTTATTAAAACGAAAGGCAGTAA
AGTAGAAATACTGCAAAGAAACTTACTTTGCATTACCAGCAACAACAACAAATTCTTTCCCA
TACATGTTCTTCAAGGTGTCAACAGTAATTGATGAAAAGTCACATGATGAATCGGTCAGATT
TTTATGGATTCTTGTTCCAGAGGGCCCAGTCATCCCAATCAAATAGGGATCCTTCAGAAAAG
CTTTGTTGTCTTCAAGAGTCAAATTATCAACAAGCTCAATTCTTCCTTTCATAGACAAGGAAA
ATACAATCGCAGTGGTTATTTTCCTGGTCAGGTTGGTCACTGATGGCTTAAAACCATTGAGT
GGATGCATCCGAAGCATTGCTTCAAAATCGGTTTGAACAGAAAGCAAGTAGTCTACAATGTT
GTAGGCTTTTTGTAGTTTAAGCCTTTCATTGTTTACCTTTTCAAGTTCTTCCATATTTTTCGGA
AGAGCTGGCATTGGGGCTCTCTCAAACTTTGAAGCAAGTACAGCATATTTCACTATCTTGTTT
CCTGCAACAGATAATTTGCACCTGGCACAATCAGCATCAGGAAGAGTAGCTGTATCAACTTT
CAAAAAGATTTTTGAGGAAACTTTTTTGTTGAAGTGCTTGATAAAAGCTGAGGAATTTCAT
TCATCATCTTTAACTTTCCATCCAATAGATGCTTTGAAATATAGCTTGGGATTGGTCTTAAAT
CTTTTTCATCAATGATCGGCAAACCACCTTGAACATAAACCATGACAGTACAAGCTCTCAAT
AAACCTTTTCCCTTTTTAATATTTCTAGCCATGACACTTTCTTTCATTTTCTCAACAACATTGA
CATTGAACATTCCATTAGATTTTTCATTGTTTCCTAGTGCCCTGTAAAGAATGGCATCAAGAG
GAAGAGTTCCAGCTCCAAGATACTCAAATAAGCTACCTTCTTCATTAGTATCTGCAAACTTA
AACACCTTAACTTCTAATGGTCCATAACCTTCTCCATCAGCAGGTAGCTCCTTGTAAAACTTA
TCAAGTTCTGCTTGAACTGCCTCAACATTTACTTCAAAGGAACGAGCAGCTAGTTTCGCCCTC
AGTTCTTCACCTTTAATTTCTTCATCAGTTTCTTCAAGTTCAGGGATTTCTTCATCATCATCAC
CTGTTTTGGTGTTAGAAGTGTTATCTCCTTCCTCTTCGTCAATGTCTTTATACATGAGTTTTTC
CGTTTCTTCGTCAACATACATAATATGATCTTTACTAGGAGTGAGAGAAAGTTCCTCAAGAG
TCCTGACATTGAGTTTAATTTCATCCCTATCTTTGTCAATGACTTTGAATTGTTCAAGTGTTTT
CCATTCGTCTTTACTCACTGTTTTCAGGTTCTTCTTCATTTTGTTGAGAATTTTGATGAGGTGT
GAAGCTTTGATGGTCGACATTTTACAGCAAGATGGAATTCAGCAAAAAAGTTTATAATTGAA
AAATTAA
>Seq101 [organism=Xerochrysum ophiovirus_visco] RNA1 genomic sequence
TTACCATTCAAACTCATCTTCATAATCACATCCTTCATTTTCTAAGAGTTTTAGATTTTCCTCA
TATGATCTGTTTCTGTTAGCCTCAATTTCTTCTTGAGTATTATCAGTGAATATCGGTATAGTG
GTTGGATCAATGACTTCTTCAGATATATTATACATTTTTTCTGTTTCCTTTTTCACATTTGATA

CTAGCATTGAAAAGAAATCTGGTATTGCATTTCCCTCATCATCAAATTCAGTGTTACTTTCTA
GACTTTCAACTATCTTCATTAATTCTGTTGAACTCAATTCAGGTTCATCACATACAGGTGTAT
CATCAATGTCAGCCCACTTTTCATTTTCAATTAGACTTCTCCTATATCTTGGCAAGAGGTTGA
ACTTTTCTTTTAATAATCTTATTCTATTTATTAGTAGTTTATAATCCATAATCTTATTTCTTGTA
GTTTTACCTTTATGCTTCATTAATTCATTCAGACTTCCTCGTCTCATCCCAGTTAGTAATATTA
AATTTTCCATTGCCTTACCTGATTTATAAGAAACACAAGCTATTTTCGCAATTTTGTGTGTTG
CATCAATAGCTTTTATTTCTTCTTTCCTCAATTCTGTCAAGTTTATTATGTCTATTAATGAATT
CAGTGAAACTTTTTTGTCTGATTCTTTTACAATTTTAATAAAAATTTCCCACAGTAATTTAAC
TTTTGTGTAATTTAATTTATTGTTGTCAATCTCCAAATGGATTTCTTCTAATTCTGATTTGTTA
AGATTATCAAAGGTTTTTATTGCTTTTACATAAGGAGTAGAAAGGATCTTGTTGTTGACTCTT
ACAAAAACATCATCAGAATATAAATCATTGTAAGTTTCTGTATATAAGCAATTATCATTTGA
AATAAATCTTGCTTCTCTGAATTCTAGCTTACTACATATTATTGGATTATTTTCTATAAATGTT
CCACTAAGAAATGCCATTGAATATTTCTCTACTTCTTCTTTGGTAGAAAAGTGTGAGTGAAA
AGATAATGGTACATGATTAACATCACTGTCTGGATCTAATATCATCTCACACAAAAAGTCAT
CTGATACTGTTTCCATTGTGTGGTCTAATATGTTTTCAGGTTCTCTAGGAAATGGTTTTAAAC
CTACCATGCATTTTATATTGTTAGTTAATCTTTCTAGTATTACTGAATCAGTAAATTTTCTTAC
TTTTAATGATCTTTCAAGAGTTTCATTACTTGGTGCTCCTTTTGGTTCAATGGTTAGGTAAAT
GATTCCTGGTGATGATAAGGTTGGAATCCTCAGGTTTAGTATTGTATTTGTCTCTAAAAAATT
TCTAAACTCTTCTGATTCAAATATTTCACATGTTGTATTGAATCTCATAATTACCTTTTTATTT
TGTTTTATAACTAATTCTATGAGAGAAATCAATTTTTCCTTAGTTCCAGTTAAGTGTGAAATA
TCTAATAAGAATATATCATTCTCTAAATATTTTACATATTCAAATTCATCAAGTGCATTCCAT
GATGCTTTTTTCTTTTCCATTCCTACTATTCTATTAATGGTATTATAACCATCTAGTCTTGATA
CTGAATGATGATTGATGTCTTTTTCAATCATTGCTAGATGAAAATCACCTCGGCCACCAAAT
AAATCAATTATTTTTTCGTCTAAATGTTGATTTATAAATTTTTCATTGCACAATAGACTCATT
AATCCTCTAGCTGAAGGATAGATATCTGACTTGGTAGGAGAAGATACATGTTTTGGTAAGCA
ATACATTGCAGCCATCATTTCATATCCTTTAATAGCAGCTTTCATTGCTTTTGTATTTTCTAAA
TCTTTAGGATGTACTATATGTGAATCAAAATCAATGAATCCATATTCAATATTTTTATCTTTC
ATTATTTCTTCTGATAGTTGTTCCTTATAATATTCTGTAATTCTTTCTTGTTTAAATTTCCTTCC
ATGCAATGAATTACATAATTCTTCACAAGCTGATTCTAGCTCATCAAATGAATAAGACAATA
CTGGCATTGCATTAATTAATAAATTGCATATTTTTTTATCTTTTATCCAAAAGTGATGTTCTCC
CTTTCTTATGTTGTTTAGTGCATTTCTTGAATTTATTTTTGTCAATTGTTTGTTTACTCTTATTA
TGTTTTCTGTTTCAGAAGCATCTTCAACTTTAAAAATCAGTCCACATGATATCACAAACAATT
TTAACAATGATATCCTATAACTGACTGAAAAACCACTTAATGCTAATTCCTTGACCCAAGAC
TTGGAGTCATGTTCTCTATGAAATAATTCATGATTGATTAAAGATCTGGTTATTGCCTTTAAG
GGTGTTTCGTTTCCTATTATGTTTAAATCTTTGTAATGATTATAGAATTCTTCGAAAAAGTAT
TCTTTAGAAGCAAAGTCATCTAAATTGGGGACTATCTTTTTAAGTGTTTGTAATGGAATAGTT
TCCGGGGAAATTATCAATTGATCTTCCATATAATTGTAGAGATTAATAAATGATGATTCATTT
TTTATAATTTCATTGGGTATGATAGCACTTTCAGATATGAATTTTCCTGCCAAGCATTCATCT
TTTGCTATAAAATTTGAGTATGATTTGTATATGAATGCTTTTTTCTTTACATTTTCAAATGGAA
TTAAGGTCATTGCTTTTTTCGACATTCCAATGTGTAGTTGATTTATTCCATGAAGATTGAAAG

TAACATCTTTAATTTCTTCTAAAATATCAGACCTGATACTCAAGGTTCTTAGTTCTCTTCTTGA
AGGTTGTAACAATCCCATGCAAACTTTTACTATAGTGATTAGATATTGTAAGTTCAAATTATT
GTCTTCTCCACCTGTAGAGAAAATGAGGCCTGTTATTCCTGAAACATCATATTGACCACTTAT
TCTATTTGAACTTAAATCACCGGTTCTGGGATTGAACCCTCCTGCAGAAGCTCTATGAAAAT
ATCTTCCTCCTCTTGGACAAACTACGTAATCAACTATATCTTTAAATTTTACAGTTGAAAAAG
TAGATAATGTTAAATCACAAACACTTACTAAGGCATCTTTTTCTTCGCTGGATATCTCGCTAA
ACCTTTCTAATTCCATCATTATCCATTTGGTATATCTGGTAAGTTCATATGCACTAAAAATTA
ACTTAGTTTGAAACAACCCTTGAATACCTATGTCTCTGTCAAACTTAGCTAGATTTATATGTG
ATTTCATGGGTGGATGTTTTTTGGGGCCAGAACTAGTTAATACTGTGTCTCCTTTGAATATAG
GCAATATGTTGTGTGATAAGACTCTGTGTTCTAAAGCTCCTAATAAAGGTATCTCTTCTATGT
TGATGAATTTTAATTTAATTGGGATTCTTTCTTCTAGTTTTATGTTCATTGTTTTTTCCTTATTG
TTCCTTTCATCAACAATAGACCAATAGGATACTAAATGTAAGCCAGACCTGATTATTTCTATT
TTTCTATTCTTTTTCCAGACTTTGTGCCATAATTTTCCCATTTCTTCTTTGCTTACTAATTGCAT
AAAAGTAGAAGAATTTTCTACCTTGCTGCAAATTGTTTCAACATAGCTAAAAATTGATTCCT
CATAATATTTACCTGCTATTCTAGCAGAGAAACAATCCTTAAATGCAAATACTAATTTGTTTA
AAAACTCTAGTTTATTGTTTTCTTTTTTCATTTCAAAGACCTTTTTCAAATTTCTATTTGTGGT
GTGATTTTCTAATATTCTTTCTACTTTGTCAGACAAAACCGTTCCTGTGTCAAATCTTCCTCCT
GTTAAGGGATATTGATTAGTCACATAATGTTCCACTTTTGTATTGAAATTTATTAATTCACCT
ATAAAAGTCTTCCCTTGATATGATAATAGACTTTGAACATAGATTATCCTTTTAACATTTGAC
AATGAATACCCTGATATGTGAGCATCAGGAATTGGGGTCATCGCATAACCATACATTGTATA
AGGCAGATACATAAAAGATAGTACCATGGATTTTACCTCATCTATTTCTCTATTAAAGACAA
AAAAGTTTGCTGTATTTTCATCTCTTTTATCAACTAGATCATTTATTTCATTTTCACTGATTTT
TTCTTTTAATAATTCTTTTTGATGTGTAACACTAAAACTTTTTAATGACCTATATCTGGTATCC
ATATTCAAATAAGGAACTTGTTCAACTAAGCTTATTATTTCTTTTTTGGATATGCATAGGAAT
CGGTAATTCCTGATTATGGTTTGAATTTTTACACTATCAGACCTTGCAGATGCTGATCCATAC
CCAGAATCAATGAACTTCACTAATTCTAATTCATCACCCCACATTGCACAAAGATTAGGAGA
AATTCCCATCATTCTTTTGTAAGTAGTGTCTACAATGACATCTTTGAAATAATGTTCTTTTAG
CATACTGCATCTGCTGTTTGTGACTTGAGTTTGTGAAAGTTTGACTAATAAACCCATTTTTAA
TCCAGATAATTGGACATGTTTAATTATTTCATTTAATTTATTATCTGTCATTTTTTCCATTTTG
AAAACTGCACAAGAATCATCAGAATAAGTAGTGGCCAAATACCTAGAAAGTGCAAGTGAGC
TTAACATGTCTTCTAGCATCAGTTGTGATTGTATTCCCCATATAGGACCAAACCAACCTTCAA
TTGCACCTTTTTGACCTTTCGAATAATATATCATATCAGTGAAAGGTTCATCATTGATGACAA
ATATGTTAGAAAACAGATATACAATAGAGATCATATTATCATATTCTTCCATTCCCTCAGTAT
AACCATACATGTTTGCTATCTCTTCTATTATAAATAATGTGTTTTCCGGCCTCTGTGATGTGTT
ATGCCCAGAATAATCTAGAAACAACGAATAACAATTTTCATCAGATATCATAGTACTCATTT
TATACATTATATCTCTTCTTTCGTCTTCAGACATAGTCATCATTTGTCCTGGTAATAATTTCAT
TGCTCTTTTCACTCTTTCCATCATTGATGATATCCACAGTTTGAGTCTAAAGCTTGCTATACC
AAAGAATCGTGCAGCTGGTTTTAATTCTTTTTCTTTTTCAGCAAGTCTTACAATAAAGTACTT
TCTCATCTTTTTTAACTCATCATCTTTTCTATCATACACTTTAACTACCCTTTCTTGATCATTGT
AATATTCTTCTTTATTTACTTTCAGCTCTCTTGAAAAGTCTCCATCAAAATCATTTTTTATATA

ATAAATTAACTCTTTTATGGAATTGGTTTTTCATCATAGTCATCTTTGGTACAGGCCTTGTC
AATTATTCTGGACTCAAGATTCATTTCGTCACCTTCTTCTAACATTTTTCCTAGTCTAACATCT
TTGTAATTCACAGGTTCAGAAACTATCAAGTTATTATAATTTCCTCCAGCTGCCATTATTTGT
AACTCTTGATCTAAGAATGAGTTTGAGTTCATGAGTAGTGGAACTCTTCCATATTTTTTAATG
TAATTACGAATTACATTCATTCTAAATAACCTCCTTAATTTTTCAATTTGGTGCAAATCTACA
GGATGATCCCTGTTTGTTCTTTGAGTGTATTTTGTTAAACCTTGATCTTTTGATACCTCAGCAT
AAATAGTGGTTTTACCAATACAAGACATTTTGATACATGTCATTCCATCTGATTCATCACATA
TTTTTATCAATTTTTCTGTGTATTCTGGTGATAATTCATATGAATCTAAGACATTATCAAGAA
TTGGTAAAGCAGCTGCAGTTCCTTTGGTGTCGGCCATTAATAAGCAAGTTGACTCATATAAT
CCAGCTAATCCACTTCTTATCTTTGTATCAACAATCTGTGTTATAGATAGGATGTAATCTATG
AATGGTTTTTCTGTTGGTTCAGCCATTTCCTTAATAAAATTTAAGGTGAACTCATGATCAATC
ATCGATACTACATACCTTATCATGGATCCATCTCCAATGTTGATTGTTCCATTCCCATATGAA
AATGCAGAGAAAGTTGATGAAATGTACCAAGTTGTTACAATACCTTCTCCTTCAATTTCTAG
ATCAATTATTGACTGACCAGTACTGTACACTTCAACTCTTACCCTCTTTCCTTCATTGTAGTAT
AATTCACTTTCCCTAGTTTGATTCTGCATCTTTACCGACATGTTTATTTTGATTTTCCTATTAA
TATTTTCAATAATGTCTAGTAGCCATGCAGATTTCATCACTTCTTCTGAGAACTCAGTCTTCA
TGCAACAATTCTTTTTATTGAGTAATGAATTACTTAGCTGATTTAATTCATGATCATAGAGAT
CAGCAGAGAATGAGAGAAGTTTTCCAGCAGTAAGAGAAGATCTACTAAGAGATCTCAGCTT
ATTCATCAGTTTTATACACATGTACTGTGATGAATTACAATTTAATTCTCCATGTCTTTCTTTC
ATTTCTGCTAAATAACAATTCCTAAGGACCCACAAATCCTCTTTCATTTTCAATCCCCATTTT
TCAGGATATAGATGGGTTGATGTAAACATCTCAATATTACTTTCAGAAAATGATTTAAAAGG
AGAATTGATTTTTTCCAATAAAATTGGCTTTGTTTTAATATTCCTTTCAATCCATCGAAATCC
GACAGTGTGATTAAATTTCATCTCAATTTTAACTTCTTCTGATTTCGGTTCCATGTCAGCTTCT
TGTTTTCTCAAAGATGTTAAGTCATTATAGAATTTTCCATACTTATTTAAGATATTTCTTGTGT
GAACATGGGAATCATGAATAGGGTTAAATAGGGCGCTCATGATTAAAATTATTAGCAATGA
TAATAACTTAATAGCCAGTTTGTTAATATAAACTTATGGTTTAATAAACCAAATGATTTTTAT
TAAAACGATGAGATTTAGTTACTAGAACTTGAAGAAGTCTTTCCTTTATTTTTCTTTTTATCTT
TTTTACATAATCTGCAAATTTTCTTTTTGATTGGGCTTCTTATTACTGTTCTTTTCTTAACTCCA
AACATTTTCATGATGGTTTTTAATTCATGGTCTATCCTTCTACAGGTTAAATTCACGTATTGG
AGTTCAGGATTCTCTTCGTCAGTATAATCAGATGTGATTAATTCCACTTTTCCTGAATGGTAT
AGAATGAATTGATTCTCCATACCCATAAGACGAGGTATCTTATTAAAATCATCGACTGATCT
AGCATAGACTAGCCTGAGTTCTTCAGAATCATCGGAACACGGAATTATTGTTTCATCACAAA
TAACTAGTGCAATTTTACTCAATAGATCATTTGTTTTGAAAATCCACAATCCGAATAAAAGA
ATCATTTTTGTGTTTTCTTTTAGAACATTTCTTTCTTTAGCTATTTCTAACAAGTTGTTAAGTT
CATTTGTTTTTTCCTCACAAAGAGTTATTTCAATCTCCTTTGTCCATTCAGGGAATATATGGG
GATCAATATAGGACTTTTCAACTTTCTCTAGACATTCAATCGACATATTTTAATTAAAATTGA
GGAAAAGTTTTTACTTTTTAAAGTAAACCCACAGAGTTATCTCAATTTCTTTTGTCCATTCTG
GGAATATATGAG
>Seq102 [organism=Xerochrysum ophiovirus_visco] RNA2 genomic sequence

CTTGTTTCCCTTTCGGAGGAGGACGTGGCCGACTAGGAGGTAGTACCAAGATCGCAATGATC
AGCAACATTGCTCTATAGATTTACATGGTGAGATGGTCGCATTGACTTGTATCCACTCTTATA
AATCTAAGAGACAACGGGGAATCTCCCTATTCCGAATATCCTAGTTTTATTAAAACGAATA
CGATGTGAAAATTATCCAATCATCCTCTTTGAAGCTCACCAGAGGCACCTTGTTTCATGAAC
CACATCCTACCATCGGACGTGTTATCAACAACAAATTTGATCAAGTCTCCTTTCATCAAATG
AATTGTATCACCTTCATCCATCATCCTATCAATTATTCCTTGTTTTTTAATTGACTATAAGAA
ATGAGATTTGGATTGATATTGTTTCCTACAAACACATTGTTCAGCTCAATTATTTCCCCACAC
AAATCTAATACTAGTGAATTTATTTCAGTAACCCATAATTCACCCATTTTTGTTTTTCCTGAA
TGCCCTTTTAAATGAATGAAATGAAATGGAGCACCTGTATCCAAGATATAACCTATGTCGCT
TGAATATGGTTTTCTAGACCACTCTTCAAGATTAATTACACAATGTGGAATTAATTCTTCTAC
AGATTCTTCATCAATTATAGAAGTAGAATTTCTTCTAGGAATATTGGAATTTCTTTCAGCAAC
ATTTTCTAACAAACCGTCTACAAATGTTTCCTCAGCTCTTTCTAATTTATCCAATTTCTTTTCT
GTTAATAAAGAAGCTCTTTCAAGTTTTTTTCCAATGTTCCTAAGCACAGACCCGGATTTAATT
GTTTTCGGGTGACTTGTTGGATCAATGTAAAACACTTTCGATCTTTCTACTTCATATGTGCCA
GGAAAATAGGATGAATGAACATAAAATCCTATCATCATGGATGCAAATGAACCAATCTTTGT
TGGTTCGTTATCAACTGTTGTTATTAACTTGATTCTTTTAATATCATTTTTTGGGACAAAAAA
AGACATGTTCATCTCAGACATTGACATCTCATTTATTGCAGCAGTGATACTAGCAATTACTCT
ATTTTCTTTACTTCCTTTCATCCTTTCATCAATTAGAGAGAATGTAATCATTTCTCCCTTATTT
TTGCGTTGATCAAACAATGGACAATAAAGAATTTGCACTTTACTCAATCTCATATATGGCTT
GTCAGAAACATTTACCATCTTCTTCAGTCCAGACCAAACTTGTGCTAGTGAACTGACAGTGA
AAGATGCTTCACCAACCTTTCCTCTCAAATTCAACTCTACTGGTGAAAAAGCTGAATTTATTT
TACCTCTAGTCATTTTGCTAAATTCAGCAGCCATGCATTGCACTTCAGGACTTAATGGTTCAT
CTCCTGAAAGGCTGATTATTGTTCCTTCTCCAGCTGGGAATGTTTCACTTATAGCCCTTTCAA
TTGATATTCGTGTTTCCGATCGTTGTAATTCCGTTGGAAGTGTTGTTGATCTCCTGAGCAATC
GTAATCCAGAGTTGACAGATTCCATCGAATTGTTTTCTGTTTAACAGCAAGAACGTTTTTCCT
TTCTAAA
>Seq103 [organism=Xerochrysum ophiovirus_visco] RNA3 genomic sequence
TGTAGCCATAGTTCCCTATTGACCTAGGAGTTTTATTAAAACGAAATGCAGTAGTATTGAAA
TACTGCAAAGAAACTTACTTTGCATTTCCAGCAACAACAACAAATTCCTTCCCATACATATTC
TTCAAGGTGTCAACTGTGATTGATGAAAAGTCACATGATGAATCGGTCAAGTTTTTGTGAAT
TCTCGTACCAGAGGGCCCAGTCATTCCAATCAAATAAGTGTCCTTCAGAAAAGCTTTGTTGT
CTTCAAGGATTAAATTGTCAACAAGATCAATTCTCCCCTTCATAGACAATGAAAATACAATT
GCAGTGGTTATTTTCCTTGTCAAGTTTGTCACTGATGGTTTGAATCCATTTAGTGGATGCATT
CGGAGCATTGCTTCAAAGTCGGTTTGAACAGAAAGGAGATAGTCAACAATATTGTAGGCTTT
TTGCAGCTTTAGCCTTTCATTGTTCACTTTTTCAAGTTCTTCCATGTTTTTTGGAAGAGCTGGC
ATTGGGGCTCTCTCAAACTTTGAAGCAAGAACGGCATATTTTACTATCTTGTTTCCAGCAACA
GATAGTTTGCATCTAGCACAATCAGCATCAGGTAGAGTAGCTGTGTCAACTTTCAAAAAGAT
TTTTTGAGGGAACTTTTTTGTTGAAGTGCTTGACAAAAGTTGAGGAATTTCATTCATCATTTT
GTGTTTTCCATCCAAAAGATGTTTTGATATGTAACTCGGTATTGGTCTCATATCTTTTTCATCT
ATTATTGGTAAACCACCTTGAACATAAACCATGACAGTACAAGCTCTCAACAAACCCTTTCC

CTTCTTAATGTTCCTGGCCATAGCACTTTCCTTCATTTTCTCAACAACAAAAACATTGAACAT
TCCACTTGATTTTTCATTCTTACCCAAAGCGCTGTAAAGAATAGCATCAAGAGGGAGAGTAC
CAGCTCCGAGATATTCAAACAAACTCCCTTCCTCATCAGTGTCAGCAAATTTGAAAACTTTA
ACTTCCATTGGCCCATAACCTTCTCCATCAGCAGGTAGTTCTTTGTAAAACTTGTCAAGTTCT
GCTTGAACTGCCTCAATATTTACTTCAAAAGAACGAGCAGCTAGTTTTGCTCTTAATTCTTCA
CCTTTTATTTCTTCATCATTTTCTTCTAATTCAGGGATCCTCTCCTCTTCTTCATCTTTTTTTGT
GTTAGAAACATTCCCTTCGTCTTCTTCATCTATGTCCTTGTACATGAGTTTTCCCGTTTCTTCA
TCTACAAACATAAGATGATCCTTGCTGGGAGTGAGAGCAAGTTCATCAAGAGTCCTGACATT
GATTTTAATTTCATCTCTGTCTTTGTCAATAACTTTGAATTGCTCAAGAGTTTTCCACTCTTCT
TTGCTCACTGTTTTTTGGTTTTTCTTGAACTTGTTAAGAATTTTGTTGAGGTGAGAAGCTTTGA
TGGTCGACATTCTGCAGCAAAATGGAATTCTATGAAAAAGTATATAATTGAAAAATTAAACC
TGGTTTTTCTTGAA
>Seq104 [organism=Zostera ophiovirus] RNA1 genomic sequence
CCAAAGCTATGAAGATTAAGCTTAATACAGCAAAGGGAGATTTTAATCAAATTTTTTCCCTT
TTCAGAGTCTCAATGACTGCTTTGACAAAAACAATGGCTATCACTCCTCATCTTCATAATCGT
CATCTTCTTCCCAACCGATTCCTCCTTCTTCCAGTAATGCTTTACAATCATCTATTGTCATATT
CTTATTTCTCTCTATTTCCTCATCTGTTAACTTAAACATACCAAATACGGAATCCCCCTCTACT
TCTTTAAACAATTTCTCTTTTTCGATTTCTTTTGAATGAATATCTAGTGCTTTAAACTTTACGT
TCATAATGTCAGATTGTCTCATACAATTAAACATTGTATCTTCTTCTTTATCATTTAGGAAGT
TAAGTATACCAGTTGACTCTATATCCGTTAATAATTTTGCTTCATGTTCATCTATTATATGCTT
TACTTTATCTCCTGTGTAGTAACTTATGTGATCTTCTATGTTATAATCTTTTGCTATTAACTTT
AGATCATATTTTTCATTTATCTTTAATAATCTATTGTAAATTAATTTATAATTAACTATTAAAT
TTTTTGTTGCTTTGAATTTATTTTTCAACAAAACATCTTTACTCAACATAGACAACTTTGTGAT
CTCCTTTAGTCCACGTGTTTGTATGCCATTCCTATAACATGTCACAGCATACCTTGCAGCTCT
TGATACTTCATTTATAGCTTTACCTCTTTGATCCTTAAATTTCTGTTGTCTAATTCTAGCTTTT
AATAGAGAAGTGGACACTTTTTCATCATTTTTAATGTTGTGGAAGTATGCTAACCAAAGACT
TCTAGTATTTAAAGAATTTTTAGTATTATTAATGACATTTAATGATAGATCTAAAAATTCATC
TGCGGAAATATCTTTACATCCATCTATTATATTTTTCCCTGTTAACAACAACTCTTTGCTCACC
TTTATTTCAGGAGTTAAGTCTGACCCTACATTTGATTTTAATGCTCTTGACTCTTCATAATATA
AATCATATAAATTATCTATCTTGTCAGTGGTATCACTAGTATCTAAAACATAATTATCTATAG
ACGGAATTATCAATTTATTCCTATACTTTTCAAATAAATTATTATCAATAAATGCTCTCTGCA
TTGCATTACATTGTGTTTCATCAAGTAACTTGGCTATTATATTCTCATTTATTTTGTATTCTTC
ATCTTTGTCTACTAGTAACATTTCAATCAGTTTTTCGTCAGATATGTGTTCTTCTGTATGGTCT
ATAACATTTTCATATTTTCTATTCAAAATATCATAATCTGTAATATTGTTTAAATCATTTGTTA
ACATTTCACTTAATAAGCTCCTGTGATAACCTTTTACACTACTTTCAAATTCTTGTGTTCTCTT
ATTAAAGTCTATCGTCAAATAAATTATACCAGGAGAATACTGATATGGAACATAGCAATTCA
TTTCTACATCCTTGATTTGATCATAAAACAATTTATCAAAGTATTTTATCATTGTATTTAATCT
TATGATAACTTTTTTCTTGAAAAACTTCATATCATCAATTATGTCTTTCAAACAATCTTTGGT
ATTAGTTACATGAGATGCATCTATTAAGAATATAGATGATCCTATATAATTCATATAATTATT
TCTATCAAATGAATTAAAATTTGATTTTTCTTCTCTCATACCTATGATCCTGTTTATAATGTTA

TACCCATCATTTCTTGATATCGAGATATGATCAACTTTTTCTTCAATCATTGCAAGATGAAAA
TCACCTCTGCCTCCAAATAAATCAATGATTTGGTCTTCACTTCTTATAAATCTATTATTTTTCA
ATGTTTGTATTAATGATATTGCAGTAGGATATATATCAGACATAGTTGGAGATGACAAATGT
TTAGGTCTCAAACTTAATGCACAAATCATCTCATAAGCTCTAACTGCAGAAGATAATCCCTT
TTTATCTACTAAATCTTTACTTGTCACACTTATTTCTCTAAACATTATATTTCCAAAAACATTG
TTATCATTCTTTTCAATGTAGTGATGGAACTCAGTAGAGAAGTATTCGTTGATCTTGTTTTGA
TGAATATTCATTTTATTTAACATTTCATATACAATGTGTACTTGTTCAGTGATTTCATATGGTT
CGTATGATAATATGGGCATCACATCAACAATTGCTCTAAATATCTTTTGATCTCTAGTATAAA
TTTGTAGCTTTCCAGATTTGAGACCTCGAATATTTTTCCTTGTATTTTCCAGTGTTCTTTTCTC
CATTATATGAAGTTTATAATTTCCATTATTAAGTGTATCAAGCTTATATATCATACTAGTTGC
CATAATGAATAAACGTAATACAATAAATCTCATATCATTACTATATCCTTTATCCATCAATTC
ATCAAACCATTTGGGTTGTAATGGATTTAAATACATGTGATCGTAAAGAATGGATCTGATAA
TACATTTTGAAGGAGTGTCACTTTCAATTATGTTCAATCCTGCATAGAACTTGTAAAATAAAT
GATACAAATTTTCCTTTGATCCGAATGTATCCTCTAAATTTGGTACTAACTTCCTAAGTGTCT
TTTCAGGTATTAAGCATACAGAGGATTCAAATGAGTTATCCATGTAACGTTTTATATTTATAT
ATGCTGTTTGTATTGTCATTTCAGTCTCACTACTACTTATCAGCTGGTTTATGAATGACTTTTC
TAAATCTTCATCTACTGCTATAAAATTGGAAAAAGTGAGATACAGTGGTCCCCTTTCTATCA
ATTCATTAACTTTGATTATGTTCGATTCTTTCAACTTCCGTAAAACACTTGGATAACCTATAT
TGAATAAATTGAAAGTAACATCTTTTGCATATCTCATAACATCTTCAGTTAGAGTAAGTCTTA
ATGCTTTATCTTTTCTTGTGTGAGTTATAGATAAACTAACTCTTAGATATGTCATCACATACT
GTAGATTTACATTATGATCTTTTCCTCCAGATTTCATTATAAAGTCTCTTATCCTACTCAAATC
CCATTTAGTAGTTTCCTTATTAGATGATAAGTCTCCAGTTTTAGGATTGAAATTTGCAGTTAA
TGCCCTATGAAAAAACCTTCCACCTTTTGGAGATGTCACATATTTTTCAAGATTGTAATAATT
TCCATGACTAAATGTTGACAAGGATCTATTACACACTTCTATTAAACACAATTTTTCATCATC
ACTTATTTTTGAATATTTCTTCAGTTCCATCAGTATCCATTTTGTATATCTAACTAAATCAAAT
GCCATAAATATTAACTTGTTTTTGAAGAAACCCTCAATTCCAATTTCTCTTTCAAATTTTGCC
AGATTTGTATGAGATTTTACAGGAGGTCTTGCACATATTTTATTATTGATTTTTTGAGTATTT
GGCACATTGATAGGTTGTATAATAGTATCGTGAGAAAATGAGACAAGCTTTCCCATAACCGG
TAATTCTTCTATGCCAATAAATTTCAATTGTATTATTTCATCATTCTTCATGTACTTCAGACAA
TGTTCTCTCGCTAGAAACAAATGATCATAATCTAGTATAGGATATTTGTTCAATTTCACCTTT
CTATTGTAATTAATAGACCAAGCTTTGTTCCTAATTTTATTTAGAATCTTACCTCCAAGCATC
ATTTTCATAGTACCAGAAGAATCTATCTTGTTCAATATTTCACTTATATAATTGAAAATTGTA
CATTCCTTGTATTTTCCTGTTATCCTGCAAGAAAAGCAATCCTTAAAAGTTCTCAACAACATC
ATATCAAACTCTTCTTCTGATTCATCATTATCAATGCTCATATATTTTAACAAATCTGGGTTTC
TTGTATATTCTTTTAACTTCAATTTTAATATATCATTTAATAATGTTTTAGTATCAACAAACCC
ACCTGACAAAGGGAAAGGTGTAGTCAAATATTGCCTGGCATTTGTTGATGGATTAATCAATT
TTCCAAACAGTTTTGAATTATCTCTCTTAAGGAATCCTTGTATATATGATATTCTCTTAACATT
AGATAATGAATATCCAGATATCAATGTGTCAGGCATAGGTGTTAGAGAATAACCGTAACAA
CTTGTTGGTGCATACATTAACATTAATAACAATTCTAAAATTCTAATATTCTTTCTATGAAAG
TGTATGAAGTTAACAGTATCATGTCTACTAGTTGGCAAAGACTTCATATCAAAATCAGTTGT

TAAATCTTTCGTTTGTGCCTTCAACAATAACATTTCTCTAACATAATTTTTGTTTGACACTAA
GAATCTATGATCTAATTCAACTTCTTCAGGAATTTTCAGTAAAAACCTTTTAAAGCTACTAGC
CAATAACTTTAGAACACGATAATTCCGTAATATTGTCTGAATATGGATTTCAGTGGACCGGA
AACAAGCAGAATTGTACCCTGAGTCTATCAATCCTGACATCTCTTGATCATCATATAATAAC
CCTGAACCATTGGGAGATATTGACATAAATTTCTTATAATTCGTTTCTATATATTCTTCATAT
GTATAATGCTCTTTCAACATGCTACATCTTCCATTGGTAACTTGAGTTTGTGACAACTTTACT
AGTAATCCCATATCTGAACCAAACCTTTGAATATTTTGAATTATATTATTCAACTTGTTTCTG
TCCATATTCTTCTGTACAAAAACACCACATGAATCATCAGAATAAGTTGTTGCTATATACTGT
TTCATCCCCATGTTTGAGAACATATCTTCAAGCATTAACTGTGATTGTATTCCCCATAATGAT
CCAAACCAGCCCTCAATAGCACCCTTTTGACCCTTTGAATAGTATACACTATCTCTCCATTTA
TCTTCAGAAATGACTGTAATATTAGAAAATAGATATGCTAATGATGTAAATTTTTCATAGAA
AGGTTCTCCAGAATAACTTCCATACATGTTTGCTATTTCTTCAAGTAAGAACAATGTATTCTC
AGGTCTCTGTGAAGTGTTGTGACCCGAATAATCTAGAAACAATGAATAAGAATCATCTTCAG
CTAATATCATTGACATCTTGTGCATTGTTTCTCTTCTTTTATCATCTGTCATAGTCATCATCTG
TCCTGGTAACAGTTTCATTGCTCTCTTGACAAACTCCATCATTGTACTAACCCACAATTTCAA
ATTAAAAGATGCTATACCAAAGTATCTAGCTTTTTCTTTTAGTTCCTTTTCTTTTTCACTAAGT
CTTACCAGAAAATGCTTTCTATGATTGAATAATATTTCGTCTTTTCTATCAACTATCTTGATGT
TTCTAGTCTCTTGATAATCTGAACTGAATATATACAATTCTTCTTCTCTAATTTTGCTTATATC
ATTTCCTATGTAATAACTTATCTCTTTAACTGTATTATTATGTGAATTATAATTATCTGATGTG
CAGGCTTTATCTATTATCCTTGTCTGTAAGTTGATTTCTTGACCTGGTTCTAAAAATTTTCCTA
ATGTCAAATTGTTATATAATGTCAGATCAGATATGATTAAATTAGAATAGTCTGAAGATGAT
GCCATCTTCTCCAGTTCATATGCAAGTGACTCAGGAACATCATATAATTTTGGAACAACTCC
ATATTTTTTGATAAACTCTATTATAACTTTCTTTCTGAATAATGATCTCAATCTTTTAATTGTG
TTCTCATTCACAGGATTTTTTTGCAATCCTCCCTCTGTGTCTGTTTCTGCCAATGACATCCTTA
TACATTGAGACACTGAACTTTGAAGACACAAATTAACAAGCTTTTCAGTATAAAATGGACTG
ACTTTTATAGATTCAATTAAATTATCCATTAAAGGCATAGAAGATGATTTCTTCCTATAATCA
GACATTAATAAACAAGTTTGCTCATACAAACCTGCCAGTCCTACTCTTTTGCTAGTTTCAGGA
ATATCTAATAATGATTTCATATATTCTAAAAAGCTCCTCTCGACATAACCTAGATTGATATCA
TTCATCATATTTAATGTCATCATGTTATCAGTAATAAACATAGTGTAACTTAACATTTCCAAT
CTGCCCACATGTACTATATCTTCATCTGCAAAATTGTACGATGAGATAACATTTGATATAAA
CATGTTCAAATTCATTTCAGGATAATCTTTGAATTCTACAAGAACAAGACTGTTTCCTGTAAT
ATGTGTTAATACATTTATTCTTTTACCTTCATAAAAGCTCACATTTCTTTTAATTTTCTTTCGA
TTCTCCCTTGAGCTACTGTTGATTTTTATCATTTTATTGATTGTTTCAAATATATCTAGTACCC
AATGGATTTTACATGATTCTTCTGACATCTCTATATTTTCACTAATATGATATTTGTCTTCCAT
ATTCTTCTCAGTCAAATGTTGTGATAAGTTGTCTGCTTCCATTGAATATAACATCTTTGACAT
TGTAAATATATCAGCACATTCGACTGAAGGTGAGGCTATTTTCATTAATCTCTTATACAAATG
GATAGCTGAATATTGATTATGATTATAATGATTTATAGGATGAAGTGATTTCATATCAGAAA
GATAAGCAGTTCTCCAATGATACAATGAAAATTGATCTTTCTTTGACCATTTTTCAGGATACA
GATTTCCTAACATCATTTCGATATTTTCATCCGATAAAGATTTATAAGGACTATCAATTTTTG
TAATCAAAGCAGGAGTACTCTCTTTAGTCAATATCCAATAATCAAAGTTCACATTTACCAGA

TGATTATGTTTTATGGGTATTTCAAATGATACTGGTTCAGATTCCATTTCATCCTCATTTCTTA
TAGAGCGATAAAGTTCAGAATAATGATCACTCATCTCTATCAGCAACTTGAACACGCAAGGA
TGTTGAAGCTCATTAATGTTATATCTTTCTAGCAAGTCACTTATGTTCGCAGAATGTTGGTCC
ATTGTTTTCTATTCAAATTTTAGTTTATTTAAAACCATTCTCATTAATTAACATGATCAAATTA
TCATTCAACAAACAGAAAATGATTTTCATGCAATTTCTTTAGAGTTGAATTTAACTTGTGCCT
TCTAGATCTAACTTTTCTCTTTCTTTTTCTTAGTGCACTCATTTCATCATCATCATGCATTAAC
TTCTCATATGGCAAATCAACAACTCCCCTAGTGTCTAAATAGATTGTAATTTCACAAGATGCT
TGAACTATCATATCACAAATAAACATGATATCTTTATGCTCAATCATCATCTTTCTGACATCA
TTTGTAATAAACACAGACATTTCTTCTATTTCGATCACTTGTGTATTGTAAAACATGCTATCA
ACCACTATCAAACCCATTGGATTCATAGGATAATCACGAATTTTATCATCTATTAACCATGAT
CCAAACAAGCACCTTGTTAATACTTTATTCGTGATAATCAGATTCTCTAAAAATTCCATATAC
CTTCGATTTCTCAGCTTTAAACTCTCATCAGCTTTCAAGTTACCCATGAACAGACCATCCAAA
GTGAAGAACTTTATATTGCGACAATCATAACACAATTCTTCAGTATGGTCAATTGGGTCAAC
AGATGAAATGTACATATCCGGAAGAGACATATGCACGAGAGGCCGATCAGCTAAATTGTTA
TAAATGAGATCGAGAGAGTCCATGATTTAGAGTGATCTGTTGACAGCGTAAATTACGTTTAA
GACCATCCAAAGTGAAGAACTTTATATTGCGACAATCATAACACAATT
>Seq105 [organism=Zostera ophiovirus] RNA3 genomic sequence
ATGTATCTTCTGACTGAAACACACAAAAAAAGTTTGTTTAAAGCCCCATCAAAACGCAGCAC
AAAATATCAGCAAAACAGACTAACAAACTGTTCATTGTTTACCCCAAACCCCTCTGATCGTT
TCAATTGACAAGCCACTGAAGTCAGCAACGCTCTCATCGAATACAGGATAAGTCCTTATATT
CGAATCAATGTCATTGCTGGTACTAATTCCCCAGAAATCGATGTCTTTCTTCCACGACAGAC
GATTTTCAGAGACGACACGAGATGCCATCTGTCTCAGACCATCTATTGAAAGGCTATAGAGA
ACAGCATGTGTCAATTTCTTCGTCAATCCAGGAAGAATGACTTTCGTTTCAGAGAAGGGATG
ATTCATACATTGTGCTTCAAAATCACTTTCTAAGCTTGCCAGAAAAGTCCTTATCTCAATAGA
AAATGCAATCTTTATATTGTCAGCCTCTAAATTCAGTCGATTGTGAGCAGGGGTAAACATAA
ATCTGTTTTCAAAACAATTCGCCATCGCAGCATACCGGATAACTCTATTACCAGCAACATTG
AGACGACAGCGAGCAACACAAATTGAAGGAAGAAGATTTGATATATCAGGAAACAGAAAA
GCACTACCAGGAAATTTCTTTAAACTCGCAGAAGAAAGCAAATTGGCTAATTCACCTTGGTT
CTTCAGCTCATTATGAAAAACAGTTTCACGGATAAACTTTGGTAACTGTTTTGTTTCATTGAG
CTGAGGAAGATAACCTTGATTATAGACAAACACAAAAGCAGATGTCAGTGCTTTGTGTCCAA
CAGAAATCTTGCGACAAATTTCAGCATCAGAAATGGTAAAAGTTCCATCAGGGTTCGGAGG
AAGAGTGCTAATATCAAAAGCGTCAATATTGCTTTTCATGAAATGCTTGAACAGTATAGCAT
CTATAACCCGAGTGCCAGCACCTATTAGAGACCGGAATGTGGATTCAGAGTTCTCAAAGAA
GACAATTTTCACCTCTTTCGGATCATATGTGTTATATTCACCAGGTATCTCTCTAAGACATTC
AATCAGAGAATCAGTGATATCATCTTTCCGAACTCCCAGGGCAACAGAGTTAAAGATCCTAT
TCAATTCTGCTACACTGACATTACGTGTACCAGATGAACTGCCAGCACTGAAAACTGATGAA
GCAGGTCTAGGATTAATAGCTGGAGCAGGAACAATGACTTCATCTCGAACATAATCGACATT
ACCATCTTTATCCCAAAGAACCAGTCTATTTCTCTCATTGACAGGAAGCAAGAAACTGACAG
CCAAATCTAGTTTCCTAGTTCTGACATATTTCCCATGAGCATCAACATCAAAAAATCCTCTTC
CCTTGAGAACTTCTCGACTTTGCTCATCTGTCAAGACTGAACGAGCGAGTGCTAAGAATTCA

TCAACGCTGAGACGTGAACTCTCCATTTCTTACAAATAAGTGACAATGGAAGAAGGCTGAA
AACTACAAAAAGAGCAGGAGTGTGAAACGAGAAGATCGGGAAAGTCTGTGAGAAGGGATT
TGTTCACAAGGTTGAAACTGAGCATACGCGCAAAAAGATATCA
