# Supplementary material for: Expanding the Repertoire of the Plant-Infecting Ophioviruses through Metatranscriptomics Data
Source: Viruses. 2023 Mar 25;15(4):840. doi: 10.3390/v15040840 (PMC10144540; doi:10.3390/v15040840)
Supplement: Supplementary file 1 [file viruses-15-00840-s001.zip › Supp Table S1.pdf]

Supplementary Table S1. Virus names, abbreviations and NCBI accession numbers of ophiovirus sequences used in this study

Virus name	Abbreviation	Accession number
blueberry mosaic associated virus	BlMaV	KJ704366- KJ704368
carrot ophiovirus 1	CaOV1	OM419178-OM419181
citrus psorosis virus	CPsV	MZ330078-MZ330080
Dactylorhiza hatagirea ophiovirus	DhOV	BK013328
freesia sneak virus	FreSV	MN365016
lettuce ring necrosis virus	LRNV	MW594439- MW594442
Mirafiori lettuce big-vein virus	MLBVV	AF525933- AF525936
ranunculus white mottle virus	RWMV	KY769680
tulip mild mottle mosaic virus	TMMMV	KY769675
pepper chlorosis-associated virus	PCaV	BDO47352-BDO47349
